# Kallopterolides A–I, a New Subclass of *seco*-Diterpenes Isolated from the Southwestern Caribbean Sea Plume *Antillogorgia kallos*

**DOI:** 10.3390/molecules29112493

**Published:** 2024-05-24

**Authors:** Jeffrey Marrero, Luis A. Amador, Ivan M. Novitskiy, Andrei G. Kutateladze, Abimael D. Rodríguez

**Affiliations:** 1 Department of Chemistry, University of Puerto Rico, 17 Ave. Universidad STE 1701, San Juan, PR 00931, USA; jeffrey.marrero2@upr.edu (J.M.); luisalberto.amador@upr.edu (L.A.A.); 2 Molecular Sciences Research Center, University of Puerto Rico, 1390 Ponce de León Avenue, San Juan, PR 00926, USA; 3 Department of Chemistry and Biochemistry, University of Denver, Denver, CO 80208, USA; ivan.novitskiy@du.edu (I.M.N.); andrei.kutateladze@du.edu (A.G.K.)

**Keywords:** *Antillogorgia kallos*, *seco*-diterpene, kallopterolides, gorgonian corals

## Abstract

Kallopterolides A–I (**1**–**9**), a family of nine diterpenoids possessing either a cleaved pseudopterane or a severed cembrane skeleton, along with several known compounds were isolated from the Caribbean Sea plume *Antillogorgia kallos*. The structures and relative configurations of **1**–**9** were characterized by analysis of HR-MS, IR, UV, and NMR spectroscopic data in addition to computational methods and side-by-side comparisons with published NMR data of related congeners. An investigation was conducted as to the potential of the kallopterolides as plausible in vitro anti-inflammatory, antiprotozoal, and antituberculosis agents.

## 1. Introduction

In the field of natural products chemistry cleavage of a ring with the addition of one or more hydrogen atoms at each terminal group thus created is often indicated by the prefix “*seco*-” [1]. One of the first *seco*-diterpenes to be described in the literature was isolated from the tobacco plant in 1984 [2]. Such Δ^11,12^ *seco*-cembrane stemmed from selective oxidative cleavage of one olefin double bond within the cembrane ring. In most cases, either a C–C double bond or a vicinal diol is ruptured by oxidative cleavage. Several types of *seco*-cembranes have been reported throughout the literature [3]. Within the cembrane family of natural products cleavage usually takes place at the Δ^4,5^, Δ^5,6^, or Δ^11,12^ position, with most such open-chain congeners having thus far been isolated from tobacco [4].

**Figure 1:**
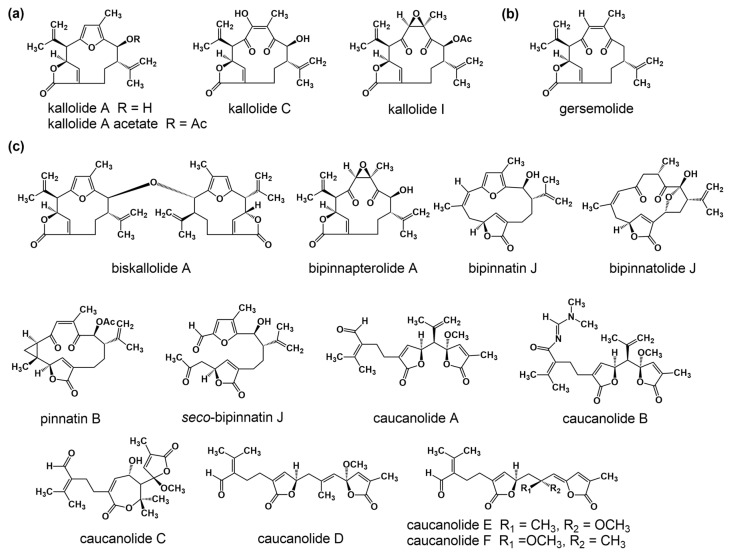
Structures of known secondary metabolites co-isolated during this investigation. (**a**) Pseudopterane diterpenes originally isolated from *Pseudopterogorgia kallos*. (**b**) Structure of gersemolide, a pseudopterane diterpene first isolated from *Gersemia rubiformis*. (**c**) Structures of pseudopterane, cembrane, *seco*-cembrane, and *seco*-pseudopterane diterpenes previously reported from *Pseudopterogorgia bipinnata*.

The sea plume *Pseudopterogorgia bipinnata* has been previously reported as being a prolific source of structurally interesting Δ^2,3^ and Δ^7,8^ *seco*-cembranes endowed with unusual functionality [5]. For instance, in 2000 our laboratory reported the isolation of *seco*-bipinnatin J (Figure 1), the first Δ^7,8^ *seco*-cembrane containing 4-furanal and α,β-unsaturated-γ-lactone moieties [6]. Subsequently, three additional members of the same family, caucanolides D–F (Figure 1), were isolated following a re-collection of the same sea feather [7]. The latter metabolites possessed two distinct α,β-unsaturated-γ-lactone moieties arranged in a linear fashion [5]. Interestingly, in the preceding 2005 investigation we also described a small subset of ring-opened compounds whose structures could not be rationalized as those stemming from a cembrane precursor but rather from a pseudopterane-based diterpene. Up until now, caucanolides A–C (Figure 1) represented the only family of marine natural products based on a Δ^2,3^ *seco*-pseudopterane skeleton. Like the Δ^2,3^ *seco*-cembranes, the Δ^2,3^ *seco*-pseudopteranes arise presumably from oxidative cleavage of the C-2/C-3 bond. Salient structural features of the latter compounds worth highlighting are the *N^1^,N^1^*-dimethyl-*N^2^*-acylformamidine and α,β–unsaturated-γ-hydroxy-ε-lactone moiety present in caucanolides B and C, respectively.

**Figure 2:**
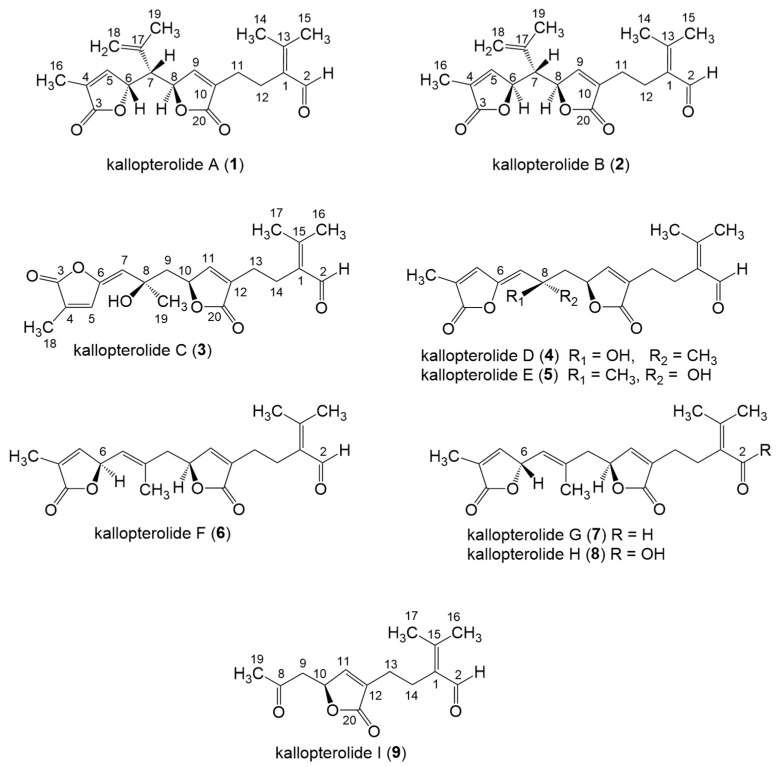
Chemical structures of kallopterolides A–I (**1**–**9**).

As part of our ongoing investigation of Caribbean marine invertebrates as likely sources of bioactive natural products, we recently investigated the chemical composition of the marine sea plume *Antillogorgia kallos* (previously described as *Pseudopterogorgia kallos*) collected in Old Providence Island, Colombia [8]. During this investigation, we isolated and characterized two new *seco*-pseudopterane diterpenes, which we have named kallopterolides A and B (**1** and **2**), along with ten *seco*-cembranes of which six are new, namely kallopterolides C–H (**3**–**8**), and four are known (caucanolides C–F, Figure 1). We also isolated and partially characterized a labile compound, which we named kallopterolide I (**9**). The latter congener is a C_14_ derivative that seems to arise from the further degradation of either kallopterolide F (**6**), G (**7**), or H (**8**). The chemical structures of all the natural products stemming from this investigation were established from the analysis of 1D and 2D NMR, IR, UV, and HRMS spectral data. A modern computational method was used to validate the assigned stereochemistry. After solving all their molecular structures, samples of pure compounds were screened in vitro for antimicrobial activity against *Mycobacterium tuberculosis* and *Plasmodium falciparum*. Some of the compounds isolated were also screened for anti-inflammatory activity. Figure 2 depicts the chemical structures of the title compounds.

## 2. Results and Discussion

### 2.1. Chemical Structural Analysis

Kallopterolide A (**1**) was obtained as a pure yellowish oil, $\alpha_{D}^{20}$ +20.0 (c 1.0, MeOH). Nine degrees of unsaturation were deduced from its molecular formula C_20_H_24_O_5_, established by HR-FAB-MS of the pseudomolecular ion [M + H]^+^ at *m*/*z* 345.1702 (calcd 345.1702). The IR spectrum of **1** indicated the presence of olefin (3093, 1660, 1626 cm^−^^1^), ester (1775, 1757 cm^−^^1^), and aldehyde (2858, 2772 cm^−^^1^) functionalities. The UV spectrum (MeOH) showed absorption maxima at λ_max_ 209 nm (ε 26,500) and λ_max_ 245 nm (ε 15,800) in accord with the presence of α,β-unsaturated-γ-lactone and α,β-unsaturated aldehyde arrays in **1**. The ^13^C NMR spectrum recorded in CDCl_3_ exhibited all twenty signals (Table 1), and a DEPT NMR experiment indicated the presence of four methyl, three methylene, and six methines in addition to seven quaternary carbon atoms. Furthermore, the ^13^C and DEPT NMR spectra of **1** indicated the presence of eleven sp^2^-hybridized carbon atoms in the molecule corresponding to four C–C double bonds between δ_C_ 156.8 and 117.1, two α,β-unsaturated-γ-lactone carbonyls at δ_C_ 173.8 (C, C-3) and 172.9 (C, C-20), and one aldehyde carbonyl at δ_C_ 190.6 (CH, C-2). Two oxygenated sp^3^ methine carbons at δ_C_ 79.8 (CH, C-6) and 80.1 (CH, C-8) were also identified. These combined ^13^C NMR data for kallopterolide A revealed four carbon–carbon and three carbon–oxygen double bonds accounting for seven sites of unsaturation. Thus, the remaining two degrees of unsaturation suggested that compound **1** must be bicyclic.

The ^1^H-NMR spectrum of **1** indicated the presence of two trisubstituted olefins [δ_H_ 7.06 (dd, 1H, *J* = 1.2, 1.0 Hz, H-5); 7.09 (dd, 1H, *J* = 1.6, 1.2 Hz, H-9)]; an isopropenyl group [δ_H_ 4.89 (br s, 1H, H-18α); 5.07 (br s, 1H, H-18β); 1.72 (br s, 3H, Me-19)]; and a vinyl methyl group [δ_H_ 1.92, (d, 1H, *J* = 1.7 Hz, Me-16)]. Furthermore, combined ^1^H-NMR and COSY spectroscopic data revealed signals consistent with the presence of an isolated pair of mutually coupled methylene groups [δ_H_ 2.52 (m, 2H, H_2_-12); 2.29 (m, 2H, H_2_-11)], three adjacent sp^3^ methines [δ_H_ 5.34 (ddd, 1H, J = 3.9, 2.0, 1.9 Hz, H-6); 5.16 (ddd, 1H, *J* = 10.3, 1.4, 1.0 Hz, H-8); 2.21 (dd, 1H, *J* = 10.0, 4.3, H-7)] and an α-substituted-β,β-dimethyl-α,β-unsaturated aldehyde [δ_H_ 10.10 (s, 1H, H-2); 2.21 (s, 3H, Me-15); 2.03 (s, 3H, Me-14)].

**Figure 3:**
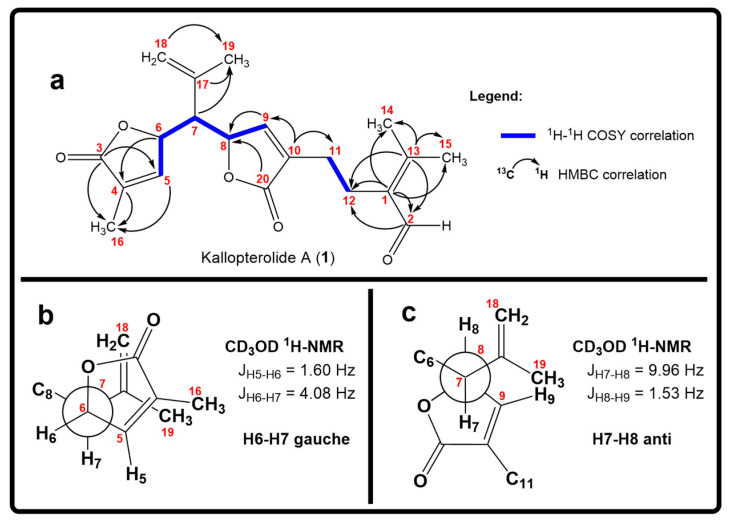
(**a**) Relevant ^1^H-^1^H COSY spin systems and HMBC correlations for kallopterolide A (**1**). (**b**) Newman projection for **1** alongside the C6–C7 bond. (**c**) Newman projection for **1** alongside the C7–C8 bond.

The first partial structure deduced from the 2D NMR spectral data was the α-substituted-β,β-dimethyl-α,β-unsaturated aldehyde functionality (Figure 3a). HMBC correlations of H-2 (δ_H_ 10.10, s, 1H) with C-1 (δ_C_ 135.5, C) and C-13 (δ_C_ 156.8, C) along with HMBC correlations of the latter carbon atoms with the vinylic methyls at δ_H_ 2.03 [s, 3H, Me-14] and 2.21 [s, 3H, Me-15] confirmed the presence in **1** of the trisubstituted α,β-unsaturated aldehyde moiety. The ^1^H-^1^H COSY spectrum showed a cross-peak between H-11 and H-12, which, together with key HMBC correlations between H-2 with C-12 and between H_2_-12 with C-2 connected the spin system –CH_2_-CH_2_– (C-11 to C-12) to C-1. These HMBC correlations validated the presence of a (CH_3_)_2_C=C(CHO)-CH_2_-CH_2_- fragment in **1**. Moreover, H-9 [δ_H_ 7.09, dd, 1H, *J* = 1.6, 1.2 Hz] showed a ^1^H-^1^H COSY correlation with H-8 [δ_H_ 5.16, ddd, 1H, *J* = 10.3, 1.4, 1.0 Hz] which in turn gave strong HMBC correlations with C-20 (δ_C_ 172.9, C) and C-9 (δ_C_ 147.4, CH). Further HMBC correlations of C-10 [δ_c_ 134.6, C] with H-9 and H_2_-11, combined with the intense IR absorption band at 1757 cm^−^^1^, vouched for the presence in **1** of an α,β-unsaturated-γ-lactone functionality (C-8 to C-10 and C-20) connected to the prior –CH_2_-CH_2_-(CHO)C=C(CH_3_)_2_ fragment through C-10.

The proton resonances at δ_H_ 5.07 [br s, 1H, H-18β]; 4.89 [br s, 1H, H-18α]; and 1.72 [br s, 3H, Me-19] and the ^13^C NMR signals at δ_C_ 137.8 (C, C-17), 117.1 (CH_2_, C-18), and 23.7 (CH_3_, C-19) were assigned to an isopropenyl group on the basis of HMBC correlations between H_3_-19 with C-17 and C-18. HMBC correlations from H_2_-18αβ and H_3_-19 to C-7 placed the isopropenyl group at the C-7 position. The presence of an additional α-substituted-α,β-unsaturated-γ-lactone unit was deduced from ^1^H NMR signals at δ_H_ 5.34 [ddd, 1H, *J* = 3.9, 2.0, 1.9 Hz, H-6]; 7.06 [dd, 1H, *J* = 1.2, 1.0 Hz, H-5] and ^13^C NMR resonances at δ_C_ 173.8 (C, C-3), 130.9 (C, C-4), 146.8 (CH, C-5), and 79.8 (CH, C-6). HMBC correlations from H_3_-16 with C-3, C-4, C-5, H-6 with C-4, and H-5 with C-3 corroborated the presence of the latter functionality. Interestingly, ^1^H-^1^H COSY cross-peaks between H-7 with both oxymethines H-6 and H-8 established the remaining spin system =CH-CH-CH-CH-CH= (H-5 through H-9). These combined ^1^H-^1^H COSY and HMBC correlations established the planar structure of **1** (Figure 3a).

**Figure 4:**
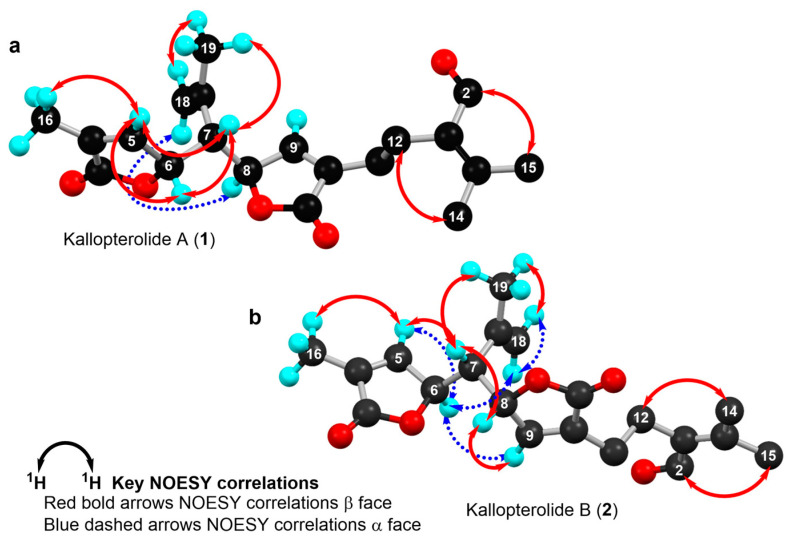
Computer-generated perspective views for the lowest energy conformers for (**a**) kallopterolide A (**1**) and (**b**) kallopterolide B (**2**). Some hydrogen atoms have been omitted for clarity.

The relative stereochemistry of **1** was established through a combination of 2D-NOESY (Figure 4a) and coupling constant (*J*) data analyses (Table 1, Figure 3b,c) in tandem with molecular modeling studies. This task was facilitated by the fact that the stereogenic centers within **1** are contiguous. First off, the ^1^H NMR spectrum of **1** was re-recorded in CD_3_OD (see Table S1 in the Supplementary Materials), which enhanced signal splitting and facilitated the *J* analysis of the pivotal proton H-7. The improved resolution revealed that the conformational flexibility of **1**, particularly alongside the C-6–C-7–C-8 bonds, was somewhat restricted, allowing some helpful conclusions to be drawn from these data. In point, the proton resonance ascribed to H-7, which occurs as a doublet of doublet, is coupled with both oxymethines H-6 and H-8. The magnitude of the coupling constant between H-7 and H-8 (*J*_H7–H8_ = 10.0 Hz) indicated that rotation along the C-7–C-8 bond is restricted and that these protons adopt an anti-periplanar arrangement (Figure 4a). If we place H-7 above the plane (β-configuration), then H-8 must have the α-configuration (below the plane). The absence of a NOESY cross-peak between these vicinal protons supported this contention. On the other hand, H-7 showed a NOESY correlation with H-6, which established their spatial proximity on the β face of the molecule.

This conclusion was validated by the small axial-equatorial coupling constant between these protons (*J*_H6–H7_ = 4.1 Hz). Furthermore, the fixed conformation depicted in Figure 4a was supported by the strong NOESY correlations observed between H-5/Me-16, H-5/H-7, H-7/Me-19, Me-19/H-18β, and H-8/H-18α. Farther along the eastern quadrant, the peak assignment for Me-14 and Me-15 in the ^1^H-NMR spectrum of **1** was based on the NOESY cross-peak between H-2 and Me-15. While these methods allowed us to establish the assignments described, we should point out that the relative configuration drawn for **1**, namely, 6S*, 7S*, and 8R*, correlates well with the known absolute configuration of other diterpenes co-isolated during this investigation (namely, kallolide A, kallolide A acetate, kallolide C, bipinnapterolide A, and gersemolide). This observation aligns with our contention that, most likely, **1** originates following oxidation/cleavage at C-2/C-3 of a suitable pseudopterane-based precursor (see Figure 1 and Scheme S1 in the Supplementary Materials).

Kallopterolide B (**2**), an optically active yellowish oil, ${\left[\alpha \right]}_{D}^{20}$ +5.0 (c 1.0, MeOH), showed a pseudomolecular [M + 1]^+^ ion peak at *m*/*z* 345.1696 in the HR-FAB-MS corresponding to a molecular formula of C_20_H_25_O_5_ (calcd 345.1702). The IR and UV spectroscopic data for **2** were very similar to those recorded for kallopterolide A (**1**). Further examination revealed that their ^1^H and ^13^C NMR data in CDCl_3_ were also almost identical, indicating that both compounds possess identical functionality, namely, two α,β-unsaturated-γ-lactones, one α-substituted-β,β-dimethyl-α,β-unsaturated aldehyde, and one isopropenyl group. Therefore, we concluded that these compounds must be diastereomers. A detailed side-by-side comparison of the ^1^H NMR spectra of **1** and **2** (Table 1) revealed that the minor differences observed could be explained by inverting the configuration in **2** at C-6. The reversal at C-6 from S* in **1** to R* in **2** was rendered by subtle differences in the ^1^H NMR chemical shifts and coupling constants for H-6 [δ_H_ 5.34 (ddd, 1H, 3.9, 2.0, 1.9 Hz) in **1** vs. δ_H_ 5.05 (dd, 1H, 1.7, 1.6 Hz) in **2**] and H-7 [δ_H_ 2.21 (dd, 1H, 10.0, 4.3 Hz) in **1** vs. δ_H_ 2.60 (dd, 1H, 7.0, 7.0 Hz) in **2**] (Table 2).

**Figure 5:**
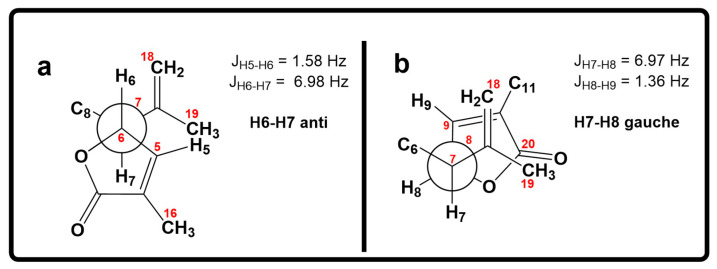
(**a**) Newman projection for **2** alongside the C6–C7 bond. (**b**) Newman projection for **2** alongside the C7–C8 bond.

Moreover, the coupling constant values between H-6/H-7 (*J*_H6-H7_ = 7.0 Hz) and H-7/H-8 (*J*_H7–H8_ = 7.0 Hz) [9] (Figure 5a,b), combined with molecular modeling studies and key NOESY correlations between H-6 and H-9 and between H-6 and H-18α (Figure 4b) suggested that these proton pairs lie within spatial proximity toward the α face. Interestingly, the chemical shift of H-6 (δ_H_ 5.05) in **2** appears upfield when compared to that of H-6 (δ_H_ 5.34) in **1**. This shielding albeit small can be explained by the proximity of H-6 to Δ^17^ (anisotropic effect). Conversely, when H-6 has the opposite equatorial-like orientation (as in **1**) the olefin functionality and the latter proton lie too far away from each other (Figure 4b).

Given that kallopterolides A (**1**) and B (**2**) possess very similar NMR spectra, we sought additional computational support for our stereochemical assignment. For this, we used a machine learning-augmented DFT method, DU8ML, which in the past proved both fast and accurate for natural products of this size [10,11]. As shown in Table 3, calculated chemical shifts alone are not sufficient to differentiate between the potential diastereomers. This was not unexpected, given that the actual experimental-experimental RMSD value for ^13^C NMR shifts of the two compounds is a mere 0.38 ppm. We, therefore, have included all three parameters, i.e., RMSDs for the ^1^H-^1^H spin–spin coupling constants, ^1^H chemical shifts, and ^13^C chemical shifts, presented as triads in Table 3, e.g., {1.73/0.19/1.49}. Analysis of the four diastereomers reveals that the most important differentiating factor is the calculated proton spin–spin coupling constants, with RMSD values for the wrong diastereomers exceeding 1.5 Hz. The two correct diastereomers have shown good matches across all three calculated RMSDs. For details, see Scheme S2 in the Supplementary Materials.

**Figure 6:**
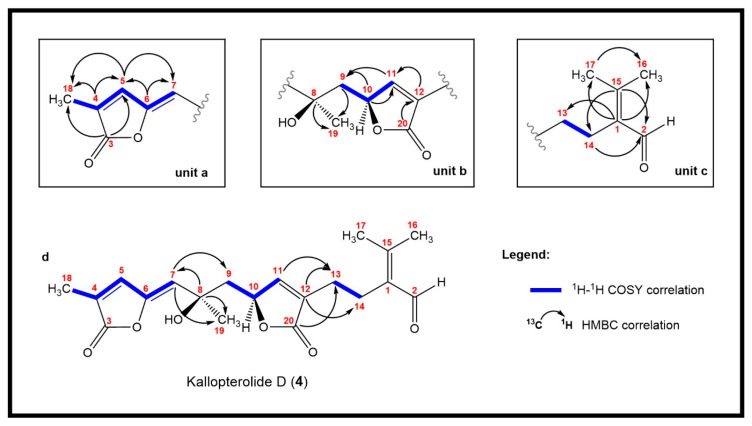
Top: Key HMBC correlations required for the assembly of partial units (**a**–**c**). Bottom: ^1^H-^1^H COSY and HMBC correlations required to interconnect units (**a**–**c**), thus yielding the complete planar structure (**d**) for kallopterolide D (**4**).

Kallopterolide D (**4**), ${\left[\alpha \right]}_{D}^{20}$ –10.0 (*c* 0.9, CHCl_3_), was isolated as an optically active yellowish oil. The molecular formula C_20_H_24_O_6_, deduced from HR-FAB-MS analysis of its pseudomolecular ion (*m*/*z* [M + H]^+^ 361.1652, calcd for C_20_H_25_O_6_ 361.1651), required nine sites of unsaturation. The IR spectrum of **4** indicated the presence of hydroxyl (3461 cm^−1^), aldehyde (2870 cm^−1^), ester (1775, 1752 cm^−1^), and olefin (3090, 1663, 1625 cm^−1^) functionalities. The UV spectrum (MeOH) showed maxima at λ_max_ 210 nm (ε 16,800) and λ_max_ 263 nm (ε 19,800). The ^13^C NMR spectrum displayed twenty signals (Table 4), of which eight were olefinic and three were carbonyl carbon resonances, suggesting that compound **4** was also bicyclic. Interpretation of the 1D and 2D NMR spectra revealed the presence in **4** of the following fragments: –CH_2_–CH_2_–C(CHO)=C(CH_3_)_2_ (C-1, C-2 and C-13 through C-17) (see Figure 6c) as well as a tertiary methyl carbinol linked to a α,β-unsaturated-γ-lactone through a methylene carbon (C-8 through C-12 and C-19 through C-20) (see Figure 6b). The connectivity between the C-9 methylene bridge and the internal γ-butenolide was accomplished from ^1^H-^1^H COSY correlations between oxymethine H-10 [δ_H_ 5.15, ddd, 1H, *J* = 8.2, 4.2, 2.6 Hz)] with H_2_-9αβ [δ_H_ 2.11 (dd, 1H, *J* = 14.6, 4.3 Hz) and 1.92 (dd, 1H, *J* = 14.6, 8.3 Hz)]. Key HMBC correlations between H_2_-9αβ with C-8 (δ_C_ 71.8, C), C-10 (δ_C_ 80.9, CH), and C-19 (δ_C_ 30.1, CH_3_) allowed us to complete the assembly of unit **b**. Further analyses of the 2D NMR data revealed the presence of a relatively unusual 5-ethylidenyl-3-methyl-2(5H)-furanone functionality (C-3 through C-7 and C-18) (see Figure 6a). The presence in **4** of subunit **a** was deduced from the carbon resonances at δ_C_ 170.0 (C, C-3), 129.3 (C, C-4), 138.6 (CH, C-5), 146.9 (C, C-6), 118.3 (CH, C-7), and 10.5 (CH_3_, C-18), and the proton signals at δ_H_ 7.05 (dd, 1H, *J* = 1.5, 1.0 Hz, H-5), 5.43 (s, 1H, H-7), and 2.01 (s, 3H, H_3_-18). The following key HMBC correlations supported this contention: H-5/C-4, C-6; H-7/C-5, C-6; and H_3_-18/C-3, C-4, C-5. Additionally, the presence of strong absorptions in the IR (1775 cm^−1^) and UV spectra [λ_max_ 263 nm (ε 19,800)] of **4** supported our conclusions. Finally, strong HMBC correlations between H-7 with C-8, C-9, and C-19 secured the connectivity between partial units **a** and **b**. In turn, units **c** and **b** were connected through HMBC correlations between H-13 with C-11, C-12, and C-20, and those between H-14 with C-12. These combined data established the structure of kallopterolide D (**4**) devoid of all stereochemical elements.

**Figure 7:**
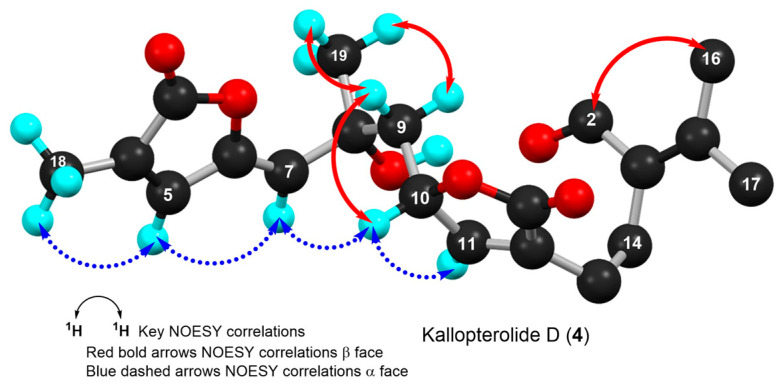
Computer-generated perspective view for the lowest energy conformer of kallopterolide D (**4**) showing important NOESY correlations. Several hydrogen atoms have been omitted for clarity.

In all, three stereogenic centers are present in kallopterolide D: the two asymmetric carbon atoms at C-8 and C-10 and the Δ^6^ trisubstituted double bond. From the outset, we realized that the relative stereochemistry of kallopterolide D was going to be difficult to ascertain given the acyclic nature of its structure as well as the non-adjacency of its two chiral carbons [12]. It should, therefore, be noticed that, except for the Z-configuration assigned to Δ^6^ (vide infra), the 8R* and 10S* configurations depicted in **4** should be taken as tentative. First off, since the 10S absolute configuration for bipinnatin J (see Scheme S1 in the Supplementary Materials), a likely biogenetic precursor to **4**, has been established by asymmetric synthesis, we assigned the S* configuration at C-10 in **4** [13]. Interestingly, after conducting a series of molecular modeling studies and 2D-NOESY experiments we envisioned that kallopterolide D has the propensity to adopt the S-shaped conformation shown in Figure 7.

Strong NOESY correlations between H-7 with both H-5 and H-10 quickly established the Z geometry about the Δ^6^ olefin and vouched for our assignment for the 10S* relative stereochemistry in **4**. Furthermore, the conspicuous absence of NOEs between H-10 and Me-19 and between H-7 and H_2_-9αβ, combined with strong NOEs between Me-19 and H_2_-9αβ, all argued for the 8R* configuration. Additional validation for the proposed S-shape conformation of **4** stems from the multiplicity and coupling constant values for H-10 (ddd, *J*_H-9α/H-10_ = 4.2 Hz, *J*_H-9β/H-10_ = 8.2 Hz, and *J*_H-10/H-11_ = 2.6 Hz) as well as the supplementary NOESY correlations depicted in Figure 7. These arguments notwithstanding, the shown stereochemical assignments for **4** (8R*,10S*) are subject to confirmation.

Kallopterolide E (**5**) was isolated as an optically active yellowish oil, ${\left[\alpha \right]}_{D}^{20}$ –11.4 (c 0.7, CHCl_3_). HR-EI-MS of **5** showed a molecular ion [M]^+·^ at *m*/*z* 360.1573 appropriate for a molecular formula of C_20_H_24_O_6_. The IR and UV spectra were quite like those recorded for kallopterolide D (**4**). The 1D NMR data (Table 4) and subsequent analysis of 2D NMR data suggested that **5** possessed the same partial structures and identical interconnectivity as those of **4**. Thus, we concluded that kallopterolides D (**4**) and E (**5**) are diastereomers. Following side-by-side comparisons of their ^1^H and ^13^C NMR spectra, we quickly realized that the minor spectral differences observed were ascribable to a change in relative stereochemistry in **5** at the C-8 position. Specifically, we argue that the change at C-8 from R* in kallopterolide D (**4**) to S* in kallopterolide E (**5**) could be inferred from subtle differences in the ^13^C chemical shifts of C-7 [δ_C_ 118.3 (CH) in **4** vs. 119.2 (CH) in **5**] and C-19 [δ_C_ 30.1 (CH_3_) in **4** vs. 28.9 (CH_3_) in **5**]. Furthermore, the small differences observed in ^1^H NMR chemical shifts and coupling constant data for H-7 [δ_H_ 5.43 (s, 1H) in **4** vs. 5.37 (s, 1H) in **5**], H-9α [2.11 (dd, 1H, *J* = 14.6, 4.3 Hz) in **4** vs. 2.40 (dd, 1H, *J* = 14.7, 3.5 Hz in **5**], and H-9β [1.92 (dd, 1H, *J* = 14.6, 8.3 Hz) in **4** vs. 1.85 (dd, 1H, *J* = 14.6, 5.3 Hz) in **5**] also supported this conclusion.

**Figure 8:**
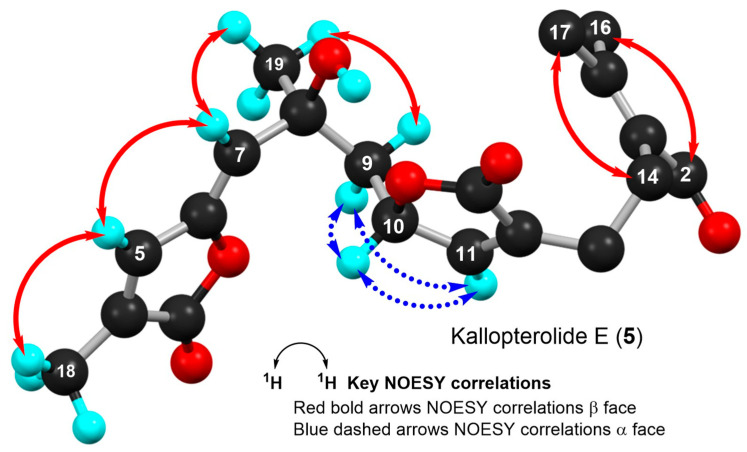
Computer-generated perspective view of the lowest energy conformer for kallopterolide E (**5**) showing important NOESY correlations. Several hydrogen atoms have been omitted for clarity.

As in **4**, molecular modeling analyses in combination with 2D-NOESY experiments indicated that at 20 °C, a solution of kallopterolide E (**5**) in CDCl_3_ does not adopt a linear conformation either. Instead, compound **5** attains a more stable S-shape conformation (Figure 8). As we saw before in **4**, strong NOESY correlations were observed in **5** between H-5/H-7 and H-5/H-18, which strongly argued for the Z-geometry of Δ^6^. This time, however, and contrary to what was observed for kallopterolide D (**4**), a strong NOESY correlation between H-7 and H-9α combined with the conspicuous absence of NOESY cross-peak between H-10 and H-7 clearly upholds the 8S*, 10S* configuration shown in kallopterolide E (**5**) (Figure 8). The proposed change in relative configuration at C-8 is consistent with the variations observed in coupling constant values for H-10 (*J*_H-9α/H-10_ = 3.4 Hz, J_H-9β/H-10_ = 5.1 Hz, *J*_H-10/H-11_ = 1.7 Hz). As with **4**, the 8S* and 10S* stereochemical assignments for kallopterolide E (**5**) are subject to confirmation.

**Figure 9:**
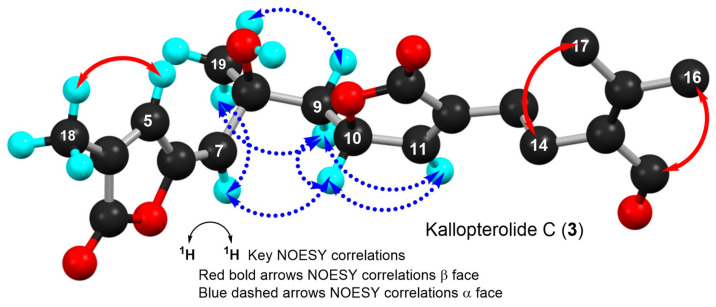
Computer-generated perspective view of the lowest energy conformer for kallopterolide C (**3**) showing important NOESY correlations. Several hydrogen atoms have been omitted for clarity.

Kallopterolide C (**3**) was isolated as an optically active yellowish oil, ${\left[\alpha \right]}_{D}^{20}$ +12.5 (c 0.4, MeOH). The LR-EI-MS of **3** exhibited its molecular ion [M]^+·^ at *m*/*z* 360, appropriate for a molecular formula of C_20_H_24_O_6_. However, attempts to measure the exact mass of **3** using HR-MS techniques (HR-EI-MS, HR-FAB-MS and HR-ESI-MS) failed to secure this information. Interestingly, the IR spectrum of **3** was quite similar to those of kallopterolide D (**4**) and kallopterolide E (**5**). Side-by-side comparisons of the 1D-NMR (Table 4) and 2D-NMR spectroscopic data of **3** with those for stereoisomers **4** and **5** quickly revealed the presence in **3** of the already familiar partial structures **a**–**c** (devoid of relative stereochemistry) previously remarked in Figure 6. On the other hand, when the UV spectra in MeOH of these stereoisomers were compared, **3** revealed a subtle hypsochromic effect (λ_max_ 257 nm for **3** vs. 263 nm for **4**), suggesting a change in the geometry of kallopterolide C (**3**) about the 5-ethylidenyl-3-methyl-2(5H) furanone functionality [7]. Careful comparisons of the ^1^H- and ^13^C-NMR spectra of these compounds supported this contention [14,15,16,17]. For instance, the change in geometry at Δ^6^ from Z in kallopterolide E (**5**) to E in kallopterolide C (**3**) was clearly implied by the differences in the ^13^C chemical shifts of C-4 [δ_C_ 129.5 (C) in **5** vs. 131.3 (C) in **3**], C-5 [δ_C_ 138.5 (CH) in **5** vs. 136.5 (CH) in **3**], C-6 [δ_C_ 146.0 (C) in **5** vs. 149.5 (C) in **3**], and C-7 [δ_C_ 119.2 (CH) in **5** vs. 117.4 (CH) in **3**]. Additionally, differences observed in the ^1^H NMR chemical shift and coupling constant data for H-5 [δ_H_ 7.02 (dd, 1H, *J* = 1.5, 1.0 Hz) in **5** vs. 7.88 (q, 1H, *J* = 1.5 Hz) in **3**] and H-7 [5.37 (s, 1H) in **5** vs. 5.62 (s, 1H) in **3**] plus the conspicuous absence of NOESY correlations between H-5 and H-7 firmly established the E geometry for Δ^6^ in **3**. As in **5**, extensive molecular modeling studies of the lowest-energy conformation shown in Figure 9 combined with 2D-NOESY experiments allowed us to assign the remaining relative stereochemistry for **3** as 8S*, 10S*. Only with the shown configuration could we explain the coupling constant values for H-10 (*J*_H-9α/H-10_ = 2.8 Hz and *J*_H-9β/H-10_ = 10.3 Hz) and the most salient NOESY correlations observed for kallopterolide C (**3**) (Figure 9).

The results of DU8ML calculations for the potential candidate structures of kallopterolides C (**3**), D (**4**), and E (**5**) are presented in Table 5. The fact that C-8 is quaternary presents an additional challenge of stereochemical assignment, as the ^1^H-^1^H spin-spin coupling constants are not informative. In this case, the assignment was solely based on ^13^C data. Two unambiguous matches were identified: kallopterolide C (**3**) as the SS-E isomer, RMSD (δ_13C_) = 1.23 ppm, and kallopterolide E (**5**) as the SS-Z isomer, RMSD (δ_13C_) = 1.12 ppm. The stereoconfiguration of kallopterolide D (**4**) was then confirmed as SR-Z, i.e., as the remaining choice between the SR-Z and SR-E stereoconfigurations.

**Figure 10:**
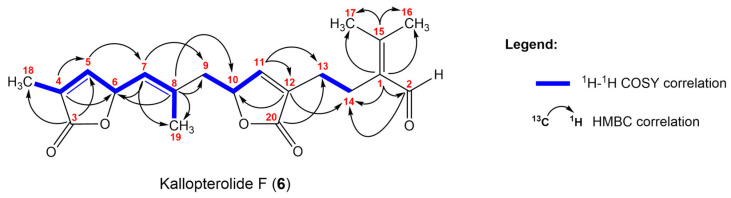
Selected ^1^H-^1^H COSY and HMBC correlations for kallopterolide F (**6**).

Kallopterolide F (**6**) and kallopterolide G (**7**) were isolated as optically active yellowish oils with similar optical rotations and spectroscopic data. The HR-MS analysis of each compound suggested the same molecular formula of C_20_H_24_O_5_, which indicated nine degrees of unsaturation. The IR, ^1^H, and ^13^C NMR spectra indicated the presence of olefin, aldehyde, and ester functionalities. Careful analysis of the ^1^H, ^13^C (Table 6), DEPT-135, HMQC, ^1^H-^1^H COSY, and HMBC (Figure 10) revealed the presence of partial structures –CH_2_–CH_2_–C(CHO)=C(CH_3_)_2_ (C-1 to C-2 and C-13 to C-17), α-methyl-α,β-unsaturated-γ-lactone (C-3 to C-6 and C-18), and α,β-unsaturated-γ-lactone (C-10 to C-12 and C-20) as in kallopterolides C–E (**3**–**5**).

The difference of sixteen mass units in the molecular formula of **6** and **7** suggested that the tertiary hydroxy group in kallopterolides C–E (**3**–**5**) must have been replaced by a trisubstituted alkene across C-7/C-8. This observation was corroborated by the absence of a broad IR absorption band ascribable to hydroxy functionality in the IR spectra of kallopterolide F (**6**) and kallopterolide G (**7**). The presence of a distinct trisubstituted olefin in kallopterolide F (**6**) was inferred by the proton signals at δ_H_ 5.08 (dd, 1H, *J* = 8.6, 1.0 Hz, H-7) and 1.87 (d, 3H, *J* = 1.0 Hz, H_3_-19), combined with carbon resonances at δ_C_ 122.3 (CH, C-7), 138.1 (C, C-8), and 18.1 (CH_3_, C-19). The –CH=C(CH_3_)CH_2_– (C-7 to C-9) connectivity was established by key HMBC correlations: H_3_-19/C-7, C-8, C-9; H-7/C-8, C-19; H-7/C-9 (Figure 10).

Key HMBC correlations between H-6 with C-8, H-7 with C-5, C-9, in addition to those of H-10 with C-8, C-9 connected the α-methyl-α,β-unsaturated-γ-lactone (C-3 to C-6) at C-7, and the α,β-unsaturated-γ-lactone at C-8 (C-10 to C-12 and C-20). Concurrent ^1^H-^1^H COSY experiments distinctively indicated the coupled proton spin systems across all these substructures. Careful evaluation of the overall 2D NMR data recorded for kallopterolide F (**6**) and kallopterolide G (**7**) demonstrated that these compounds shared the same planar structure.

**Figure 11:**
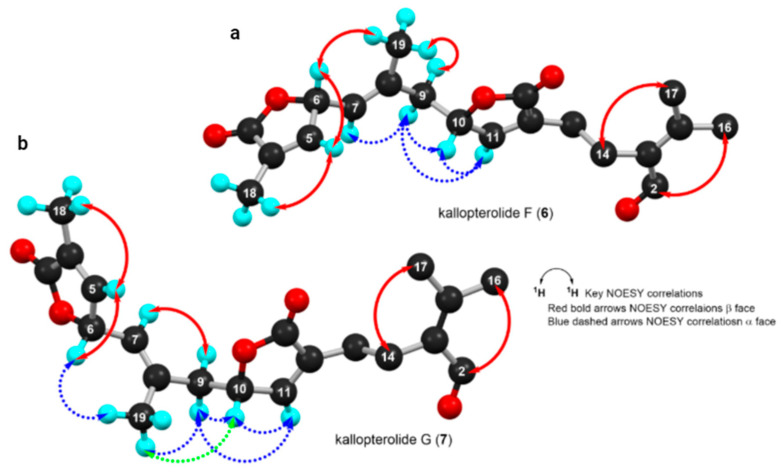
Computer-generated perspective views for the lowest energy conformers for (**a**) kallopterolide F (**6**) and (**b**) kallopterolide G (**7**). Some hydrogen atoms have been omitted for clarity.

The “open-chain” nature of structures **6** and **7** severely hampered our ability to assign their relative stereochemistry. In addition, DU8ML failed to differentiate compounds **6** and **7**’s relative configurations confidently, thus our assignments should be taken as tentative. As a convenient starting point, we adopted for **6** and **7** the same 10S configuration as that usually found in cembranolides from other sea plume species belonging to the *Pseudopterogorgia* genus. Moreover, careful analysis of the NOESY spectra of compounds **6** and **7**, as well as side–side comparisons of their coupling constant data together with molecular modeling studies, established that both molecules share a similar 3D conformation (Figure 11). In particular, the pivotal H-10 proton, in **7**, showed a NOESY cross-peak with the H_3_-19 protons. The latter methyl protons, in turn, showed a NOESY correlation with H-6. If we assume that H-10 lies in the β face, H-6, too, must be assigned to the same face. The anti-periplanar relationship between H-6 and H-7 was deduced from the coupling constant value of 8.6 Hz.

Interestingly, the NOESY spectrum of kallopterolide G (**7**) displayed similar NOESY correlations except for the key correlation between H-10 and H_3_-19 that was not present between these protons in the NOESY spectrum of kallopterolide F (**6**). These dissimilarities connote that *bis*-butenolides **6** and **7** are epimers at C-6 (Figure 11). Molecular modeling experiments corroborated these observations and established that the most likely relative stereochemistry for kallopterolides F (**6**) and G (**7**) is 6R*,10S* and 6S*,10S*, respectively. In both **6** and **7**, the E geometry was assigned to the Δ^7^-trisubstituted olefin based on the shielded methyl carbon resonance at δ_C_ 18.1 in kallopterollide F (**6**) and δ_C_ 17.2 in kallopterolide G (**7**), respectively. The respective absence of a NOESY cross-peak between H-7 and H_3_-19 in the spectrum of each compound confirmed the proposed geometry.

Kallopterolide H (**8**) was isolated as a yellowish oil, ${\left[\alpha \right]}_{D}^{20}$ +56.9 (c 1.0, acetone). The HR-ESI-MS exhibited a pseudomolecular ion [M + H]^+^ at *m*/*z* 361.1654 (calcd 361.1651, C_20_H_25_O_6_), appropriate for a molecular formula of C_20_H_24_O_6_. The latter required nine degrees of unsaturation, which was supported by ^13^C NMR and DEPT NMR data (Table 6). A difference of sixteen mass units in the molecular formula of kallopterolide G (**7**) in relation to kallopterolide H (**8**) revealed the presence of an extra oxygen atom in compound **8**. The ^1^H and ^13^C NMR data for kallopterolide H (**8**) were very similar to those for kallopterolide G (**7**), but they did not show the characteristic aldehyde resonances at δ_H_ 10.10 (s, 1H, H-2) or δ_C_ 190.7 (C, C-2). On the other hand, the appearance of a shielded carbonyl resonance at δ_C_ 172.3 (C, C-2), in addition to a broad IR absorption band at 3446 cm^−^^1^, corroborated the presence of a carboxylic acid functionality. This information suggested that kallopterolide H (**8**) is the C-2 carboxylic acid derivative of kallopterolide G (**7**). Because similar NOEs and 1D NMR data (^1^H and ^13^C NMR) were observed for each compound, it was concluded that most likely they possess identical stereochemistry. Unfortunately, the DU8ML method failed to assign the relative configuration for **8** confidently due to intra- or inter-molecular H-bonds in the conformational equilibrium.

Lastly, kallopterolide I (**9**) was isolated as a homogeneous yellowish oil. Unfortunately, ensuing decomposition of this compound after purification made it impossible for us to obtain IR, [α]_D_, UV, or HR-MS data. The planar structure of this compound was however elucidated using 2D NMR data collected prior to its decomposition. The overall 1D NMR data (Table 1) for compound **9** showed fourteen carbon resonances corresponding to two carbon-carbon and three carbon-oxygen double bonds indicating six sites of unsaturation. Careful analysis of the ^1^H, ^13^C DEPT-135, HMQC, ^1^H-^1^H COSY, and HMBC quickly revealed the presence of the –CH_2_–CH_2_–C(CHO)=C(CH_3_)_2_ and α,β-unsaturated-γ-lactone substructures. The appearance of two carbon resonances at δ_C_ 204.5 (C, C-8) and 30.5 (CH_3_, C-19), combined with the proton signal at δ_H_ 2.21 (s, 3H, H_3_-19) swiftly led us to identify a methyl ketone functionality. The presence of the latter functionality was confirmed by HMBC correlations of C-8 with H_2_-9 and H_3_-19. The lactone moiety was connected with the C-9 methylene from HMBC correlations of H_2_-9 to C-10 and C-11. This compound must likely stem from the C-7/C-8 oxidative cleavage of kallopterolides F (**6**), G (**7**) or H (**8**). The 10S stereochemistry depicted in **9**, although as likely as not to be correct, is implied and thus subject to confirmation.

### 2.2. Biogenesis

The co-occurrence of kallopterolides A-I with pseudopterane and cembrane diterpenes within the same organism suggests that compounds **1**–**9** could arise from successional oxidation–ring cleavage of the latter metabolites. As a matter of convenience, we could envision the title compounds as belonging to one of the following three subclasses, respectively, the Δ^2,3^ *seco*-pseudopteranes [kallopterolides A (**1**) and B (**2**)], the Δ^2,3^ *seco*-cembranes [kallopterolides C–H (**3**–**8**)], and the Δ^2,3^, Δ^7,8^ *bis*-*seco*-cembrane [kallopterolide I (**9**)]. Hereafter, a hypothetical biogenetic proposal has been put forward (see Scheme S1 in the Supplementary Materials) linking the kallopterolides to either the furanocembranolides or the furanopseudopteranolides, related diterpenes concomitant with this sea plume as well as other gorgonian species of the same order [18,19,20,21,22,23]. In so far as the absolute structures of some of these plausible precursors have been established, Scheme S1 in the Supporting Materials justifies our bias in choosing the stereochemistry depicted in structures **1**–**9**.

### 2.3. Biological Activity

Marine natural products are important sources of biologically active agents, and a plethora of bioactive compounds have been extracted from marine organisms like tunicates, sponges, soft corals, and molluscs [24]. These biologically active compounds have been reported to modulate various biological activities and have anti-inflammatory, antifungal, and anticancer effects [25]. Despite our best efforts to detect meaningful bioactivity, the kallopterolides demonstrated marginal or no activity at all as potential anti-inflammatory, antiprotozoal, or antituberculosis agents. Thus, kallopterolides A–E (**1**–**5**) demonstrated minimal effects on the release of TXB_2,_ O_2_^−^, or lactate dehydrogenase (LDH) (a marker for cell cytotoxicity) from *E. coli* lipopolysaccharide-activated rat neonatal microglia in vitro [26]. On the other hand, kallopterolides D (**4**) and E (**5**) showed no activity against chloroquine-resistant *Plasmodium falciparum* W2 (IC_50_ values ≥ 50 μg/mL) [27]. Kallopterolide E (**5**) was tested for in vitro antituberculosis activity against *Mycobacterium tuberculosis* H_37_Rv, but it was found to exhibit only marginal mycobacterial growth inhibition (23%) at a concentration of 6.25 μg/mL. Likewise, kallopterolide A (**1**) showed 0% growth inhibition at the same concentration [28]. It should be remarked here that kallopterolides F–I (**6**–**9**) could not be tested in the anti-inflammatory, antiplasmodial, or antituberculosis assays due to either scarcity or untimely decomposition of the natural products.

## 3. Materials and Methods

### 3.1. General Experimental Procedures

1D- and 2D-NMR spectra were recorded with a Bruker DPX-300 or DRX-500 FT-NMR spectrometers (Bruker Corporation, Billerica, MA, USA). Infrared and UV spectra were obtained with a Nicolet Magna FT-IR 750 spectrometer (Nicolet Instrument Corporation, Madison, WI, USA) and a Shimadzu UV-2401 PC UV-Visible (Shimadzu Corporation, Columbia, MD, USA) recording spectrophotometers, respectively. Optical rotations were obtained with an Autopol IV (Rudolph Research Analytical, Hackettstown, NJ, USA) automatic polarimeter. HR-EI-MS, HR-FAB-MS, HR-ESI-MS, and LR-EI-MS analyses were generated at the Mass Spectrometry Laboratory of the University of Illinois at Urbana–Champaign. Routine molecular modeling studies were performed with MacSpartan Pro and/or Spartan 04’ Programs (Wavefunction, Irvine, CA, USA). Column chromatography was performed using silica gel (35–75 mesh) (Analtech, Newark, DE, USA) and TLC analyses were carried out using glass precoated silica gel plates. HPLC was performed using either an Ultrasphere polar-bonded Cyano semi-preparative (Avantor, Radnor, PA, USA) column (5 μ, 10 mm *×* 25 cm) or an Ultrasphere normal-phase Si gel semi-preparative column (5 μ, 10 mm *×* 25 cm). All HPLC separations were carried out using a flow rate = 2 mL/min with isocratic elution of the mobile phase with the UV detector set at λ = 220 nm. All solvents used were either spectral grade or were distilled from glass before use. The percentage yield of each compound is based on the weight of the dry gorgonian specimen.

### 3.2. Collection and Extraction of Antillogorgia kallos

Fresh specimens of the sea plume *Antillogorgia kallos* (Bielschowsky, 1918) [8] were collected by hand using SCUBA at depths of 83–91 ft in Old Providence Island, Colombia, on 15–16 March 2002. A voucher specimen is stored in the Chemistry Department of the University of Puerto Rico–Río Piedras Campus. The organism was partially air-dried, frozen, and lyophilized prior to extraction. The dry specimens (1.07 kg) were blended using a mixture of CH_2_Cl_2_/MeOH (1:1) (20 × 1 L). After filtration, the crude extract was concentrated and stored under vacuum to yield a greenish gum (166 g). The crude extract was suspended in water (2 L) and extracted with n-hexane (3 × 2 L), CHCl_3_ (3 × 2 L), and EtOAc (2 × 2 L). Each extract was concentrated under reduced pressure to yield 71.9 g of the n-hexane extract, 39.3 g of the CHCl_3_ extract, and 1.47 g of the EtOAc extract.

### 3.3. Isolation of Natural Products

The structure for each known natural product co-isolated during this investigation is given in Figure 1. The crude CHCl_3_ extract (39.3 g), a brown amorphous solid, was chromatographed over silica gel (673 g) using a step gradient of EtOAc/n-hexane as eluent and separated into 32 fractions (A-FF) based on TLC and ^1^H NMR analyses. Purification of fraction L (1.34 g) by silica gel CC (70 g) using 1% of acetone in CHCl_3_ gave 12 fractions (L1–L12). Subfraction L8 (769.8 mg) was separated into 14 subfractions (L8.1–L8.14) by silica gel CC using 98:2 CHCl_3_/acetone. Further HPLC purification of subfraction L8.14 (110 mg) by HPLC using a polar bonded Ultrasphere-CN column in 85:15 n-hexane/IPA led to the isolation of known biskallolide A (16.2 mg, 0.0015%) along with two new seco-pseudopteranes, namely, kallopterolide A (**1**) (23.9 mg, 0.0022%) and kallopterolide B (**2**) (7.8 mg, 0.00073%). Direct purification of subfraction L9 by HPLC following the previous method also yielded **1** (2.0 mg, 0.00010%) and **2** (4.0 mg, 0.00037%). Separation of fraction N (331 mg) by normal phase HPLC using an Ultrasphere-Si gel column with 85:15 n-hexane/IPA as eluant led to the isolation of four additional compounds, namely, bipinnatolide J (10.1 mg, 0.0009%), bipinnapterolide A (88.9 mg, 0.0083%), kallopterolide A (**1**) (25.5 mg, 0.0024%), and kallopterolide B (**2**) (13.3 mg, 0.0012%). Separation of fraction O (1.59 g) by silica gel CC (75 g) in 1% acetone in CHCl_3_ gave 13 fractions (O1–O13). Subfraction O6 (335.1 mg) was purified by HPLC using an Ultrasphere-CN column with 90:10 n-hexane/IPA to yield additional quantities of the following compounds: bipinnapterolide A (17.1 mg, 0.0016%), caucanolide E (1.0 mg, 0.00009%), kallopterolide A (**1**) (31.2 mg, 0.0029%), kallopterolide B (**2**) (16.2 mg, 0.0015%), caucanolide F (2.7 mg, 0.00025%), caucanolide C (1.0 mg, 0.00009.3%), and caucanolide D (12.1 mg, 0.0011%). Fraction Q (351 mg) was chromatographed over 20 g of silica gel with 75:25 n-hexane/acetone to give 6 subfractions (Q1–Q6), the second of which (Q2) was later identified as kallopterolide I (**9**) (26.4 mg, 0.0025%). Fraction S (1.85 g) was chromatographed successively over 70 g of silica gel with 80:20 n-hexane/EtOAc and then 50 g of silica gel with 85:15 n-hexane/acetone to give 6 subfractions (S1–S6). The last fraction (410 mg) was purified further by HPLC using an Ultrasphere-CN column with 70:30 n-hexane/IPA to yield pure kallopterolide H (**8**) (18.0 mg, 0.0017%). Fraction T (510 mg) was separated in 10 subfractions (T1–T10) by silica gel CC (35 g) with 2.5% acetone in CHCl_3_. Purification of subfraction T3 (39.6 mg) by HPLC using an Ultrasphere-CN column with 86:14 n-hexane/IPA yielded kallopterolides F (**6**) (10.0 mg, 0.00093%) and G (**7**) (1.0 mg, 0.000093%). Fraction U (849 mg) was divided into 12 fractions (U1–U12) by silica gel CC with 95:5 CHCl_3_/EtOAc as mobile phase. Further purification of subfraction U8 (49.5 mg) by HPLC using an Ultrasphere-CN column with 85:15 n-hexane/IPA afforded kallopterolide C (**3**) (17.8 mg, 0.0017%). Fraction V (1.23 g) was separated over silica gel (65 g) with 97:3 CHCl_3_/EtOAc to afford 18 subfractions (V1–V18). Subfraction V6 (78.1 mg) was purified by HPLC with an Ultrasphere-CN column with 85:15 n-hexane/IPA yielding kallopterolide D (**4**) (5.1 mg, 0.00048%) and kallopterolide E (**5**) (10.1 mg, 0.00094%). Column chromatography of fraction W (1.94 mg) over silica gel (70 g) with 97:3 CHCl_3_/acetone generated 14 subfractions (W1–W14). Subsequent purification of subfraction W9 (206 mg) by HPLC using an Ultrasphere-CN column with 85:15 n-hexane/IPA afforded additional quantities of **4** (15.6 mg, 0.0015%) and **5** (29.3 mg, 0.0027%).

The crude n-hexane extract (71.9 g) was dissolved in a small volume of toluene, filtered over Celite^®^ (MilliporeSigma, Burlington, MA, USA), and loaded onto a large Bio-Beads SX-3 (Bio-Rad Laboratories, Hercules, CA, USA) column using toluene as the mobile phase. A total of seven fractions (H1–H7) were obtained based on TLC and ^1^H-NMR analyses. Fraction H5 (7.0 g) was separated into 19 fractions (H5.1–H5.19) over silica gel CC (340 g) with 85:15 n-hexane/acetone. Subfraction H5.15 (126 mg) was purified by HPLC using an Ultrasphere-Si Gel column with 91.5:8.5 n-hexane/IPA to yield kallolide I (25.0 mg, 0.0023%), pinnatin B (26 mg, 0.0024%), kallopterolide B (**2**) (4.3 mg, 0.00040%), and kallopterolide A (**1**) (9.4 mg, 0.00088%). Subfraction H5.18 (1.6 g) was divided into several subfractions by silica gel CC (70 g) with 97:3 CH_2_Cl_2_/acetone. Further purification of subfraction H5.18.6 (63.5 mg) by HPLC using an Ultrasphere-CN column with 85:15 n-hexane/IPA yielded additional quantities of **3** (2.0 mg, 0.00019%), **4** (2.0 mg, 0.00019%), and **5** (4.0 mg, 0.00037%). Fraction H6 (2.2 g) was separated into 22 fractions (H6.1–H6.22) by silica gel CC (140 g) with 20% EtOAc–n-hexane. Subfraction H6.17 (113 mg) was purified by HPLC using an Ultrasphere-Si Gel column with 88:12 n-hexane/IPA to afford kallolide I (1 mg, 0.000093%), bipinnapterolide A (11.8 mg, 0.0011%), kallopterolide A (**1**) (8.7 mg, 0.00081%), and kallopterolide B (**2**) (3.9 mg, 0.00036%).

kallopterolide A (**1**): yellowish oil; ${\left[\alpha \right]}_{D}^{20}$ +20.0 (c 1.0, MeOH); IR (neat) 3093, 2976, 2931, 2858, 2772, 1775, 1757, 1660, 1626, 1445, 1377, 1254, 1058, 997, 957, 883, 759 cm^−^^1^; UV (MeOH) λ_max_ 209 (ε 26,500), 245 (ε 15,800) nm; ^1^H NMR (500 MHz, CDCl_3_) see Table 1; ^13^C NMR (125 MHz, CDCl_3_) see Table 1; LR-EI-MS *m*/*z* [M]^+^ 344 (8), 343 (30), 342 (20), 331 (12), 314 (11), 296 (26), 245 (22), 244 (16), 192 (30), 177 (26), 164 (28), 153 (62), 151 (42), 137 (55), 135 (37), 123 (38), 123 (38), 111 (89), 95 (43), 91 (58), 85 (69), 83 (100), 69 (97), 67 (74); HR-FAB-MS *m*/*z* [M + H]^+^ 345.1702 (calcd for C_20_H_25_O_5_ 345.1702).

kallopterolide B (**2**): yellowish oil; ${\left[\alpha \right]}_{D}^{20}$ +5.0 (c 1.0, MeOH); IR (neat) 3080, 2970, 2929, 2860, 1750, 1716, 1670, 1654, 1457, 1375, 1052, 952 cm^−^^1^; UV (MeOH) λ_max_ 208 (ε 24,400), 242 (ε 15,500) nm; ^1^H NMR (500 MHz, CDCl_3_) see Table 1; ^13^C NMR (125 MHz, CDCl_3_) see Table 1; HR-FAB-MS *m*/*z* [M + H]^+^ 345.1696 (calcd for C_20_H_25_O_5_ 345.1702).

kallopterolide C (**3**): yellowish oil; ${\left[\alpha \right]}_{D}^{20}$ +12.5 (c 0.4, MeOH); IR (neat) 3438, 3012, 2975, 2932, 2918, 2879, 1775, 1662, 1623, 1441, 1376, 1237, 1194, 1062, 997, 867, 761 cm^−^^1^; UV (MeOH) λ_max_ 209 (ε 26,700), 257 (ε 17,100) nm; ^1^H NMR (500 MHz, CDCl_3_) see Table 4; ^13^C NMR (125 MHz, CDCl_3_) see Table 4; LR-EI-MS *m*/*z* [M]^+^ 360 (7), 342 (7), 330 (11), 153 (58), 151 (36), 137 (47), 135 (28), 123 (35), 111 (100), 91 (74), 111 (29), 91 (74), 69 (96).

kallopterolide D (**4**): yellowish oil; ${\left[\alpha \right]}_{D}^{20}$ −10.0 (c 0.9, CHCl_3_); IR (neat) 3461, 3090, 2970, 2926, 2871, 1775, 1752, 1663, 1625, 1447, 1373, 1056, 1194, 994, 958, 880, 810, 758 cm^−^^1^; UV (MeOH) λ_max_ 210 (ε 16,800), 263 (ε 19,800) nm; ^1^H NMR (500 MHz, CDCl_3_) see Table 4; ^13^C NMR (125 MHz, CDCl_3_) see Table 4; LR-EI-MS: *m*/*z* [M]^+^ 360 (2), 342 (25), 330 (12), 314 (16), 296 (17), 169 (17), 153 (58), 151 (36), 137 (46), 135 (28), 124 (56), 123 (35), 105 (56), 91 (87), 69 (100), 68 (92), 67 (97); HR-FAB-MS *m*/*z* [M + H]^+^ 361.1652 (calcd for C_20_H_25_O_6_ 361.1651).

kallopterolide E (**5**): yellowish oil; ${\left[\alpha \right]}_{D}^{20}$ −11.4 (c 0.7, CHCl_3_); IR (neat) 3453, 3093, 2976, 2931, 2871, 1775, 1757, 1660, 1626, 1445, 1377, 1254, 1058, 997, 957, 883, 759 cm^−^^1^; UV (MeOH) λ_max_ 211 (ε 17,700), 259 (ε 21,400) nm; ^1^H NMR (500 MHz, CDCl_3_) see Table 4; ^13^C NMR (125 MHz, CDCl_3_) see Table 4; LR-EI-MS: *m*/*z* [M]^+^ 360 (8), 343 (30), 342 (20), 331 (12), 314 (11), 296 (26), 245 (22), 244 (16), 192 (30), 177 (26), 164 (28), 153 (62), 151 (42), 137 (55), 135 (37), 123 (38), 123 (38), 111 (89), 95 (43), 91 (58), 85 (69), 83 (100), 69 (97), 67 (74); HR-EI-MS *m*/*z* [M]^+^ 360.1573 (calcd for C_20_H_24_O_6_ 360.1573).

kallopterolide F (**6**): yellowish oil; ${\left[\alpha \right]}_{D}^{20}$ −30.0 (c 1.0, MeOH); IR (neat) 3081, 2932, 2864, 1750, 1658, 1621, 1437, 1375, 1254, 1203, 1092, 947 cm^−^^1^; UV (MeOH) λ_max_ 204 (ε 26,600), 243 (ε 18,600) nm; ^1^H NMR (500 MHz, CDCl_3_) see Table 6; ^13^C NMR (125 MHz, CDCl_3_) see Table 6; HR-FAB-MS *m*/*z* [M + Na]^+^ 367.1528 (calcd for C_20_H_24_O_5_Na 367.1521).

kallopterolide G (**7**): yellowish oil; ${\left[\alpha \right]}_{D}^{20}$ −46.5 (c 1.0, MeOH); IR (neat) 3080, 2954, 2817, 2849, 1750, 1732, 1716, 1683, 1653, 1457, 1375, 1262, 1204, 1098 cm^−^^1^; UV (MeOH) λ_max_ 205 (ε 28,200), 256 (ε 19,400) nm; ^1^H NMR (500 MHz, CDCl_3_) see Table 6; ^13^C NMR (125 MHz, CDCl_3_) see Table 6; HR-ESI-MS: *m*/*z* [M + H]^+^ 345.1704 (calcd for C_20_H_25_O_5_ 345.1702).

kallopterolide H (**8**): yellowish oil; ${\left[\alpha \right]}_{D}^{20}$ +56.9 (c 1.0, acetone); IR (neat) 3446, 3089, 2982, 2930, 1749, 1650, 1437, 1375, 1258, 1204, 1095 cm^−^^1^; UV (MeOH) λ_max_ 207 (ε 24,100), 258 (ε 18,900) nm; ^1^H NMR (500 MHz, CDCl_3_) see Table 6; ^13^C NMR (125 MHz, CDCl_3_) see Table 6; HR-ESI-MS *m*/*z* [M + H]^+^ 361.1654 (calcd for C_20_H_25_O_6_ 361.1651).

kallopterolide I (**9**): yellowish oil; ^1^H NMR (500 MHz, CDCl_3_) see Table 1; ^13^C NMR (125 MHz, CDCl_3_) see Table 1. Shortly after purification (by a combination of silica gel CC and HPLC) this compound slowly begun to decompose. However, several additional NMR experiments (DEPT-135, HMQC, HMBC, and COSY) were acquired successfully prior to its decomposition.

### 3.4. Computational Method

For the calculations and details regarding the computational method used, see Scheme S2 in the Supplementary Materials.

### 3.5. Anti-Inflammation Bioassay

The anti-inflammation assays were performed at the Department of Pharmacology, Chicago College of Osteopathic Medicine, Midwestern University, 555 31st Street, Downers Grove, Illinois by members of Professor Alejandro M. S. Mayer’s Research Group. Rat neonatal microglia (2 × 10^5^ cells) were seeded into each well of 24-well flat-bottom culture clusters and stimulated with bacterial lipopolysaccharide (LPS) (0.3 ng/mL) in Dulbecco’s modified Eagle medium + 10% fetal bovine serum + penicillin + streptomycin for 17 h in a humidified 5% CO_2_ incubator at 35.9 °C. Media were then removed, and microglia were washed with warm (37 °C) Hanks’ balanced salt solution (HBSS) and then incubated with the title compounds (0.01–10 µM) or vehicle (DMSO) for 15 min prior to stimulation with phorbol 12-myristate 13-acetate (PMA) (1 µM). All experimental treatments were run in triplicate and in a final volume of 1 mL. Seventy minutes after PMA stimulation, HBSS was aspirated from each well and O_2_^–^, TXB_2_, and LDH release were determined as described elsewhere [26].

### 3.6. Antiplasmodial Bioassay

The antiplasmodial activity of some of the isolated compounds was evaluated against a chloroquine-resistant (Indochina W2) strain of *Plasmodium falciparum* using a novel DNA-based microfluorimetric method. This method was developed, and all the antiplasmodial bioassays were performed, at the Instituto de Investigaciones Científicas Avanzadas y Servicios de Alta Tecnología, Ciudad del Saber, Clayton, Panama. Detailed description of the experimental method used for this assay is given elsewhere [27]. In this bioassay compounds displaying an IC_50_ value > 10 μg/mL are considered inactive and those with an IC_50_ value ≤ 10 μg/mL are considered active.

### 3.7. Antimycobacterial Bioassay

The antitubercular activity of some of the isolated compounds was evaluated against the laboratory strain *Mycobacterium tuberculosis* H_37_Rv. A detailed description of the experimental method used for this antitubercular assay has been previously described [28]. All the antimycobacterial assays were performed in the Institute for Tuberculosis Research, University of Illinois at Chicago, College of Pharmacy, 833 S. Wood Street (M/C 964) Chicago, IL by members of Professor Scott G. Franzblau’s Research Group. In this bioassay compounds displaying inhibitory growth percentage ≥ of 90% at 6.25 μg/mL are considered active.

## 4. Conclusions

The molecular structures for two new Δ^2,3^ *seco*-pseudopteranes, kallopterolides A–B (**1**–**2**), six new Δ^2,3^ *seco*-cembranes, kallopterolides C-H (**3**–**8**), and arguably the first Δ^2,3^, Δ^7,8^ *bis-seco*-cembrane named kallopterolide I (**9**), were elucidated using spectroscopic techniques, such as 1D-, 2D-NMR, IR, UV, and HR-MS, and supported by a modern computational method. The co-occurrence of kallopterolide, cembrane, and pseudopterane diterpenoids within the same specimen of *Antillogorgia kallos* provides circumstantial evidence supporting our contention that the kallopterolides could arise following oxidative cleavage of the latter two families of cyclic diterpenes. Kallopterolides A-H (**1**–**8**) possess chemical structures reminiscent of other *seco*-cembranes and *seco*-pseudopteranes previously isolated from *Pseudopterogorgia bipinnata* [7] (see Tables S2–S4 as Supplementary Materials). Although an inarguable similarity exists between the structures of **1** and **2** with that of caucanolide A, the structures of kallopterolides D (**4**) and E (**5**) are in closer proximity to those of caucanolides E and F, respectively. Briefly, **4** and **5** are the free alcohols of methyl ethers caucanolides E and F. Additionally, kallopterolide F (**6**) differs structurally with caucanolide D only in the substitution of an -H for an –OMe. The relative stereochemistry for all these compounds must be considered tentative as their “open-chain” nature made it almost impossible to interpret the results of the 2D-NMR NOESY spectra rigorously.

At this moment, no sort of meaningful biological activity has been ascribed to the new compounds **1**–**9**. It is interesting to remark, however, that while caucanolides A and D demonstrated significant in vitro antiplasmodial activity (IC_50_ ~15–17 µg/mL), neither caucanolides E and F nor kallopterolides D (**4**) and E (**5**) showed significant activity [7]. Sadly, since kallopterolide F (**6**) could not be assayed for antiplasmodial activity during this investigation it would be impossible at this time to draw any data regarding plausible structure–activity relationships between the latter compound and caucanolide D. To conclude, we should also call attention to the fact that many natural products bearing the same γ-alkylidenebutenolide functionality as kallopterolides C–E (**3**–**5**) and caucanolides E and F have been found to exhibit compelling biological activities, such as cytotoxic, α-glucosidase inhibitory, nitric oxide production inhibitory, melanogenesis inhibitory, fungicidal, and antibacterial activities [17].

## Figures and Tables

**Table 1:** ^1^H NMR (500 MHz) and ^13^C NMR (125 MHz) spectral data for kallopterolide A (**1**), kallopterolide B (**2**), and kallopterolide I (**9**) ^a^.

	Kallopterolide A (1)		Kallopterolide B (2)		Kallopterolide I (9)	
Atom	δ_H_, Mult, Intgrt (*J* in Hz)	δ_C_ (Mult) ^b^	δ_H_, Mult, Intgrt (*J* in Hz)	δ_C_ (Mult) ^b^	δ_H_, Mult, Intgrt (*J* in Hz)	δ_C_ (Mult) ^b^
1		135.5 (C)		135.4 (C)		135.4 (C)
2	10.10, s, 1H	190.6 (CH)	10.10, s, 1H	190.7 (CH)	10.10, s, 1H	190.6 (CH)
3		173.8 (C)		173.5 (C)		
4		130.9 (C)		130.9 (C)		
5	7.06, dd, 1H (1.2, 1.0)	146.8 (CH)	7.10, dd, 1H (1.6, 1.5)	147.0 (CH)		
6	5.34, ddd, 1H (3.9, 2.0, 1.9)	79.8 (CH)	5.05, dd, 1H (1.7, 1.6) ^c^	80.0 (CH)		
7	2.21, dd, 1H (10.0, 4.3)	53.5 (CH)	2.60, dd, 1H (7.0, 7.0)	52.8 (CH)		
8	5.16, ddd, 1H (10.3, 1.4, 1.0)	80.1 (CH)	5.07, m, 1H ^c^	79.9 (CH)		204.5 (C)
9α	7.09, dd, 1H (1.6, 1.2)	147.4 (CH)	7.13, d, 1H (1.4)	146.8 (CH)	2.98, dd, 1H (17.6, 6.8)	46.9 (CH_2_)
9β					2.63, dd, 1H (17.6, 7.0)	
10		134.6 (C)		134.6 (C)	5.28, ddd, 1H (7.0, 6.8, 1.6)	76.8 (CH)
11	2.29, m, 2H	24.5 (CH_2_)	2.28, m, 2H	24.6 (CH_2_)	7.17, dd, 1H (1.5, 1.0)	147.8 (CH)
12	2.52, m, 2H	23.4 (CH_2_)	2.52, m, 2H	23.4 (CH_2_)		133.9 (C)
13		156.8 (C)		156.9 (C)	2.25, m, 2H	24.4 (CH_2_)
14	2.03, s, 3H	23.4 (CH_3_)	2.03, s, 3H	23.4 (CH_3_)	2.50, m, 2H	23.3 (CH_2_)
15	2.21, s, 3H	19.4 (CH_3_)	2.21, s, 3H	19.4 (CH_3_)		156.9 (C)
16	1.92, d (1.7)	10.8 (CH_3_)	1.94, d (1.6)	10.7 (CH_3_)	2.19, s, 3H	19.3 (CH_3_)
17		137.8 (C)		139.2 (C)	2.01, s, 3H	23.4 (CH_3_)
18α	4.89, br s, 1H	117.1 (CH_2_)	4.83, br s, 1H	116.8 (CH_2_)		
18β	5.07, br s, 1H		5.08, br s, 1H			
19	1.72, br s, 3H	23.7 (CH_3_)	1.81, br s, 3H	23.6 (CH_3_)	2.21, s, 3H	30.5 (CH_3_)
20		172.9 (C)		173.0 (C)		173.2 (C)

^a^ NMR spectra were recorded in CDCl_3_ at 25 °C; ^1^H and ^13^C NMR chemical shift values are in ppm and referenced to the residual CHCl_3_ (δ = 7.26) or CDCl_3_ (δ = 77.0) ppm signals. ^b 13^C NMR multiplicities were deduced from a DEPT NMR experiment. ^c^ Chemical shifts and ^1^H–^1^H coupling constant values are approximated due to second-order effects.

**Table 2:** Key differences observed between the ^1^H and ^13^C NMR spectra of kallopterolides A (**1**) and B (**2**) in CDCl_3_ at 25 °C.

	Kallopterolide A (1)		Kallopterolide B (2)	
Atom	δ_H_, Mult, Intgrt (*J* in Hz)	δ_C_ (Mult)	δ_H_, Mult, Intgrt (*J* in Hz)	δ_C_ (Mult)
5	7.06, dd, 1H (1.2, 1.0)	146.8 (CH)	7.10, dd, 1H (1.6, 1.5)	147.0 (CH)
6	5.34, ddd, 1H (3.9, 2.0, 1.9)	79.8 (CH)	5.05, dd, 1H (1.7, 1.6)	80.0 (CH)
7	2.21, dd, 1H (10.0, 4.3)	53.5 (CH)	2.60, dd, 1H (7.0, 7.0)	52.8 (CH)
8	5.16, ddd, 1H (10.3, 1.4, 1.0)	80.1 (CH)	5.07, m, 1H	79.9 (CH)
9	7.09, dd, 1H (1.6, 1.2)	147.4 (CH)	7.13, d, 1H (1.4)	146.8 (CH)

**Table 3:** Comparison of the experimental NMR spectroscopic data of kallopterolides A (**1**) and B (**2**) with DU8ML-calculated NMR parameter of possible diastereomers ^a^.

	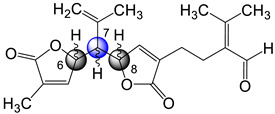			
	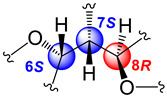	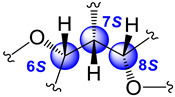	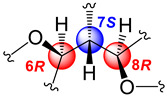	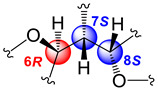
	6*S*,7*S*,8*R*	6*S*,7*S*,8*S*	6*R*,7*S*,8*R*	6*R*,7*S*,8*S*
**A**	{**1.73**/0.19/1.49}	{0.50/0.20/1.39}	{0.41/0.19/1.28}	{**1.51**/0.18/1.65}
**B**	{0.42/0.20/1.32}	{**2.05**/0.25/1.28}	{**1.73**/0.25/1.24}	{0.59/0.20/1.49}

^a^ Root Mean Square Deviations (RMSDs) for the ^1^H-^1^H spin-spin coupling constants, ^1^H chemical shifts, and ^13^C chemical shifts, presented as triads in curly brackets. Blue circles (and alphanumeric characters) denote the *S* configuration. Red circles (and alphanumeric characters) denote the *R* configuration. Red bold font highlights bad (i.e., too high) RMSD numbers for *J*’s indicating a mismatch.

**Table 4:** ^1^H NMR (500 MHz) and ^13^C NMR (125 MHz) spectral data for kallopterolide C (**3**), kallopterolide D (**4**), and kallopterolide E (**5**) ^a^.

	Kallopterolide C (3)		Kallopterolide D (4)		Kallopterolide E (5)	
Atom	δ_H_, Mult, Intgrt (*J* in Hz)	δ_C_ (Mult) ^b^	δ_H_, Mult, Intgrt (*J* in Hz)	δ_C_ (Mult) ^b^	δ_H_, Mult, Intgrt (*J* in Hz)	δ_C_ (Mult) ^b^
1		135.4 (C)		135.5 (C)		135.5 (C)
2	10.10, s, 1H	190.7 (CH)	10.10, s, 1H	192.7 (CH)	10.10, s, 1H	190.6 (CH)
3		170.0 (C)		170.0 (C)		170.0 (C)
4		131.3 (C)		129.3 (C)		129.5 (C)
5	7.88, q, 1H (1.5)	136.5 (CH)	7.05, dd, 1H (1.5, 1.0)	138.6 (CH)	7.02, dd, 1H (1.5, 1.0)	138.5 (CH)
6		149.5 (C)		146.9 (C)		146.0 (C)
7	5.62, s, 1H	117.4 (CH)	5.43, s, 1H	118.3 (CH)	5.37, s, 1H	119.2 (CH)
8		72.7 (C)		71.8 (C)		72.0 (C)
9α	2.09, dd, 1H (15.0, 2.8)	46.3 (CH_2_)	2.11, dd, 1H (14.6, 4.3)	45.8 (CH_2_)	2.40, dd, 1H (14.7, 3.5)	45.1 (CH_2_)
9β	1.77, dd, 1H (15.0, 10.3)		1.92, dd, 1H (14.6, 8.3)		1.85, dd, 1H (14.6, 5.3)	
10	5.08, ddd, 1H (10.3, 2.8, 1.5)	78.8 (CH)	5.15, ddd, 1H (8.2, 4.2, 2.6)	80.9 (CH)	5.07, ddd, 1H (5.1, 3.4, 1.7)	78.8 (CH)
11	7.07, d, 1H (1.5)	148.6 (CH)	7.14, dd, 1H (2.8, 1.4)	149.6 (CH)	7.12, dd, 1H (1.6, 1.2)	149.1 (CH)
12		133.6 (C)		133.1 (C)		133.2 (C)
13	2.27, m, 2H	24.2 (CH_2_)	2.26, m, 2H	24.4 (CH_2_)	2.26, m, 2H	24.4 (CH_2_)
14	2.50, m, 2H	23.3 (CH_2_)	2.50, m, 2H	23.4 (CH_2_)	2.49, m, 2H	23.3 (CH_2_)
15		157.1 (C)		156.7 (C)		156.8 (C)
16	2.21, s, 3H	19.4 (CH_3_)	2.20, s, 3H	19.4 (CH_3_)	2.20, s, 3H	19.4 (CH_3_)
17	2.03, s, 3H	23.4 (CH_3_)	2.01, s, 3H	23.4 (CH_3_)	2.01, s, 3H	23.4 (CH_3_)
18	2.02, s, 3H	10.8 (CH_3_)	2.01, s, 3H	10.5 (CH_3_)	2.00, s, 3H	10.5 (CH_3_)
19	1.48, s, 3H	30.8 (CH_3_)	1.55, s, 3H	30.1 (CH_3_)	1.53, s, 3H	28.9 (CH_3_)
20		172.7 (C)		173.5 (C)		173.2 (C)
8 OH	2.99, br s, 1H					

^a^ NMR spectra were recorded in CDCl_3_ at 25 °C; ^1^H and ^13^C NMR chemical shift values are in ppm and referenced to the residual CHCl_3_ (δ = 7.26) or CDCl_3_ (δ = 77.0) ppm signals. ^b 13^C NMR multiplicities were deduced from a DEPT NMR experiment.

**Table 5:** Comparison of the experimental ^13^C-NMR spectroscopic data of kallopterolides C (**3**), D (**4**), and E (**5**) with calculated ^13^C chemical shift RMSDs of possible diastereomers ^a^.

	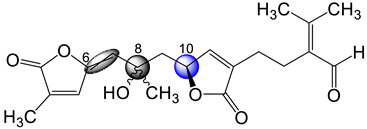			
	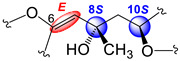	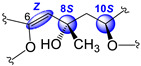	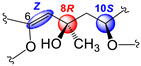	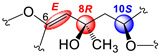
	SS-E	SS-Z	SR-Z	SR-E
**C**	1.23	1.83	2.02	1.37
**D**	1.71	1.34	1.66	1.45
**E**	1.75	1.12	1.66	1.50

^a^ Blue circles (and alphanumeric characters) denote the *S* configuration. Red circles (and alphanumeric characters) denote the *R* configuration. Values highlighted represent unambiguous matches.

**Table 6:** ^1^H NMR (500 MHz) and ^13^C NMR (125 MHz) spectral data for kallopterolide F (**6**), kallopterolide G (**7**), and kallopterolide H (**8**) ^a^.

	Kallopterolide F (6)		Kallopterolide G (7)		Kallopterolide H (8)	
Atom	δ_H_, Mult, Intgrt (*J* in Hz)	δ_C_ (Mult) ^b^	δ_H_, Mult, Intgrt (*J* in Hz)	δ_C_ (Mult) ^b^	δ_H_, Mult, Intgrt (*J* in Hz)	δ_C_ (Mult) ^b^
1		135.5 (C)		135.5 (C)		124.8 (C)
2	10.10, s, 1H	190.7 (CH)	10.10, s, 1H	190.7 (CH)		172.3 (C)
3		174.3 (C)		174.4 (C)		174.4 (C)
4		130.1 (C)		130.1 (C)		130.1 (C)
5	6.93, dd, 1H (1.6, 1.5)	147.5 (CH)	6.95, dd, 1H (1.5, 1.0)	147.6 (CH)	6.94, dd, 1H (1.6, 1.5)	147.6 (CH)
6	5.59, ddq, 1H (8.6, 1.7, 1.6)	77.5 (CH)	5.59, ddq, 1H (8.6, 1.8, 1.7)	77.5 (C)	5.59, ddd, 1H (8.8, 3.6, 1.8)	77.5 (C)
7	5.08, dd, 1H (8.6, 1.0)	122.3 (CH)	5.04, dd, 1H (8.6, 1.7)	122.9 (CH)	5.03, dd, 1H (8.9, 1.5)	122.9 (CH)
8		138.1 (C)		138.1 (C)		138.0 (C)
9α	2.43, dd, 1H (14.7, 5.4)	42.7 (CH_2_)	2.36, m, 2H	43.4 (CH_2_)	2.37, m, 2H	43.4 (CH_2_)
9β	2.34, dd, 1H (14.7, 7.7)					
10	4.98, ddd, 1H (7.3, 5.5, 1.6)	79.6 (CH)	5.02, m, 1H	79.1 (CH)	5.00, ddd, 1H (9.0, 7.6, 1.4)	79.0 (CH)
11	7.05, d, 1H (1.3)	147.7 (CH)	7.05, d, 1H (1.3)	147.8 (CH)	7.05, d, 1H (1.2)	148.1 (CH)
12		134.2 (C)		134.2 (C)		134.0 (C)
13	2.27, m, 2H	24.5 (CH_2_)	2.27, m, 2H	24.5 (CH_2_)	2.42, m, 2H	24.8 (CH_2_)
14	2.50, m, 2H	23.4 (CH_2_)	2.51, m, 2H	23.4 (CH_2_)	2.58, m, 2H	27.8 (CH_2_)
15		157.1 (C)		157.0 (C)		149.0 (C)
16	2.21, s, 3H	19.4 (CH_3_)	2.21, s, 3H	19.4 (CH_3_)	1.90, s, 3H	23.1 (CH_3_)
17	2.03, s, 3H	23.5 (CH_3_)	2.03, s, 3H	23.5 (CH_3_)	2.10, s, 3H	23.5 (CH_3_)
18	1.93, d, 3H (1.6)	10.7 (CH_3_)	1.93, d, 3H (1.5)	10.7 (CH_3_)	1.93, d, 3H (1.5)	10.6 (CH_3_)
19	1.87, d, 3H (1.0)	18.1 (CH_3_)	1.91, d, 3H (1.7)	17.2 (CH_3_)	1.91, d, 3H (1.5)	17.1 (CH_3_)
20		173.3 (C)		173.4 (C)		173.4 (C)

^a^ NMR spectra were recorded in CDCl_3_ at 25 °C; ^1^H and ^13^C NMR chemical shift values are in ppm and referenced to the residual CHCl_3_ (δ = 7.26) or CDCl_3_ (δ = 77.0) ppm signals. ^b 13^C NMR multiplicities were deduced from a DEPT NMR experiment.

## Data Availability

The data presented in this study are available in this article and the Supplementary Materials.

## Supplementary Materials

The following supporting information can be downloaded at: https://www.mdpi.com/article/10.3390/molecules29112493/s1, Scheme S1: Proposed biogenetic pathways for the formation of the kallopterolides; Scheme S2: How to read the NMR tables in the SI DU8ML, machine learning protocols, computational details, and DU8ML data; Figures S1–S35: Spectra of kallopterolides; Tables S1–S4: ^1^HNMR (500 MHz) spectroscopic data for kallopterolide A (**1**) in CD_3_OD and side-by-side comparisons of NMR spectroscopic data between selected kallopterolides and caucanolides.
