# Correction: Marrero et al. Kallopterolides A–I, a New Subclass of *seco*-Diterpenes Isolated from the Southwestern Caribbean Sea Plume *Antillogorgia kallos*. *Molecules* 2024, *29*, 2493

**DOI:** 10.3390/molecules30142929

**Published:** 2025-07-11

**Authors:** Jeffrey Marrero, Luis A. Amador, Ivan M. Novitskiy, Andrei G. Kutateladze, Abimael D. Rodríguez

**Affiliations:** 1 Department of Chemistry, University of Puerto Rico, 17 Ave. Universidad STE 1701, San Juan, PR 00931, USA; jeffrey.marrero2@upr.edu (J.M.); luisalberto.amador@upr.edu (L.A.A.); 2 Molecular Sciences Research Center, University of Puerto Rico, 1390 Ponce de León Avenue, San Juan, PR 00926, USA; 3 Department of Chemistry and Biochemistry, University of Denver, Denver, CO 80208, USA; dr.novitskiy@gmail.com (I.M.N.); andrei.kutateladze@du.edu (A.G.K.)

**Text Correction**

A typographic error involving S* and R* was made in the original publication [1]. A correction has been made to Section 2. Results and Discussion section, specifically in Section 2.1. Chemical Structural Analysis, in the 6th and 7th paragraphs:

This conclusion was validated by the small axial-equatorial coupling constant between these protons (*J*_H6–H7_ = 4.1 Hz). Furthermore, the fixed conformation depicted in Figure 4a was supported by the strong NOESY correlations observed between H-5/Me-16, H-5/H-7, H-7/Me-19, Me-19/H-18β, and H-8/H-18α. Farther along the eastern quadrant, the peak assignment for Me-14 and Me-15 in the ^1^H-NMR spectrum of **1** was based on the NOESY cross-peak between H-2 and Me-15. While these methods allowed us to establish the assignments described, we should point out that the relative configuration drawn for **1**, namely, 6R*, 7S*, and 8R*, correlates well with the known absolute configuration of other diterpenes co-isolated during this investigation (namely, kallolide A, kallolide A acetate, kallolide C, bipinnapterolide A, and gersemolide). This observation aligns with our contention that, most likely, **1** originates following oxidation/cleavage at C-2/C-3 of a suitable pseudopterane-based precursor (see Figure 1 and Scheme S1 in the Supplementary Materials).

Kallopterolide B (**2**), an optically active yellowish oil, ${\left[\alpha \right]}_{D}^{20}$ +5.0 (c 1.0, MeOH), showed a pseudomolecular [M + 1]^+^ ion peak at *m*/*z* 345.1696 in the HR-FAB-MS corresponding to a molecular formula of C_20_H_25_O_5_ (calcd 345.1702). The IR and UV spectroscopic data for **2** were very similar to those recorded for kallopterolide A (**1**). Further examination revealed that their ^1^H and ^13^C NMR data in CDCl_3_ were also almost identical, indicating that both compounds possess identical functionality, namely, two α,β-unsaturated-γ-lactones, one α-substituted-β,β-dimethyl-α,β-unsaturated aldehyde, and one isopropenyl group. Therefore, we concluded that these compounds must be diastereomers. A detailed side-by-side comparison of the ^1^H NMR spectra of **1** and **2** (Table 1) revealed that the minor differences observed could be explained by inverting the configuration in **2** at C-6. The reversal at C-6 from R* in **1** to S* in **2** was rendered by subtle differences in the ^1^H NMR chemical shifts and coupling constants for H-6 [δ_H_ 5.34 (ddd, 1H, 3.9, 2.0, 1.9 Hz) in **1** vs. δ_H_ 5.05 (dd, 1H, 1.7, 1.6 Hz) in **2**] and H-7 [δ_H_ 2.21 (dd, 1H, 10.0, 4.3 Hz) in **1** vs. δ_H_ 2.60 (dd, 1H, 7.0, 7.0 Hz) in **2**] (Table 2).

**Error in Figure**

In the original publication, there was a mistake in Scheme S1, Figures 2 and S17 as published. The relative stereochemistry for compound **3** about the C8 stereocenter was drawn incorrectly. The corrected Scheme S1, Figure 2 and Figure S17 appear below.

The authors state that the scientific conclusions are unaffected. This correction was approved by the Academic Editor. The original publication has also been updated.

## Figures and Tables

**Figure 2:**
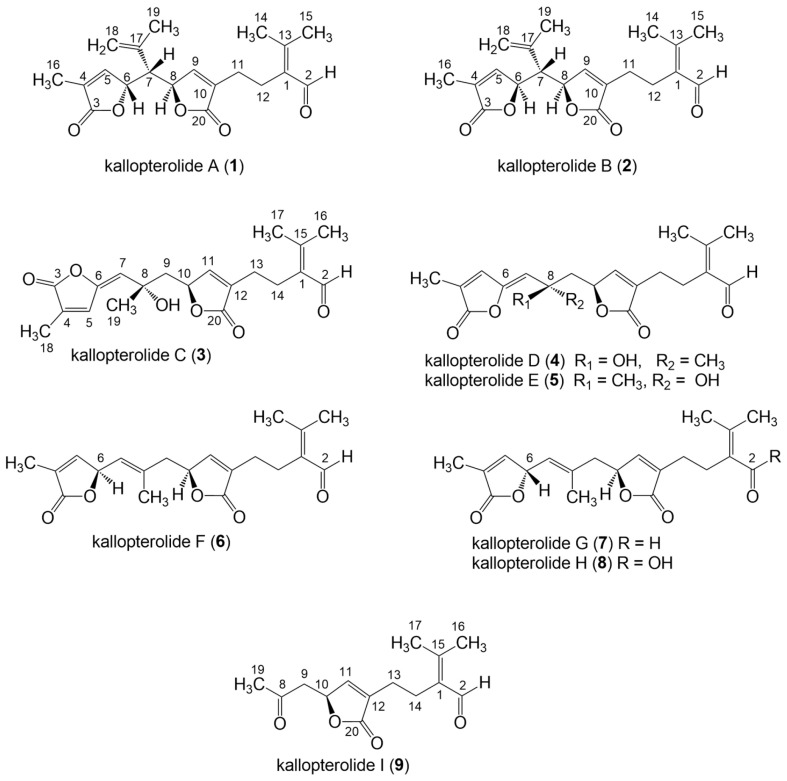
Chemical structures of kallopterolides A–I (**1**–**9**).

**Scheme S1:**
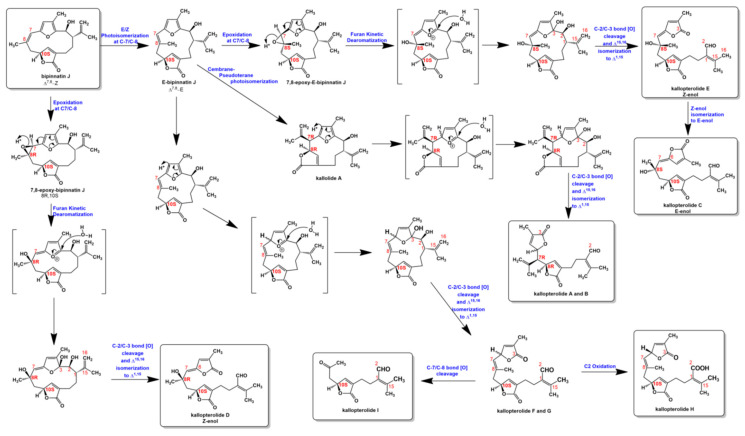
Proposed biogenetic pathways for the formation of the kallopterolides.

**Figure S17:**
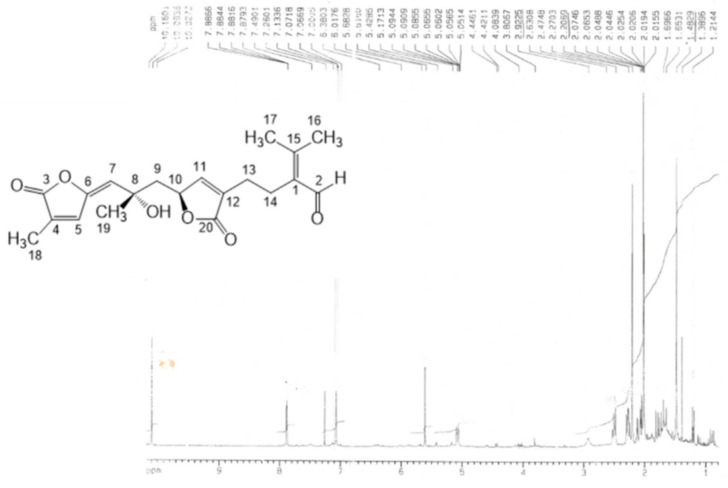
1H-NMR spectrum (CDCl3, 300 MHz) of Kallopterolide C (**3**).
