# Supplementary material for: Kallopterolides A–I, a New Subclass of seco-Diterpenes Isolated from the Southwestern Caribbean Sea Plume Antillogorgia kallos
Source: Molecules. 2024 May 24;29(11):2493. doi: 10.3390/molecules29112493 (PMC11173908; doi:10.3390/molecules29112493)
Supplement: Supplementary file 1 [file molecules-29-02493-s001.zip › molecules-3012914-supplementary.pdf]

Kallopterolides A–I, a new subclass of *seco*-diterpenes isolated from the Southwestern Caribbean Sea plume *Antillogorgia kallos*

Jeffrey Marrero[a], Luis A. Amador[a,b], Ivan M. Novitskiy,[c] Andrei G. Kutateladze[c] and Abimael D. Rodríguez*[a,b]

[a] Department of Chemistry, University of Puerto Rico, 17 Ave. Universidad STE 1701, San Juan, PR 00925-2537, United States

[b] Molecular Sciences Research Center, University of Puerto Rico, 1390 Ponce de León Avenue, San Juan, Puerto Rico 00926, United States

[c] Department of Chemistry and Biochemistry, University of Denver, Denver, Colorado 80208, United States

* Corresponding author. Tel.: +1-787-523-5320; fax: +1-787-522-2150; e-mail: abimael.rodriguez1@upr.edu

Supplementary Materials

Contents: **Page**

Scheme S1. Proposed biogenetic pathways for the formation of the kallopterolides S4
Scheme S2. How to read the NMR tables in the SI DU8ML S5
DU8ML and machine learning protocols. S5
Computational details S7
DU8ML data for 3-methyl-2-(2-((R)-5-((S)-2-methyl-1-((S)-4-methyl-5-oxo-2,5-dihydrofuran-2-yl)allyl)-2-oxo-2,5-dihydrofuran-3-yl)ethyl)but-2-enal (**6S,7S,8R diastereomer**) S9
DU8ML data for 3-methyl-2-(2-((S)-5-((S)-2-methyl-1-((S)-4-methyl-5-oxo-2,5-dihydrofuran-2-yl)allyl)-2-oxo-2,5-dihydrofuran-3-yl)ethyl)but-2-enal (**6S,7S,8S diastereomer**) S32
DU8ML data for 3-methyl-2-(2-((R)-5-((S)-2-methyl-1-((R)-4-methyl-5-oxo-2,5-dihydrofuran-2-yl)allyl)-2-oxo-2,5-dihydrofuran-3-yl)ethyl)but-2-enal (**6R,7S,8R diastereomer**) S48
DU8ML data for 3-methyl-2-(2-((S)-5-((S)-2-methyl-1-((R)-4-methyl-5-oxo-2,5-dihydrofuran-2-yl)allyl)-2-oxo-2,5-dihydrofuran-3-yl)ethyl)but-2-enal (**6R,7S,8S diastereomer**) S71
DU8ML data for 2-(2-((S)-5-((S,E)-2-hydroxy-2-methyl-3-(4-methyl-5-oxofuran-2(5H)-ylidene)propyl)-2-oxo-2,5-dihydrofuran-3-yl)ethyl)-3-methylbut-2-enal (**10S,8S,6E diastereomer**) S103
DU8ML data for 2-(2-((S)-5-((S,Z)-2-hydroxy-2-methyl-3-(4-methyl-5-oxofuran-2(5H)-ylidene)propyl)-2-oxo-2,5-dihydrofuran-3-yl)ethyl)-3-methylbut-2-enal (**10S,8S,6Z diastereomer**) S112
DU8ML data for 2-(2-((S)-5-((R,Z)-2-hydroxy-2-methyl-3-(4-methyl-5-oxofuran-2(5H)-ylidene)propyl)-2-oxo-2,5-dihydrofuran-3-yl)ethyl)-3-methylbut-2-enal (**10S,8R,6Z diastereomer**) S134
DU8ML data for 2-(2-((S)-5-((R,E)-2-hydroxy-2-methyl-3-(4-methyl-5-oxofuran-2(5H)-ylidene)propyl)-2-oxo-2,5-dihydrofuran-3-yl)ethyl)-3-methylbut-2-enal (**10S,8R,6E diastereomer**) S149
Figure S1. ^{1}H-NMR spectrum ($CDCl_3$, 300 MHz) of Kallopterolide D (**4**) S169
Figure S2. ^{13}C-NMR spectrum ($CDCl_3$, 75 MHz) of Kallopterolide D (**4**) S170
Figure S3. DEPT-135 spectrum ($CDCl_3$, 75 MHz) of Kallopterolide D (**4**) S171
Figure S4. 1H-1H-COSY spectrum (CDCl3) of Kallopterolide D (**4**) S172
Figure S5. ^{1}H-^{13}C-HMQC spectrum ($CDCl_3$) of Kallopterolide D (**4**) S173
Figure S6. ^{1}H-^{13}C-HMBC spectrum ($CDCl_3$) of Kallopterolide D (**4**) S174
Figure S7. ^{1}H-^{1}H-NOESY spectrum ($CDCl_3$) of Kallopterolide D (**4**) S175
Figure S8. FTIR spectrum of Kallopterolide D (**4**) S176
Figure S9. HRFAB-MS (glycerol) spectrum of Kallopterolide D (**4**) S177
Figure S10. ^{1}H-NMR spectrum ($CDCl_3$, 300 MHz) of Kallopterolide A (**1**) S178
Figure S11. ^{13}C-NMR spectrum ($CDCl_3$, 75 MHz) of Kallopterolide A (**1**) S179
Figure S12. ^{1}H-^{1}H-NOESY spectrum ($CDCl_3$) of Kallopterolide A (**1**) S180
Figure S13. HRFAB-MS (glycerol) spectrum of Kallopterolide A (**1**) S181
Figure S14. ^{1}H-NMR spectrum ($CDCl_3$, 300 MHz) of Kallopterolide B (**2**) S182
Figure S15. ^{13}C-NMR spectrum ($CDCl_3$, 75 MHz) of Kallopterolide B (**2**) S183
Figure S16. ^{1}H-^{1}H-NOESY spectrum ($CDCl_3$) of Kallopterolide B (**2**) S184
Figure S17. ^{1}H-NMR spectrum ($CDCl_3$, 300 MHz) of Kallopterolide C (**3**) S185
Figure S18. ^{13}C-NMR spectrum ($CDCl_3$, 75 MHz) of Kallopterolide C (**3**) S186

Figure S19. ^{1}H-^{1}H-NOESY spectrum ($CDCl_3$) of Kallopterolide C (**3**) S187
Figure S20. LR-EI-MS spectrum of Kallopterolide C (**3**) S188
Figure S21. ^{1}H-NMR spectrum ($CDCl_3$, 300 MHz) of Kallopterolide E (**5**) S189
Figure S22. ^{13}C-NMR spectrum ($CDCl_3$, 75 MHz) of Kallopterolide E (**5**) S190
Figure S23. ^{1}H-^{1}H-NOESY spectrum ($CDCl_3$) of Kallopterolide E (**5**) S191
Figure S24. LR-EI-MS spectrum of Kallopterolide E (**5**) S192
Figure S25. ^{1}H-NMR spectrum ($CDCl_3$, 300 MHz) of Kallopterolide F (**6**) S193
Figure S26. ^{13}C-NMR spectrum ($CDCl_3$, 75 MHz) of Kallopterolide F (**6**) S194
Figure S27. ^{1}H-^{1}H-NOESY spectrum ($CDCl_3$) of Kallopterolide F (**6**) S195
Figure S28. ^{1}H-NMR spectrum ($CDCl_3$, 300 MHz) of Kallopterolide G (**7**) S196
Figure S29. ^{13}C-NMR spectrum ($CDCl_3$, 75 MHz) of Kallopterolide G (**7**) S197
Figure S30. ^{1}H-^{1}H-NOESY spectrum ($CDCl_3$) of Kallopterolide G (**7**) S198
Figure S31. ^{1}H-NMR spectrum ($CDCl_3$, 300 MHz) of Kallopterolide H (**8**) S199
Figure S32. ^{13}C-NMR spectrum ($CDCl_3$, 75 MHz) of Kallopterolide H (**8**) S200
Figure S33. ^{1}H-^{1}H-NOESY spectrum ($CDCl_3$) of Kallopterolide H (**8**) S201
Figure S34. ^{1}H-NMR spectrum ($CDCl_3$, 300 MHz) of Kallopterolide I (**9**) S202
Figure S35. ^{13}C-NMR spectrum ($CDCl_3$, 75 MHz) of Kallopterolide I (**9**) S203
Table S1. ^{1}H NMR (500 MHz) spectroscopic data for kallopterolide A (**1**) in CD_3OD S204
Table S2. ^{1}H NMR (500 MHz) and ^{13}C NMR (125 MHz) spectral data for kallopterolide A (**1**), kallopterolide B (**2**), and caucanolide A S205
Table S3. ^{1}H NMR (500 MHz) and ^{13}C NMR (125 MHz) spectral data for kallopterolide D (**4**), caucanolide E, kallopterolide E (**5**) and caucanolide F S206
Table S4. ^{1}H NMR (500 MHz) and ^{13}C NMR (125 MHz) spectral data for kallopterolide F (**6**), caucanolide D, and kallopterolide G (**7**) S207

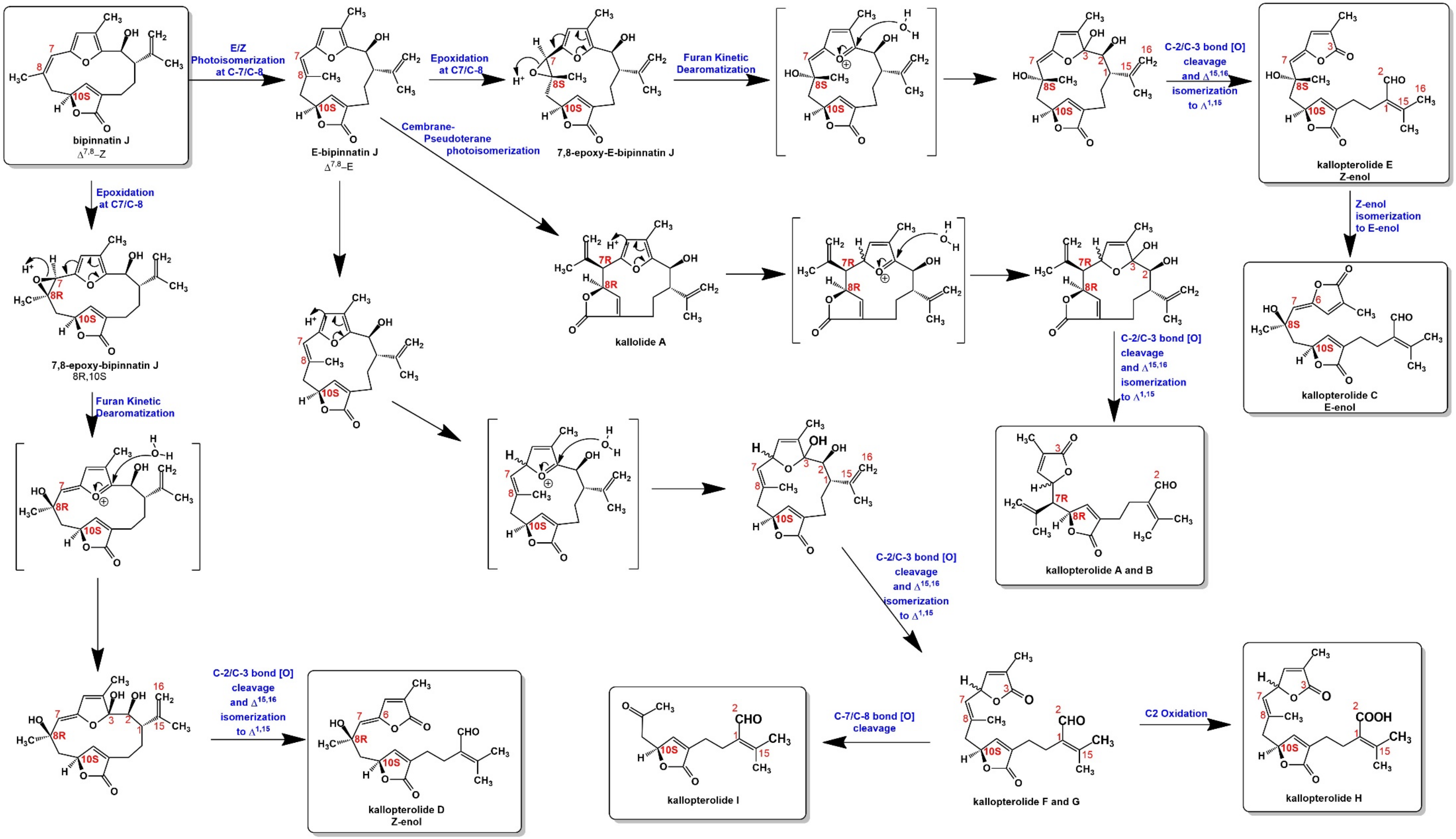

Scheme S1. Proposed biogenetic pathways for the formation of the kallopterolides.

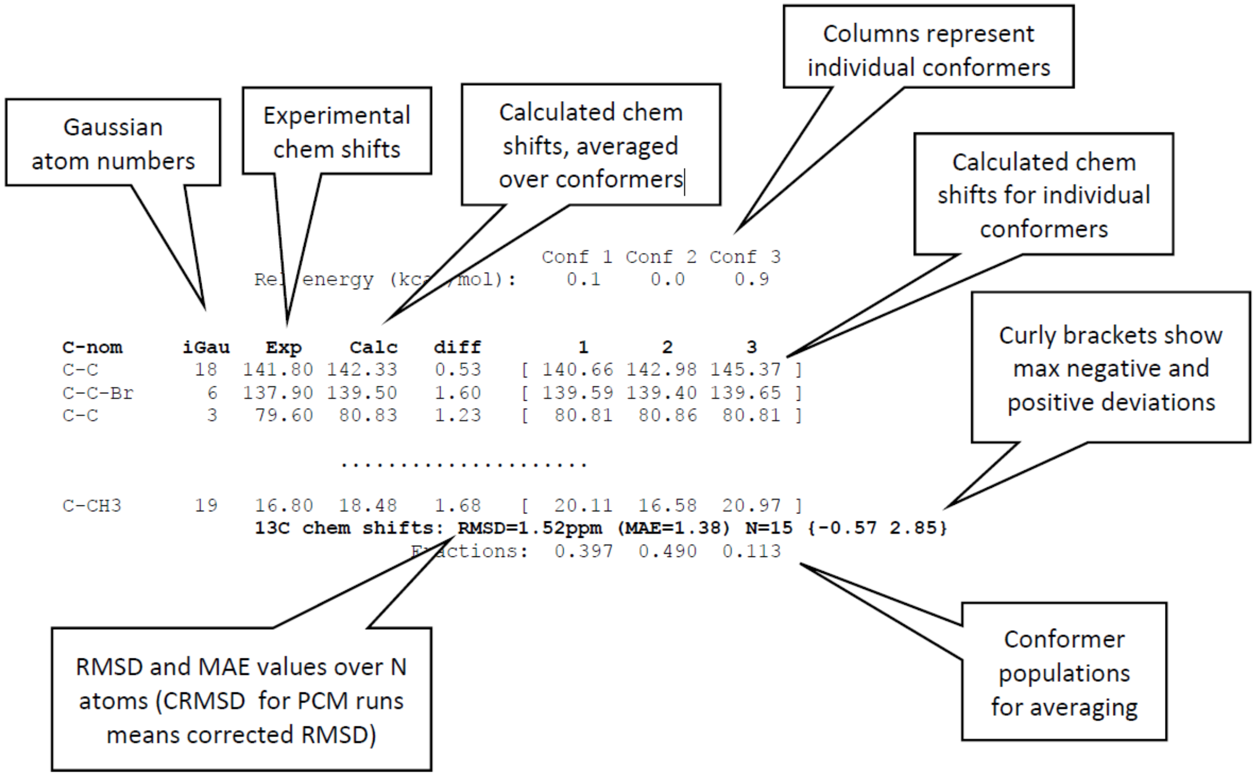

Scheme S2. How to read the NMR tables in the SI DU8ML

DU8ML and machine learning protocols.

The training set of reliable ^{13}C NMR chemical shifts (currently, approaching 28,000 entries), is matched with the calculated values (obtained as described above for DU8+) and is analyzed/re-sorted for largest deviations from the experimental data. The structural fragments responsible for the highest deviations are "labeled" with SMARTS strings, and a single fixed value parametric correction χ_{par} is assigned to each entry. The DFT-calculated isotropic magnetic shielding values, I_{iso}^{DFT}, are then individually corrected according to the formula, Eq S1:

$$I'_{iso} = I_{iso}^{DFT} + \chi_{par} \frac{A - I_{iso}^{DFT}}{B} \qquad \textbf{(Eq S1)}$$

i.e. the fixed value parametric corrections, χ_{par}, are additionally scaled by a linear function of I_{iso}^{DFT} (notice that the parameters A and B are global in the current implementation of DU8ML).

Due to the large size of the training set, we found it "safe" to convert the corrected magnetic shielding values I'_{iso} into ppm chemical shifts using a quadratic formula, Eq S2:

$$\delta(ppm) = \; a\,(I'_{iso})^2 + b\,I'_{iso} + c \qquad \textbf{(Eq S2)}$$

In the current implementation of DU8ML, with the addition of new structures to the training set, any carbon atom could be a member of up to six overlapping SMARTS substructures, with corrections applied cumulatively. The individual χ_{par} values, together with the global A, B and a, b, c values, are reoptimized with the addition of new entries to the training set. Effectively, this could mean that as the system "learns," some of the earlier introduced χ_{par} values change their magnitude, or could decrease below a threshold, at which point they are "retired."

The current set of SMARTS substructures has 600+ entries, which translates into more than 40 points in the training set on average (for statistical significance, every SMARTS substructure corresponds to no less than 10 training set points).

ADDITIONAL LAYERS

Heavy atom

As described above the I_{iso}^{DFT} values calculated for carbons bearing heavy atoms are scaled by distinct quadratic functions [for details see *J. Org. Chem.* **2017**, *82* (7), 3368–3381. DOI: 10.1021/acs.joc.7b00188]

Intramolecular hydrogen bonds

Due to the limitations of smaller basis sets (used by design to accelerate the DFT calculations), intramolecular H-bonds are not accurately computed, and the calculated chemical shifts of the involved carbons require additional corrections. The current implementation of DU8ML adds additional LAYER of correction to improve the chemical shift calculations in molecules containing intramolecular hydrogen bonds to carbonyl groups and to double and triple CC bonds. This additional layer of corrections is applied using a simplified Eq S3:

$$\Delta\delta_{H-bond} = C_1\left(\frac{\sin(\varphi)}{d}\right)^2 + C_2 \qquad \textbf{(Eq S3)}$$

where φ is the dihedral angle H--O--C--Z in the shown H-bonded substructure, and d is the distance H----O

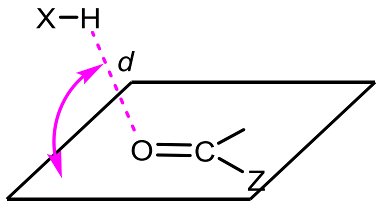

At this juncture, the H-bond training set does not have a statistically significant number of entries to introduce the angle X---H---O into the equation. As soon as we clear this threshold, the next generation of Eq S3 will have this angle as an additional argument. Nonetheless, even this first-generation approximation allows for a considerable improvement of rmsd (reduction by 0.6-0.8ppm) in molecules with intramolecular H-bonds.

Solvent.

The current implementation of DU8ML is developed to predict the absolute chemical shifts in chloroform (i.e. the calculated chemical shift values are not subjected to additional linear scaling to account for solvent effects). For these calculations we report the actual rmsd values.

However, for other solvents, as it is universally accepted, an additional linear correction is applied to match the calculated and experimental values. In these cases, we report the corrected rmsd, i.e. crmsd values.

COMPUTATIONAL DETAILS

Computations were performed using Gaussian 16, Revision C.01

M. J. Frisch, G. W. Trucks, H. B. Schlegel, G. E. Scuseria, M. A. Robb, J. R. Cheeseman, G. Scalmani, V. Barone, G. A. Petersson, H. Nakatsuji, X. Li, M. Caricato, A. V. Marenich, J. Bloino, B. G. Janesko, R. Gomperts, B. Mennucci, H. P. Hratchian, J. V. Ortiz, A. F. Izmaylov, J. L. Sonnenberg, D. Williams-Young, F. Ding, F. Lipparini, F. Egidi, J. Goings, B. Peng, A. Petrone, T. Henderson, D. Ranasinghe, V. G. Zakrzewski, J. Gao, N. Rega, G. Zheng, W. Liang, M. Hada, M. Ehara, K. Toyota, R. Fukuda, J. Hasegawa, M. Ishida, T. Nakajima, Y. Honda, O. Kitao, H. Nakai, T. Vreven, K. Throssell, J. A. Montgomery, Jr., J. E. Peralta, F. Ogliaro, M. J. Bearpark, J. J. Heyd, E. N. Brothers, K. N. Kudin, V. N. Staroverov, T. A. Keith, R. Kobayashi, J. Normand, K. Raghavachari, A. P. Rendell, J. C. Burant, S. S. Iyengar, J. Tomasi, M. Cossi, J. M. Millam, M. Klene, C. Adamo, R. Cammi, J. W. Ochterski, R. L. Martin, K. Morokuma, O. Farkas, J. B. Foresman, and D. J. Fox, Gaussian, Inc., Wallingford CT, 2019.

DU8ML workflow

(I) Structure optimization: B3LYP/6-31G(d);

(II) Magnetic shielding: ωB97xD/6-31G(d);

(III) Fermi contacts: B3LYP/DU8;

(IV) Scaling of the computed Fermi contacts according to [Kutateladze, A. G.; Mukhina, O. A. Minimalist Relativistic Force Field: Prediction of Proton–Proton Coupling Constants in 1H NMR Spectra Is Perfected with NBO Hybridization Parameters. *J.*

Org. Chem. **2015**, *80* (10), 5218–5225. DOI: 10.1021/acs.joc.5b00619] to obtain spin-spin coupling constants;

(V) Scaling of isotropic magnetic shielding values according to [Kutateladze, A. G.; Reddy, D. S. High-Throughput in Silico Structure Validation and Revision of Halogenated Natural Products Is Enabled by Parametric Corrections to DFT-Computed 13C NMR Chemical Shifts and Spin–Spin Coupling Constants. *J. Org. Chem.* **2017**, *82* (7), 3368–3381. DOI: 10.1021/acs.joc.7b00188] to obtain chemical shifts.

The initial structures were created with Chem3D and pre-optimized with the force field MMFF94 as implemented in OpenBabel. For structures with freely rotatable groups, conformers were generated using OpenBabel's confab, whereas the conformers resulting from conformational changes in cyclic cores were generated manually, using Chem3D.

DU8ML data for 3-methyl-2-(2-((*R*)-5-((*S*)-2-methyl-1-((*S*)-4-methyl-5-oxo-2,5-dihydrofuran-2-yl)allyl)-2-oxo-2,5-dihydrofuran-3-yl)ethyl)but-2-enal (**6*S*,7*S*,8*R* diastereomer**)

NMR parameters calculated for 6*S*,7*S*,8*R* diastereomer vs experimental data of kallopterolide A (**1**)

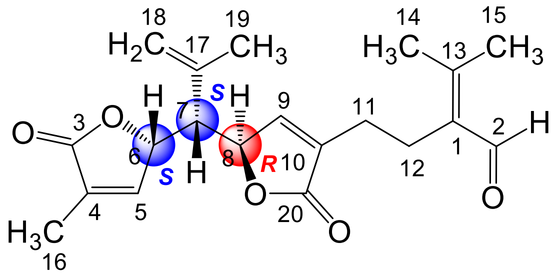

Parameter	RMSD
J_{HH}	1.73 Hz
δ_{1H}	0.19 ppm
δ_{13C}	1.49 ppm

	Conf1	Conf2	Conf3	Conf4	Conf5	Conf6	Conf7	Conf8	Conf9	Conf10	Conf11	Conf12	Conf13
Rel energy (kcal/mol):	0.00	0.15	0.15	0.18	0.19	0.19	0.22	0.32	0.34	0.43	0.44	0.59	0.82

Conf14	Conf15	Conf16	Conf17	Conf18	Conf19	Conf20
0.85	0.91	0.92	0.93	0.94	0.98	1.00

iGau	jGau	Jexp	Jcalc	diff		1	2	3	4	5	6	7	8	9	10	11	12	13
30	31	1.60	1.52	-0.08	[	-1.54	-1.52	-1.52	-1.52	-1.54	-1.52	-1.52	-1.52	-1.52	-1.52	-1.52	-1.52	-1.54
22	29	4.00	7.08	3.08	[	11.93	1.38	5.96	1.45	11.91	5.98	10.23	1.37	10.61	1.38	9.97	10.12	11.92
22	30	2.00	2.02	0.02	[	1.93	2.11	2.02	2.10	1.93	2.03	1.98	2.10	1.98	2.10	2.02	2.01	1.89
22	31	1.90	1.92	0.02	[	1.79	2.01	1.96	2.01	1.79	1.96	1.88	2.01	1.90	2.01	1.90	1.91	1.80
23	29	10.00	7.10	-2.90	[	5.93	10.14	11.95	10.15	6.04	11.96	1.33	9.92	0.84	9.86	1.37	1.12	4.33
23	28	1.60	2.00	0.40	[	1.99	1.97	1.91	1.95	1.99	1.89	2.09	2.00	2.17	1.99	2.07	2.12	2.03

14	15	16	17	18	19	20			
-1.52	-1.51	-1.54	-1.52	-1.51	-1.52	-1.51	]	H5	H16-Me
10.64	1.56	11.91	10.52	4.33	5.90	4.38	]	H6	H7
1.95	2.09	1.89	1.96	2.06	2.01	2.06	]	H6	H5
1.88	1.98	1.80	1.88	2.03	1.96	2.03	]	H6	H16-Me
1.05	10.17	4.31	1.09	11.96	11.80	11.94	]	H8	H7
2.12	1.95	2.02	2.13	1.85	1.95	1.88	]	H8	H9

For Js: RMSD=1.73Hz N=6 {-2.90 3.08}

NOTICE:
removed constants H11 and H12 from A-exp set, b/c they are not available in the B-exp set

H-nom	iGau	Exp	Calc	diff		1	2	3	4	5	6	7	8	9	10	11	12	13
H2	49	10.10	10.09	-0.01	[	10.09	10.09	10.09	10.08	10.10	10.08	10.11	10.09	10.13	10.08	10.11	10.13	10.10
H5	30	7.23	7.32	0.09	[	7.31	7.34	7.46	7.33	7.28	7.46	7.19	7.30	7.22	7.28	7.21	7.19	7.29

H6	22	5.35	5.09	-0.26	[	4.93	4.98	5.38	4.96	4.89	5.37	5.06	5.06	5.21	5.04	4.92	4.98	5.09
H7	29	2.41	2.51	0.10	[	2.68	2.48	2.70	2.44	2.68	2.65	2.48	2.13	2.52	2.09	2.11	2.15	2.87
H8	23	5.23	5.09	-0.14	[	5.40	5.09	4.91	5.10	5.41	4.93	5.00	4.95	4.95	4.97	5.08	5.05	5.40
H9	28	7.31	7.37	0.06	[	7.52	7.29	7.36	7.29	7.54	7.36	7.40	7.29	7.38	7.31	7.36	7.37	7.54
H11	34	2.27	2.42	0.15	[	2.23	2.17	2.20	2.58	2.64	2.59	2.67	2.19	2.17	2.60	2.67	2.17	2.24
H11	35	2.27	2.41	0.14	[	2.63	2.55	2.61	2.14	2.20	2.22	2.30	2.56	2.71	2.16	2.30	2.71	2.61
H12	36	2.52	2.58	0.06	[	2.50	2.51	2.56	2.48	2.53	2.56	2.59	2.53	2.90	2.49	2.58	2.87	2.59
H12	37	2.52	2.55	0.03	[	2.52	2.50	2.55	2.50	2.57	2.54	2.58	2.53	2.46	2.51	2.58	2.44	2.54
H14-Me	41	2.04	2.33	0.29	[	2.33	2.33	2.34	2.31	2.33	2.34	2.37	2.33	2.32	2.31	2.37	2.32	2.34
H15-Me	38	2.22	2.28	0.06	[	2.28	2.27	2.28	2.27	2.28	2.28	2.28	2.28	2.29	2.27	2.28	2.29	2.28
H16-Me	31	1.86	2.05	0.19	[	2.06	2.09	2.07	2.09	2.05	2.06	1.98	2.09	2.00	2.09	2.00	2.02	2.06
H18a	45	4.89	5.21	0.32	[	4.95	5.21	4.85	5.14	4.80	4.86	5.17	5.87	5.17	5.89	5.86	5.86	5.26
H18b	44	5.07	5.39	0.32	[	5.46	5.29	5.43	5.24	5.39	5.44	5.25	5.58	5.25	5.58	5.58	5.57	5.38
H19-Me	46	1.72	2.02	0.30	[	1.96	2.16	1.99	2.17	1.94	1.94	2.15	2.00	2.15	1.93	1.95	1.96	1.86

14	15	16	17	18	19	20	
10.14	10.02	10.10	10.14	10.09	9.98	10.09	]
7.25	7.50	7.28	7.25	7.48	7.42	7.48	]
5.15	4.93	5.06	5.19	5.37	5.29	5.39	]
2.51	2.55	2.86	2.47	2.83	2.41	2.89	]
5.00	4.94	5.42	4.95	5.10	4.84	5.08	]
7.30	6.84	7.55	7.28	7.35	7.48	7.35	]
2.17	2.63	2.64	2.79	2.61	2.67	2.21	]
2.50	2.22	2.19	2.13	2.21	2.40	2.61	]
3.05	3.04	2.54	2.40	2.56	2.22	2.56	]
2.59	2.33	2.56	3.15	2.56	3.27	2.53	]
2.31	2.19	2.33	2.33	2.34	2.34	2.34	]
2.30	2.25	2.28	2.29	2.27	2.28	2.28	]
2.01	2.10	2.05	2.01	2.07	2.05	2.06	]
5.18	5.14	5.24	5.16	5.24	4.77	5.29	]
5.26	5.16	5.35	5.25	5.37	5.39	5.41	]
2.15	2.08	1.75	2.16	1.81	2.11	1.80	]

1H chem shifts: RMSD=0.19ppm (MAE=0.16) N=16 {-0.26 0.32}
m=1.000 b=0.00

C-nom	iGau	Exp	Calc	diff		1	2	3	4	5	6	7	8	9	10	11	12	13
C1-C**	18	135.50	136.72	1.22	[	136.72	136.62	136.73	136.74	136.73	136.67	136.81	136.61	137.21	136.74	136.85	137.15	136.68
C2-CH	26	190.60	189.46	-1.14	[	189.43	189.33	189.45	189.38	189.43	189.42	189.34	189.33	189.43	189.40	189.30	189.36	189.42
C3-C	10	173.80	173.24	-0.56	[	172.96	173.90	173.14	173.82	172.93	173.13	172.76	174.12	172.60	174.06	172.81	172.67	172.77
C4-C	11	130.90	131.46	0.56	[	131.77	129.09	134.19	129.03	131.89	134.24	131.42	129.66	131.35	129.65	132.16	132.15	131.68
C5-CH	12	146.80	145.76	-1.04	[	145.83	147.94	142.41	147.89	145.72	142.38	146.03	147.91	146.05	147.90	146.09	145.82	145.40
C6-CH	7	79.80	80.58	0.78	[	80.54	83.54	81.66	83.62	80.57	81.69	76.57	82.18	77.09	82.17	78.76	79.23	78.01
C7-CH	6	53.50	53.33	-0.17	[	51.68	55.83	51.43	55.83	51.53	51.49	55.75	51.67	55.28	51.67	51.62	51.41	55.03
C8-CH	1	80.10	80.67	0.57	[	81.88	77.05	80.92	77.03	82.06	80.95	83.90	79.21	83.21	79.16	82.55	82.76	80.45
C9-CH	5	147.40	145.91	-1.49	[	142.66	146.42	146.02	146.37	142.65	146.01	148.22	146.45	148.19	146.46	148.18	148.25	142.52
C10-C	4	134.60	134.84	0.24	[	137.47	134.63	134.98	134.62	137.40	134.99	132.26	135.39	132.74	135.35	132.81	132.90	136.06

C11-CH2	16	24.50	26.93	2.43	[	27.33	27.05	26.96	26.96	27.18	27.03	27.18	27.12	26.53	27.00	27.22	26.61	27.37
C12-CH2	17	23.40	25.47	2.07	[	26.12	25.52	25.62	25.50	25.58	25.37	24.87	25.59	26.45	25.53	24.92	26.34	25.50
C13-C**	19	156.80	158.72	1.92	[	158.80	158.73	158.78	158.67	158.67	158.77	158.60	158.82	158.06	158.66	158.64	158.07	159.00
C14-CH3	21	23.40	21.71	-1.69	[	21.70	21.68	21.68	21.67	21.65	21.68	21.74	21.68	21.70	21.67	21.74	21.67	21.71
C15-CH3	20	19.40	17.07	-2.33	[	17.06	17.02	17.06	17.08	17.05	17.08	16.98	17.00	17.25	17.07	16.99	17.23	17.01
C16-CH3	14	10.80	11.16	0.36	[	11.15	11.10	11.31	11.10	11.10	11.32	11.09	11.14	11.10	11.13	11.11	11.12	11.15
C17-C	8	137.80	141.24	3.44	[	138.61	144.21	138.90	144.53	138.75	138.72	144.43	142.46	144.18	142.26	142.46	142.93	135.65
C18-CH2	24	117.10	116.51	-0.59	[	115.22	116.70	114.84	116.47	114.90	115.00	116.48	117.01	116.34	117.22	116.88	116.64	121.90
C19-CH3	25	23.70	22.95	-0.75	[	26.05	19.49	26.05	19.54	26.05	26.04	19.43	25.32	18.90	25.33	25.23	25.15	20.02
C20-C	3	172.90	174.10	1.20	[	174.07	173.79	173.87	173.80	174.03	173.82	174.78	173.80	175.03	173.82	175.05	175.15	174.02

**14**	**15**	**16**	**17**	**18**	**19**	**20**	
136.66	136.45	136.79	137.18	136.65	134.32	136.70	]
189.66	191.14	189.46	189.63	189.43	190.19	189.44	]
173.22	174.10	172.79	173.31	173.06	173.16	173.05	]
131.17	128.30	131.73	131.19	132.78	133.92	132.80	]
146.67	148.70	145.33	146.69	142.40	142.49	142.41	]
77.25	84.37	77.96	77.26	80.09	81.71	80.11	]
55.80	54.52	55.04	55.71	55.05	51.76	55.00	]
83.41	76.45	80.44	82.90	78.41	80.60	78.40	]
147.21	144.10	142.66	148.21	145.61	149.08	145.60	]
134.25	134.43	135.95	133.42	134.83	133.30	134.83	]
25.65	27.61	27.15	27.09	27.12	22.04	26.99	]
23.98	23.44	25.60	25.27	25.28	23.96	25.62	]
158.33	159.31	158.74	157.30	158.86	162.44	158.82	]
21.74	21.90	21.63	21.69	21.65	22.75	21.73	]
17.12	16.74	17.05	17.35	17.06	17.26	17.09	]
11.05	11.09	11.16	11.06	11.33	11.30	11.33	]
144.13	145.33	135.50	144.29	135.48	140.08	135.33	]
116.52	115.59	121.92	116.37	122.09	113.99	122.34	]
19.03	19.59	19.84	19.11	19.79	25.56	19.84	]
173.97	172.89	173.98	173.35	173.69	174.55	173.72	]

13C chem shifts: RMSD=1.49ppm (MAE=1.23) N=20 {-2.33 3.44}

Fractions: 0.104 0.081 0.080 0.077 0.076 0.076 0.071 0.061 0.058 0.050 0.050 0.038 0.026 0.025 0.022 0.022 0.022 0.021 0.020 0.019

NMR parameters calculated for 6*S*,7*S*,8*R* diastereomer vs experimental data of kallopterolide B (**2**)

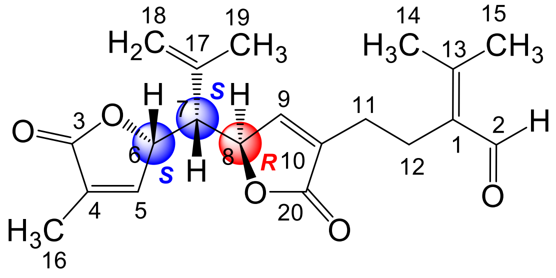

Parameter	RMSD
J_{HH}	0.42 Hz
δ_{1H}	0.20 ppm
δ_{13C}	1.32 ppm

						Conf1	Conf2	Conf3	Conf4	Conf5	Conf6	Conf7	Conf8	Conf9	Conf10	Conf11	Conf12	Conf13
		Rel energy (kcal/mol):				0.00	0.15	0.15	0.18	0.19	0.19	0.22	0.32	0.34	0.43	0.44	0.59	0.82
**iGau**	**jGau**	**Jexp**	**Jcalc**	**diff**		**1**	**2**	**3**	**4**	**5**	**6**	**7**	**8**	**9**	**10**	**11**	**12**	**13**
22	30	1.65	2.02	0.37	[	1.93	2.11	2.02	2.10	1.93	2.03	1.98	2.10	1.98	2.10	2.02	2.01	1.89
30	31	1.50	1.52	0.02	[	-1.54	-1.52	-1.52	-1.52	-1.54	-1.52	-1.52	-1.52	-1.52	-1.52	-1.52	-1.52	-1.54
22	29	7.00	7.08	0.08	[	11.93	1.38	5.96	1.45	11.91	5.98	10.23	1.37	10.61	1.38	9.97	10.12	11.92
22	31	1.70	1.92	0.22	[	1.79	2.01	1.96	2.01	1.79	1.96	1.88	2.01	1.90	2.01	1.90	1.91	1.80
23	29	7.80	7.10	-0.70	[	5.93	10.14	11.95	10.15	6.04	11.96	1.33	9.92	0.84	9.86	1.37	1.12	4.33
23	28	1.40	2.00	0.60	[	1.99	1.97	1.91	1.95	1.99	1.89	2.09	2.00	2.17	1.99	2.07	2.12	2.03

Conf14	Conf15	Conf16	Conf17	Conf18	Conf19	Conf20			
0.85	0.91	0.92	0.93	0.94	0.98	1.00			
**14**	**15**	**16**	**17**	**18**	**19**	**20**			
1.95	2.09	1.89	1.96	2.06	2.01	2.06	]	H6	H5
-1.52	-1.51	-1.54	-1.52	-1.51	-1.52	-1.51	]	H5	H16-Me
10.64	1.56	11.91	10.52	4.33	5.90	4.38	]	H6	H7
1.88	1.98	1.80	1.88	2.03	1.96	2.03	]	H6	H16-Me
1.05	10.17	4.31	1.09	11.96	11.80	11.94	]	H8	H7
2.12	1.95	2.02	2.13	1.85	1.95	1.88	]	H8	H9

For Js: RMSD=0.42Hz N=6 {-0.70 0.60}

**H-nom**	**iGau**	**Exp**	**Calc**	**diff**		**1**	**2**	**3**	**4**	**5**	**6**	**7**	**8**	**9**	**10**	**11**	**12**	**13**
H2	49	10.10	10.09	-0.01	[	10.09	10.09	10.09	10.08	10.10	10.08	10.11	10.09	10.13	10.08	10.11	10.13	10.10
H5	30	7.10	7.32	0.22	[	7.31	7.34	7.46	7.33	7.28	7.46	7.19	7.30	7.22	7.28	7.21	7.19	7.29
H6	22	5.05	5.09	0.04	[	4.93	4.98	5.38	4.96	4.89	5.37	5.06	5.06	5.21	5.04	4.92	4.98	5.09
H7	29	2.61	2.51	-0.10	[	2.68	2.48	2.70	2.44	2.68	2.65	2.48	2.13	2.52	2.09	2.11	2.15	2.87
H8	23	5.07	5.09	0.02	[	5.40	5.09	4.91	5.10	5.41	4.93	5.00	4.95	4.95	4.97	5.08	5.05	5.40
H9	28	7.13	7.37	0.24	[	7.52	7.29	7.36	7.29	7.54	7.36	7.40	7.29	7.38	7.31	7.36	7.37	7.54
H11	34	2.28	2.42	0.14	[	2.23	2.17	2.20	2.58	2.64	2.59	2.67	2.19	2.17	2.60	2.67	2.17	2.24
H11	35	2.28	2.41	0.13	[	2.63	2.55	2.61	2.14	2.20	2.22	2.30	2.56	2.71	2.16	2.30	2.71	2.61
H12	36	2.52	2.58	0.06	[	2.50	2.51	2.56	2.48	2.53	2.56	2.59	2.53	2.90	2.49	2.58	2.87	2.59
H12	37	2.52	2.55	0.03	[	2.52	2.50	2.55	2.50	2.57	2.54	2.58	2.53	2.46	2.51	2.58	2.44	2.54

H14-Me	41	2.03	2.33	0.30	[	2.33	2.33	2.34	2.31	2.33	2.34	2.37	2.33	2.32	2.31	2.37	2.32	2.34
H15-Me	38	2.21	2.28	0.07	[	2.28	2.27	2.28	2.27	2.28	2.28	2.28	2.28	2.29	2.27	2.28	2.29	2.28
H16-Me	31	1.95	2.05	0.10	[	2.06	2.09	2.07	2.09	2.05	2.06	1.98	2.09	2.00	2.09	2.00	2.02	2.06
H18a	44	4.83	5.39	0.56	[	5.46	5.29	5.43	5.24	5.39	5.44	5.25	5.58	5.25	5.58	5.58	5.57	5.38
H18b	45	5.08	5.21	0.13	[	4.95	5.21	4.85	5.14	4.80	4.86	5.17	5.87	5.17	5.89	5.86	5.86	5.26
H19-Me	46	1.81	2.02	0.21	[	1.96	2.16	1.99	2.17	1.94	1.94	2.15	2.00	2.15	1.93	1.95	1.96	1.86

**14**	**15**	**16**	**17**	**18**	**19**	**20**	
10.14	10.02	10.10	10.14	10.09	9.98	10.09	]
7.25	7.50	7.28	7.25	7.48	7.42	7.48	]
5.15	4.93	5.06	5.19	5.37	5.29	5.39	]
2.51	2.55	2.86	2.47	2.83	2.41	2.89	]
5.00	4.94	5.42	4.95	5.10	4.84	5.08	]
7.30	6.84	7.55	7.28	7.35	7.48	7.35	]
2.17	2.63	2.64	2.79	2.61	2.67	2.21	]
2.50	2.22	2.19	2.13	2.21	2.40	2.61	]
3.05	3.04	2.54	2.40	2.56	2.22	2.56	]
2.59	2.33	2.56	3.15	2.56	3.27	2.53	]
2.31	2.19	2.33	2.33	2.34	2.34	2.34	]
2.30	2.25	2.28	2.29	2.27	2.28	2.28	]
2.01	2.10	2.05	2.01	2.07	2.05	2.06	]
5.26	5.16	5.35	5.25	5.37	5.39	5.41	]
5.18	5.14	5.24	5.16	5.24	4.77	5.29	]
2.15	2.08	1.75	2.16	1.81	2.11	1.80	]

1H chem shifts: RMSD=0.20ppm (MAE=0.15) N=16 {-0.10 0.56}

m=1.000 b=0.00

**C-nom**	**iGau**	**Exp**	**Calc**	**diff**		**1**	**2**	**3**	**4**	**5**	**6**	**7**	**8**	**9**	**10**	**11**	**12**	**13**
C1-C**	18	135.40	136.72	1.32	[	136.72	136.62	136.73	136.74	136.73	136.67	136.81	136.61	137.21	136.74	136.85	137.15	136.68
C2-CH	26	190.70	189.46	-1.24	[	189.43	189.33	189.45	189.38	189.43	189.42	189.34	189.33	189.43	189.40	189.30	189.36	189.42
C3-C	10	173.50	173.24	-0.26	[	172.96	173.90	173.14	173.82	172.93	173.13	172.76	174.12	172.60	174.06	172.81	172.67	172.77
C4-C	11	130.90	131.46	0.56	[	131.77	129.09	134.19	129.03	131.89	134.24	131.42	129.66	131.35	129.65	132.16	132.15	131.68
C5-CH	12	147.00	145.76	-1.24	[	145.83	147.94	142.41	147.89	145.72	142.38	146.03	147.91	146.05	147.90	146.09	145.82	145.40
C6-CH	7	80.00	80.58	0.58	[	80.54	83.54	81.66	83.62	80.57	81.69	76.57	82.18	77.09	82.17	78.76	79.23	78.01
C7-CH	6	52.80	53.33	0.53	[	51.68	55.83	51.43	55.83	51.53	51.49	55.75	51.67	55.28	51.67	51.62	51.41	55.03
C8-CH	1	79.90	80.67	0.77	[	81.88	77.05	80.92	77.03	82.06	80.95	83.90	79.21	83.21	79.16	82.55	82.76	80.45
C9-CH	5	146.80	145.91	-0.89	[	142.66	146.42	146.02	146.37	142.65	146.01	148.22	146.45	148.19	146.46	148.18	148.25	142.52
C10-C	4	134.60	134.84	0.24	[	137.47	134.63	134.98	134.62	137.40	134.99	132.26	135.39	132.74	135.35	132.81	132.90	136.06
C11-CH2	16	24.60	26.93	2.33	[	27.33	27.05	26.96	26.96	27.18	27.03	27.18	27.12	26.53	27.00	27.22	26.61	27.37
C12-CH2	17	23.40	25.47	2.07	[	26.12	25.52	25.62	25.50	25.58	25.37	24.87	25.59	26.45	25.53	24.92	26.34	25.50
C13-C**	19	156.90	158.72	1.82	[	158.80	158.73	158.78	158.67	158.67	158.77	158.60	158.82	158.06	158.66	158.64	158.07	159.00
C14-CH3	21	23.40	21.71	-1.69	[	21.70	21.68	21.68	21.67	21.65	21.68	21.74	21.68	21.70	21.67	21.74	21.67	21.71
C15-CH3	20	19.40	17.07	-2.33	[	17.06	17.02	17.06	17.08	17.05	17.08	16.98	17.00	17.25	17.07	16.99	17.23	17.01
C16-CH3	14	10.70	11.16	0.46	[	11.15	11.10	11.31	11.10	11.10	11.32	11.09	11.14	11.10	11.13	11.11	11.12	11.15
C17-C	8	139.20	141.24	2.04	[	138.61	144.21	138.90	144.53	138.75	138.72	144.43	142.46	144.18	142.26	142.46	142.93	135.65
C18-CH2	24	116.80	116.51	-0.29	[	115.22	116.70	114.84	116.47	114.90	115.00	116.48	117.01	116.34	117.22	116.88	116.64	121.90
C19-CH3	25	23.60	22.95	-0.65	[	26.05	19.49	26.05	19.54	26.05	26.04	19.43	25.32	18.90	25.33	25.23	25.15	20.02

```
C20-C       3  173.00 174.10   1.10   [ 174.07 173.79 173.87 173.80 174.03 173.82 174.78 173.80 175.03 173.82 175.05 175.15 174.02

     14     15     16     17     18     19     20
136.66 136.45 136.79 137.18 136.65 134.32 136.70 ]
189.66 191.14 189.46 189.63 189.43 190.19 189.44 ]
173.22 174.10 172.79 173.31 173.06 173.16 173.05 ]
131.17 128.30 131.73 131.19 132.78 133.92 132.80 ]
146.67 148.70 145.33 146.69 142.40 142.49 142.41 ]
  77.25  84.37  77.96  77.26  80.09  81.71  80.11 ]
  55.80  54.52  55.04  55.71  55.05  51.76  55.00 ]
  83.41  76.45  80.44  82.90  78.41  80.60  78.40 ]
147.21 144.10 142.66 148.21 145.61 149.08 145.60 ]
134.25 134.43 135.95 133.42 134.83 133.30 134.83 ]
  25.65  27.61  27.15  27.09  27.12  22.04  26.99 ]
  23.98  23.44  25.60  25.27  25.28  23.96  25.62 ]
158.33 159.31 158.74 157.30 158.86 162.44 158.82 ]
  21.74  21.90  21.63  21.69  21.65  22.75  21.73 ]
  17.12  16.74  17.05  17.35  17.06  17.26  17.09 ]
  11.05  11.09  11.16  11.06  11.33  11.30  11.33 ]
144.13 145.33 135.50 144.29 135.48 140.08 135.33 ]
116.52 115.59 121.92 116.37 122.09 113.99 122.34 ]
  19.03  19.59  19.84  19.11  19.79  25.56  19.84 ]
173.97 172.89 173.98 173.35 173.69 174.55 173.72 ]

               13C chem shifts: RMSD=1.32ppm (MAE=1.12) N=20 {-2.33 2.33}
                              Fractions:  0.104  0.081  0.080  0.077  0.076  0.076  0.071  0.061  0.058  0.050  0.050  0.038  0.026
0.025  0.022  0.022  0.022  0.021  0.020  0.019
```

Conformer 1
Energy: -1152.66039 Hartree (Rel: 0.0 kcal/mol)
XYZ coordinates for conf 1:

C	1.19209	0.72971	-0.96939
O	0.43380	1.95531	-0.88299
C	-0.90500	1.66981	-0.88349
C	-1.07731	0.19821	-0.99309
C	0.14649	-0.34479	-1.03629
C	2.20259	0.68980	0.21171
C	3.06289	-0.59150	0.15721
C	1.54729	0.88750	1.57711
O	3.64079	-0.73580	-1.15809
C	5.00959	-0.80150	-1.05739
C	5.38189	-0.70160	0.37771
C	4.24859	-0.58070	1.08051
O	5.72069	-0.92350	-2.02889
C	6.80959	-0.74270	0.81641
O	-1.74840	2.53941	-0.81639
C	-2.43841	-0.42319	-1.02719
C	-3.15111	-0.33989	0.34841
C	-4.58631	-0.82478	0.29071
C	-5.64741	-0.00828	0.03931
C	-7.09401	-0.44278	-0.00619
C	-5.48641	1.47052	-0.22079
H	2.43769	-1.47520	0.33181
H	1.76829	0.76600	-1.90079
C	0.85019	-0.07669	2.19031
C	1.73470	2.25270	2.19181
C	-4.75131	-2.27499	0.48261
O	-3.81621	-3.04809	0.66301
H	0.38559	-1.39739	-1.13979
H	2.88639	1.52870	0.03511
H	4.14659	-0.48860	2.15521
H	6.89469	-0.66080	1.90311
H	7.28479	-1.67751	0.49651
H	7.37869	0.07429	0.35741
H	-3.04591	0.10141	-1.77539
H	-2.36741	-1.47059	-1.33609
H	-2.60151	-0.96939	1.05461
H	-3.10001	0.68591	0.71941
H	-7.27951	-1.47248	0.29771
H	-7.69031	0.21162	0.64301
H	-7.48981	-0.31078	-1.02209
H	-4.46030	1.78221	-0.42029
H	-6.10830	1.76912	-1.07419
H	-5.85660	2.04262	0.64221
H	0.39249	0.09521	3.16121
H	0.69689	-1.06259	1.76071
H	2.80030	2.47180	2.34531
H	1.34440	3.03151	1.52521
H	1.22540	2.33360	3.15681
H	-5.77971	-2.67638	0.45071

Conformer 2
Energy: -1152.65903 Hartree (Rel: 0.1 kcal/mol)
XYZ coordinates for conf 2:

C	-1.30930	0.41860	-0.58780
O	-0.99360	-0.94810	-0.24020
C	0.35800	-1.14939	-0.33270
C	0.99930	0.11811	-0.76520
C	0.02700	1.02740	-0.91220
C	-2.05320	1.09970	0.58550
C	-3.43440	0.45210	0.87840
C	-2.16741	2.61170	0.39630

O	-4.27680	0.44000	-0.29260
C	-4.77930	-0.82661	-0.48920
C	-4.23310	-1.71840	0.56210
C	-3.43350	-0.97830	1.33990
O	-5.53870	-1.07881	-1.39760
C	-4.58229	-3.17030	0.61430
O	0.86510	-2.22389	-0.08680
C	2.48220	0.21701	-0.94560
C	3.24120	0.16411	0.40620
C	4.74580	0.10571	0.22770
C	5.44820	-1.05758	0.13370
C	6.94760	-1.16398	-0.02190
C	4.78120	-2.41139	0.18520
H	-3.92970	1.08780	1.62490
H	-1.95920	0.38300	-1.46990
C	-1.65541	3.43240	1.32090
C	-2.87161	3.14220	-0.83190
C	5.40060	1.41921	0.11280
O	4.79559	2.48581	0.14160
H	0.14260	2.06051	-1.21740
H	-1.44770	0.92290	1.48280
H	-2.86630	-1.31150	2.20130
H	-4.10619	-3.66240	1.46640
H	-5.66749	-3.30621	0.69090
H	-4.26239	-3.67760	-0.30350
H	2.81530	-0.61839	-1.57420
H	2.73700	1.14401	-1.46830
H	2.99580	1.06941	0.96970
H	2.88450	-0.68899	0.98740
H	7.49100	-0.22328	0.06110
H	7.34400	-1.84898	0.73890
H	7.18930	-1.61238	-0.99480
H	3.69671	-2.38199	0.07210
H	5.19111	-3.05929	-0.60000
H	5.01291	-2.90469	1.14030
H	-1.73061	4.51260	1.22320
H	-1.14581	3.05950	2.20600
H	-2.38050	2.81350	-1.75750
H	-3.90461	2.78380	-0.88850
H	-2.87961	4.23600	-0.82970
H	6.49750	1.42882	-0.01560

Conformer 3
Energy: -1152.65890 Hartree (Rel: 0.2 kcal/mol)
XYZ coordinates for conf 3:

C	-0.96990	-0.28369	-0.94080
O	-0.48609	-1.55099	-0.44210
C	0.88521	-1.54159	-0.41230
C	1.36150	-0.22559	-0.91120
C	0.28020	0.50271	-1.21850
C	-1.91100	0.36230	0.09910
C	-3.00270	-0.64450	0.56050
C	-2.48121	1.69290	-0.38840
O	-3.84530	0.00440	1.53470
C	-5.15730	-0.08961	1.14660
C	-5.21920	-0.84481	-0.13160
C	-3.96280	-1.15900	-0.47200
O	-6.05930	0.38319	1.80190
C	-6.52180	-1.13801	-0.80140
O	1.52491	-2.49869	-0.03210
C	2.82270	0.09232	-0.98340
C	3.45120	0.27952	0.42250
C	4.95790	0.44062	0.36920
C	5.83040	-0.60117	0.46530

C	7.33680	-0.48527	0.44100
C	5.36941	-2.03077	0.62080
H	-2.49740	-1.47610	1.06470
H	-1.51610	-0.49400	-1.86800
C	-3.06391	1.83440	-1.58490
C	-2.34981	2.84410	0.57800
C	5.41889	1.82213	0.15420
O	4.66209	2.77652	0.00980
H	0.25610	1.51081	-1.61500
H	-1.30130	0.55690	0.98940
H	-3.64799	-1.72900	-1.33900
H	-6.37839	-1.72041	-1.71540
H	-7.18469	-1.69611	-0.12980
H	-7.04190	-0.20721	-1.05700
H	3.33530	-0.73128	-1.49610
H	2.98190	0.99892	-1.57510
H	3.02290	1.18182	0.86930
H	3.17180	-0.56308	1.05850
H	7.72470	0.53273	0.45680
H	7.75570	-1.01777	1.30480
H	7.73370	-0.98747	-0.45140
H	4.31011	-2.18608	0.41220
H	5.94981	-2.68397	-0.04290
H	5.57131	-2.37777	1.64450
H	-3.46431	2.79390	-1.90200
H	-3.17760	1.01570	-2.28990
H	-2.84141	2.60450	1.52880
H	-1.29461	3.04610	0.80800
H	-2.79501	3.75960	0.17730
H	6.50939	1.99193	0.11330

Conformer 4

Energy: -1152.65908 Hartree (Rel: 0.2 kcal/mol)

XYZ coordinates for conf 4:

C	1.26442	0.51577	0.16047
O	0.92844	-0.84891	-0.17567
C	-0.41628	-0.94953	-0.41570
C	-1.03330	0.38889	-0.23473
C	-0.05333	1.23818	0.09989
C	2.33176	1.04947	-0.82405
C	3.68387	0.29302	-0.71360
C	2.51988	2.56151	-0.70810
O	4.21053	0.33665	0.62865
C	4.54068	-0.93521	1.03906
C	4.19629	-1.88461	-0.04675
C	3.67685	-1.17119	-1.05349
O	5.02924	-1.14932	2.12566
C	4.42567	-3.35397	0.09853
O	-0.93615	-2.00128	-0.72474
C	-2.50547	0.59550	-0.41117
C	-3.32729	-0.04834	0.73625
C	-4.82140	0.00845	0.48485
C	-5.51810	-0.98282	-0.13773
C	-7.00863	-0.98100	-0.38646
C	-4.85364	-2.23664	-0.65463
H	4.39718	0.82579	-1.35700
H	1.66954	0.50519	1.17920
C	2.33773	3.33308	-1.78618
C	2.92267	3.14665	0.62655
C	-5.47006	1.25449	0.92496
O	-4.86641	2.19093	1.43798
H	-0.15102	2.29851	0.29997
H	1.95778	0.83825	-1.83330
H	3.31228	-1.54670	-2.00271

H	4.13468	-3.89242	-0.80712
H	5.48122	-3.56318	0.30853
H	3.84876	-3.75310	0.94119
H	-2.73304	1.66447	-0.46370
H	-2.81095	0.14533	-1.36401
H	-2.99610	-1.07843	0.88425
H	-3.11071	0.49927	1.65847
H	-7.55656	-0.16389	0.08162
H	-7.20538	-0.94883	-1.46646
H	-7.43791	-1.92419	-0.02416
H	-3.76621	-2.17550	-0.71566
H	-5.11283	-3.08846	-0.00936
H	-5.24104	-2.48012	-1.65196
H	2.47679	4.41046	-1.74159
H	2.04715	2.92068	-2.74945
H	2.18169	2.93034	1.40773
H	3.87082	2.72700	0.97810
H	3.02448	4.23351	0.55652
H	-6.55994	1.34112	0.76811

Conformer 5
Energy: -1152.66009 Hartree (Rel: 0.2 kcal/mol)
XYZ coordinates for conf 5:

C	1.09660	0.20440	-0.83080
O	0.36980	1.31190	-1.40460
C	-0.92800	1.28570	-0.96970
C	-1.10770	0.11030	-0.07870
C	0.07570	-0.51200	0.00390
C	2.36140	0.75890	-0.11660
C	3.18430	-0.38650	0.51240
C	2.05340	1.86690	0.88850
O	3.42980	-1.41010	-0.47570
C	4.78290	-1.60890	-0.60980
C	5.48850	-0.69090	0.32180
C	4.55690	0.01700	0.97280
O	5.24040	-2.42060	-1.38170
C	6.97970	-0.66770	0.41130
O	-1.73800	2.12200	-1.31120
C	-2.44060	-0.21190	0.52100
C	-3.44770	-0.72990	-0.53930
C	-4.84600	-0.90360	0.02020
C	-5.79890	0.06910	-0.01840
C	-7.21080	-0.06880	0.50230
C	-5.54010	1.43230	-0.61440
H	2.61940	-0.85560	1.32690
H	1.43220	-0.43160	-1.65770
C	1.51960	1.62340	2.09140
C	2.38770	3.26510	0.43050
C	-5.09110	-2.20510	0.66290
O	-4.24110	-3.08350	0.76560
H	0.29480	-1.41960	0.55530
H	2.98180	1.18350	-0.91480
H	4.71580	0.77660	1.72900
H	7.32290	0.06080	1.15040
H	7.36530	-1.65620	0.68720
H	7.42130	-0.41610	-0.56020
H	-2.32870	-0.96140	1.31050
H	-2.84540	0.69720	0.98300
H	-3.45200	-0.05150	-1.39510
H	-3.09530	-1.70230	-0.89610
H	-7.49080	-1.07090	0.82550
H	-7.36460	0.61700	1.34620
H	-7.91720	0.24180	-0.27850
H	-4.48630	1.65330	-0.78910

H	-6.07330	1.52760	-1.57130
H	-5.95010	2.21010	0.04220
H	1.30800	2.43400	2.78410
H	1.26280	0.62530	2.43490
H	3.45940	3.36040	0.20780
H	1.84950	3.50500	-0.49470
H	2.12680	4.01060	1.18770
H	-6.09820	-2.38240	1.08030

Conformer 6

Energy: -1152.66014 Hartree (Rel: 0.2 kcal/mol)

XYZ coordinates for conf 6:

C	0.90640	0.05829	0.46340
O	0.43660	-1.30081	0.31800
C	-0.89360	-1.29561	-0.01680
C	-1.35590	0.11399	-0.10100
C	-0.30720	0.89819	0.18050
C	2.09070	0.30559	-0.49600
C	3.17420	-0.79851	-0.33690
C	2.64610	1.72319	-0.37260
O	4.24920	-0.52381	-1.25840
C	5.43970	-0.47791	-0.57950
C	5.17520	-0.74931	0.85730
C	3.85510	-0.92791	0.99460
O	6.49210	-0.25801	-1.13680
C	6.28270	-0.79631	1.85850
O	-1.51350	-2.32171	-0.19730
C	-2.77740	0.44469	-0.43460
C	-3.74820	0.07870	0.71860
C	-5.20300	0.26840	0.33650
C	-5.97250	-0.71420	-0.20950
C	-7.43010	-0.57730	-0.58370
C	-5.43450	-2.09470	-0.50100
H	2.72340	-1.75271	-0.63250
H	1.23000	0.17449	1.50460
C	2.97910	2.26919	0.80300
C	2.80130	2.47119	-1.67380
C	-5.72100	1.62880	0.55650
O	-5.04320	2.55130	0.99690
H	-0.28410	1.98109	0.21130
H	1.69650	0.18009	-1.51150
H	3.32400	-1.16811	1.90900
H	5.90730	-1.03021	2.85810
H	7.02620	-1.55131	1.57700
H	6.80820	0.16519	1.89770
H	-2.87220	1.51039	-0.66390
H	-3.06560	-0.11351	-1.33410
H	-3.55930	-0.94861	1.03760
H	-3.52740	0.73059	1.56910
H	-7.90250	0.35550	-0.27750
H	-7.54550	-0.67680	-1.67130
H	-7.99850	-1.40380	-0.13780
H	-4.34730	-2.17161	-0.45590
H	-5.85891	-2.81770	0.21040
H	-5.76100	-2.41940	-1.49710
H	3.37570	3.27979	0.85810
H	2.88400	1.74389	1.74900
H	3.45660	1.91859	-2.35830
H	1.83330	2.58259	-2.18150
H	3.22330	3.46839	-1.51750
H	-6.77790	1.81690	0.29680

Conformer 7

Energy: -1152.66010 Hartree (Rel: 0.2 kcal/mol)

XYZ coordinates for conf 7:

C	1.19450	-1.40739	0.81000
O	0.69630	-1.95749	-0.42840
C	-0.53080	-1.40950	-0.71660
C	-0.87180	-0.42760	0.34090
C	0.14510	-0.40960	1.21200
C	2.64900	-0.88359	0.66120
C	2.80270	0.14471	-0.48440
C	3.65130	-2.03079	0.54320
O	2.01590	1.32011	-0.19760
C	2.82359	2.42941	-0.18730
C	4.21519	2.00331	-0.48330
C	4.19960	0.67571	-0.65510
O	2.39099	3.53841	0.03420
C	5.33689	2.98851	-0.54630
O	-1.15860	-1.73630	-1.70150
C	-2.15890	0.33680	0.30850
C	-3.38780	-0.57180	0.57240
C	-4.70320	0.16049	0.39170
C	-5.36540	0.23639	-0.79630
C	-6.68440	0.93939	-1.02020
C	-4.83770	-0.40191	-2.05890
H	2.41790	-0.28019	-1.41890
H	1.22680	-2.23039	1.53670
C	4.50110	-2.27139	1.54850
C	3.64560	-2.87699	-0.70970
C	-5.20110	0.83609	1.60150
O	-4.61370	0.82589	2.67820
H	0.23350	0.20130	2.10310
H	2.86550	-0.34769	1.59340
H	5.04580	0.03791	-0.88110
H	6.28559	2.49762	-0.77830
H	5.13959	3.74981	-1.31010
H	5.44049	3.51821	0.40810
H	-2.13460	1.14220	1.04910
H	-2.26490	0.80380	-0.67870
H	-3.33620	-1.44660	-0.07960
H	-3.33240	-0.92990	1.60470
H	-7.17020	1.31379	-0.11980
H	-6.54261	1.78459	-1.70690
H	-7.38220	0.25319	-1.51770
H	-3.80480	-0.74590	-1.99110
H	-5.46780	-1.26021	-2.33320
H	-4.90940	0.30779	-2.89310
H	5.21670	-3.08859	1.50190
H	4.51210	-1.66749	2.45270
H	2.66931	-3.34499	-0.87350
H	3.85430	-2.27889	-1.60680
H	4.40461	-3.66229	-0.64910
H	-6.15500	1.38639	1.51740

Conformer 8

Energy: -1152.65883 Hartree (Rel: 0.3 kcal/mol)

XYZ coordinates for conf 8:

C	-1.32760	0.40741	-0.61420
O	-1.02120	-0.95119	-0.22870
C	0.32950	-1.16289	-0.30860
C	0.97950	0.08740	-0.77730
C	0.01270	0.99761	-0.95390
C	-2.08160	1.12761	0.52890
C	-3.46700	0.50151	0.82930
C	-2.16930	2.63321	0.26160

O	-4.28980	0.44531	-0.35400
C	-4.79080	-0.82669	-0.51030
C	-4.26750	-1.67809	0.58540
C	-3.47990	-0.90949	1.34750
O	-5.53300	-1.11369	-1.42280
C	-4.62240	-3.12599	0.68740
O	0.83000	-2.23210	-0.02850
C	2.46290	0.17110	-0.96060
C	3.22140	0.14640	0.39240
C	4.72580	0.07910	0.21590
C	5.42370	-1.08850	0.14190
C	6.92250	-1.20340	-0.01250
C	4.75140	-2.43850	0.21700
H	-3.97280	1.16931	1.53960
H	-1.97560	0.35911	-1.49730
C	-2.90210	3.14361	-0.73510
C	-1.35790	3.50311	1.19060
C	5.38580	1.38800	0.07960
O	4.78510	2.45730	0.09150
H	0.12790	2.01671	-1.30270
H	-1.48600	0.97641	1.43800
H	-2.92890	-1.20989	2.23150
H	-4.16111	-3.58709	1.56450
H	-5.70911	-3.25569	0.75220
H	-4.29041	-3.66809	-0.20590
H	2.79030	-0.68190	-1.56820
H	2.72410	1.08310	-1.50630
H	2.97920	1.06510	0.93510
H	2.86140	-0.69210	0.99250
H	7.46950	-0.26330	0.05020
H	7.31720	-1.87390	0.76210
H	7.16170	-1.67290	-0.97600
H	3.66620	-2.40620	0.11220
H	5.15240	-3.09870	-0.56250
H	4.98890	-2.91990	1.17670
H	-2.93660	4.21671	-0.90720
H	-3.49930	2.52361	-1.39530
H	-1.68510	3.37701	2.23190
H	-0.29430	3.22901	1.16370
H	-1.44559	4.56211	0.93040
H	6.48280	1.39130	-0.04870

Conformer 9

Energy: -1152.65880 Hartree (Rel: 0.3 kcal/mol)

XYZ coordinates for conf 9:

C	1.62470	-1.66280	0.91160
O	1.12041	-2.27710	-0.29320
C	-0.24419	-2.11800	-0.35400
C	-0.68980	-1.36970	0.84600
C	0.40150	-1.08820	1.56790
C	2.78080	-0.66649	0.60820
C	2.44680	0.29701	-0.55540
C	4.10210	-1.39489	0.37260
O	1.25340	1.04910	-0.25530
C	1.52130	2.39270	-0.31560
C	2.95459	2.57221	-0.66380
C	3.48930	1.35231	-0.80190
O	0.66939	3.22770	-0.10760
C	3.56049	3.93091	-0.80320
O	-0.89659	-2.55800	-1.27660
C	-2.12650	-0.99800	1.03840
C	-2.53990	0.18390	0.12090
C	-4.02990	0.45969	0.16110
C	-4.91760	-0.07541	-0.72270

C	-6.40400	0.19609	-0.74300
C	-4.49310	-1.02101	-1.82180
H	2.23840	-0.28180	-1.46230
H	2.04401	-2.46619	1.53190
C	5.10990	-1.23979	1.23900
C	4.22431	-2.29299	-0.83730
C	-4.46610	1.32169	1.27160
O	-3.70770	1.76289	2.12840
H	0.44700	-0.54050	2.50260
H	2.89290	-0.05219	1.50960
H	4.51670	1.11361	-1.05020
H	4.61939	3.86931	-1.06750
H	3.03919	4.50891	-1.57530
H	3.46489	4.49521	0.13210
H	-2.75219	-1.86851	0.80610
H	-2.31140	-0.72500	2.08160
H	-2.00560	1.07860	0.45490
H	-2.21150	-0.02040	-0.90070
H	-6.74210	0.97929	-0.06520
H	-6.70680	0.47839	-1.75980
H	-6.95420	-0.72371	-0.50310
H	-3.50930	-1.46791	-1.67110
H	-5.22620	-1.83071	-1.92310
H	-4.48360	-0.49221	-2.78610
H	6.05660	-1.75749	1.10590
H	5.02680	-0.59559	2.11110
H	3.44441	-3.06249	-0.84770
H	4.11460	-1.73109	-1.77430
H	5.20041	-2.78609	-0.85680
H	-5.54190	1.56649	1.33060

Conformer 10
Energy: -1152.65894 Hartree (Rel: 0.4 kcal/mol)
XYZ coordinates for conf 10:

C	1.27860	0.51300	0.19739
O	0.94840	-0.83860	-0.19351
C	-0.39450	-0.93390	-0.44451
C	-1.01770	0.39330	-0.20881
C	-0.04160	1.23190	0.16319
C	2.35860	1.08920	-0.74891
C	3.71170	0.34080	-0.64831
C	2.51100	2.60010	-0.54991
O	4.20200	0.31530	0.70799
C	4.52960	-0.97430	1.05869
C	4.22370	-1.86670	-0.08561
C	3.72580	-1.10320	-1.06611
O	4.98840	-1.24400	2.14609
C	4.46240	-3.33990	-0.01321
O	-0.90960	-1.97340	-0.79991
C	-2.49120	0.59940	-0.37381
C	-3.30740	-0.09720	0.74649
C	-4.80240	-0.03620	0.50119
C	-5.49560	-1.00151	-0.16451
C	-6.98640	-0.99491	-0.41181
C	-4.82620	-2.22790	-0.73801
H	4.43880	0.91210	-1.24081
H	1.68010	0.47120	1.21679
C	3.02510	3.13030	0.56609
C	2.02280	3.45380	-1.69491
C	-5.45610	1.18519	0.99909
O	-4.85580	2.10090	1.55199
H	-0.14060	2.27900	0.42289
H	2.00040	0.91470	-1.77131
H	3.39000	-1.42970	-2.04381

H	4.19820	-3.83150	-0.95311
H	5.51450	-3.55210	0.21109
H	3.86910	-3.78790	0.79259
H	-2.72480	1.66840	-0.38031
H	-2.79630	0.18850	-1.34441
H	-2.97120	-1.13120	0.84879
H	-3.09070	0.41080	1.69109
H	-7.53730	-0.20181	0.09259
H	-7.18400	-0.91521	-1.48921
H	-7.41150	-1.95521	-0.09191
H	-3.73920	-2.15910	-0.79761
H	-5.08040	-3.10880	-0.13091
H	-5.21400	-2.42861	-1.74461
H	3.10920	4.20810	0.68219
H	3.39120	2.52070	1.38519
H	2.58150	3.23330	-2.61511
H	0.96600	3.25190	-1.91741
H	2.13000	4.52060	-1.47711
H	-6.54710	1.27249	0.85059

Conformer 11
Energy: -1152.66015 Hartree (Rel: 0.4 kcal/mol)
XYZ coordinates for conf 11:

C	1.17820	-1.44820	0.74311
O	0.66930	-1.95450	-0.50950
C	-0.55610	-1.39200	-0.77109
C	-0.88780	-0.44550	0.32131
C	0.13460	-0.46140	1.18600
C	2.62870	-0.92380	0.59540
C	2.77640	0.14310	-0.51540
C	3.63930	-2.05840	0.40210
O	2.00750	1.31780	-0.18060
C	2.82620	2.41870	-0.15499
C	4.20620	1.98859	-0.49669
C	4.17370	0.66609	-0.70410
O	2.41041	3.52470	0.10980
C	5.33411	2.96609	-0.56600
O	-1.19130	-1.68180	-1.76320
C	-2.17240	0.32370	0.32190
C	-3.40220	-0.58880	0.56841
C	-4.71700	0.14991	0.41060
C	-5.38450	0.25351	-0.77240
C	-6.70350	0.96311	-0.97430
C	-4.86360	-0.35749	-2.05130
H	2.37350	-0.25770	-1.45320
H	1.21300	-2.29670	1.43951
C	3.57470	-2.90940	-0.62870
C	4.72450	-2.14250	1.44761
C	-5.20790	0.79921	1.63750
O	-4.61590	0.76321	2.71101
H	0.23070	0.11880	2.09671
H	2.85700	-0.42060	1.54330
H	5.00180	0.02539	-0.98199
H	6.27191	2.47359	-0.83600
H	5.12281	3.74759	-1.30540
H	5.46811	3.47139	0.39770
H	-2.14000	1.10690	1.08560
H	-2.28440	0.81960	-0.65050
H	-3.35490	-1.44740	-0.10509
H	-3.34330	-0.97230	1.59130
H	-7.18470	1.31761	-0.06349
H	-6.56360	1.82351	-1.64240
H	-7.40440	0.28931	-1.48409
H	-3.82950	-0.70000	-1.99750

H	-5.49350	-1.21169	-2.33889
H	-4.94280	0.36891	-2.87030
H	4.31250	-3.69961	-0.74530
H	2.78660	-2.86370	-1.37279
H	5.29280	-1.20411	1.50640
H	4.29570	-2.30870	2.44541
H	5.42530	-2.95591	1.23690
H	-6.16070	1.35361	1.56991

Conformer 12

Energy: -1152.66003 Hartree (Rel: 0.6 kcal/mol)

XYZ coordinates for conf 12:

C	1.54749	-1.69501	0.92151
O	1.01689	-2.33740	-0.25759
C	-0.34201	-2.14300	-0.31619
C	-0.75831	-1.33580	0.85581
C	0.34579	-1.05770	1.56021
C	2.73149	-0.75711	0.56901
C	2.39039	0.24619	-0.55829
C	4.00669	-1.53441	0.23031
O	1.31129	1.11289	-0.15019
C	1.70609	2.42369	-0.24059
C	3.10779	2.46099	-0.73139
C	3.50209	1.19439	-0.91399
O	0.96869	3.33960	0.04821
C	3.82579	3.75369	-0.94529
O	-1.01331	-2.59960	-1.21719
C	-2.18451	-0.92440	1.04631
C	-2.58521	0.22250	0.08041
C	-4.06891	0.53060	0.12491
C	-4.97911	-0.03260	-0.71749
C	-6.46031	0.26671	-0.73219
C	-4.58871	-1.04350	-1.77009
H	2.04839	-0.31101	-1.43829
H	1.94429	-2.48951	1.56701
C	4.06209	-2.41151	-0.77889
C	5.20019	-1.23611	1.10441
C	-4.47291	1.45800	1.19431
O	-3.69441	1.92800	2.01691
H	0.41149	-0.47660	2.47311
H	2.91999	-0.16421	1.47261
H	4.46409	0.85329	-1.27729
H	4.84199	3.58909	-1.31299
H	3.28840	4.37889	-1.66799
H	3.87910	4.32579	-0.01139
H	-2.83051	-1.79140	0.86151
H	-2.35121	-0.60020	2.07791
H	-2.02921	1.11970	0.36901
H	-2.27401	-0.03500	-0.93449
H	-6.77321	1.09241	-0.09389
H	-6.77291	0.49871	-1.75869
H	-7.02401	-0.62760	-0.43409
H	-3.60981	-1.49840	-1.61159
H	-5.33661	-1.84480	-1.81489
H	-4.58791	-0.56830	-2.76189
H	4.98239	-2.94501	-1.00399
H	3.20099	-2.64131	-1.39799
H	5.45249	-0.16711	1.08121
H	4.98859	-1.48191	2.15431
H	6.08139	-1.80391	0.79131
H	-5.54291	1.72670	1.25421

Conformer 13

Energy: -1152.65945 Hartree (Rel: 0.8 kcal/mol)

XYZ coordinates for conf 13:

C	-1.18720	-0.96411	-0.53301
O	-0.47550	-2.04710	0.09819
C	0.87160	-1.84750	-0.02201
C	1.10030	-0.59030	-0.78121
C	-0.10290	-0.07800	-1.07521
C	-2.18730	-0.36131	0.49739
C	-3.18181	0.58929	-0.20791
C	-1.51650	0.28269	1.70619
O	-3.79510	-0.08771	-1.32511
C	-5.16070	-0.08952	-1.17431
C	-5.49561	0.61238	0.09169
C	-4.34461	1.00818	0.64919
O	-5.89640	-0.59892	-1.98861
C	-6.91071	0.77288	0.54319
O	1.68301	-2.62910	0.42929
C	2.48390	-0.10749	-1.08581
C	3.21800	0.40201	0.18229
C	4.67099	0.74351	-0.08411
C	5.69030	-0.15069	0.04829
C	7.15290	0.14892	-0.18551
C	5.45970	-1.58349	0.46539
H	-2.65511	1.46089	-0.61661
H	-1.77980	-1.39751	-1.34631
C	-1.38650	-0.42091	2.83679
C	-1.00251	1.70199	1.60229
C	4.90199	2.12061	-0.55011
O	4.00529	2.93881	-0.72701
H	-0.30401	0.82140	-1.64681
H	-2.77430	-1.21741	0.84899
H	-4.22191	1.54489	1.58269
H	-6.96711	1.31508	1.49059
H	-7.49611	1.31618	-0.20801
H	-7.38920	-0.20542	0.66989
H	3.05670	-0.93689	-1.51921
H	2.44880	0.69290	-1.83141
H	2.70989	1.30621	0.53059
H	3.13150	-0.34559	0.97369
H	7.38869	1.19632	-0.37111
H	7.73420	-0.17688	0.68699
H	7.52100	-0.43918	-1.03671
H	4.41590	-1.89819	0.43299
H	6.04190	-2.25688	-0.17631
H	5.83270	-1.73788	1.48819
H	-0.89690	-0.00191	3.71249
H	-1.75420	-1.43971	2.92459
H	-0.29161	1.82220	0.77669
H	-1.81211	2.42339	1.42919
H	-0.49501	1.99440	2.52609
H	5.94569	2.41691	-0.75661

Conformer 14

Energy: -1152.66009 Hartree (Rel: 0.8 kcal/mol)

XYZ coordinates for conf 14:

C	-1.59927	-1.78078	-0.52958
O	-1.49648	-2.28451	0.81728
C	-0.18520	-2.22053	1.23534
C	0.63238	-1.64455	0.14015
C	-0.19275	-1.39165	-0.88282
C	-2.65550	-0.64283	-0.64238
C	-2.44729	0.47396	0.40967
C	-4.08218	-1.18668	-0.61282

O	-1.12919	1.04716	0.27749
C	-1.22420	2.39554	0.06447
C	-2.66103	2.77169	0.05191
C	-3.36874	1.65198	0.25206
O	-0.24235	3.09236	-0.07973
C	-3.09900	4.18486	-0.15787
O	0.15047	-2.59046	2.33805
C	2.09122	-1.35502	0.32096
C	2.65947	-0.39497	-0.74304
C	4.09556	0.00160	-0.46269
C	4.46188	1.13162	0.20543
C	5.88588	1.53746	0.51326
C	3.46843	2.13184	0.74549
H	-2.50822	0.04686	1.41717
H	-1.94686	-2.60876	-1.16122
C	-4.84786	-1.09628	-1.70659
C	-4.58620	-1.83173	0.65818
C	5.09334	-0.96676	-0.94983
O	4.80222	-2.00604	-1.53256
H	0.05893	-0.94956	-1.83937
H	-2.49335	-0.18618	-1.62638
H	-4.44756	1.56108	0.29648
H	-4.18852	4.27088	-0.13803
H	-2.68188	4.83773	0.61793
H	-2.73497	4.56445	-1.11990
H	2.22840	-0.93166	1.32455
H	2.66340	-2.29210	0.31348
H	2.62892	-0.89484	-1.71698
H	2.01419	0.48442	-0.81055
H	6.64856	0.80933	0.24035
H	6.11822	2.47971	-0.00077
H	5.98420	1.74120	1.58739
H	2.44139	1.99339	0.40589
H	3.46777	2.08926	1.84397
H	3.78097	3.15024	0.48090
H	-5.86214	-1.48757	-1.72221
H	-4.49165	-0.63383	-2.62399
H	-3.94643	-2.66545	0.96697
H	-4.59647	-1.12442	1.49803
H	-5.60582	-2.20452	0.52443
H	6.15804	-0.73035	-0.77830

Conformer 15

Energy: -1152.65970 Hartree (Rel: 0.9 kcal/mol)

XYZ coordinates for conf 15:

C	-0.85391	-0.01229	-0.88761
O	-1.03060	-1.35459	-0.38721
C	0.14350	-2.05179	-0.50151
C	1.15770	-1.16878	-1.13151
C	0.56890	0.00751	-1.37771
C	-1.13181	1.00290	0.24749
C	-2.59781	0.95500	0.75519
C	-0.71732	2.42451	-0.12891
O	-3.54221	1.19419	-0.31001
C	-4.50421	0.21029	-0.30531
C	-4.18800	-0.75331	0.77579
C	-3.06620	-0.33230	1.37339
O	-5.42431	0.21288	-1.09281
C	-5.04490	-1.95091	1.03009
O	0.24481	-3.20489	-0.14451
C	2.56150	-1.64008	-1.34951
C	3.38510	-1.70777	-0.03271
C	3.70250	-0.35407	0.57799
C	4.77099	0.40933	0.21009

C	5.13719	1.75443	0.79489
C	5.74329	-0.01706	-0.86221
H	-2.70911	1.77880	1.47329
H	-1.57321	0.12450	-1.70341
C	0.12338	3.09771	0.66579
C	-1.28762	3.04380	-1.38471
C	2.75669	0.10762	1.60819
O	1.75470	-0.51278	1.95039
H	1.02069	0.88111	-1.83251
H	-0.51321	0.68821	1.09639
H	-2.54560	-0.80350	2.19889
H	-4.67019	-2.53451	1.87499
H	-6.07860	-1.65172	1.24009
H	-5.07489	-2.59911	0.14619
H	2.52821	-2.64978	-1.77711
H	3.06440	-0.99098	-2.07431
H	2.81581	-2.29208	0.69509
H	4.30560	-2.26277	-0.23461
H	4.47959	2.11613	1.58389
H	6.15659	1.71054	1.20029
H	5.15598	2.51003	-0.00161
H	5.51310	-0.98036	-1.31681
H	5.77339	0.73774	-1.65951
H	6.75980	-0.06516	-0.44871
H	0.42307	4.11821	0.43929
H	0.53458	2.65871	1.57139
H	-1.01312	2.47271	-2.28181
H	-2.38192	3.06640	-1.35711
H	-0.91852	4.06541	-1.51521
H	2.97459	1.07452	2.09319

Conformer 16
Energy: -1152.65988 Hartree (Rel: 0.9 kcal/mol)
XYZ coordinates for conf 16:

C	1.09380	0.06889	-0.73229
O	0.40450	1.10489	-1.45949
C	-0.90670	1.14569	-1.07509
C	-1.13810	0.08499	-0.05989
C	0.03110	-0.54061	0.13571
C	2.33990	0.69699	-0.04039
C	3.27720	-0.40981	0.49511
C	1.99800	1.73969	1.01891
O	3.57740	-1.34691	-0.56039
C	4.93650	-1.41361	-0.74959
C	5.59190	-0.49051	0.21261
C	4.62610	0.08859	0.93661
O	5.43500	-2.13461	-1.58359
C	7.07700	-0.33321	0.25191
O	-1.69260	1.94249	-1.54469
C	-2.49570	-0.14461	0.52741
C	-3.47810	-0.77101	-0.49699
C	-4.89830	-0.84611	0.02861
C	-5.82640	0.13170	-0.16729
C	-7.25760	0.08850	0.31611
C	-5.51640	1.40230	-0.92229
H	2.78310	-0.97881	1.29281
H	1.46000	-0.65511	-1.46879
C	2.02000	3.03749	0.69431
C	1.63180	1.28719	2.41561
C	-5.19560	-2.05001	0.82161
O	-4.37060	-2.92401	1.06651
H	0.21600	-1.38191	0.79471
H	2.88410	1.19799	-0.84899
H	4.75220	0.82069	1.72581

H	7.38130	0.38499	1.01761
H	7.56130	-1.29461	0.45971
H	7.45500	0.00779	-0.71899
H	-2.42370	-0.79551	1.40421
H	-2.89830	0.81919	0.86331
H	-3.43740	-0.20391	-1.42939
H	-3.13670	-1.78691	-0.71799
H	-7.57210	-0.85871	0.75311
H	-7.42260	0.87870	1.06081
H	-7.93030	0.31139	-0.52229
H	-4.45210	1.58329	-1.07859
H	-6.00810	1.38010	-1.90559
H	-5.93720	2.26470	-0.39009
H	1.76390	3.80759	1.41781
H	2.28430	3.37559	-0.30399
H	0.78840	0.58709	2.41191
H	2.46450	0.77569	2.91631
H	1.35320	2.14429	3.03551
H	-6.22050	-2.15390	1.22011

Conformer 17

Energy: -1152.65888 Hartree (Rel: 0.9 kcal/mol)

XYZ coordinates for conf 17:

C	1.69999	-1.65781	0.95189
O	1.36489	-2.39231	-0.24221
C	-0.00371	-2.40740	-0.41041
C	-0.62211	-1.64280	0.70089
C	0.37100	-1.20661	1.48519
C	2.73470	-0.53141	0.66479
C	2.30650	0.39079	-0.50221
C	4.13920	-1.09372	0.45549
O	1.04770	1.03009	-0.19831
C	1.18741	2.39069	-0.26241
C	2.59381	2.70539	-0.61961
C	3.24100	1.54079	-0.75771
O	0.25981	3.14159	-0.04881
C	3.06731	4.11489	-0.76731
O	-0.52101	-2.98030	-1.34251
C	-2.09410	-1.37760	0.77889
C	-2.56390	-0.43100	-0.36101
C	-3.99790	0.02690	-0.18401
C	-4.36200	1.22581	0.35279
C	-5.78690	1.69001	0.56049
C	-3.36870	2.25620	0.83369
H	2.14900	-0.20781	-1.40661
H	2.17679	-2.36391	1.64449
C	5.09330	-0.84472	1.36009
C	4.40779	-1.93572	-0.77111
C	-5.00500	-0.95529	-0.62361
O	-4.72481	-2.05819	-1.07951
H	0.27880	-0.60451	2.38219
H	2.75580	0.08979	1.56849
H	4.28500	1.40118	-1.01211
H	4.12661	4.15268	-1.03401
H	2.49211	4.63609	-1.54161
H	2.92061	4.67259	0.16509
H	-2.65251	-2.31720	0.70199
H	-2.32530	-0.93110	1.75229
H	-1.88080	0.41860	-0.41851
H	-2.48310	-0.98010	-1.30381
H	-6.55610	0.94681	0.35489
H	-5.91360	2.02621	1.59779
H	-5.98409	2.56581	-0.07221
H	-2.34100	2.09490	0.50679

H	-3.67719	3.25610	0.50319
H	-3.37270	2.28420	1.93299
H	6.09850	-1.24352	1.24799
H	4.90690	-0.23922	2.24389
H	3.73679	-2.79991	-0.82011
H	4.24700	-1.36852	-1.69751
H	5.44119	-2.29432	-0.77531
H	-6.06750	-0.66619	-0.53871

Conformer 18
Energy: -1152.65892 Hartree (Rel: 0.9 kcal/mol)
XYZ coordinates for conf 18:

C	-0.88761	0.13291	-0.52820
O	-0.41651	-1.23169	-0.57650
C	0.90759	-1.27709	-0.22220
C	1.36569	0.10401	0.07800
C	0.32089	0.92171	-0.10460
C	-2.08351	0.23911	0.44590
C	-3.17071	-0.82019	0.09590
C	-2.61861	1.66281	0.56100
O	-4.12001	-0.87639	1.17710
C	-5.38761	-0.69379	0.69210
C	-5.30951	-0.52529	-0.78270
C	-4.01611	-0.59729	-1.12510
O	-6.36271	-0.69869	1.41090
C	-6.53601	-0.33069	-1.61300
O	1.52609	-2.31889	-0.18570
C	2.77859	0.38011	0.48850
C	3.77649	0.18571	-0.68330
C	5.22199	0.30291	-0.24110
C	5.97199	-0.75749	0.16970
C	7.41949	-0.69089	0.59840
C	5.42049	-2.16169	0.23510
H	-2.67171	-1.79479	0.05110
H	-1.19641	0.39611	-1.54770
C	-2.90671	2.16011	1.76930
C	-2.81981	2.48471	-0.69340
C	5.75289	1.67601	-0.24140
O	5.09229	2.66031	-0.55660
H	0.30069	1.99621	0.03520
H	-1.70811	-0.06339	1.43000
H	-3.60531	-0.54049	-2.12710
H	-6.28971	-0.24179	-2.67450
H	-7.22781	-1.17109	-1.48260
H	-7.07431	0.57241	-1.30150
H	2.86609	1.40031	0.87450
H	3.04679	-0.30479	1.30260
H	3.59149	-0.77969	-1.15920
H	3.58029	0.96091	-1.43000
H	7.90439	0.27321	0.44880
H	7.50439	-0.95379	1.66130
H	7.99619	-1.44599	0.04850
H	4.33449	-2.22219	0.15180
H	5.86020	-2.77179	-0.56700
H	5.71980	-2.63609	1.17820
H	-3.30641	3.16401	1.88890
H	-2.76301	1.58161	2.67780
H	-1.87401	2.68071	-1.21590
H	-3.47871	1.98261	-1.41120
H	-3.26581	3.45331	-0.44960
H	6.80319	1.81311	0.07120

Conformer 19
Energy: -1152.65969 Hartree (Rel: 1.0 kcal/mol)

XYZ coordinates for conf 19:

C	-0.45323	-0.99263	-0.96221
O	0.12083	-1.85752	0.04281
C	1.48486	-1.89596	-0.10692
C	1.85871	-1.04410	-1.26455
C	0.72878	-0.52314	-1.76068
C	-1.24816	0.14151	-0.27773
C	-2.26950	-0.43965	0.73980
C	-1.87321	1.10391	-1.28610
O	-2.96553	0.65388	1.37271
C	-4.31886	0.47998	1.23906
C	-4.56219	-0.78704	0.50198
C	-3.36709	-1.31504	0.20855
O	-5.11851	1.27102	1.68820
C	-5.94669	-1.27006	0.21675
O	2.19050	-2.55238	0.62804
C	3.29202	-0.85699	-1.67084
C	3.87647	0.52550	-1.27580
C	3.65556	0.92274	0.17439
C	4.32747	0.36628	1.22187
C	4.11821	0.69547	2.68054
C	5.36325	-0.71139	1.01950
H	-1.70310	-0.96269	1.51894
H	-1.12156	-1.61157	-1.57261
C	-2.59986	0.68449	-2.32906
C	-1.61480	2.56677	-1.02166
C	2.63148	1.95829	0.38731
O	1.97172	2.47057	-0.51282
H	0.62630	0.15176	-2.60164
H	-0.52387	0.70370	0.32272
H	-3.17985	-2.25082	-0.30641
H	-5.93417	-2.22772	-0.31029
H	-6.51445	-1.38838	1.14709
H	-6.49107	-0.54085	-0.39501
H	3.87885	-1.65726	-1.20970
H	3.38799	-0.97227	-2.75695
H	4.94526	0.52447	-1.52002
H	3.40731	1.29145	-1.89867
H	3.30461	1.38809	2.89212
H	3.92329	-0.23205	3.23454
H	5.04223	1.11619	3.09889
H	5.80471	-0.70572	0.02041
H	6.16848	-0.61863	1.75697
H	4.89457	-1.69309	1.17363
H	-3.03287	1.39350	-3.03014
H	-2.80299	-0.36437	-2.52737
H	-2.02736	2.85502	-0.04639
H	-0.53724	2.77273	-0.98289
H	-2.06838	3.20057	-1.78994
H	2.46186	2.28994	1.42596

Conformer 20
Energy: -1152.65984 Hartree (Rel: 1.0 kcal/mol)
XYZ coordinates for conf 20:

C	-0.95072	-0.20950	-1.03264
O	-0.45971	-1.51618	-0.66069
C	0.91053	-1.49757	-0.60742
C	1.38030	-0.13220	-0.95715
C	0.29605	0.61343	-1.20636
C	-1.91078	0.31868	0.05802
C	-3.01058	-0.73759	0.37565
C	-2.45669	1.70559	-0.26566
O	-3.70705	-0.32972	1.56811
C	-5.05394	-0.28126	1.32541

C	-5.29539	-0.69166	-0.08279
C	-4.10222	-0.95478	-0.63257
O	-5.85418	0.03735	2.17708
C	-6.67594	-0.76541	-0.64879
O	1.55435	-2.48351	-0.31969
C	2.83860	0.20579	-0.96690
C	3.43839	0.25138	0.46318
C	4.94491	0.42245	0.45771
C	5.82063	-0.62096	0.46647
C	7.32663	-0.49850	0.48788
C	5.36383	-2.06037	0.46578
H	-2.49950	-1.67957	0.60440
H	-1.48092	-0.33216	-1.98536
C	-2.49059	2.63757	0.69375
C	-2.95570	2.00170	-1.66326
C	5.40282	1.82004	0.39383
O	4.64418	2.78176	0.33020
H	0.27376	1.65985	-1.48787
H	-1.32166	0.39223	0.97905
H	-3.91419	-1.30561	-1.64142
H	-6.66345	-1.10059	-1.68927
H	-7.29606	-1.45581	-0.06510
H	-7.16541	0.21465	-0.60218
H	3.36971	-0.55543	-1.55200
H	2.99859	1.17038	-1.45804
H	2.99590	1.10179	0.99058
H	3.15166	-0.65262	1.00473
H	7.70843	0.51348	0.61863
H	7.72924	-1.11704	1.30058
H	7.74582	-0.90350	-0.44289
H	4.31184	-2.19729	0.21182
H	5.96705	-2.64291	-0.24182
H	5.53772	-2.50644	1.45568
H	-2.88837	3.63160	0.50487
H	-2.13155	2.43977	1.70003
H	-2.15462	1.93504	-2.41148
H	-3.73952	1.30208	-1.97587
H	-3.36899	3.01310	-1.71573
H	6.49308	1.99642	0.39380

LIST OF FILES (technical info - delete in the final SI version

kallopterolideA-du8ml-chloroform_10106.log kallopterolideA-du8ml-chloroform_1028.log kallopterolideA-du8ml-chloroform_1019.log kallopterolideA-du8ml-chloroform_1020.log kallopterolideA-du8ml-chloroform_1047.log kallopterolideA-du8ml-chloroform_1017.log kallopterolideA-du8ml-chloroform_1032.log kallopterolideA-du8ml-chloroform_1053.log kallopterolideA-du8ml-chloroform_30.log kallopterolideA-du8ml-chloroform_104.log kallopterolideA-du8ml-chloroform_23.log kallopterolideA-du8ml-chloroform_1033.log kallopterolideA-du8ml-chloroform_1012.log kallopterolideA-du8ml-chloroform_10111.log kallopterolideA-du8ml-chloroform_1027.log kallopterolideA-du8ml-chloroform_1080.log kallopterolideA-du8ml-chloroform_1011.log kallopterolideA-du8ml-chloroform_1068.log kallopterolideA-du8ml-chloroform_1022.log kallopterolideA-du8ml-chloroform_1024.log

DU8ML data for 3-methyl-2-(2-((*S*)-5-((*S*)-2-methyl-1-((*S*)-4-methyl-5-oxo-2,5-dihydrofuran-2-yl)allyl)-2-oxo-2,5-dihydrofuran-3-yl)ethyl)but-2-enal (**6*S*,7*S*,8*S* diastereomer**)

NMR parameters calculated for 6*S*,7*S*,8*S* diastereomer vs experimental data of kallopterolide A (**1**)

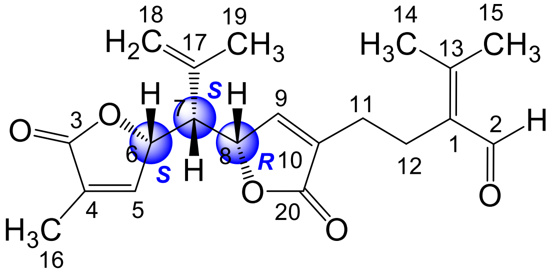

Parameter	RMSD
J_{HH}	0.50 Hz
δ_{1H}	0.20 ppm
δ_{13C}	1.39 ppm

	Conf1	Conf2	Conf3	Conf4	Conf5	Conf6	Conf7	Conf8	Conf9	Conf10	Conf11	Conf12	Conf13	Conf14
Rel energy (kcal/mol):	0.00	0.07	0.80	0.93	0.97	1.07	1.14	1.22	1.24	1.29	1.90	1.97	1.99	1.99

iGau	jGau	Jexp	Jcalc	diff		1	2	3	4	5	6	7	8	9	10	11	12	13	14			
30	31	1.60	1.52	-0.08	[	-1.52	-1.52	-1.53	-1.52	-1.52	-1.54	-1.54	-1.54	-1.54	-1.52	-1.53	-1.52	-1.52	-1.52	]	H5	H16-Me
22	29	10.00	10.88	0.88	[	10.97	10.96	10.75	10.77	10.78	10.94	10.74	10.82	10.99	10.33	10.67	11.00	10.95	10.73	]	H6	H7
22	30	2.00	1.96	-0.04	[	1.96	1.96	1.93	1.95	1.95	1.95	1.99	1.98	1.94	1.97	1.96	1.95	1.96	1.96	]	H6	H5
22	31	1.90	1.85	-0.05	[	1.86	1.87	1.80	1.87	1.87	1.83	1.83	1.83	1.83	1.79	1.83	1.87	1.86	1.87	]	H6	H16-Me
23	29	4.00	4.71	0.71	[	2.40	2.41	9.88	2.75	2.75	10.27	10.54	10.51	10.30	10.11	10.08	2.47	2.33	2.90	]	H8	H7
23	28	1.60	2.04	0.44	[	2.06	2.05	2.05	2.03	2.02	1.96	2.01	2.01	1.97	2.12	2.09	2.11	2.09	2.00	]	H8	H9

For Js: RMSD=0.50Hz N=6 {-0.08 0.88}

NOTICE:
removed Js associated with H11 and H12 b/c they are not available in "B" set

H-nom	iGau	Exp	Calc	diff		1	2	3	4	5	6	7	8	9	10	11	12	13	14	
H2	49	10.10	10.08	-0.02	[	10.09	10.10	9.93	10.09	10.10	10.11	10.10	10.10	10.10	9.96	10.23	9.92	10.12	10.10	]
H5	30	7.23	7.31	0.08	[	7.32	7.31	7.35	7.28	7.26	7.29	7.32	7.33	7.29	7.32	7.37	7.29	7.32	7.26	]
H6	22	5.35	5.18	-0.17	[	5.23	5.22	4.91	5.44	5.42	4.98	5.01	5.00	4.97	4.97	4.97	5.17	5.23	5.40	]
H7	29	2.41	2.10	-0.31	[	2.08	2.07	1.89	2.41	2.39	1.77	2.23	2.32	1.86	2.24	1.76	2.05	2.03	2.33	]
H8	23	5.23	5.33	0.10	[	5.42	5.44	4.96	5.48	5.50	5.10	5.22	5.20	5.08	5.05	5.04	5.27	5.40	5.37	]
H9	28	7.31	7.40	0.09	[	7.25	7.26	7.48	7.30	7.29	7.96	7.93	7.91	7.96	7.49	7.63	7.34	7.17	7.06	]
H11	34	2.27	2.38	0.11	[	2.57	2.16	2.24	2.58	2.20	2.18	2.17	2.62	2.65	2.25	2.55	2.66	2.63	2.56	]
H11	35	2.27	2.42	0.15	[	2.21	2.59	2.62	2.21	2.58	2.64	2.63	2.19	2.18	2.61	2.55	2.36	2.17	2.67	]
H12	36	2.52	2.51	-0.01	[	2.51	2.55	2.30	2.55	2.55	2.58	2.59	2.50	2.51	2.29	2.75	2.17	1.98	2.83	]
H12	37	2.52	2.58	0.06	[	2.50	2.53	2.88	2.54	2.53	2.55	2.55	2.56	2.57	2.87	2.45	3.25	3.34	2.72	]
H14-Me	41	2.04	2.32	0.28	[	2.34	2.34	2.27	2.35	2.35	2.34	2.34	2.33	2.34	2.27	1.78	2.33	2.13	1.97	]

H15-Me	38	2.22	2.30	0.08	[2.27	2.28	2.55	2.27	2.28	2.27	2.27	2.27	2.28	2.56	2.27	2.25	2.27	2.21]
H16-Me	31	1.86	2.04	0.18	[2.04	2.04	2.07	2.04	2.04	2.02	2.03	2.05	2.04	2.06	2.06	2.03	2.03	2.03]
H18a	44	4.89	5.40	0.51	[5.42	5.39	5.51	5.25	5.18	5.55	5.34	5.34	5.55	5.36	5.55	5.35	5.43	5.16]
H18b	45	5.07	5.27	0.20	[5.33	5.30	5.25	5.14	5.07	5.35	5.14	5.16	5.33	5.30	5.31	5.24	5.34	5.04]
H19-Me	46	1.72	1.88	0.16	[1.84	1.76	2.07	1.87	1.84	2.00	2.08	2.08	2.01	2.02	2.01	1.76	1.75	1.81]

1H chem shifts: RMSD=0.20ppm (MAE=0.16) N=16 {-0.31 0.51}

m=1.000 b=0.00

C-nom	iGau	Exp	Calc	diff	1	2	3	4	5	6	7	8	9	10	11	12	13	14
C1-C**	18	135.50	136.64	1.14	[136.75	136.75	136.25	136.75	136.74	136.89	136.88	136.85	136.89	136.32	134.52	134.16	136.94	134.75]
C2-CH	26	190.60	189.78	-0.82	[189.44	189.42	192.74	189.41	189.40	189.29	189.33	189.24	189.26	192.61	190.61	189.12	189.22	189.96]
C3-C	10	173.80	173.13	-0.67	[173.08	173.05	174.41	172.91	172.90	172.74	172.67	172.61	172.68	174.22	173.79	173.00	173.09	172.94]
C4-C	11	130.90	131.72	0.82	[131.82	131.87	131.71	131.53	131.51	131.97	131.17	131.26	131.94	130.85	131.95	131.82	131.77	131.43]
C5-CH	12	146.80	146.15	-0.65	[146.26	146.14	146.98	145.64	145.61	145.73	146.04	145.81	145.59	147.02	146.54	146.15	146.30	145.70]
C6-CH	7	79.80	80.41	0.61	[80.76	80.71	81.14	78.75	78.71	81.04	79.51	79.68	81.20	79.64	80.63	80.79	80.79	78.75]
C7-CH	6	53.50	53.40	-0.10	[52.17	52.14	53.69	55.48	55.51	53.76	56.79	56.36	53.34	57.52	54.25	51.92	52.30	55.55]
C8-CH	1	80.10	81.35	1.25	[79.82	79.93	86.32	81.72	81.81	86.48	81.10	80.96	86.28	80.37	86.55	79.61	79.35	81.52]
C9-CH	5	147.40	145.30	-2.10	[143.89	143.95	145.85	146.76	146.71	147.65	148.38	148.50	147.66	146.61	147.81	146.41	143.86	148.51]
C10-C	4	134.60	135.22	0.62	[136.40	136.28	133.22	134.29	134.20	133.84	133.59	133.59	133.88	132.66	133.27	134.92	137.03	133.31]
C11-CH2	16	24.50	26.96	2.46	[27.20	27.03	26.60	27.09	27.08	27.09	27.10	27.08	27.12	26.19	25.39	22.12	27.31	25.82]
C12-CH2	17	23.40	25.33	1.93	[25.83	25.56	23.48	25.82	25.35	25.28	25.29	25.35	25.37	23.52	25.18	23.80	25.14	24.07]
C13-C**	19	156.80	158.94	2.14	[158.78	158.78	160.74	158.72	158.72	158.46	158.48	158.44	158.40	160.70	158.91	161.58	156.69	158.65]
C14-CH3	21	23.40	21.89	-1.51	[21.70	21.68	23.20	21.70	21.69	21.68	21.68	21.67	21.70	23.20	23.17	22.77	21.70	21.82]
C15-CH3	20	19.40	17.10	-2.30	[17.01	17.09	17.59	17.04	17.06	16.97	16.96	17.05	17.11	17.58	16.88	17.18	16.97	16.87]
C16-CH3	14	10.80	11.11	0.31	[11.15	11.13	11.01	11.16	11.14	11.03	11.05	11.08	11.07	11.02	11.06	11.14	11.11	11.15]
C17-C	8	137.80	139.66	1.86	[137.74	137.65	146.04	139.91	140.17	144.37	140.95	140.88	144.27	140.90	144.96	137.54	137.23	140.11]
C18-CH2	24	117.10	116.06	-1.04	[116.36	116.44	111.67	118.36	118.10	112.16	117.93	118.05	112.15	118.47	112.33	116.23	116.84	118.10]
C19-CH3	25	23.70	23.82	0.12	[25.38	25.30	25.72	19.99	19.96	25.81	17.56	17.64	25.87	17.35	25.72	25.22	25.34	19.83]
C20-C	3	172.90	174.27	1.37	[174.49	174.51	173.21	174.75	174.85	173.93	173.80	173.85	173.99	173.16	173.51	175.09	172.90	174.58]
				Fractions:	0.297	0.262	0.077	0.062	0.058	0.049	0.043	0.038	0.037	0.034	0.012	0.011	0.010	0.010

13C chem shifts: RMSD=1.39ppm (MAE=1.19) N=20 {-2.30 2.46}

NMR parameters calculated for 6*S*,7*S*,8*S* diastereomer vs experimental data of kallopterolide B (**2**)

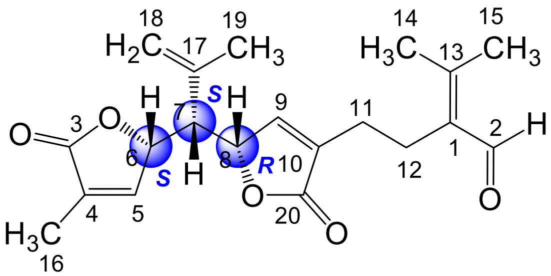

Parameter	RMSD
J_{HH}	2.05 Hz
δ_{1H}	0.25 ppm
δ_{13C}	1.28 ppm

	Conf1	Conf2	Conf3	Conf4	Conf5	Conf6	Conf7	Conf8	Conf9	Conf10	Conf11	Conf12	Conf13	Conf14
Rel energy (kcal/mol):	0.00	0.07	0.80	0.93	0.97	1.07	1.14	1.22	1.24	1.29	1.90	1.97	1.99	1.99

iGau	jGau	Jexp	Jcalc	diff		1	2	3	4	5	6	7	8	9	10	11	12	13	14			
22	30	1.65	1.96	0.31	[	1.96	1.96	1.93	1.95	1.95	1.95	1.99	1.98	1.94	1.97	1.96	1.95	1.96	1.96	]	H6	H5
30	31	1.50	1.52	0.02	[	-1.52	-1.52	-1.53	-1.52	-1.52	-1.54	-1.54	-1.54	-1.54	-1.52	-1.53	-1.52	-1.52	-1.52	]	H5	H16-Me
22	29	7.00	10.88	3.88	[	10.97	10.96	10.75	10.77	10.78	10.94	10.74	10.82	10.99	10.33	10.67	11.00	10.95	10.73	]	H6	H7
22	31	1.70	1.85	0.15	[	1.86	1.87	1.80	1.87	1.87	1.83	1.83	1.83	1.83	1.79	1.83	1.87	1.86	1.87	]	H6	H16-Me
23	29	7.80	4.71	-3.09	[	2.40	2.41	9.88	2.75	2.75	10.27	10.54	10.51	10.30	10.11	10.08	2.47	2.33	2.90	]	H8	H7
23	28	1.40	2.04	0.64	[	2.06	2.05	2.05	2.03	2.02	1.96	2.01	2.01	1.97	2.12	2.09	2.11	2.09	2.00	]	H8	H9

For Js: RMSD=2.05Hz N=6 {-3.09 3.88}

H-nom	iGau	Exp	Calc	diff		1	2	3	4	5	6	7	8	9	10	11	12	13	14	
H2	49	10.10	10.08	-0.02	[	10.09	10.10	9.93	10.09	10.10	10.11	10.10	10.10	10.10	9.96	10.23	9.92	10.12	10.10	]
H5	30	7.10	7.31	0.21	[	7.32	7.31	7.35	7.28	7.26	7.29	7.32	7.33	7.29	7.32	7.37	7.29	7.32	7.26	]
H6	22	5.05	5.18	0.13	[	5.23	5.22	4.91	5.44	5.42	4.98	5.01	5.00	4.97	4.97	4.97	5.17	5.23	5.40	]
H7	29	2.61	2.10	-0.51	[	2.08	2.07	1.89	2.41	2.39	1.77	2.23	2.32	1.86	2.24	1.76	2.05	2.03	2.33	]
H8	23	5.07	5.33	0.26	[	5.42	5.44	4.96	5.48	5.50	5.10	5.22	5.20	5.08	5.05	5.04	5.27	5.40	5.37	]
H9	28	7.13	7.40	0.27	[	7.25	7.26	7.48	7.30	7.29	7.96	7.93	7.91	7.96	7.49	7.63	7.34	7.17	7.06	]
H11	34	2.28	2.38	0.10	[	2.57	2.16	2.24	2.58	2.20	2.18	2.17	2.62	2.65	2.25	2.55	2.66	2.63	2.56	]
H11	35	2.28	2.42	0.14	[	2.21	2.59	2.62	2.21	2.58	2.64	2.63	2.19	2.18	2.61	2.55	2.36	2.17	2.67	]
H12	36	2.52	2.51	-0.01	[	2.51	2.55	2.30	2.55	2.55	2.58	2.59	2.50	2.51	2.29	2.75	2.17	1.98	2.83	]
H12	37	2.52	2.58	0.06	[	2.50	2.53	2.88	2.54	2.53	2.55	2.55	2.56	2.57	2.87	2.45	3.25	3.34	2.72	]
H14-Me	41	2.03	2.32	0.29	[	2.34	2.34	2.27	2.35	2.35	2.34	2.34	2.33	2.34	2.27	1.78	2.33	2.13	1.97	]
H15-Me	38	2.21	2.30	0.09	[	2.27	2.28	2.55	2.27	2.28	2.27	2.27	2.27	2.28	2.56	2.27	2.25	2.27	2.21	]
H16-Me	31	1.95	2.04	0.09	[	2.04	2.04	2.07	2.04	2.04	2.02	2.03	2.05	2.04	2.06	2.06	2.03	2.03	2.03	]
H18a	44	4.83	5.40	0.57	[	5.42	5.39	5.51	5.25	5.18	5.55	5.34	5.34	5.55	5.36	5.55	5.35	5.43	5.16	]
H18b	45	5.08	5.27	0.19	[	5.33	5.30	5.25	5.14	5.07	5.35	5.14	5.16	5.33	5.30	5.31	5.24	5.34	5.04	]

H19-Me	46	1.81	1.88	0.07	[	1.84	1.76	2.07	1.87	1.84	2.00	2.08	2.08	2.01	2.02	2.01	1.76	1.75	1.81	]

1H chem shifts: RMSD=0.25ppm (MAE=0.19) N=16 {-0.51 0.57}

m=1.000 b=0.00

**C-nom**	**iGau**	**Exp**	**Calc**	**diff**		**1**	**2**	**3**	**4**	**5**	**6**	**7**	**8**	**9**	**10**	**11**	**12**	**13**	**14**	
C1-C**	18	135.40	136.64	1.24	[	136.75	136.75	136.25	136.75	136.74	136.89	136.88	136.85	136.89	136.32	134.52	134.16	136.94	134.75	]
C2-CH	26	190.70	189.78	-0.92	[	189.44	189.42	192.74	189.41	189.40	189.29	189.33	189.24	189.26	192.61	190.61	189.12	189.22	189.96	]
C3-C	10	173.50	173.13	-0.37	[	173.08	173.05	174.41	172.91	172.90	172.74	172.67	172.61	172.68	174.22	173.79	173.00	173.09	172.94	]
C4-C	11	130.90	131.72	0.82	[	131.82	131.87	131.71	131.53	131.51	131.97	131.17	131.26	131.94	130.85	131.95	131.82	131.77	131.43	]
C5-CH	12	147.00	146.15	-0.85	[	146.26	146.14	146.98	145.64	145.61	145.73	146.04	145.81	145.59	147.02	146.54	146.15	146.30	145.70	]
C6-CH	7	80.00	80.41	0.41	[	80.76	80.71	81.14	78.75	78.71	81.04	79.51	79.68	81.20	79.64	80.63	80.79	80.79	78.75	]
C7-CH	6	52.80	53.40	0.60	[	52.17	52.14	53.69	55.48	55.51	53.76	56.79	56.36	53.34	57.52	54.25	51.92	52.30	55.55	]
C8-CH	1	79.90	81.35	1.45	[	79.82	79.93	86.32	81.72	81.81	86.48	81.10	80.96	86.28	80.37	86.55	79.61	79.35	81.52	]
C9-CH	5	146.80	145.30	-1.50	[	143.89	143.95	145.85	146.76	146.71	147.65	148.38	148.50	147.66	146.61	147.81	146.41	143.86	148.51	]
C10-C	4	134.60	135.22	0.62	[	136.40	136.28	133.22	134.29	134.20	133.84	133.59	133.59	133.88	132.66	133.27	134.92	137.03	133.31	]
C11-CH2	16	24.60	26.96	2.36	[	27.20	27.03	26.60	27.09	27.08	27.09	27.10	27.08	27.12	26.19	25.39	22.12	27.31	25.82	]
C12-CH2	17	23.40	25.33	1.93	[	25.83	25.56	23.48	25.82	25.35	25.28	25.29	25.35	25.37	23.52	25.18	23.80	25.14	24.07	]
C13-C**	19	156.90	158.94	2.04	[	158.78	158.78	160.74	158.72	158.72	158.46	158.48	158.44	158.40	160.70	158.91	161.58	156.69	158.65	]
C14-CH3	21	23.40	21.89	-1.51	[	21.70	21.68	23.20	21.70	21.69	21.68	21.68	21.67	21.70	23.20	23.17	22.77	21.70	21.82	]
C15-CH3	20	19.40	17.10	-2.30	[	17.01	17.09	17.59	17.04	17.06	16.97	16.96	17.05	17.11	17.58	16.88	17.18	16.97	16.87	]
C16-CH3	14	10.70	11.11	0.41	[	11.15	11.13	11.01	11.16	11.14	11.03	11.05	11.08	11.07	11.02	11.06	11.14	11.11	11.15	]
C17-C	8	139.20	139.66	0.46	[	137.74	137.65	146.04	139.91	140.17	144.37	140.95	140.88	144.27	140.90	144.96	137.54	137.23	140.11	]
C18-CH2	24	116.80	116.06	-0.74	[	116.36	116.44	111.67	118.36	118.10	112.16	117.93	118.05	112.15	118.47	112.33	116.23	116.84	118.10	]
C19-CH3	25	23.60	23.82	0.22	[	25.38	25.30	25.72	19.99	19.96	25.81	17.56	17.64	25.87	17.35	25.72	25.22	25.34	19.83	]
C20-C	3	173.00	174.27	1.27	[	174.49	174.51	173.21	174.75	174.85	173.93	173.80	173.85	173.99	173.16	173.51	175.09	172.90	174.58	]

13C chem shifts: RMSD=1.28ppm (MAE=1.10) N=20 {-2.30 2.36}

Fractions: 0.297 0.262 0.077 0.062 0.058 0.049 0.043 0.038 0.037 0.034 0.012 0.011 0.010 0.010

Conformer 1

Energy: -1152.66040 Hartree (Rel: 0.0 kcal/mol)

XYZ coordinates for conf 1:

C	1.16200	-0.62670	-0.81520
O	0.65840	-1.60890	0.11120
C	-0.71140	-1.58660	0.10630
C	-1.16670	-0.56950	-0.87680
C	-0.07120	-0.01690	-1.41430
C	2.10780	0.37230	-0.09800
C	3.35380	-0.36400	0.44799
C	1.41680	1.20630	0.97740
O	4.01100	-1.06020	-0.63280
C	5.31070	-0.63110	-0.73840
C	5.55770	0.38850	0.31339
C	4.41880	0.53990	1.00140
O	6.06590	-1.06550	-1.57910
C	6.89030	1.04790	0.46220
O	-1.36920	-2.32380	0.81000
C	-2.62260	-0.31280	-1.11041
C	-3.29040	0.40030	0.09470
C	-4.79290	0.52710	-0.06391
C	-5.67860	-0.40500	0.38599
C	-7.18240	-0.31030	0.27120
C	-5.23710	-1.67200	1.07889
H	3.04400	-1.12020	1.17799
H	1.75100	-1.16420	-1.56730
C	1.04300	0.69200	2.15570
C	1.18390	2.65630	0.62940
C	-5.23290	1.72680	-0.79490
O	-4.46300	2.56190	-1.25811
H	-0.03220	0.75380	-2.17631
H	2.47050	1.04430	-0.88660
H	4.24090	1.20090	1.84129
H	6.88800	1.77000	1.28289
H	7.67090	0.30220	0.65360
H	7.17020	1.56780	-0.46160
H	-2.75860	0.29360	-2.01120
H	-3.12580	-1.27290	-1.27971
H	-3.03840	-0.13240	1.01409
H	-2.86560	1.40540	0.17350
H	-7.56060	0.63900	-0.10691
H	-7.55650	-1.11040	-0.38150
H	-7.63310	-0.48260	1.25720
H	-4.17060	-1.88360	0.99099
H	-5.48940	-1.61910	2.14779
H	-5.79180	-2.52930	0.67680
H	0.55300	1.31100	2.90320
H	1.19580	-0.34990	2.41819
H	2.13310	3.16820	0.42019
H	0.57060	2.75230	-0.27660
H	0.67760	3.18860	1.44009
H	-6.31970	1.86430	-0.93510

Conformer 2

Energy: -1152.66094 Hartree (Rel: 0.1 kcal/mol)

XYZ coordinates for conf 2:

C	1.06039	0.51620	-0.22970
O	0.56769	-0.38120	-1.24369
C	-0.76151	-0.63350	-1.02910
C	-1.20571	0.13800	0.16090
C	-0.14170	0.81070	0.61901
C	2.26169	-0.11441	0.52331
C	3.44339	-0.34121	-0.44899
C	1.90319	-1.38041	1.29690

O	3.78979	0.90939	-1.08200
C	5.10750	1.20479	-0.83539
C	5.68919	0.11369	-0.01170
C	4.71929	-0.78271	0.21021
O	5.63210	2.21059	-1.25829
C	7.11890	0.14119	0.42161
O	-1.40001	-1.37280	-1.74829
C	-2.62730	0.09370	0.62731
C	-3.58351	0.83970	-0.34020
C	-5.04350	0.65270	0.02351
C	-5.82371	-0.34250	-0.48319
C	-7.28721	-0.55180	-0.17000
C	-5.29321	-1.37180	-1.45260
H	3.13799	-1.03121	-1.24360
H	1.42109	1.41579	-0.74160
C	1.65919	-2.54881	0.69030
C	1.84379	-1.23351	2.79780
C	-5.55530	1.61020	1.01730
O	-4.86850	2.48390	1.53651
H	-0.10680	1.48160	1.47050
H	2.59320	0.65209	1.23581
H	4.79339	-1.70581	0.77250
H	7.37149	-0.73991	1.01730
H	7.78519	0.17889	-0.44820
H	7.32590	1.03819	1.01720
H	-2.93951	-0.95570	0.69841
H	-2.71050	0.53040	1.62721
H	-3.34960	1.90760	-0.29329
H	-3.39201	0.50930	-1.36329
H	-7.75270	0.23520	0.42230
H	-7.84941	-0.64310	-1.10840
H	-7.42101	-1.50330	0.36190
H	-4.20501	-1.40700	-1.51869
H	-5.65321	-2.36950	-1.17139
H	-5.69101	-1.17540	-2.45879
H	1.40639	-3.43420	1.26841
H	1.68919	-2.67001	-0.38770
H	2.80999	-0.89901	3.19950
H	1.10419	-0.47691	3.09231
H	1.57719	-2.17660	3.28401
H	-6.61640	1.51820	1.30991

Conformer 3
Energy: -1152.66222 Hartree (Rel: 0.8 kcal/mol)
XYZ coordinates for conf 3:

C	0.95229	-1.66895	-1.01540
O	0.75627	-2.86314	-0.22741
C	-0.58016	-3.03871	0.02162
C	-1.33457	-1.96088	-0.66426
C	-0.44279	-1.17456	-1.28118
C	1.88682	-0.73058	-0.20504
C	2.17210	0.60573	-0.93022
C	3.19272	-1.45595	0.13431
O	0.95842	1.38354	-1.04684
C	1.12207	2.59482	-0.42820
C	2.50508	2.67567	0.10092
C	3.11229	1.51859	-0.19148
O	0.22826	3.41268	-0.36399
C	2.99750	3.88877	0.82097
O	-0.99407	-3.96433	0.68507
C	-2.82462	-1.86183	-0.54748
C	-3.27440	-1.06729	0.71125
C	-2.90918	0.40409	0.66089
C	-3.63701	1.34844	-0.00014

C	-3.26867	2.81646	-0.09043
C	-4.92500	1.02853	-0.71506
H	2.52883	0.41359	-1.95037
H	1.45867	-1.96973	-1.94154
C	4.08447	-1.78500	-0.80749
C	3.39676	-1.78154	1.59093
C	-1.68133	0.78118	1.38054
O	-0.98336	-0.00732	2.01248
H	-0.64596	-0.29336	-1.87484
H	1.35843	-0.48171	0.72210
H	4.12857	1.23173	0.04847
H	4.03706	3.76879	1.13657
H	2.92543	4.77532	0.18005
H	2.38316	4.08730	1.70703
H	-3.23967	-1.39227	-1.44585
H	-3.23770	-2.87507	-0.48098
H	-4.35426	-1.19931	0.82976
H	-2.79956	-1.51182	1.59039
H	-3.71500	3.37513	0.74396
H	-2.19466	3.00934	-0.09713
H	-3.68645	3.24302	-1.00905
H	-5.29313	0.01612	-0.54464
H	-5.70570	1.73807	-0.40981
H	-4.79426	1.16718	-1.79740
H	5.00503	-2.30427	-0.55407
H	3.93499	-1.56803	-1.86285
H	3.38518	-0.87072	2.20487
H	2.57980	-2.41745	1.95426
H	4.34491	-2.30082	1.76035
H	-1.39406	1.84560	1.34579

Conformer 4
Energy: -1152.66017 Hartree (Rel: 0.9 kcal/mol)
XYZ coordinates for conf 4:

C	1.14830	-0.56652	-0.77479
O	0.64860	-1.52622	0.18251
C	-0.72180	-1.54202	0.14331
C	-1.18160	-0.54102	-0.85319
C	-0.08940	0.03408	-1.37349
C	2.13080	0.43878	-0.12219
C	3.36140	-0.31421	0.44061
C	1.50300	1.37658	0.90521
O	3.97600	-1.08001	-0.61779
C	5.28840	-0.70381	-0.76019
C	5.58730	0.35359	0.23971
C	4.46540	0.57699	0.93571
O	6.01500	-1.20311	-1.58969
C	6.94360	0.97279	0.33711
O	-1.37530	-2.28372	0.84671
C	-2.63890	-0.30812	-1.10429
C	-3.32960	0.40688	0.08651
C	-4.83210	0.51108	-0.08809
C	-5.70940	-0.42742	0.36491
C	-7.21330	-0.35402	0.23581
C	-5.25760	-1.68002	1.07701
H	3.04040	-1.02941	1.20671
H	1.71190	-1.13322	-1.52559
C	1.33100	2.66798	0.59831
C	1.10880	0.83448	2.26151
C	-5.28120	1.69588	-0.83769
O	-4.51810	2.53578	-1.30329
H	-0.05510	0.80958	-2.13059
H	2.49990	1.05129	-0.95439
H	4.32740	1.28959	1.74041

H	6.98070	1.72849	1.12601
H	7.70200	0.20929	0.54651
H	7.22310	1.44349	-0.61289
H	-2.77400	0.28798	-2.01199
H	-3.12600	-1.27722	-1.26999
H	-3.08050	-0.11372	1.01381
H	-2.91910	1.41838	0.15961
H	-7.60040	0.58538	-0.15749
H	-7.57120	-1.16712	-0.40979
H	-7.67030	-0.51972	1.22011
H	-4.18840	-1.88072	0.99751
H	-5.51620	-1.61582	2.14381
H	-5.80030	-2.54892	0.68341
H	0.88440	3.36378	1.30431
H	1.62240	3.07639	-0.36639
H	0.37040	0.03008	2.18741
H	1.96960	0.41239	2.79681
H	0.68910	1.62948	2.88471
H	-6.36840	1.81678	-0.99019

Conformer 5

Energy: -1152.65904 Hartree (Rel: 1.0 kcal/mol)

XYZ coordinates for conf 5:

C	-1.04991	-0.46880	-0.14750
O	-0.56611	0.39560	-1.19890
C	0.77539	0.62000	-1.02710
C	1.22429	-0.08960	0.19830
C	0.15479	-0.70950	0.71410
C	-2.27901	0.13720	0.57590
C	-3.44741	0.31631	-0.42470
C	-1.99631	1.40861	1.37150
O	-3.74051	-0.95279	-1.04690
C	-5.05311	-1.28659	-0.82360
C	-5.68571	-0.20519	-0.02500
C	-4.74971	0.72451	0.20430
O	-5.53701	-2.31309	-1.24500
C	-7.12111	-0.27539	0.38340
O	1.41369	1.31010	-1.79430
C	2.65219	-0.03960	0.64550
C	3.58629	-0.85120	-0.29000
C	5.05249	-0.67310	0.05260
C	5.84369	0.28739	-0.50180
C	7.31299	0.48649	-0.20990
C	5.32079	1.28390	-1.50870
H	-3.14831	1.00291	-1.22510
H	-1.37961	-1.39770	-0.62810
C	-1.99811	1.36101	2.70910
C	-1.74591	2.70571	0.63420
C	5.55859	-1.59730	1.08060
O	4.86349	-2.43840	1.64080
H	0.11919	-1.31730	1.61140
H	-2.59771	-0.63779	1.28440
H	-4.86791	1.64651	0.76160
H	-7.41461	0.60491	0.96120
H	-7.76981	-0.34919	-0.49740
H	-7.30791	-1.17019	0.98900
H	2.98019	1.00740	0.65610
H	2.74019	-0.42200	1.66700
H	3.33389	-1.91090	-0.18700
H	3.39189	-0.56980	-1.32720
H	7.77069	-0.28001	0.41450
H	7.86879	0.52489	-1.15590
H	7.46689	1.45899	0.27640
H	4.23289	1.32510	-1.57610

H	5.68849	2.28859	-1.26460
H	5.71699	1.04750	-2.50690
H	-1.79981	2.24641	3.30810
H	-2.19241	0.44031	3.25400
H	-0.87880	2.64390	-0.03080
H	-2.59850	2.98471	0.00110
H	-1.57820	3.52260	1.34200
H	6.62359	-1.51021	1.36010

Conformer 6
Energy: -1152.66074 Hartree (Rel: 1.1 kcal/mol)
XYZ coordinates for conf 6:

C	1.09620	-0.97460	-0.90689
O	0.37950	-2.12840	-0.41189
C	-0.85600	-1.74810	0.03951
C	-1.00430	-0.28530	-0.15889
C	0.13200	0.16310	-0.70939
C	2.44980	-0.89120	-0.15329
C	3.35240	0.24640	-0.68589
C	3.18340	-2.23490	-0.22099
O	2.70930	1.52880	-0.53059
C	3.50290	2.36240	0.22001
C	4.73980	1.62740	0.58751
C	4.65040	0.40030	0.05931
O	3.17210	3.49520	0.48701
C	5.81290	2.26840	1.40591
O	-1.63970	-2.54650	0.51021
C	-2.26620	0.42710	0.21861
C	-3.44890	0.05790	-0.71469
C	-4.76700	0.63960	-0.24229
C	-5.61910	-0.01160	0.59771
C	-6.94990	0.51930	1.07851
C	-5.31860	-1.38450	1.15021
H	3.52830	0.10580	-1.75999
H	1.29220	-1.15010	-1.97279
C	3.71610	-2.69400	-1.35909
C	3.25140	-3.00750	1.07091
C	-5.04490	2.00230	-0.72599
O	-4.28190	2.63780	-1.44559
H	0.36990	1.18150	-0.98539
H	2.22380	-0.66010	0.89421
H	5.37860	-0.39870	0.12641
H	6.64880	1.58420	1.57271
H	6.19070	3.16980	0.90921
H	5.41950	2.58530	2.37901
H	-2.52780	0.15530	1.24911
H	-2.10860	1.50970	0.19481
H	-3.23350	0.45520	-1.71109
H	-3.51260	-1.02880	-0.80379
H	-7.27350	1.45050	0.61471
H	-7.72790	-0.23380	0.89811
H	-6.91760	0.67350	2.16541
H	-4.28720	-1.71000	1.00841
H	-5.54310	-1.41150	2.22411
H	-5.98000	-2.12730	0.68121
H	4.22680	-3.65270	-1.39329
H	3.65720	-2.15230	-2.30019
H	3.75410	-2.42490	1.85501
H	2.24010	-3.22630	1.43641
H	3.78720	-3.95340	0.94741
H	-5.99400	2.46890	-0.40729

Conformer 7
Energy: -1152.66052 Hartree (Rel: 1.1 kcal/mol)
XYZ coordinates for conf 7:

C	1.10020	-0.94800	-0.82140
O	0.35890	-2.12090	-0.42050
C	-0.88700	-1.75700	0.01500
C	-1.01430	-0.28240	-0.09160
C	0.14170	0.18660	-0.58020
C	2.43480	-0.90219	-0.02720
C	3.39990	0.15571	-0.62250
C	3.11231	-2.27029	0.01670
O	2.78730	1.46261	-0.62630
C	3.57430	2.34911	0.06870
C	4.77320	1.62531	0.56210
C	4.66820	0.35221	0.16110
O	3.26509	3.51091	0.20520
C	5.83029	2.31962	1.35760
O	-1.69399	-2.57510	0.40520
C	-2.27960	0.41890	0.29510
C	-3.43850	0.12180	-0.69240
C	-4.76470	0.68679	-0.22230
C	-5.64580	-0.00551	0.55240
C	-6.98630	0.50789	1.02500
C	-5.37150	-1.41131	1.03080
H	3.61750	-0.09289	-1.66950
H	1.31630	-1.05669	-1.89270
C	3.47411	-2.79719	1.19130
C	3.35811	-2.99189	-1.28840
C	-5.01770	2.07869	-0.63030
O	-4.22951	2.74879	-1.28890
H	0.40050	1.21640	-0.78620
H	2.20570	-0.59389	0.99890
H	5.36490	-0.45489	0.35110
H	6.63450	1.63332	1.63490
H	6.25979	3.15112	0.78630
H	5.40559	2.75031	2.27190
H	-2.57290	0.08610	1.29880
H	-2.11150	1.49920	0.34340
H	-3.19100	0.57890	-1.65510
H	-3.50920	-0.95630	-0.85170
H	-7.28910	1.46779	0.60810
H	-7.76400	-0.22611	0.77710
H	-6.98560	0.59699	2.11960
H	-4.34050	-1.73951	0.89320
H	-5.62040	-1.49741	2.09630
H	-6.02970	-2.11841	0.50550
H	3.96961	-3.76309	1.25090
H	3.28111	-2.29019	2.13340
H	2.41251	-3.25479	-1.77820
H	3.93031	-2.38429	-2.00180
H	3.91421	-3.91829	-1.11880
H	-5.97121	2.53499	-0.30980

Conformer 8
Energy: -1152.66025 Hartree (Rel: 1.2 kcal/mol)
XYZ coordinates for conf 8:

C	1.19779	-1.25720	-0.94720
O	0.39980	-2.36190	-0.46940
C	-0.92031	-2.00180	-0.43500
C	-1.04140	-0.59930	-0.90509
C	0.19109	-0.16900	-1.20660
C	2.29679	-0.93220	0.10321
C	3.38579	-0.01190	-0.50599
C	2.93609	-2.19810	0.67010

O	2.79949	1.19130	-1.04590
C	3.38159	2.29400	-0.46799
C	4.40379	1.82950	0.50411
C	4.40229	0.49080	0.48111
O	3.05550	3.42060	-0.76450
C	5.21640	2.79680	1.30191
O	-1.78591	-2.77250	-0.07509
C	-2.37120	0.08580	-0.97069
C	-2.94591	0.38830	0.43811
C	-4.36550	0.91900	0.38781
C	-5.46920	0.12190	0.43421
C	-6.90050	0.60650	0.41051
C	-5.37770	-1.38250	0.52700
H	3.87220	-0.52690	-1.34479
H	1.67030	-1.58620	-1.88250
C	2.99200	-2.38030	1.99381
C	3.49790	-3.21460	-0.29709
C	-4.46981	2.37990	0.23600
O	-3.50050	3.12560	0.14561
H	0.47619	0.80370	-1.58449
H	1.82459	-0.38340	0.92551
H	5.02029	-0.17650	1.06930
H	5.91850	2.27870	1.96020
H	5.78189	3.46350	0.64031
H	4.56740	3.43440	1.91361
H	-2.28631	1.01880	-1.53639
H	-3.07310	-0.56360	-1.50910
H	-2.88960	-0.51340	1.05181
H	-2.31150	1.14480	0.90960
H	-7.02600	1.68750	0.46160
H	-7.39921	0.24830	-0.50000
H	-7.44710	0.16470	1.25401
H	-4.38161	-1.78460	0.33810
H	-5.69831	-1.71410	1.52510
H	-6.07461	-1.84260	-0.18529
H	3.45320	-3.26530	2.42521
H	2.57300	-1.66020	2.69231
H	2.70540	-3.64670	-0.92009
H	4.23959	-2.77820	-0.97879
H	3.98270	-4.03360	0.24191
H	-5.48491	2.81330	0.19381

Conformer 9

Energy: -1152.66067 Hartree (Rel: 1.2 kcal/mol)

XYZ coordinates for conf 9:

C	1.20910	-1.31060	-1.02010
O	0.43429	-2.38880	-0.44840
C	-0.88571	-2.03090	-0.38960
C	-1.03180	-0.66200	-0.94320
C	0.18780	-0.24390	-1.30830
C	2.34310	-0.94941	-0.02430
C	3.32860	0.08969	-0.60820
C	3.09539	-2.21051	0.41430
O	2.64620	1.31289	-0.95400
C	3.21841	2.37149	-0.29000
C	4.33570	1.85519	0.54100
C	4.39810	0.53109	0.35370
O	2.81791	3.50529	-0.42300
C	5.16321	2.76698	1.38720
O	-1.73301	-2.77929	0.05220
C	-2.36910	0.00851	-1.01250
C	-2.90730	0.39391	0.39060
C	-4.33670	0.89851	0.34910
C	-5.42440	0.09052	0.48920

C	-6.86390	0.55102	0.47940
C	-5.30421	-1.40178	0.68650
H	3.76930	-0.30151	-1.53400
H	1.65709	-1.69371	-1.94650
C	3.89779	-2.88341	-0.41850
C	2.86009	-2.64901	1.83650
C	-4.47089	2.34281	0.09620
O	-3.51789	3.09391	-0.08150
H	0.45060	0.70720	-1.75160
H	1.87150	-0.49201	0.85320
H	5.09860	-0.16382	0.80060
H	5.93871	2.21588	1.92530
H	5.64281	3.53638	0.77080
H	4.53571	3.29229	2.11680
H	-2.30860	0.90501	-1.63700
H	-3.07970	-0.67849	-1.48930
H	-2.81820	-0.46499	1.05940
H	-2.27220	1.18891	0.79260
H	-7.00620	1.63102	0.46410
H	-7.38420	0.12642	-0.38960
H	-7.37660	0.15722	1.36680
H	-4.31111	-1.80359	0.48130
H	-5.57331	-1.66318	1.72010
H	-6.02511	-1.92208	0.04300
H	4.41799	-3.78051	-0.09330
H	4.06059	-2.58741	-1.45210
H	3.14099	-1.85901	2.54620
H	1.79519	-2.85811	1.99860
H	3.43059	-3.55021	2.08030
H	-5.49409	2.75712	0.05780

Conformer 10
Energy: -1152.65919 Hartree (Rel: 1.3 kcal/mol)
XYZ coordinates for conf 10:

C	0.92535	-1.67045	-0.95057
O	0.72264	-2.88389	-0.19885
C	-0.61518	-3.06168	0.04149
C	-1.36348	-1.96126	-0.61479
C	-0.46681	-1.16400	-1.21003
C	1.85198	-0.74059	-0.11824
C	2.17550	0.57006	-0.88249
C	3.12990	-1.46522	0.29722
O	0.97720	1.36755	-1.02643
C	1.16277	2.59308	-0.44316
C	2.54685	2.66378	0.08468
C	3.13155	1.48642	-0.16991
O	0.28357	3.42805	-0.40416
C	3.06169	3.88842	0.76863
O	-1.03509	-4.00519	0.67521
C	-2.85301	-1.85333	-0.49551
C	-3.29606	-1.02326	0.74251
C	-2.91589	0.44291	0.65623
C	-3.62340	1.37402	-0.04440
C	-3.23915	2.83478	-0.17483
C	-4.90427	1.04473	-0.76796
H	2.52848	0.34311	-1.89726
H	1.42693	-1.94770	-1.88709
C	3.45218	-1.55753	1.59160
C	4.00214	-2.06203	-0.78354
C	-1.69766	0.82995	1.38735
O	-1.02284	0.05569	2.06046
H	-0.66328	-0.26696	-1.78133
H	1.30081	-0.45442	0.78407
H	4.13986	1.18870	0.08966

H	4.09781	3.75741	1.09098
H	3.00892	4.75608	0.10051
H	2.44935	4.12622	1.64633
H	-3.26954	-1.40609	-1.40460
H	-3.26907	-2.86301	-0.40173
H	-4.37740	-1.14173	0.86340
H	-2.82578	-1.44936	1.63319
H	-3.70345	3.42553	0.62702
H	-2.16389	3.01993	-0.15898
H	-3.62837	3.23235	-1.11874
H	-5.28793	0.04378	-0.56661
H	-5.68025	1.77434	-0.50046
H	-4.75568	1.14242	-1.85248
H	4.36086	-2.05951	1.91515
H	2.81745	-1.14648	2.37222
H	3.48573	-2.87292	-1.31296
H	4.29235	-1.32269	-1.54211
H	4.91854	-2.47847	-0.35569
H	-1.39625	1.88912	1.32199

Conformer 11

Energy: -1152.65908 Hartree (Rel: 1.9 kcal/mol)

XYZ coordinates for conf 11:

C	0.73424	-1.77111	-0.89985
O	0.28825	-2.84055	-0.03788
C	-1.07554	-2.79048	0.09405
C	-1.59172	-1.66477	-0.72456
C	-0.53465	-1.05950	-1.28470
C	1.81879	-0.97340	-0.13102
C	2.37317	0.21787	-0.94706
C	2.94881	-1.90982	0.30926
O	1.33398	1.19560	-1.17109
C	1.71827	2.40871	-0.65869
C	3.08071	2.27084	-0.08686
C	3.45652	0.99623	-0.25122
O	0.99841	3.38274	-0.70101
C	3.78756	3.43056	0.53586
O	-1.68301	-3.58044	0.78418
C	-3.06108	-1.39277	-0.83192
C	-3.66416	-0.61797	0.37266
C	-3.28763	0.85034	0.40968
C	-2.36661	1.40600	1.24586
C	-2.00843	2.87655	1.28827
C	-1.57283	0.60359	2.24682
H	2.70980	-0.12680	-1.93329
H	1.19741	-2.23610	-1.78050
C	3.77986	-2.47632	-0.57293
C	3.05242	-2.15728	1.79181
C	-4.01896	1.67823	-0.56631
O	-4.85666	1.23329	-1.34423
H	-0.54514	-0.20508	-1.94839
H	1.33861	-0.55026	0.75884
H	4.39331	0.54369	0.04951
H	4.77286	3.14119	0.91019
H	3.91292	4.24171	-0.19097
H	3.20195	3.83932	1.36761
H	-3.27489	-0.84154	-1.75153
H	-3.57822	-2.35753	-0.90030
H	-4.75282	-0.67871	0.27165
H	-3.40352	-1.13089	1.30087
H	-2.75714	3.54218	0.85891
H	-1.84750	3.18445	2.32838
H	-1.06329	3.04754	0.75532
H	-1.66655	-0.47623	2.13357

H	-0.50937	0.86670	2.17416
H	-1.88382	0.87014	3.26678
H	4.57440	-3.14316	-0.24886
H	3.70067	-2.31568	-1.64564
H	3.20474	-1.21773	2.34045
H	2.12109	-2.59866	2.16872
H	3.87840	-2.83357	2.03136
H	-3.78419	2.75675	-0.59138

Conformer 12
Energy: -1152.65906 Hartree (Rel: 2.0 kcal/mol)
XYZ coordinates for conf 12:

C	-0.50563	-0.06230	0.22212
O	0.04958	0.14094	-1.09111
C	1.27962	0.73636	-0.98413
C	1.58876	0.93258	0.45590
C	0.54623	0.46238	1.15385
C	-1.89496	0.61766	0.34123
C	-2.89310	-0.02444	-0.65082
C	-1.84624	2.13683	0.20350
O	-2.95219	-1.44833	-0.41899
C	-4.24404	-1.82010	-0.14182
C	-5.10412	-0.60965	-0.19179
C	-4.31565	0.43132	-0.48869
O	-4.54791	-2.96833	0.09269
C	-6.57395	-0.68666	0.06553
O	1.94260	1.01730	-1.95985
C	2.89356	1.52416	0.90489
C	3.89301	0.47788	1.46713
C	4.09649	-0.74815	0.59272
C	4.79237	-0.73446	-0.57878
C	4.98485	-1.91107	-1.50550
C	5.43196	0.52474	-1.10863
H	-2.53541	0.12042	-1.67639
H	-0.64851	-1.14144	0.35078
C	-1.63774	2.74597	-0.97035
C	-2.05101	2.91477	1.48058
C	3.46826	-1.98190	1.09610
O	2.81734	-2.05259	2.13337
H	0.43873	0.42771	2.23229
H	-2.25627	0.36666	1.34679
H	-4.61113	1.46694	-0.60775
H	-7.04402	0.29714	-0.01244
H	-7.05631	-1.36330	-0.64970
H	-6.77110	-1.09149	1.06523
H	3.33702	2.04348	0.04988
H	2.71319	2.27766	1.68070
H	4.84650	0.98619	1.65397
H	3.52252	0.12833	2.43433
H	4.44971	-2.81807	-1.22701
H	4.66345	-1.62841	-2.51643
H	6.05358	-2.15306	-1.57760
H	5.64992	1.25928	-0.32987
H	6.36231	0.29365	-1.63966
H	4.75209	0.99440	-1.83236
H	-1.60736	3.83069	-1.03764
H	-1.47960	2.20151	-1.89577
H	-3.02965	2.69033	1.92644
H	-1.29680	2.64617	2.23247
H	-1.99090	3.99363	1.30884
H	3.61070	-2.89586	0.49365

Conformer 13
Energy: -1152.66210 Hartree (Rel: 2.0 kcal/mol)

XYZ coordinates for conf 13:

C	1.03330	0.41200	-0.30280
O	0.65120	-0.48660	-1.35970
C	-0.66850	-0.83910	-1.21590
C	-1.21860	-0.12970	-0.02960
C	-0.22400	0.59950	0.49370
C	2.23950	-0.15390	0.49310
C	3.48290	-0.25710	-0.42130
C	1.93920	-1.46890	1.20720
O	3.76570	1.03930	-0.99150
C	5.04150	1.42290	-0.66160
C	5.66140	0.34730	0.15420
C	4.75310	-0.62740	0.29070
O	5.50850	2.48150	-1.01850
C	7.05970	0.46580	0.66740
O	-1.21510	-1.60040	-1.98210
C	-2.64900	-0.26430	0.39590
C	-3.64070	0.32680	-0.64030
C	-5.08270	0.25880	-0.17260
C	-5.72580	1.27411	0.46790
C	-7.16880	1.25161	0.91880
C	-5.05160	2.58240	0.80430
H	3.27180	-0.93670	-1.25460
H	1.35410	1.34920	-0.77260
C	1.81280	-2.62980	0.55240
C	1.79610	-1.37990	2.70720
C	-5.74890	-1.03049	-0.43170
O	-5.19620	-1.98610	-0.96350
H	-0.27420	1.24620	1.36320
H	2.47620	0.60970	1.24560
H	4.86850	-1.56380	0.82340
H	7.34980	-0.41870	1.24040
H	7.76560	0.59390	-0.16160
H	7.16310	1.34870	1.30930
H	-2.88920	-1.32600	0.52800
H	-2.78380	0.22620	1.36650
H	-3.35310	1.35480	-0.87570
H	-3.54680	-0.25600	-1.56050
H	-7.74520	0.38790	0.59000
H	-7.21660	1.29550	2.01520
H	-7.67760	2.15280	0.55270
H	-3.96830	2.56840	0.68150
H	-5.45690	3.38690	0.17430
H	-5.27500	2.86250	1.84150
H	1.59840	-3.55120	1.08810
H	1.90280	-2.71000	-0.52610
H	2.71490	-0.99290	3.16860
H	0.99010	-0.68830	2.98750
H	1.57460	-2.35610	3.14870
H	-6.80200	-1.12889	-0.11370

Conformer 14

Energy: -1152.66028 Hartree (Rel: 2.0 kcal/mol)

XYZ coordinates for conf 14:

C	-0.48051	-0.02870	0.02680
O	-0.18291	-0.35650	1.40120
C	1.05969	-0.93341	1.47740
C	1.62249	-1.03641	0.10640
C	0.71519	-0.52060	-0.73410
C	-1.84611	-0.60840	-0.42050
C	-2.98001	-0.02150	0.45650
C	-1.90411	-2.13090	-0.50580
O	-2.93831	1.41980	0.38360
C	-4.14370	1.89620	-0.06830

C	-5.04821	0.74191	-0.30560
C	-4.36851	-0.37060	0.00010
O	-4.35230	3.07911	-0.21980
C	-6.44401	0.93451	-0.80240
O	1.54309	-1.27531	2.53580
C	2.96539	-1.64461	-0.15990
C	4.16419	-0.81361	0.37360
C	4.34369	0.52849	-0.31070
C	4.01820	1.73329	0.23800
C	4.21780	3.07769	-0.42630
C	3.41770	1.88149	1.61480
H	-2.81591	-0.29300	1.50570
H	-0.55841	1.06390	-0.02760
C	-1.97021	-2.71900	-1.70640
C	-1.91181	-2.94670	0.76790
C	4.92769	0.43519	-1.66110
O	5.23979	-0.62421	-2.19470
H	0.79449	-0.44111	-1.81270
H	-2.00371	-0.21050	-1.43090
H	-4.72591	-1.39179	-0.06270
H	-6.96381	-0.02099	-0.91040
H	-7.01471	1.56861	-0.11370
H	-6.44250	1.44421	-1.77320
H	3.00429	-2.62951	0.32300
H	3.09019	-1.80391	-1.23430
H	4.07279	-0.70681	1.45530
H	5.06419	-1.40871	0.18590
H	4.70740	3.05209	-1.39870
H	3.24700	3.57559	-0.54920
H	4.81250	3.72429	0.23210
H	3.15259	0.94189	2.09790
H	4.11920	2.41769	2.26910
H	2.51500	2.50419	1.56230
H	-2.01252	-3.80080	-1.80630
H	-1.97911	-2.14590	-2.63040
H	-1.00641	-2.79360	1.36350
H	-2.75501	-2.67940	1.41800
H	-1.99412	-4.01330	0.53940
H	5.08859	1.37789	-2.21270

LIST OF FILES (technical info - delete in the final SI version
kallopterolideA-epi8-du8ml-chloroform_1027.log kallopterolideA-epi8-du8ml-chloroform_1083.log
kallopterolideA-epi8-du8ml-chloroform_1023.log kallopterolideA-epi8-du8ml-chloroform_103.log
kallopterolideA-epi8-du8ml-chloroform_1053.log kallopterolideA-epi8-du8ml-chloroform_1040.log
kallopterolideA-epi8-du8ml-chloroform_1019.log kallopterolideA-epi8-du8ml-chloroform_5.log
kallopterolideA-epi8-du8ml-chloroform_104.log kallopterolideA-epi8-du8ml-chloroform_1031.log
kallopterolideA-epi8-du8ml-chloroform_1055.log kallopterolideA-epi8-du8ml-chloroform_1073.log
kallopterolideA-epi8-du8ml-chloroform_1077.log kallopterolideA-epi8-du8ml-chloroform_1038.log

DU8ML data for 3-methyl-2-(2-((*R*)-5-((*S*)-2-methyl-1-((*R*)-4-methyl-5-oxo-2,5-dihydrofuran-2-yl)allyl)-2-oxo-2,5-dihydrofuran-3-yl)ethyl)but-2-enal (**6*R*,7*S*,8*R* diastereomer**)

NMR parameters calculated for 6*R*,7*S*,8*R* diastereomer vs experimental data of kallopterolide A (**1**)

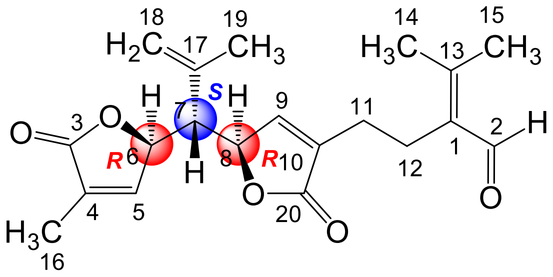

Parameter	RMSD
J_{HH}	0.41 Hz
δ_{1H}	0.19 ppm
δ_{13C}	1.28 ppm

	Conf1	Conf2	Conf3	Conf4	Conf5	Conf6	Conf7	Conf8	Conf9	Conf10	Conf11	Conf12	Conf13	Conf14	Conf15	Conf16	Conf17	Conf18	Conf19	Conf20
Rel energy (kcal/mol):	0.00	0.10	0.72	0.91	0.93	0.93	0.93	1.00	1.03	1.08	1.45	1.67	1.75	1.84	1.88	1.92	1.92	1.96	1.97	2.00

iGau	jGau	Jexp	Jcalc	diff		1	2	3	4	5	6	7	8	9	10	11	12	13	14	15	16	17	18	19	20			
30	31	1.60	1.51	-0.09	[	-1.51	-1.51	-1.51	-1.51	-1.53	-1.53	-1.51	-1.54	-1.54	-1.51	-1.51	-1.50	-1.52	-1.50	-1.51	-1.53	-1.50	-1.51	-1.51	-1.51	]	H5	H16-Me
22	29	4.00	4.20	0.20	[	2.40	2.38	2.44	2.78	10.26	10.24	2.77	10.49	10.49	2.41	2.39	2.65	10.31	3.28	2.38	10.75	2.35	2.41	2.86	2.40	]	H6	H7
22	30	2.00	2.06	0.06	[	2.08	2.08	2.08	2.06	1.99	1.99	2.06	2.04	2.04	2.08	2.08	2.06	1.99	2.06	2.08	2.02	2.08	2.08	2.06	2.08	]	H6	H5
22	31	1.90	1.92	0.02	[	1.98	1.98	1.97	2.00	1.70	1.70	2.00	1.71	1.71	1.98	1.99	1.99	1.70	2.01	1.98	1.70	1.98	1.98	2.00	1.98	]	H6	H16-Me
23	29	10.00	10.91	0.91	[	10.95	11.00	10.86	10.76	10.93	10.98	10.79	10.82	10.79	10.94	10.87	10.65	10.86	10.71	10.93	10.81	11.02	11.00	10.77	10.91	]	H8	H7
23	28	1.60	1.94	0.34	[	1.94	1.92	1.98	1.94	1.92	1.91	1.92	1.95	1.97	1.91	1.97	1.93	1.97	1.97	1.96	1.99	1.95	1.99	1.90	1.99	]	H8	H9

For Js: RMSD=0.41Hz N=6 {-0.09 0.91}

NOTICE:
removed constants H11 and H12 from A-exp set, b/c they are not available in the B-exp set

H-nom	iGau	Exp	Calc	diff	1	2	3	4	5	6	7	8	9	10	11	12	13

H2	49	10.10	10.06	-0.04	[	10.09	10.08	9.93	10.08	10.08	10.08	10.08	10.08	10.08	10.10	10.12	10.00	9.86
H5	30	7.23	7.34	0.11	[	7.19	7.18	7.17	7.23	7.90	7.90	7.21	7.86	7.87	7.17	7.16	7.28	7.83
H6	22	5.35	5.34	-0.01	[	5.40	5.40	5.30	5.46	5.08	5.08	5.45	5.18	5.18	5.37	5.38	5.51	5.01
H7	29	2.41	2.07	-0.34	[	2.09	2.03	1.85	2.41	1.83	1.77	2.36	2.25	2.29	2.01	1.94	2.47	1.59
H8	23	5.23	5.21	-0.02	[	5.25	5.27	5.19	5.45	4.97	5.00	5.47	5.05	5.02	5.14	5.19	5.31	4.93
H9	28	7.31	7.37	0.06	[	7.39	7.40	7.51	7.35	7.37	7.37	7.35	7.41	7.42	7.15	7.16	6.89	7.50
H11	34	2.27	2.44	0.17	[	2.22	2.60	2.64	2.22	2.19	2.59	2.59	2.58	2.18	2.73	2.56	2.68	2.65
H11	35	2.27	2.42	0.15	[	2.59	2.18	2.42	2.58	2.60	2.18	2.18	2.20	2.59	2.53	2.76	2.25	2.40
H12	36	2.52	2.54	0.02	[	2.54	2.55	2.23	2.54	2.51	2.53	2.56	2.53	2.53	2.71	2.89	3.05	2.19
H12	37	2.52	2.63	0.11	[	2.54	2.56	3.21	2.53	2.50	2.53	2.56	2.53	2.51	2.85	2.70	2.35	3.24
H14-Me	41	2.04	2.31	0.27	[	2.34	2.34	2.35	2.34	2.32	2.34	2.34	2.34	2.33	1.98	1.98	2.19	2.33
H15-Me	38	2.22	2.27	0.05	[	2.27	2.27	2.27	2.27	2.27	2.28	2.27	2.28	2.28	2.21	2.20	2.25	2.23
H16-Me	31	1.86	2.02	0.16	[	2.02	2.02	2.02	2.04	2.04	2.04	2.03	2.03	2.03	2.01	2.02	2.03	2.02
H18a	45	4.89	5.28	0.39	[	5.32	5.34	5.22	5.13	5.33	5.35	5.06	5.13	5.21	5.27	5.30	5.02	5.24
H18b	44	5.07	5.40	0.33	[	5.43	5.44	5.38	5.25	5.57	5.57	5.20	5.34	5.39	5.38	5.39	5.12	5.49
H19-Me	46	1.72	1.87	0.15	[	1.83	1.78	1.99	1.85	2.04	1.98	1.87	2.08	2.08	1.75	1.75	1.80	2.19

14	15	16	17	18	19	20	
9.95	10.14	9.88	9.98	9.91	10.11	10.12	]
7.24	7.19	7.78	7.17	7.16	7.21	7.19	]
5.32	5.41	5.12	5.39	5.34	5.42	5.42	]
2.23	2.05	2.09	1.99	1.97	2.35	2.08	]
5.34	5.22	4.92	5.05	5.11	5.36	5.22	]
7.48	7.30	7.55	6.96	7.46	7.10	7.32	]
2.63	2.19	2.63	2.27	2.39	2.70	2.59	]
2.43	2.66	2.41	2.63	2.66	2.54	2.22	]
2.25	3.34	2.21	2.30	3.22	2.72	1.94	]
3.13	2.00	3.20	3.17	2.19	2.84	3.31	]
2.35	2.12	2.34	2.18	2.34	1.98	2.14	]
2.28	2.27	2.25	2.26	2.26	2.21	2.27	]
2.04	2.02	2.01	2.01	2.01	2.03	2.02	]
5.76	5.31	5.76	5.25	5.33	5.05	5.31	]
5.33	5.41	5.48	5.36	5.44	5.17	5.41	]
1.74	1.77	2.06	1.74	1.77	1.79	1.80	]

1H chem shifts: RMSD=0.19ppm (MAE=0.15) N=16 {-0.34 0.39}

m=1.000 b=0.00

C-nom	iGau	Exp	Calc	diff		1	2	3	4	5	6	7	8	9	10	11	12	13
C1-C**	18	135.50	136.28	0.78	[	136.72	136.81	134.27	136.72	136.65	136.58	136.76	136.58	136.64	134.69	135.09	136.35	134.24
C2-CH	26	190.60	189.56	-1.04	[	189.45	189.46	190.03	189.42	189.45	189.40	189.47	189.38	189.40	190.02	189.89	191.18	189.94
C3-C	10	173.80	173.47	-0.33	[	173.57	173.54	173.57	173.87	172.97	173.01	173.83	172.88	172.87	173.55	173.45	174.03	173.03
C4-C	11	130.90	132.25	1.35	[	133.13	133.13	132.83	131.07	130.71	130.74	131.03	130.47	130.40	133.11	133.27	130.60	130.46
C5-CH	12	146.80	144.97	-1.83	[	143.70	143.70	143.83	146.39	147.30	147.32	146.44	148.10	148.09	143.67	143.44	147.08	147.38
C6-CH	7	79.80	80.71	0.91	[	79.57	79.57	79.55	81.40	86.01	86.05	81.40	80.67	80.70	79.54	79.51	81.89	86.30
C7-CH	6	53.50	53.25	-0.25	[	52.13	52.13	52.21	55.51	53.83	53.86	55.58	56.66	56.66	52.04	52.51	54.20	53.63

C8-CH	1	80.10	80.72	0.62	[	81.13	81.18	80.85	79.15	81.52	81.50	79.18	80.11	80.04	80.96	80.86	78.63	81.21
C9-CH	5	147.40	146.81	-0.59	[	146.52	146.50	149.53	145.98	146.04	146.06	145.98	146.35	146.45	148.43	148.49	143.69	149.06
C10-C	4	134.60	134.68	0.08	[	135.05	135.00	133.49	134.68	135.16	135.08	134.65	134.33	134.40	134.05	134.08	134.39	133.45
C11-CH2	16	24.50	26.40	1.90	[	27.05	27.04	22.43	27.10	26.86	26.99	27.10	26.99	26.94	25.67	25.56	27.63	22.18
C12-CH2	17	23.40	25.28	1.88	[	25.61	25.59	24.53	25.54	25.78	25.38	25.47	25.35	25.64	24.19	24.43	23.38	24.06
C13-C**	19	156.80	159.17	2.37	[	158.81	158.69	162.93	158.79	158.74	158.78	158.68	158.77	158.73	158.76	157.90	159.42	162.42
C14-CH3	21	23.40	21.84	-1.56	[	21.69	21.67	22.87	21.74	21.70	21.69	21.66	21.67	21.70	21.84	22.01	21.88	22.76
C15-CH3	20	19.40	17.07	-2.33	[	17.03	17.12	17.25	17.01	17.12	17.05	17.08	17.05	17.08	16.93	17.02	16.77	17.17
C16-CH3	14	10.80	11.15	0.35	[	11.17	11.18	11.19	11.13	11.07	11.10	11.17	11.05	11.02	11.18	11.14	11.14	11.04
C17-C	8	137.80	139.06	1.26	[	137.50	137.29	138.67	140.08	144.40	144.28	140.33	140.78	140.57	137.58	137.11	141.01	145.67
C18-CH2	24	117.10	116.38	-0.72	[	116.50	116.71	115.57	118.27	112.17	112.26	118.00	118.12	118.31	116.38	116.72	117.20	111.00
C19-CH3	25	23.70	23.80	0.10	[	25.35	25.32	24.78	19.95	25.80	25.79	19.92	17.60	17.61	25.27	25.34	20.10	25.29
C20-C	3	172.90	173.92	1.02	[	173.99	174.04	174.60	173.85	173.72	173.71	173.84	173.67	173.68	173.86	173.93	172.97	174.38

**14**	**15**	**16**	**17**	**18**	**19**	**20**	
134.16	136.87	134.13	136.16	134.05	134.76	136.88	]
190.20	189.30	190.04	189.68	189.01	189.99	189.10	]
173.99	173.61	172.87	173.57	173.53	173.87	173.55	]
131.18	133.04	130.16	132.96	133.04	131.04	133.13	]
146.08	143.79	148.25	143.78	143.65	146.35	143.73	]
81.12	79.59	80.70	79.59	79.60	81.32	79.56	]
54.90	52.24	56.07	52.13	52.14	55.46	52.28	]
78.67	80.55	79.64	80.32	80.82	78.89	80.45	]
149.25	146.44	149.62	144.81	148.96	147.86	146.51	]
133.11	135.74	132.84	134.63	133.84	133.70	135.87	]
22.85	27.32	22.33	27.50	22.38	25.78	27.37	]
25.09	25.16	24.53	22.77	24.03	24.13	25.47	]
163.18	156.79	162.68	157.72	161.59	158.65	156.72	]
22.90	21.71	22.78	21.82	22.82	21.84	21.71	]
17.30	16.96	17.28	16.61	17.20	16.86	16.76	]
11.14	11.18	11.04	11.15	11.18	11.15	11.18	]
139.67	137.67	139.89	137.67	137.16	140.14	137.49	]
119.94	116.42	119.70	116.39	116.82	118.14	116.44	]
19.70	25.32	17.74	25.36	25.31	19.82	25.42	]
174.35	172.40	174.33	172.77	174.59	173.69	172.37	]

13C chem shifts: RMSD=1.28ppm (MAE=1.06) N=20 {-2.33 2.37}

Fractions: 0.251 0.213 0.075 0.054 0.052 0.052 0.052 0.047 0.044 0.040 0.022 0.015 0.013 0.011 0.011 0.010 0.010 0.009 0.009 0.009

NMR parameters calculated for 6*R*,7*S*,8*R* diastereomer vs experimental data of kallopterolide B (**2**)

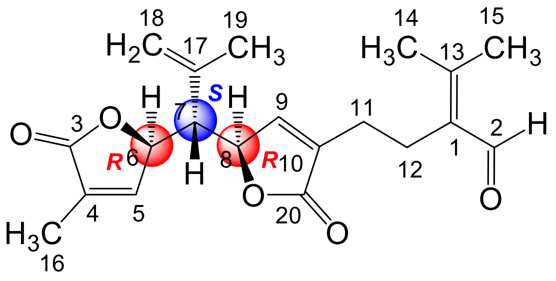

Parameter	RMSD
J_{HH}	1.73 Hz
δ_{1H}	0.25 ppm
δ_{13C}	1.24 ppm

	Conf1	Conf2	Conf3	Conf4	Conf5	Conf6	Conf7	Conf8	Conf9	Conf10	Conf11	Conf12	Conf13	Conf14	Conf15	Conf16	Conf17	Conf18	Conf19	Conf20
Rel energy (kcal/mol):	0.00	0.10	0.72	0.91	0.93	0.93	0.93	1.00	1.03	1.08	1.45	1.67	1.75	1.84	1.88	1.92	1.92	1.96	1.97	2.00

iGau	jGau	Jexp	Jcalc	diff		1	2	3	4	5	6	7	8	9	10	11	12	13	14	15	16	17	18	19	20			
22	30	1.65	2.06	0.41	[	2.08	2.08	2.08	2.06	1.99	1.99	2.06	2.04	2.04	2.08	2.08	2.06	1.99	2.06	2.08	2.02	2.08	2.08	2.06	2.08	]	H6	H5
30	31	1.50	1.51	0.01	[	-1.51	-1.51	-1.51	-1.51	-1.53	-1.53	-1.51	-1.54	-1.54	-1.51	-1.51	-1.50	-1.52	-1.50	-1.51	-1.53	-1.50	-1.51	-1.51	-1.51	]	H5	H16-Me
22	29	7.00	4.20	-2.80	[	2.40	2.38	2.44	2.78	10.26	10.24	2.77	10.49	10.49	2.41	2.39	2.65	10.31	3.28	2.38	10.75	2.35	2.41	2.86	2.40	]	H6	H7
22	31	1.70	1.92	0.22	[	1.98	1.98	1.97	2.00	1.70	1.70	2.00	1.71	1.71	1.98	1.99	1.99	1.70	2.01	1.98	1.70	1.98	1.98	2.00	1.98	]	H6	H16-Me
23	29	7.80	10.91	3.11	[	10.95	11.00	10.86	10.76	10.93	10.98	10.79	10.82	10.79	10.94	10.87	10.65	10.86	10.71	10.93	10.81	11.02	11.00	10.77	10.91	]	H8	H7
23	28	1.40	1.94	0.54	[	1.94	1.92	1.98	1.94	1.92	1.91	1.92	1.95	1.97	1.91	1.97	1.93	1.97	1.97	1.96	1.99	1.95	1.99	1.90	1.99	]	H8	H9

For Js: RMSD=1.73Hz N=6 {-2.80 3.11}

H-nom	iGau	Exp	Calc	diff		1	2	3	4	5	6	7	8	9	10	11	12	13
H2	49	10.10	10.06	-0.04	[	10.09	10.08	9.93	10.08	10.08	10.08	10.08	10.08	10.08	10.10	10.12	10.00	9.86
H5	30	7.10	7.34	0.24	[	7.19	7.18	7.17	7.23	7.90	7.90	7.21	7.86	7.87	7.17	7.16	7.28	7.83
H6	22	5.05	5.34	0.29	[	5.40	5.40	5.30	5.46	5.08	5.08	5.45	5.18	5.18	5.37	5.38	5.51	5.01
H7	29	2.61	2.07	-0.54	[	2.09	2.03	1.85	2.41	1.83	1.77	2.36	2.25	2.29	2.01	1.94	2.47	1.59
H8	23	5.07	5.21	0.14	[	5.25	5.27	5.19	5.45	4.97	5.00	5.47	5.05	5.02	5.14	5.19	5.31	4.93
H9	28	7.13	7.37	0.24	[	7.39	7.40	7.51	7.35	7.37	7.37	7.35	7.41	7.42	7.15	7.16	6.89	7.50
H11	34	2.28	2.44	0.16	[	2.22	2.60	2.64	2.22	2.19	2.59	2.59	2.58	2.18	2.73	2.56	2.68	2.65

H11	35	2.28	2.42	0.14	[	2.59	2.18	2.42	2.58	2.60	2.18	2.18	2.20	2.59	2.53	2.76	2.25	2.40
H12	36	2.52	2.54	0.02	[	2.54	2.55	2.23	2.54	2.51	2.53	2.56	2.53	2.53	2.71	2.89	3.05	2.19
H12	37	2.52	2.63	0.11	[	2.54	2.56	3.21	2.53	2.50	2.53	2.56	2.53	2.51	2.85	2.70	2.35	3.24
H14-Me	41	2.03	2.31	0.28	[	2.34	2.34	2.35	2.34	2.32	2.34	2.34	2.34	2.33	1.98	1.98	2.19	2.33
H15-Me	38	2.21	2.27	0.06	[	2.27	2.27	2.27	2.27	2.27	2.28	2.27	2.28	2.28	2.21	2.20	2.25	2.23
H16-Me	31	1.95	2.02	0.07	[	2.02	2.02	2.02	2.04	2.04	2.04	2.03	2.03	2.03	2.01	2.02	2.03	2.02
H18a	44	4.83	5.40	0.57	[	5.43	5.44	5.38	5.25	5.57	5.57	5.20	5.34	5.39	5.38	5.39	5.12	5.49
H18b	45	5.08	5.28	0.20	[	5.32	5.34	5.22	5.13	5.33	5.35	5.06	5.13	5.21	5.27	5.30	5.02	5.24
H19-Me	46	1.81	1.87	0.06	[	1.83	1.78	1.99	1.85	2.04	1.98	1.87	2.08	2.08	1.75	1.75	1.80	2.19

**14**	**15**	**16**	**17**	**18**	**19**	**20**	
9.95	10.14	9.88	9.98	9.91	10.11	10.12	]
7.24	7.19	7.78	7.17	7.16	7.21	7.19	]
5.32	5.41	5.12	5.39	5.34	5.42	5.42	]
2.23	2.05	2.09	1.99	1.97	2.35	2.08	]
5.34	5.22	4.92	5.05	5.11	5.36	5.22	]
7.48	7.30	7.55	6.96	7.46	7.10	7.32	]
2.63	2.19	2.63	2.27	2.39	2.70	2.59	]
2.43	2.66	2.41	2.63	2.66	2.54	2.22	]
2.25	3.34	2.21	2.30	3.22	2.72	1.94	]
3.13	2.00	3.20	3.17	2.19	2.84	3.31	]
2.35	2.12	2.34	2.18	2.34	1.98	2.14	]
2.28	2.27	2.25	2.26	2.26	2.21	2.27	]
2.04	2.02	2.01	2.01	2.01	2.03	2.02	]
5.33	5.41	5.48	5.36	5.44	5.17	5.41	]
5.76	5.31	5.76	5.25	5.33	5.05	5.31	]
1.74	1.77	2.06	1.74	1.77	1.79	1.80	]

1H chem shifts: RMSD=0.25ppm (MAE=0.20) N=16 {-0.54 0.57}
m=1.000 b=0.00

**C-nom**	**iGau**	**Exp**	**Calc**	**diff**		**1**	**2**	**3**	**4**	**5**	**6**	**7**	**8**	**9**	**10**	**11**	**12**	**13**
C1-C**	18	135.40	136.28	0.88	[	136.72	136.81	134.27	136.72	136.65	136.58	136.76	136.58	136.64	134.69	135.09	136.35	134.24
C2-CH	26	190.70	189.56	-1.14	[	189.45	189.46	190.03	189.42	189.45	189.40	189.47	189.38	189.40	190.02	189.89	191.18	189.94
C3-C	10	173.50	173.47	-0.03	[	173.57	173.54	173.57	173.87	172.97	173.01	173.83	172.88	172.87	173.55	173.45	174.03	173.03
C4-C	11	130.90	132.25	1.35	[	133.13	133.13	132.83	131.07	130.71	130.74	131.03	130.47	130.40	133.11	133.27	130.60	130.46
C5-CH	12	147.00	144.97	-2.03	[	143.70	143.70	143.83	146.39	147.30	147.32	146.44	148.10	148.09	143.67	143.44	147.08	147.38
C6-CH	7	80.00	80.71	0.71	[	79.57	79.57	79.55	81.40	86.01	86.05	81.40	80.67	80.70	79.54	79.51	81.89	86.30
C7-CH	6	52.80	53.25	0.45	[	52.13	52.13	52.21	55.51	53.83	53.86	55.58	56.66	56.66	52.04	52.51	54.20	53.63
C8-CH	1	79.90	80.72	0.82	[	81.13	81.18	80.85	79.15	81.52	81.50	79.18	80.11	80.04	80.96	80.86	78.63	81.21
C9-CH	5	146.80	146.81	0.01	[	146.52	146.50	149.53	145.98	146.04	146.06	145.98	146.35	146.45	148.43	148.49	143.69	149.06
C10-C	4	134.60	134.68	0.08	[	135.05	135.00	133.49	134.68	135.16	135.08	134.65	134.33	134.40	134.05	134.08	134.39	133.45
C11-CH2	16	24.60	26.40	1.80	[	27.05	27.04	22.43	27.10	26.86	26.99	27.10	26.99	26.94	25.67	25.56	27.63	22.18
C12-CH2	17	23.40	25.28	1.88	[	25.61	25.59	24.53	25.54	25.78	25.38	25.47	25.35	25.64	24.19	24.43	23.38	24.06
C13-C**	19	156.90	159.17	2.27	[	158.81	158.69	162.93	158.79	158.74	158.78	158.68	158.77	158.73	158.76	157.90	159.42	162.42
C14-CH3	21	23.40	21.84	-1.56	[	21.69	21.67	22.87	21.74	21.70	21.69	21.66	21.67	21.70	21.84	22.01	21.88	22.76

C15-CH3	20	19.40	17.07	-2.33	[	17.03	17.12	17.25	17.01	17.12	17.05	17.08	17.05	17.08	16.93	17.02	16.77	17.17
C16-CH3	14	10.70	11.15	0.45	[	11.17	11.18	11.19	11.13	11.07	11.10	11.17	11.05	11.02	11.18	11.14	11.14	11.04
C17-C	8	139.20	139.06	-0.14	[	137.50	137.29	138.67	140.08	144.40	144.28	140.33	140.78	140.57	137.58	137.11	141.01	145.67
C18-CH2	24	116.80	116.38	-0.42	[	116.50	116.71	115.57	118.27	112.17	112.26	118.00	118.12	118.31	116.38	116.72	117.20	111.00
C19-CH3	25	23.60	23.80	0.20	[	25.35	25.32	24.78	19.95	25.80	25.79	19.92	17.60	17.61	25.27	25.34	20.10	25.29
C20-C	3	173.00	173.92	0.92	[	173.99	174.04	174.60	173.85	173.72	173.71	173.84	173.67	173.68	173.86	173.93	172.97	174.38

**14**	**15**	**16**	**17**	**18**	**19**	**20**	
134.16	136.87	134.13	136.16	134.05	134.76	136.88	]
190.20	189.30	190.04	189.68	189.01	189.99	189.10	]
173.99	173.61	172.87	173.57	173.53	173.87	173.55	]
131.18	133.04	130.16	132.96	133.04	131.04	133.13	]
146.08	143.79	148.25	143.78	143.65	146.35	143.73	]
81.12	79.59	80.70	79.59	79.60	81.32	79.56	]
54.90	52.24	56.07	52.13	52.14	55.46	52.28	]
78.67	80.55	79.64	80.32	80.82	78.89	80.45	]
149.25	146.44	149.62	144.81	148.96	147.86	146.51	]
133.11	135.74	132.84	134.63	133.84	133.70	135.87	]
22.85	27.32	22.33	27.50	22.38	25.78	27.37	]
25.09	25.16	24.53	22.77	24.03	24.13	25.47	]
163.18	156.79	162.68	157.72	161.59	158.65	156.72	]
22.90	21.71	22.78	21.82	22.82	21.84	21.71	]
17.30	16.96	17.28	16.61	17.20	16.86	16.76	]
11.14	11.18	11.04	11.15	11.18	11.15	11.18	]
139.67	137.67	139.89	137.67	137.16	140.14	137.49	]
119.94	116.42	119.70	116.39	116.82	118.14	116.44	]
19.70	25.32	17.74	25.36	25.31	19.82	25.42	]
174.35	172.40	174.33	172.77	174.59	173.69	172.37	]

13C chem shifts: RMSD=1.24ppm (MAE=0.97) N=20 {-2.33 2.27}

Fractions: 0.251 0.213 0.075 0.054 0.052 0.052 0.052 0.047 0.044 0.040 0.022 0.015 0.013 0.011 0.011 0.010 0.010 0.009 0.009 0.009

Conformer 1
Energy: -1152.66068 Hartree (Rel: 0.0 kcal/mol)
XYZ coordinates for conf 1:

C	-0.97360	-0.37091	-0.89950
O	-0.46379	-1.62161	-0.38450
C	0.90571	-1.58621	-0.35730
C	1.35730	-0.27160	-0.88140
C	0.26130	0.43169	-1.19600
C	-1.92000	0.26589	0.14510
C	-3.05720	-0.72351	0.51370
C	-2.42120	1.63829	-0.29630
O	-3.87650	-1.00911	-0.63490
C	-5.18410	-0.69642	-0.35910
C	-5.26640	-0.20192	1.04000
C	-4.02640	-0.22402	1.54510
O	-6.06740	-0.84212	-1.17420
C	-6.56920	0.20428	1.64800
O	1.56461	-2.52330	0.04160
C	2.81190	0.07190	-0.96650
C	3.44550	0.28840	0.43280
C	4.94780	0.48290	0.36760
C	5.84380	-0.53909	0.45880
C	7.34710	-0.39029	0.42020
C	5.41591	-1.97799	0.62250
H	-2.59229	-1.66671	0.82330
H	-1.53500	-0.60421	-1.81100
C	-3.00300	1.84279	-1.48460
C	-2.21460	2.75799	0.69440
C	5.37650	1.87380	0.14610
O	4.59810	2.81210	0.01100
H	0.21890	1.43189	-1.61020
H	-1.32840	0.38509	1.06200
H	-3.72020	0.06128	2.54570
H	-6.44230	0.52518	2.68520
H	-7.02070	1.02618	1.07990
H	-7.28340	-0.62712	1.62260
H	3.33700	-0.74810	-1.47210
H	2.95090	0.97370	-1.57070
H	3.00020	1.18580	0.87300
H	3.18910	-0.55370	1.07920
H	7.71250	0.63601	0.43050
H	7.78590	-0.91179	1.28070
H	7.74640	-0.88519	-0.47520
H	4.35671	-2.15590	0.43220
H	5.99851	-2.61909	-0.05110
H	5.64311	-2.32020	1.64240
H	-3.34980	2.83229	-1.77200
H	-3.16580	1.04749	-2.20500
H	-2.71040	2.53769	1.64950
H	-1.14790	2.89309	0.91940
H	-2.60861	3.70639	0.31740
H	6.46260	2.06651	0.09150

Conformer 2
Energy: -1152.66072 Hartree (Rel: 0.1 kcal/mol)
XYZ coordinates for conf 2:

C	-0.91159	0.00291	-0.46000
O	-0.41649	-1.35019	-0.34670
C	0.91071	-1.32960	-0.00710
C	1.34881	0.08481	0.11110
C	0.28661	0.85641	-0.15570
C	-2.10139	0.19721	0.50910
C	-3.20209	-0.86029	0.22990
C	-2.61579	1.63421	0.50120

O	-3.74699	-0.69319	-1.09180
C	-5.10489	-0.51039	-1.01220
C	-5.50779	-0.58079	0.41670
C	-4.39699	-0.78879	1.13510
O	-5.79109	-0.33929	-1.99480
C	-6.93579	-0.43829	0.83200
O	1.54851	-2.34879	0.15460
C	2.76331	0.43160	0.45770
C	3.74211	0.12460	-0.70600
C	5.19391	0.30960	-0.31030
C	5.97361	-0.69340	0.18150
C	7.42961	-0.56190	0.56410
C	5.45041	-2.09400	0.39410
H	-2.73129	-1.84959	0.26420
H	-1.25269	0.13491	-1.49290
C	-2.95899	2.26851	-0.62670
C	-2.70899	2.30201	1.85150
C	5.69701	1.68580	-0.45210
O	5.00861	2.62490	-0.83780
H	0.24571	1.93911	-0.16210
H	-1.72169	-0.03599	1.51240
H	-4.32279	-0.90159	2.21130
H	-7.04659	-0.52619	1.91600
H	-7.33429	0.53321	0.51670
H	-7.55589	-1.20479	0.35250
H	2.83811	1.49020	0.72410
H	3.06181	-0.15250	1.33730
H	3.56371	-0.88940	-1.07010
H	3.51801	0.81040	-1.52870
H	7.89211	0.39150	0.31080
H	7.54591	-0.72080	1.64450
H	8.00681	-1.35620	0.07320
H	4.36291	-2.17570	0.36550
H	5.86411	-2.76620	-0.37160
H	5.79911	-2.48030	1.36010
H	-3.32139	3.29321	-0.60100
H	-2.90529	1.80151	-1.60480
H	-3.37109	1.74171	2.52530
H	-1.72579	2.34141	2.34000
H	-3.09259	3.32331	1.76940
H	6.75221	1.87000	-0.18290

Conformer 3

Energy: -1152.66067 Hartree (Rel: 0.7 kcal/mol)

XYZ coordinates for conf 3:

C	0.47000	-1.15350	0.70738
O	-0.16412	-1.76750	-0.43672
C	-1.52262	-1.79830	-0.25139
C	-1.83396	-1.19861	1.07089
C	-0.67178	-0.83186	1.62723
C	1.28314	0.07806	0.24226
C	2.31641	-0.34154	-0.83623
C	1.89678	0.84354	1.41163
O	3.27448	-1.26751	-0.29000
C	4.54268	-0.77056	-0.45341
C	4.45172	0.53271	-1.16179
C	3.15415	0.77516	-1.38722
O	5.52371	-1.36661	-0.06796
C	5.67417	1.31946	-1.50636
O	-2.27148	-2.26700	-1.08190
C	-3.24595	-1.05445	1.55917
C	-3.82062	0.37757	1.38770
C	-3.68352	0.95939	-0.00947
C	-4.44023	0.56170	-1.07150

C	-4.31410	1.07192	-2.48701
C	-5.49601	-0.50719	-0.94075
H	1.77548	-0.86737	-1.63168
H	1.14512	-1.90064	1.13916
C	2.62310	0.24164	2.36186
C	1.62970	2.32907	1.42593
C	-2.64457	1.99041	-0.15773
O	-1.91014	2.36774	0.75126
H	-0.52299	-0.35845	2.59005
H	0.57118	0.73719	-0.27008
H	2.72518	1.63380	-1.89237
H	5.41913	2.24075	-2.03665
H	6.23334	1.58044	-0.60007
H	6.34999	0.72807	-2.13534
H	-3.86712	-1.77316	1.01549
H	-3.29919	-1.31938	2.62156
H	-4.87149	0.36080	1.69970
H	-3.29410	1.04607	2.07393
H	-3.49983	1.77423	-2.66118
H	-4.17350	0.21943	-3.16430
H	-5.25237	1.55534	-2.78954
H	-5.86206	-0.63326	0.08043
H	-6.34975	-0.29018	-1.59283
H	-5.07532	-1.46769	-1.26823
H	3.04715	0.81278	3.18414
H	2.83241	-0.82345	2.35779
H	2.05570	2.81096	0.53484
H	0.55191	2.53496	1.40694
H	2.06649	2.80666	2.30838
H	-2.53594	2.45128	-1.15468

Conformer 4

Energy: -1152.65986 Hartree (Rel: 0.9 kcal/mol)

XYZ coordinates for conf 4:

C	-0.97350	-0.26540	-0.88720
O	-0.47751	-1.54770	-0.44230
C	0.89239	-1.53141	-0.42180
C	1.35840	-0.19541	-0.87430
C	0.27020	0.53790	-1.14330
C	-1.91070	0.32300	0.19620
C	-3.05020	-0.67939	0.50950
C	-2.36960	1.73861	-0.14180
O	-3.86381	-0.94629	-0.65210
C	-5.18610	-0.73648	-0.34600
C	-5.27900	-0.28328	1.06580
C	-4.03290	-0.23689	1.55380
O	-6.06900	-0.90938	-1.15660
C	-6.59500	0.03432	1.69830
O	1.54089	-2.49781	-0.08000
C	2.81700	0.13419	-0.94690
C	3.45920	0.26519	0.45920
C	4.96440	0.43728	0.39790
C	5.84300	-0.60332	0.42820
C	7.34860	-0.47833	0.39140
C	5.39129	-2.04162	0.51360
H	-2.57981	-1.62889	0.79250
H	-1.53900	-0.44600	-1.80850
C	-2.14949	2.72670	0.73390
C	-3.03910	2.00561	-1.47180
C	5.41660	1.83088	0.25310
O	4.65451	2.78868	0.17460
H	0.24440	1.56310	-1.49390
H	-1.31920	0.37490	1.11880
H	-3.73060	0.05361	2.55390

H	-6.47140	0.34822	2.73800
H	-7.10530	0.83462	1.14940
H	-7.25720	-0.83908	1.67200
H	3.32890	-0.66371	-1.49940
H	2.96490	1.06589	-1.50140
H	3.03090	1.14529	0.94830
H	3.19090	-0.60561	1.06120
H	7.73180	0.53967	0.45200
H	7.78080	-1.05003	1.22290
H	7.73679	-0.93433	-0.52920
H	4.32749	-2.19042	0.32390
H	5.95649	-2.65292	-0.20140
H	5.62169	-2.44552	1.51000
H	-2.46089	3.74761	0.52690
H	-1.65499	2.55420	1.68680
H	-2.39430	1.72691	-2.31570
H	-3.96450	1.43381	-1.59240
H	-3.27709	3.06841	-1.57420
H	6.50570	2.00747	0.20450

Conformer 5
Energy: -1152.66057 Hartree (Rel: 0.9 kcal/mol)
XYZ coordinates for conf 5:

C	-1.08170	0.60580	-1.42990
O	-0.72720	-0.79240	-1.51140
C	0.62370	-0.94230	-1.32780
C	1.22430	0.39980	-1.12190
C	0.23180	1.29700	-1.18410
C	-2.13680	0.84389	-0.32430
C	-3.45220	0.06189	-0.57930
C	-2.42420	2.34179	-0.17620
O	-4.39920	0.43679	0.44460
C	-4.84920	-0.68391	1.09490
C	-4.19660	-1.87261	0.49250
C	-3.38540	-1.43781	-0.48050
O	-5.65260	-0.62281	1.99940
C	-4.48749	-3.25521	0.97930
O	1.15661	-2.03090	-1.35320
C	2.69250	0.56340	-0.87980
C	3.11400	0.05060	0.52230
C	4.61710	0.06881	0.72030
C	5.42570	-0.99229	0.44510
C	6.92260	-1.02699	0.64900
C	4.89281	-2.29449	-0.10340
H	-3.87380	0.37949	-1.54210
H	-1.49850	0.88520	-2.40570
C	-2.82970	3.08919	-1.20910
C	-2.23380	2.90959	1.20640
C	5.15640	1.35411	1.19360
O	4.46570	2.34681	1.39850
H	0.31160	2.37260	-1.08420
H	-1.71240	0.46720	0.61350
H	-2.75630	-2.03561	-1.12600
H	-3.92169	-4.00211	0.41630
H	-4.23479	-3.35271	2.04180
H	-5.55639	-3.48141	0.88690
H	3.23930	-0.00120	-1.64520
H	2.97590	1.61540	-0.98020
H	2.65880	0.70290	1.27380
H	2.71260	-0.95330	0.67640
H	7.34090	-0.16489	1.16760
H	7.19010	-1.92529	1.22050
H	7.42770	-1.11899	-0.32180
H	3.87481	-2.23480	-0.49080

H	5.54601	-2.65609	-0.90750
H	4.91621	-3.06559	0.68020
H	-3.03591	4.14939	-1.08840
H	-2.98860	2.68369	-2.20560
H	-2.89380	2.40009	1.91990
H	-1.20470	2.75740	1.55930
H	-2.45301	3.98109	1.23600
H	6.24650	1.41601	1.35940

Conformer 6

Energy: -1152.65903 Hartree (Rel: 0.9 kcal/mol)

XYZ coordinates for conf 6:

C	-0.96800	0.66010	-0.91250
O	-0.64530	-0.74320	-1.02900
C	0.61830	-0.97260	-0.54780
C	1.19180	0.31870	-0.09140
C	0.26891	1.26530	-0.30640
C	-2.24870	0.86230	-0.06940
C	-3.48760	0.16661	-0.69250
C	-2.52149	2.35580	0.13740
O	-4.63940	0.50521	0.11050
C	-5.26080	-0.64029	0.53850
C	-4.51780	-1.80799	0.00360
C	-3.48810	-1.33760	-0.71220
O	-6.25280	-0.61229	1.23330
C	-4.95340	-3.20899	0.28740
O	1.10700	-2.08200	-0.53590
C	2.57730	0.39640	0.47030
C	3.66190	0.17110	-0.61580
C	5.05910	0.08339	-0.03340
C	5.64350	-1.08631	0.34850
C	7.03900	-1.22351	0.91200
C	4.93840	-2.41711	0.23810
H	-3.65960	0.57871	-1.69560
H	-1.13190	1.03460	-1.93080
C	-2.63789	3.20270	-0.89170
C	-2.66429	2.80060	1.56990
C	5.73351	1.38079	0.13760
O	5.21831	2.45729	-0.14570
H	0.35221	2.32410	-0.09340
H	-2.07070	0.39330	0.90530
H	-2.74140	-1.91270	-1.24280
H	-4.29350	-3.93539	-0.19390
H	-4.95800	-3.40189	1.36680
H	-5.97730	-3.37529	-0.06770
H	2.73720	1.36880	0.94590
H	2.68460	-0.37220	1.24600
H	3.42080	-0.72800	-1.18680
H	3.62950	1.01670	-1.30940
H	7.63780	-0.31351	0.89600
H	6.98740	-1.57911	1.94970
H	7.58320	-1.99381	0.35020
H	3.86620	-2.34330	0.05160
H	5.38720	-3.00981	-0.57210
H	5.08810	-2.99591	1.15840
H	-2.83829	4.25850	-0.73010
H	-2.55599	2.88720	-1.92920
H	-3.49529	2.26900	2.05050
H	-1.76009	2.56840	2.14900
H	-2.85299	3.87590	1.64160
H	6.75631	1.36919	0.55400

Conformer 7

Energy: -1152.65898 Hartree (Rel: 0.9 kcal/mol)

XYZ coordinates for conf 7:

C	-0.91630	0.05620	-0.41711
O	-0.43510	-1.30530	-0.36281
C	0.89560	-1.31230	-0.03631
C	1.35000	0.09159	0.13529
C	0.29370	0.88430	-0.08881
C	-2.09590	0.22030	0.57289
C	-3.20400	-0.81469	0.25369
C	-2.57269	1.66671	0.67069
O	-3.74380	-0.63309	-1.07221
C	-5.11230	-0.53769	-1.00291
C	-5.52140	-0.62599	0.42259
C	-4.40660	-0.77079	1.15029
O	-5.79810	-0.39829	-1.99101
C	-6.95720	-0.54698	0.82869
O	1.52360	-2.34371	0.07699
C	2.77050	0.41019	0.48419
C	3.73840	0.13239	-0.69581
C	5.19430	0.29388	-0.30481
C	5.96760	-0.72822	0.15649
C	7.42670	-0.61932	0.53449
C	5.43330	-2.12922	0.33629
H	-2.73770	-1.80719	0.27339
H	-1.26410	0.23000	-1.44181
C	-2.61709	2.26421	1.86759
C	-2.96129	2.40741	-0.58981
C	5.70921	1.66858	-0.41531
O	5.02751	2.62329	-0.77381
H	0.26821	1.96660	-0.04091
H	-1.71300	-0.07350	1.55839
H	-4.33440	-0.86389	2.22819
H	-7.06950	-0.63348	1.91259
H	-7.39920	0.40292	0.50559
H	-7.53810	-1.34478	0.35109
H	2.85860	1.45809	0.78629
H	3.06780	-0.20671	1.34149
H	3.54930	-0.86831	-1.09021
H	3.51430	0.84549	-1.49481
H	7.89510	0.33778	0.30749
H	7.54760	-0.81172	1.60889
H	7.99520	-1.40272	0.01649
H	4.34549	-2.20261	0.30089
H	5.84619	-2.78782	-0.44151
H	5.77469	-2.53862	1.29549
H	-2.95179	3.29301	1.97569
H	-2.32579	1.75040	2.78059
H	-2.12949	2.45830	-1.30481
H	-3.78849	1.92221	-1.11701
H	-3.25849	3.43361	-0.35511
H	6.76761	1.83638	-0.14791

Conformer 8

Energy: -1152.66052 Hartree (Rel: 1.0 kcal/mol)

XYZ coordinates for conf 8:

C	-0.95379	0.69560	-0.82090
O	-0.62269	-0.69350	-1.04150
C	0.64691	-0.94660	-0.58870
C	1.21381	0.31250	-0.04320
C	0.28051	1.26380	-0.17760
C	-2.24269	0.82550	0.03110
C	-3.42989	0.08120	-0.64140
C	-2.56389	2.29820	0.27810

O	-4.65799	0.47610	0.00460
C	-5.32069	-0.63280	0.46510
C	-4.51999	-1.83490	0.12420
C	-3.42349	-1.41910	-0.52250
O	-6.38419	-0.55450	1.03940
C	-4.97749	-3.20870	0.49320
O	1.14311	-2.05050	-0.66060
C	2.60281	0.36080	0.51320
C	3.68061	0.22540	-0.59430
C	5.08361	0.11200	-0.03060
C	5.68301	-1.07530	0.26400
C	7.08531	-1.23700	0.80310
C	4.98951	-2.40230	0.06840
H	-3.50569	0.39120	-1.69240
H	-1.12189	1.13830	-1.81130
C	-2.41199	2.81690	1.50120
C	-3.04599	3.13320	-0.88580
C	5.74661	1.40140	0.22540
O	5.21881	2.48960	0.02130
H	0.35391	2.30150	0.12340
H	-2.04739	0.34560	0.99660
H	-2.62209	-2.02740	-0.91960
H	-4.26739	-3.96830	0.15600
H	-5.09649	-3.29840	1.57950
H	-5.95709	-3.42260	0.04940
H	2.75781	1.29710	1.05780
H	2.72251	-0.46150	1.22980
H	3.44411	-0.63350	-1.22600
H	3.63361	1.11760	-1.22570
H	7.67461	-0.32170	0.84660
H	7.04811	-1.66680	1.81300
H	7.63181	-1.95890	0.18220
H	3.91541	-2.32650	-0.10590
H	5.43811	-2.93500	-0.78250
H	5.15081	-3.04000	0.94680
H	-2.62099	3.86450	1.70260
H	-2.08319	2.21530	2.34500
H	-2.34929	3.11100	-1.73400
H	-4.01349	2.77300	-1.25630
H	-3.17139	4.17750	-0.58590
H	6.77261	1.37150	0.63320

Conformer 9
Energy: -1152.66102 Hartree (Rel: 1.0 kcal/mol)
XYZ coordinates for conf 9:

C	-1.06429	0.70819	-1.33640
O	-0.69319	-0.67301	-1.54270
C	0.66051	-0.82031	-1.37890
C	1.24461	0.50589	-1.05580
C	0.23961	1.39109	-1.03020
C	-2.13279	0.83009	-0.21940
C	-3.38509	-0.02821	-0.55270
C	-2.49410	2.29699	0.00490
O	-4.45829	0.34329	0.33710
C	-4.91449	-0.76371	1.00630
C	-4.12849	-1.94051	0.55940
C	-3.24339	-1.51271	-0.34990
O	-5.82009	-0.70261	1.80820
C	-4.38739	-3.30681	1.10640
O	1.20611	-1.89591	-1.50280
C	2.71181	0.66700	-0.80560
C	3.14971	0.02900	0.53910
C	4.65421	0.04240	0.72720
C	5.47051	-0.97780	0.34190

C	6.96931	-1.01760	0.53070
C	4.94611	-2.22440	-0.32990
H	-3.72109	0.19869	-1.57360
H	-1.48379	1.06419	-2.28630
C	-2.09910	2.91689	1.12190
C	-3.29420	3.01019	-1.06080
C	5.18590	1.27900	1.32340
O	4.48800	2.23840	1.63440
H	0.30540	2.45209	-0.82280
H	-1.68969	0.43629	0.70200
H	-2.51289	-2.10271	-0.88680
H	-3.71989	-4.04751	0.65830
H	-4.24679	-3.32161	2.19380
H	-5.42429	-3.60931	0.91790
H	3.26181	0.18610	-1.62420
H	2.97930	1.72790	-0.80710
H	2.69501	0.60330	1.35190
H	2.75861	-0.98890	0.60080
H	7.38391	-0.20650	1.12850
H	7.24961	-1.96490	1.00930
H	7.46751	-1.00870	-0.44800
H	3.92071	-2.14150	-0.69240
H	5.59001	-2.49020	-1.17780
H	4.99521	-3.07210	0.36880
H	-2.33040	3.96389	1.30120
H	-1.53910	2.40239	1.89880
H	-2.81870	2.95509	-2.04880
H	-4.29490	2.57239	-1.16200
H	-3.41650	4.06739	-0.80880
H	6.27710	1.33510	1.48450

Conformer 10
Energy: -1152.66216 Hartree (Rel: 1.1 kcal/mol)
XYZ coordinates for conf 10:

C	-0.39319	-0.04010	0.19850
O	-0.06869	-0.71190	1.43560
C	1.15091	-0.28040	1.89070
C	1.69011	0.71780	0.93160
C	0.78941	0.85110	-0.05180
C	-1.73959	0.70520	0.35580
C	-2.85099	-0.28350	0.79700
C	-2.09489	1.51380	-0.88900
O	-3.06999	-1.28720	-0.21150
C	-4.38739	-1.27581	-0.59660
C	-5.10179	-0.24150	0.19600
C	-4.20409	0.33020	1.00860
O	-4.81819	-2.02780	-1.44200
C	-6.56519	0.00219	0.02000
O	1.63851	-0.70390	2.91700
C	3.00751	1.39810	1.14550
C	4.24251	0.46440	1.02140
C	4.45811	-0.10200	-0.36950
C	4.20371	-1.39080	-0.73380
C	4.44531	-1.97270	-2.10920
C	3.65261	-2.42710	0.21520
H	-2.49969	-0.80020	1.69780
H	-0.49019	-0.81350	-0.57160
C	-2.13029	0.97180	-2.11270
C	-2.39919	2.97330	-0.65360
C	4.99681	0.87370	-1.33410
O	5.25181	2.03970	-1.05100
H	0.85551	1.49860	-0.91810
H	-1.61199	1.39590	1.19930
H	-4.38269	1.11729	1.73320

H	-6.92289	0.78499	0.69400
H	-6.78539	0.30099	-1.01170
H	-7.13539	-0.91421	0.21230
H	3.11291	2.22560	0.43890
H	3.01811	1.82730	2.15570
H	5.11821	1.07100	1.27590
H	4.17241	-0.32380	1.77200
H	4.92671	-1.30550	-2.82260
H	5.06741	-2.87270	-2.01910
H	3.49161	-2.30150	-2.54270
H	3.32791	-2.03430	1.17780
H	2.80051	-2.94120	-0.24840
H	4.41131	-3.20080	0.39910
H	-2.38589	1.57360	-2.98130
H	-1.92169	-0.07690	-2.29800
H	-3.23799	3.09490	0.04490
H	-1.53949	3.48680	-0.20180
H	-2.65499	3.48660	-1.58520
H	5.17551	0.52420	-2.36600

Conformer 11

Energy: -1152.65923 Hartree (Rel: 1.4 kcal/mol)

XYZ coordinates for conf 11:

C	0.47608	-1.25537	0.56592
O	0.12513	-2.03079	-0.60129
C	-1.23297	-2.21677	-0.64199
C	-1.83862	-1.56084	0.54539
C	-0.84041	-0.99993	1.24180
C	1.23660	0.01716	0.12459
C	2.48951	-0.36729	-0.70625
C	1.54000	0.94065	1.30100
O	3.41912	-1.12049	0.09385
C	4.63898	-0.49119	0.10087
C	4.54755	0.72485	-0.74878
C	3.29542	0.79072	-1.21871
O	5.58667	-0.92840	0.71410
C	5.72637	1.61780	-0.95985
O	-1.76980	-2.82652	-1.54237
C	-3.31050	-1.60861	0.82079
C	-4.18802	-0.82610	-0.19361
C	-3.97301	0.67572	-0.17978
C	-3.32653	1.37804	-1.15261
C	-3.14146	2.87973	-1.16865
C	-2.72088	0.73207	-2.37571
H	2.16121	-1.02037	-1.52328
H	1.13228	-1.87941	1.18302
C	2.16672	0.51626	2.40523
C	1.08005	2.36941	1.14349
C	-4.55414	1.35313	0.99401
O	-5.15897	0.76787	1.88586
H	-0.91881	-0.44647	2.16998
H	0.57307	0.54302	-0.57457
H	2.88158	1.54516	-1.87909
H	5.48398	2.44706	-1.62957
H	6.07510	2.02915	-0.00522
H	6.56500	1.05535	-1.38670
H	-3.63394	-2.65731	0.79536
H	-3.50693	-1.23210	1.82796
H	-4.04352	-1.24491	-1.19024
H	-5.23012	-1.01753	0.08471
H	-3.65987	3.42385	-0.38056
H	-2.07228	3.12197	-1.10034
H	-3.48466	3.27806	-2.13223
H	-2.65312	-0.35418	-2.32824

H	-3.31031	0.99895	-3.26445
H	-1.71294	1.13077	-2.54949
H	2.36758	1.19734	3.22848
H	2.51024	-0.50551	2.53049
H	1.53725	2.83696	0.26086
H	-0.00787	2.41807	0.99671
H	1.33523	2.97391	2.01889
H	-4.42969	2.44806	1.06333

Conformer 12
Energy: -1152.66043 Hartree (Rel: 1.7 kcal/mol)
XYZ coordinates for conf 12:

C	-0.51795	1.11785	0.81042
O	-0.32175	2.21786	-0.10374
C	1.01608	2.49663	-0.21264
C	1.76416	1.57630	0.68208
C	0.86815	0.79133	1.29241
C	-1.21242	-0.04398	0.05499
C	-2.54247	0.45172	-0.56540
C	-1.33402	-1.30458	0.90594
O	-3.47891	0.88392	0.44582
C	-4.67822	0.24072	0.26764
C	-4.55401	-0.68124	-0.89033
C	-3.30559	-0.56604	-1.36090
O	-5.63079	0.44453	0.98786
C	-5.69893	-1.53087	-1.33781
O	1.43348	3.38166	-0.92596
C	3.25911	1.61119	0.74862
C	3.93444	0.98529	-0.50396
C	3.75086	-0.51681	-0.63023
C	4.55814	-1.43078	-0.01992
C	4.42800	-2.93307	-0.12623
C	5.71589	-1.03103	0.86145
H	-2.30803	1.33038	-1.17814
H	-1.16440	1.48353	1.61620
C	-0.87051	-2.46662	0.42955
C	-1.95654	-1.21463	2.28175
C	2.61183	-0.94262	-1.46057
O	1.82651	-0.16893	-2.00000
H	1.06931	0.00537	2.01103
H	-0.56650	-0.27363	-0.80132
H	-2.86944	-1.09517	-2.20096
H	-5.42686	-2.14150	-2.20276
H	-6.02755	-2.19527	-0.52987
H	-6.56091	-0.90845	-1.60566
H	3.58106	2.65770	0.81580
H	3.60344	1.10099	1.65465
H	3.51200	1.46193	-1.39246
H	4.99745	1.24130	-0.48138
H	3.63836	-3.28884	-0.78644
H	5.37743	-3.35667	-0.47888
H	4.25304	-3.35937	0.87042
H	5.81400	0.04418	1.01020
H	5.61604	-1.50476	1.84703
H	6.65646	-1.40671	0.43584
H	-0.94425	-3.38595	1.00565
H	-0.40982	-2.54349	-0.55232
H	-1.40533	-0.52284	2.93254
H	-2.98601	-0.84487	2.24549
H	-1.95968	-2.19477	2.76775
H	2.46591	-2.02802	-1.59529

Conformer 13
Energy: -1152.65917 Hartree (Rel: 1.8 kcal/mol)
XYZ coordinates for conf 13:

C	0.48214	-1.22221	-1.38422
O	0.05088	0.03751	-1.94384
C	-1.31565	0.13868	-1.86668
C	-1.84630	-1.10036	-1.24575
C	-0.79959	-1.88862	-0.96760
C	1.48559	-0.99517	-0.22892
C	2.76674	-0.25844	-0.70373
C	1.85143	-2.32341	0.44248
O	3.68882	-0.19536	0.40612
C	4.04573	1.10754	0.64148
C	3.35432	1.97176	-0.34673
C	2.61213	1.17664	-1.12831
O	4.81115	1.41451	1.52900
C	3.54612	3.45383	-0.35790
O	-1.90717	1.11482	-2.27445
C	-3.31585	-1.29087	-1.00363
C	-3.73732	-1.09122	0.47674
C	-3.24217	0.19517	1.11678
C	-3.75346	1.42683	0.83424
C	-3.27282	2.73640	1.41204
C	-4.87315	1.61430	-0.15866
H	3.24767	-0.85676	-1.48910
H	0.97052	-1.77819	-2.19466
C	2.29703	-3.37146	-0.26026
C	1.69405	-2.35910	1.94066
C	-2.13654	0.02847	2.07379
O	-1.62112	-1.05109	2.35005
H	-0.81699	-2.86815	-0.50639
H	0.98954	-0.35178	0.50649
H	1.97883	1.47315	-1.95343
H	2.96832	3.92379	-1.15805
H	3.23833	3.89133	0.59927
H	4.60442	3.70604	-0.49439
H	-3.85603	-0.58694	-1.64431
H	-3.61368	-2.30021	-1.31108
H	-4.83049	-1.15569	0.52916
H	-3.33968	-1.92205	1.06525
H	-2.39508	2.67087	2.05376
H	-3.03961	3.42772	0.59174
H	-4.08246	3.20339	1.98837
H	-5.47691	0.71605	-0.30538
H	-5.53388	2.43166	0.15180
H	-4.44629	1.89471	-1.13135
H	2.56091	-4.30445	0.23080
H	2.43190	-3.34695	-1.33911
H	2.35980	-1.62006	2.40506
H	0.67034	-2.09705	2.23693
H	1.93865	-3.34565	2.34654
H	-1.76303	0.93933	2.57226

Conformer 14
Energy: -1152.65902 Hartree (Rel: 1.8 kcal/mol)
XYZ coordinates for conf 14:

C	-0.48790	0.66200	-1.17851
O	0.13850	-0.55170	-1.65001
C	1.49830	-0.38071	-1.70091
C	1.81820	1.00079	-1.26001
C	0.65860	1.61020	-0.98041
C	-1.27500	0.36080	0.12399
C	-2.26130	-0.80840	-0.10681
C	-1.88220	1.62110	0.73449

O	-3.20040	-0.52610	-1.16691
C	-4.47900	-0.74200	-0.71801
C	-4.41810	-1.14230	0.71189
C	-3.12680	-1.16480	1.06639
O	-5.44600	-0.60030	-1.43361
C	-5.65740	-1.43539	1.49369
O	2.24110	-1.26471	-2.07021
C	3.23251	1.48589	-1.13911
C	3.79561	1.38679	0.30539
C	3.68540	0.01349	0.94789
C	4.47810	-1.04311	0.61039
C	4.38550	-2.43711	1.18309
C	5.54400	-0.93231	-0.45041
H	-1.67060	-1.67260	-0.43381
H	-1.17390	0.99240	-1.96621
C	-1.44179	2.04270	1.92679
C	-2.93879	2.38840	-0.02891
C	2.63240	-0.12161	1.96659
O	1.86030	0.77749	2.28869
H	0.52051	2.62380	-0.62401
H	-0.53770	-0.02600	0.83789
H	-2.71750	-1.41400	2.03909
H	-5.42110	-1.71399	2.52399
H	-6.32050	-0.56239	1.51059
H	-6.22240	-2.25259	1.03019
H	3.85590	0.90119	-1.82321
H	3.29361	2.53279	-1.45801
H	4.83950	1.72079	0.28469
H	3.24671	2.09009	0.93739
H	3.57400	-2.60061	1.89099
H	4.26370	-3.15471	0.36109
H	5.32960	-2.69431	1.68109
H	5.87480	0.09269	-0.63001
H	6.41760	-1.53951	-0.18681
H	5.14950	-1.32931	-1.39581
H	-1.84059	2.94360	2.38749
H	-0.66060	1.51770	2.47039
H	-2.61989	2.62200	-1.05231
H	-3.87449	1.82650	-0.11951
H	-3.16389	3.33250	0.47619
H	2.54750	-1.10011	2.46979

Conformer 15

Energy: -1152.65950 Hartree (Rel: 1.9 kcal/mol)

XYZ coordinates for conf 15:

C	-0.90230	-0.05030	-0.44360
O	-0.51880	-1.43610	-0.56570
C	0.79970	-1.58860	-0.21200
C	1.34610	-0.25410	0.15290
C	0.35320	0.63500	0.01400
C	-2.09320	0.06880	0.53700
C	-3.26980	-0.82510	0.06350
C	-2.48880	1.52170	0.78590
O	-3.76920	-0.37740	-1.21040
C	-5.10890	-0.09800	-1.10940
C	-5.54890	-0.39050	0.27970
C	-4.47560	-0.81780	0.95700
O	-5.75600	0.30460	-2.05030
C	-6.96960	-0.20490	0.70310
O	1.34320	-2.67020	-0.22420
C	2.76940	-0.05530	0.57910
C	3.78500	-0.36600	-0.55140
C	5.21940	-0.09741	-0.13520
C	5.88290	1.06479	-0.38760

C	7.32010	1.35209	-0.01620
C	5.24090	2.23310	-1.09630
H	-2.88150	-1.84030	-0.07860
H	-1.21100	0.28910	-1.43880
C	-2.75490	2.37510	-0.21090
C	-2.55760	1.94130	2.23410
C	5.85220	-1.19610	0.61690
O	5.27650	-2.23680	0.91220
H	0.39820	1.70210	0.19700
H	-1.75510	-0.37160	1.48390
H	-4.43630	-1.12980	1.99510
H	-7.11380	-0.48670	1.74940
H	-7.27710	0.83990	0.57620
H	-7.64020	-0.80930	0.08100
H	2.89940	0.97580	0.92670
H	2.99150	-0.71740	1.42450
H	3.68960	-1.42560	-0.80370
H	3.52230	0.20530	-1.44550
H	7.87640	0.50369	0.38050
H	7.85660	1.72039	-0.90030
H	7.35820	2.16099	0.72570
H	4.15850	2.15020	-1.19840
H	5.46350	3.16410	-0.55980
H	5.67180	2.34639	-2.10110
H	-3.03410	3.40510	-0.00290
H	-2.71890	2.08770	-1.25680
H	-3.27930	1.32770	2.79000
H	-1.58640	1.80740	2.72980
H	-2.85380	2.98960	2.33560
H	6.90040	-1.05421	0.93450

Conformer 16

Energy: -1152.65911 Hartree (Rel: 1.9 kcal/mol)

XYZ coordinates for conf 16:

C	0.49650	-1.41829	-1.21365
O	-0.00228	-0.26956	-1.93418
C	-1.36951	-0.21062	-1.82798
C	-1.82964	-1.36748	-1.02076
C	-0.74444	-2.07357	-0.67745
C	1.50295	-0.98752	-0.11366
C	2.66553	-0.15106	-0.71570
C	2.01517	-2.21516	0.63891
O	3.71168	-0.02533	0.27107
C	3.98911	1.29885	0.49227
C	3.10448	2.11593	-0.37484
C	2.33978	1.27239	-1.07976
O	4.83925	1.65624	1.27776
C	3.16547	3.60911	-0.36795
O	-2.01199	0.67382	-2.35162
C	-3.27771	-1.56364	-0.67916
C	-3.64023	-1.10570	0.75975
C	-3.19510	0.30294	1.11760
C	-3.79810	1.42938	0.64246
C	-3.37316	2.84893	0.93269
C	-4.97577	1.36544	-0.29743
H	3.09602	-0.68666	-1.57268
H	1.00264	-2.05486	-1.95085
C	1.58141	-2.45798	1.88079
C	2.99712	-3.12776	-0.05935
C	-2.03215	0.38286	2.01654
O	-1.42602	-0.59254	2.45073
H	-0.70893	-2.97442	-0.07788
H	0.95855	-0.35263	0.59327
H	1.58539	1.52328	-1.81315

H	2.45034	4.03960	-1.07391
H	2.94851	4.00120	0.63283
H	4.17131	3.95681	-0.63192
H	-3.87665	-1.01050	-1.40935
H	-3.54274	-2.62271	-0.77833
H	-4.72384	-1.21378	0.88616
H	-3.16560	-1.78878	1.46920
H	-2.46232	2.95433	1.52074
H	-3.22444	3.38058	-0.01622
H	-4.18130	3.37493	1.45804
H	-5.52850	0.42539	-0.23723
H	-5.67038	2.19112	-0.10559
H	-4.61603	1.47857	-1.32930
H	1.91835	-3.33226	2.43314
H	0.87499	-1.80091	2.38159
H	2.62089	-3.48345	-1.02779
H	3.94639	-2.61403	-0.25416
H	3.21260	-4.00580	0.55640
H	-1.70064	1.39157	2.31705

Conformer 17
Energy: -1152.65911 Hartree (Rel: 1.9 kcal/mol)
XYZ coordinates for conf 17:

C	-0.41820	0.33631	0.02130
O	-0.20000	1.76001	0.11490
C	1.01970	2.08011	-0.42590
C	1.66920	0.82830	-0.89510
C	0.82590	-0.18099	-0.64400
C	-1.72620	0.07091	-0.76050
C	-2.91700	0.79731	-0.08090
C	-1.96900	-1.41829	-0.98870
O	-3.15010	0.27121	1.23870
C	-4.44220	-0.17988	1.33510
C	-5.12780	0.07112	0.04050
C	-4.23700	0.64442	-0.77850
O	-4.87810	-0.67688	2.34960
C	-6.56190	-0.29328	-0.16580
O	1.41381	3.22270	-0.49260
C	3.03610	0.83830	-1.50640
C	4.16920	1.05370	-0.46580
C	4.31420	-0.07380	0.54050
C	5.01030	-1.22040	0.30000
C	5.18509	-2.36490	1.27230
C	5.71060	-1.48571	-1.01020
H	-2.64290	1.85241	0.03400
H	-0.51560	-0.04389	1.04450
C	-1.95890	-2.30989	0.01020
C	-2.21920	-1.82759	-2.41980
C	3.62990	0.15110	1.82740
O	2.97360	1.15270	2.08800
H	0.97460	-1.23209	-0.86080
H	-1.60490	0.56121	-1.73510
H	-4.40000	0.97172	-1.79970
H	-6.89980	-0.02888	-1.17130
H	-6.71220	-1.36918	-0.01730
H	-7.19970	0.21852	0.56450
H	3.20160	-0.09750	-2.05140
H	3.08860	1.65730	-2.23460
H	5.10520	1.20920	-1.01060
H	3.96020	1.97700	0.08040
H	4.65599	-2.25820	2.21820
H	4.85249	-3.30040	0.80390
H	6.25229	-2.49591	1.49580
H	5.66440	-0.65611	-1.71590

H	6.76670	-1.72701	-0.83000
H	5.27370	-2.36960	-1.49480
H	-2.13571	-3.36519	-0.18310
H	-1.78991	-2.03109	1.04530
H	-3.09170	-1.30449	-2.83400
H	-1.36550	-1.56569	-3.05970
H	-2.39581	-2.90399	-2.50580
H	3.72760	-0.63710	2.59410

Conformer 18

Energy: -1152.65937 Hartree (Rel: 2.0 kcal/mol)

XYZ coordinates for conf 18:

C	0.40360	0.13439	0.03500
O	-0.17390	-0.88221	-0.81320
C	-1.40240	-0.46870	-1.26010
C	-1.68740	0.87320	-0.69040
C	-0.63320	1.22019	0.06050
C	1.77290	0.56459	-0.54190
C	2.70350	-0.66741	-0.69630
C	2.39700	1.70789	0.25340
O	2.98770	-1.25681	0.58590
C	4.34480	-1.29711	0.78560
C	5.01570	-0.73541	-0.41570
C	4.05610	-0.37471	-1.27720
O	4.83460	-1.74591	1.79780
C	6.50450	-0.65831	-0.51220
O	-2.07460	-1.14950	-2.00500
C	-2.97660	1.58960	-0.97040
C	-4.00360	1.50850	0.19090
C	-4.26760	0.10870	0.72030
C	-4.98000	-0.83430	0.04220
C	-5.23320	-2.25000	0.50240
C	-5.58020	-0.55640	-1.31340
H	2.16730	-1.41931	-1.28700
H	0.54390	-0.31191	1.02580
C	2.56730	1.65129	1.58000
C	2.80770	2.91609	-0.55280
C	-3.68580	-0.17640	2.04360
O	-3.03350	0.62850	2.70010
H	-0.50150	2.13249	0.62950
H	1.57870	0.91159	-1.56520
H	4.18820	0.06219	-2.26110
H	6.82080	-0.23301	-1.46830
H	6.91230	-0.04161	0.29750
H	6.95070	-1.65411	-0.40610
H	-2.77540	2.64770	-1.17490
H	-3.40810	1.16200	-1.88080
H	-3.62960	2.10630	1.02620
H	-4.93450	1.97850	-0.14840
H	-4.72620	-2.54050	1.42170
H	-6.31120	-2.40540	0.64280
H	-4.92100	-2.94570	-0.28730
H	-5.74560	0.50650	-1.50390
H	-4.90230	-0.93600	-2.08990
H	-6.53390	-1.08360	-1.43030
H	3.01390	2.48239	2.12030
H	2.28320	0.78829	2.17350
H	3.53700	2.64559	-1.32840
H	1.94520	3.35389	-1.07390
H	3.25490	3.68859	0.07980
H	-3.86780	-1.18150	2.46210

Conformer 19
Energy: -1152.66200 Hartree (Rel: 2.0 kcal/mol)
XYZ coordinates for conf 19:

C	-0.39990	0.01100	0.19701
O	-0.09950	-0.70260	1.41620
C	1.12710	-0.31350	1.89010
C	1.69390	0.70160	0.96520
C	0.80261	0.88500	-0.01850
C	-1.73359	0.77941	0.36911
C	-2.85840	-0.20259	0.78380
C	-2.05589	1.65871	-0.83590
O	-3.07210	-1.22479	-0.21260
C	-4.40250	-1.26639	-0.55129
C	-5.11940	-0.21599	0.21651
C	-4.21420	0.41001	0.97930
O	-4.83580	-2.05729	-1.35949
C	-6.59050	-0.00388	0.06280
O	1.59920	-0.77960	2.90501
C	3.02421	1.34590	1.20810
C	4.23870	0.38920	1.05930
C	4.45320	-0.12890	-0.35030
C	4.16250	-1.39310	-0.76960
C	4.39930	-1.92561	-2.16569
C	3.56900	-2.44940	0.13051
H	-2.52470	-0.71329	1.69511
H	-0.50590	-0.74080	-0.59349
C	-2.27109	2.96741	-0.65490
C	-2.10169	1.04151	-2.21630
C	5.03430	0.86729	-1.26869
O	5.32061	2.01259	-0.93580
H	0.88981	1.56550	-0.85750
H	-1.59979	1.43771	1.23690
H	-4.39089	1.23101	1.66531
H	-6.94489	0.80562	0.70620
H	-6.84020	0.24022	-0.97650
H	-7.14080	-0.91818	0.31411
H	3.15161	2.19380	0.52980
H	3.03821	1.74130	2.23201
H	5.12570	0.96539	1.34320
H	4.14390	-0.42460	1.77940
H	4.88800	-1.23721	-2.85350
H	5.01180	-2.83501	-2.10929
H	3.44270	-2.22790	-2.61170
H	3.27300	-2.09250	1.11610
H	2.68760	-2.89500	-0.34909
H	4.28900	-3.26880	0.26431
H	-2.50339	3.62451	-1.48940
H	-2.22719	3.42951	0.32841
H	-1.14770	0.56841	-2.48350
H	-2.86350	0.25961	-2.29569
H	-2.31489	1.80481	-2.97030
H	5.21720	0.55339	-2.31120

Conformer 20
Energy: -1152.66068 Hartree (Rel: 2.0 kcal/mol)
XYZ coordinates for conf 20:

C	0.97600	-0.61174	0.80536
O	0.58686	-1.83178	0.13983
C	-0.78293	-1.91755	0.08658
C	-1.35571	-0.71578	0.74956
C	-0.32912	0.03896	1.16415
C	1.87557	0.22146	-0.13773
C	3.10714	-0.61345	-0.57852
C	2.23920	1.57714	0.46099

O	3.92836	-0.94573	0.55621
C	5.20551	-0.48639	0.35320
C	5.26698	0.16237	-0.98254
C	4.04424	0.08171	-1.52253
O	6.08302	-0.63711	1.17361
C	6.53689	0.74933	-1.50671
O	-1.34539	-2.85610	-0.43098
C	-2.83262	-0.48843	0.86538
C	-3.50529	-0.19349	-0.50126
C	-4.98819	0.10122	-0.36808
C	-5.50553	1.34960	-0.19894
C	-6.97584	1.68166	-0.08259
C	-4.64177	2.58446	-0.11163
H	2.73849	-1.55595	-0.99982
H	1.54454	-0.89719	1.69768
C	2.77474	1.70454	1.68141
C	1.95207	2.77347	-0.41310
C	-5.85339	-1.09284	-0.39646
O	-5.43021	-2.23641	-0.51473
H	-0.38066	0.98269	1.69419
H	1.29099	0.38531	-1.05239
H	3.73226	0.44444	-2.49591
H	6.39849	1.17181	-2.50539
H	6.90129	1.53943	-0.83960
H	7.32409	-0.01229	-1.55405
H	-3.30350	-1.38194	1.29232
H	-3.01519	0.34092	1.55773
H	-2.98351	0.63247	-0.99272
H	-3.38569	-1.07763	-1.13322
H	-7.65541	0.84496	-0.23855
H	-7.18298	2.10731	0.90835
H	-7.23163	2.46149	-0.81169
H	-3.57349	2.37613	-0.04821
H	-4.81491	3.22695	-0.98635
H	-4.92679	3.17864	0.76620
H	3.02384	2.68451	2.08104
H	2.99474	0.85433	2.31900
H	2.48455	2.70048	-1.37100
H	0.88221	2.83621	-0.65489
H	2.25041	3.70777	0.07173
H	-6.94108	-0.93123	-0.29513

LIST OF FILES (technical info - delete in the final SI version
kallopterolideB-du8ml-chloroform_1054.log kallopterolideB-du8ml-chloroform_24.log
kallopterolideB-du8ml-chloroform_104.log kallopterolideB-du8ml-chloroform_1011.log
kallopterolideB-du8ml-chloroform_1010.log kallopterolideB-du8ml-chloroform_27.log
kallopterolideB-du8ml-chloroform_1012.log kallopterolideB-du8ml-chloroform_1015.log
kallopterolideB-du8ml-chloroform_1039.log kallopterolideB-du8ml-chloroform_1061.log
kallopterolideB-du8ml-chloroform_1014.log kallopterolideB-du8ml-chloroform_1078.log
kallopterolideB-du8ml-chloroform_109.log kallopterolideB-du8ml-chloroform_1060.log
kallopterolideB-du8ml-chloroform_1063.log kallopterolideB-du8ml-chloroform_107.log
kallopterolideB-du8ml-chloroform_1080.log kallopterolideB-du8ml-chloroform_1023.log
kallopterolideB-du8ml-chloroform_1077.log kallopterolideB-du8ml-chloroform_1025.log

DU8ML data for 3-methyl-2-(2-((*S*)-5-((*S*)-2-methyl-1-((*R*)-4-methyl-5-oxo-2,5-dihydrofuran-2-yl)allyl)-2-oxo-2,5-dihydrofuran-3-yl)ethyl)but-2-enal (**6*R*,7*S*,8*S* diastereomer**)

NMR parameters calculated for 6*R*,7*S*,8*S* diastereomer vs experimental data of kallopterolide A (**1**)

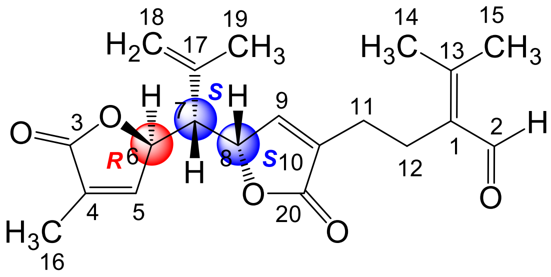

Parameter	RMSD
J_{HH}	1.51 Hz
δ_{1H}	0.18 ppm
δ_{13C}	1.65 ppm

	Conf1	Conf2	Conf3	Conf4	Conf5	Conf6	Conf7	Conf8	Conf9	Conf10	Conf11	Conf12	Conf13
Rel energy (kcal/mol):	0.00	0.09	0.09	0.10	0.12	0.16	0.24	0.27	0.28	0.74	0.85	1.03	1.03

Conf14	Conf15	Conf16	Conf17	Conf18	Conf19	Conf20	Conf21	Conf22	Conf23	Conf24	Conf25	Conf26	Conf27	Conf28	Conf29	Conf30	Conf31
1.10	1.11	1.14	1.15	1.25	1.26	1.35	1.37	1.42	1.43	1.55	1.58	1.59	1.64	1.68	1.81	1.89	1.93

iGau	jGau	Jexp	Jcalc	diff		1	2	3	4	5	6	7	8	9	10	11	12	13	14	15	16
30	31	1.60	1.52	-0.08	[	-1.53	-1.52	-1.51	-1.52	-1.53	-1.53	-1.52	-1.53	-1.52	-1.51	-1.51	-1.51	-1.52	-1.52	-1.53	-1.52
22	29	4.00	6.06	2.06	[	2.74	10.35	10.35	3.97	3.40	2.73	10.72	3.28	10.75	3.56	3.06	4.09	3.51	2.86	2.80	11.17
22	30	2.00	2.01	0.01	[	2.05	1.96	1.96	2.03	2.03	2.05	1.99	2.03	1.99	2.02	2.05	2.05	2.03	2.03	2.05	1.94
22	31	1.90	1.88	-0.02	[	1.90	1.85	1.85	1.91	1.88	1.90	1.87	1.88	1.87	2.07	1.90	1.88	1.86	1.89	1.90	1.73
23	29	10.00	6.96	-3.04	[	10.36	2.72	2.76	1.72	10.71	10.33	3.37	10.84	3.37	10.70	9.35	9.96	10.56	10.03	10.28	3.78
23	28	1.60	1.99	0.39	[	1.94	2.02	2.03	2.05	1.96	1.94	1.99	1.94	2.00	1.98	2.04	2.04	2.00	1.97	1.92	2.04

17	18	19	20	21	22	23	24	25	26	27	28	29	30	31			
-1.52	-1.53	-1.53	-1.52	-1.53	-1.53	-1.53	-1.51	-1.53	-1.54	-1.53	-1.52	-1.51	-1.52	-1.53	]	H5	H16-Me
11.19	3.50	3.61	3.90	11.23	11.24	3.85	10.70	3.44	2.76	3.41	5.35	10.38	10.82	2.83	]	H6	H7
1.93	2.03	2.07	2.01	1.97	1.97	2.06	1.98	2.05	2.05	2.03	2.07	1.96	1.98	2.05	]	H6	H5
1.73	1.88	2.07	1.87	1.72	1.72	2.07	1.88	2.08	1.91	1.89	2.03	1.85	1.87	1.90	]	H6	H16-Me
3.80	10.73	11.18	10.25	5.21	5.33	11.23	3.43	10.96	10.30	10.75	11.26	2.72	3.30	10.14	]	H8	H7
2.03	1.93	1.90	2.00	2.04	2.03	1.91	1.96	2.08	1.97	1.98	1.96	2.09	2.07	1.98	]	H8	H9

For Js: RMSD=1.51Hz N=6 {-3.04 2.06}

NOTICE:
removed constants H11 and H12 from A-exp set, b/c they are not available in the B-exp set

H-nom	iGau	Exp	Calc	diff		1	2	3	4	5	6	7	8	9	10	11	12	13	14	15	16
H2	49	10.10	10.09	-0.01	[	10.10	10.09	10.09	10.09	10.10	10.10	10.09	10.10	10.09	10.14	9.95	9.96	9.96	10.00	10.13	10.10

H5	30	7.23	7.35	0.12	[	7.16	7.55	7.54	7.55	7.22	7.13	7.54	7.19	7.53	7.50	7.22	7.29	7.30	7.14	7.11	7.09
H6	22	5.35	5.10	-0.25	[	5.04	5.26	5.27	4.94	5.07	4.99	5.40	5.02	5.42	4.80	5.27	5.24	5.14	4.76	4.91	5.00
H7	29	2.41	2.37	-0.04	[	2.14	2.12	2.13	2.90	2.48	2.08	2.48	2.45	2.49	2.66	1.80	2.20	2.34	2.21	2.06	2.63
H8	23	5.23	5.14	-0.09	[	5.28	5.03	5.01	4.85	5.43	5.30	5.06	5.46	5.04	4.85	5.17	5.31	5.35	5.15	5.19	4.90
H9	28	7.31	7.39	0.08	[	7.60	7.22	7.22	7.20	7.60	7.62	7.28	7.60	7.28	6.58	7.80	7.77	7.71	7.16	7.36	7.56
H11	34	2.27	2.41	0.14	[	2.63	2.20	2.60	2.15	2.63	2.20	2.21	2.20	2.60	2.23	2.46	2.47	2.48	2.29	2.57	2.63
H11	35	2.27	2.46	0.19	[	2.25	2.57	2.17	2.57	2.25	2.64	2.58	2.64	2.17	2.57	2.63	2.63	2.63	2.72	2.69	2.26
H12	36	2.52	2.57	0.05	[	2.57	2.52	2.48	2.50	2.55	2.59	2.52	2.59	2.52	2.27	3.19	3.16	3.11	2.38	2.82	2.64
H12	37	2.52	2.56	0.04	[	2.53	2.54	2.51	2.48	2.52	2.56	2.53	2.56	2.55	2.97	2.27	2.26	2.28	3.07	2.75	2.57
H14-Me	41	2.04	2.31	0.27	[	2.35	2.33	2.33	2.31	2.34	2.35	2.33	2.34	2.33	2.24	2.32	2.32	2.33	2.22	1.94	2.37
H15-Me	38	2.22	2.28	0.06	[	2.28	2.27	2.27	2.27	2.28	2.28	2.27	2.28	2.27	2.59	2.27	2.27	2.27	2.27	2.23	2.30
H16-Me	31	1.86	2.05	0.19	[	2.03	2.08	2.07	2.10	2.04	2.02	2.08	2.03	2.07	2.10	2.03	2.05	2.04	2.01	2.01	1.99
H18a	45	4.89	5.26	0.37	[	5.38	5.35	5.38	5.43	5.07	5.38	5.05	5.04	5.12	5.35	5.30	5.04	5.02	5.30	5.34	5.47
H18b	44	5.07	5.35	0.28	[	5.41	5.38	5.41	5.42	5.21	5.42	5.18	5.20	5.26	5.61	5.36	5.16	5.16	5.37	5.39	5.70
H19-Me	46	1.72	1.91	0.19	[	1.88	1.84	1.91	1.91	1.92	1.86	1.90	1.93	1.93	2.09	1.83	1.82	1.87	1.94	1.85	2.03

17	18	19	20	21	22	23	24	25	26	27	28	29	30	31	
10.11	10.13	10.08	10.01	10.10	10.10	10.10	10.11	10.23	10.11	10.11	10.10	9.90	9.90	10.11	]
7.06	7.17	7.48	7.20	7.32	7.31	7.49	7.49	7.52	7.10	7.16	7.40	7.50	7.49	7.14	]
4.88	4.95	4.85	4.80	4.86	4.76	4.91	5.39	4.87	4.88	4.92	4.97	5.21	5.36	4.96	]
2.64	2.41	2.60	2.51	3.10	3.11	2.68	2.41	2.62	1.98	2.33	3.13	2.10	2.47	2.09	]
4.92	5.34	4.97	5.29	4.99	5.02	4.93	4.94	4.95	5.22	5.37	4.81	4.85	4.89	5.26	]
7.57	7.34	7.13	7.16	7.49	7.49	7.20	7.04	6.81	7.39	7.38	7.41	7.31	7.36	7.54	]
2.27	2.57	2.17	2.29	2.65	2.24	2.58	2.56	2.39	2.88	2.89	2.60	2.64	2.65	2.65	]
2.65	2.70	2.56	2.71	2.24	2.66	2.14	2.73	2.54	2.58	2.57	2.14	2.38	2.40	2.24	]
2.55	2.82	2.52	2.38	2.60	2.56	2.50	2.86	2.74	2.70	2.69	2.51	2.20	2.22	2.02	]
2.55	2.75	2.52	3.06	2.56	2.54	2.60	2.71	2.24	2.98	2.97	2.61	3.18	3.17	3.33	]
2.35	1.96	2.33	2.22	2.36	2.35	2.33	1.95	2.01	2.02	2.01	2.33	2.33	2.33	2.15	]
2.29	2.24	2.27	2.27	2.29	2.29	2.27	2.19	2.58	2.19	2.20	2.27	2.25	2.25	2.27	]
1.99	2.02	2.13	2.02	2.01	2.00	2.10	2.06	2.12	2.02	2.03	2.10	2.06	2.07	2.02	]
5.41	5.05	5.41	5.22	5.31	5.31	5.40	5.01	5.42	5.35	5.03	5.32	5.28	5.04	5.36	]
5.69	5.19	5.67	5.23	5.43	5.45	5.68	5.17	5.66	5.41	5.20	5.43	5.35	5.16	5.41	]
2.03	1.89	2.02	1.85	2.18	2.14	2.03	1.88	2.05	1.86	1.90	2.15	1.83	1.86	1.87	]

1H chem shifts: RMSD=0.18ppm (MAE=0.15) N=16 {-0.25 0.37}

m=1.000 b=0.00

**C-nom**	**iGau**	**Exp**	**Calc**	**diff**		**1**	**2**	**3**	**4**	**5**	**6**	**7**	**8**	**9**	**10**	**11**	**12**	**13**	**14**	**15**	**16**
C1-C**	18	135.50	136.32	0.82	[	136.57	136.63	136.71	136.73	136.55	136.77	136.61	136.74	136.70	134.65	134.63	134.35	134.16	136.53	134.64	136.49
C2-CH	26	190.60	189.68	-0.92	[	189.41	189.49	189.45	189.41	189.44	189.44	189.47	189.47	189.41	192.41	191.01	190.86	190.58	190.95	190.05	189.44
C3-C	10	173.80	173.25	-0.55	[	173.44	172.80	172.84	173.50	173.61	173.37	172.63	173.61	172.65	174.46	173.68	173.80	173.82	173.50	173.41	172.75
C4-C	11	130.90	132.50	1.60	[	133.60	133.56	133.52	131.41	130.68	133.60	133.07	130.75	133.04	133.05	132.95	130.29	130.24	133.30	133.53	131.23
C5-CH	12	146.80	144.60	-2.20	[	143.53	143.68	143.62	145.85	146.38	143.29	143.88	146.20	143.85	144.78	144.20	146.64	146.88	143.71	143.42	145.64
C6-CH	7	79.80	80.75	0.95	[	79.67	83.98	83.97	80.42	80.94	79.64	79.17	80.88	79.09	80.52	80.37	81.35	81.20	79.97	79.64	79.16
C7-CH	6	53.50	52.97	-0.53	[	52.35	52.44	52.41	48.70	55.07	52.55	54.93	54.99	54.94	51.18	53.27	55.39	54.55	51.43	52.39	51.80
C8-CH	1	80.10	80.91	0.81	[	84.41	79.97	79.88	76.91	79.50	84.28	81.37	79.48	81.17	79.65	84.79	79.27	79.18	83.97	84.10	80.13

C9-CH	5	147.40	144.78	-2.62	[	143.90	143.69	143.60	145.17	144.16	143.95	146.58	144.16	146.57	143.68	147.86	147.81	146.97	141.81	145.79	144.32
C10-C	4	134.60	135.79	1.19	[	136.61	136.81	136.86	136.29	136.16	136.54	133.92	136.04	133.96	133.98	134.50	134.36	134.93	136.52	135.63	136.56
C11-CH2	16	24.50	26.70	2.20	[	27.26	27.03	27.02	26.92	27.18	27.26	26.96	27.16	26.94	26.68	22.38	22.62	23.01	27.77	25.98	27.24
C12-CH2	17	23.40	25.35	1.95	[	25.48	25.35	26.04	25.56	25.63	25.44	25.39	25.59	25.93	23.10	24.92	25.23	25.39	23.57	24.21	25.88
C13-C**	19	156.80	159.18	2.38	[	158.98	158.85	158.71	158.69	158.98	158.78	158.83	158.79	158.68	161.37	162.94	163.02	163.16	159.53	158.72	158.85
C14-CH3	21	23.40	21.81	-1.59	[	21.69	21.66	21.67	21.69	21.69	21.69	21.64	21.68	21.68	22.96	22.60	22.65	22.72	21.88	21.82	21.74
C15-CH3	20	19.40	17.08	-2.32	[	17.03	17.04	17.08	17.07	17.04	17.07	17.06	17.08	17.07	17.65	17.25	17.25	17.29	16.76	16.85	16.96
C16-CH3	14	10.80	11.19	0.39	[	11.09	11.31	11.33	11.32	11.06	11.11	11.30	11.06	11.31	11.15	11.14	11.06	11.11	11.08	11.11	11.07
C17-C	8	137.80	140.72	2.92	[	140.15	140.19	140.35	137.83	141.77	139.95	141.56	141.73	141.32	142.95	141.15	142.16	142.06	141.12	140.19	141.17
C18-CH2	24	117.10	115.54	-1.56	[	114.25	114.24	114.08	117.29	116.83	114.43	116.90	116.79	117.20	113.30	113.93	116.54	116.45	113.75	114.16	114.70
C19-CH3	25	23.70	23.04	-0.66	[	25.65	25.61	25.68	25.69	19.31	25.64	19.31	19.33	19.33	24.12	25.45	18.93	19.26	25.65	25.60	24.09
C20-C	3	172.90	174.00	1.10	[	173.75	174.32	174.36	174.67	173.60	173.78	174.51	173.60	174.52	172.83	174.22	174.11	173.97	172.89	173.54	174.44

**17**	**18**	**19**	**20**	**21**	**22**	**23**	**24**	**25**	**26**	**27**	**28**	**29**	**30**	**31**	
136.50	134.64	136.80	136.54	136.48	136.49	136.88	134.92	133.88	135.11	135.04	136.90	134.08	134.09	136.91	]
189.35	190.06	189.30	190.94	189.42	189.37	189.24	189.97	190.72	189.97	190.00	189.27	189.11	189.10	189.33	]
172.66	173.60	173.46	173.68	172.48	172.45	173.44	172.58	173.94	173.28	173.48	172.69	172.85	172.66	173.41	]
131.19	130.61	133.49	130.47	130.86	130.86	133.33	133.03	133.48	133.76	130.86	133.24	133.36	132.83	133.59	]
145.63	146.31	143.69	146.42	146.66	146.77	143.88	143.76	144.27	143.11	146.02	143.63	143.80	143.96	143.43	]
79.14	80.79	79.68	81.18	77.86	77.87	79.72	79.05	80.10	79.55	80.71	84.11	83.77	79.12	79.84	]
51.68	54.83	51.69	54.09	53.43	53.35	51.52	54.95	51.84	52.67	55.20	53.22	52.37	54.75	52.59	]
80.19	79.23	79.54	78.96	84.56	84.57	79.45	81.07	79.73	84.06	79.22	78.30	79.60	80.94	83.92	]
144.25	146.00	145.80	142.09	144.02	143.99	146.13	148.52	145.51	146.00	146.25	147.07	146.26	149.17	143.92	]
136.45	135.09	134.38	135.98	136.39	136.29	134.20	132.98	134.43	135.72	135.20	133.97	135.53	132.74	137.37	]
27.09	25.94	27.16	27.72	27.27	27.08	27.08	25.65	23.81	25.74	25.68	27.11	22.47	22.56	27.46	]
25.44	24.22	24.95	23.60	25.83	25.38	25.40	24.08	25.91	24.28	24.30	25.43	24.19	24.33	25.17	]
158.82	158.74	158.53	159.41	158.88	158.88	158.40	158.14	160.54	158.12	158.35	158.46	161.96	161.96	156.94	]
21.66	21.85	21.65	21.88	21.74	21.66	21.69	21.78	23.58	21.88	21.90	21.70	22.77	22.76	21.78	]
17.08	16.90	16.96	16.72	17.04	17.08	17.04	16.93	17.47	17.12	17.14	17.04	17.14	17.13	16.98	]
11.03	11.08	11.26	11.06	11.07	11.04	11.23	11.33	11.18	11.12	11.08	11.19	11.28	11.27	11.09	]
141.12	141.58	141.40	141.86	141.25	141.10	141.16	141.38	141.97	139.75	141.90	141.02	139.95	141.77	140.35	]
114.58	116.86	114.28	117.08	118.26	118.36	114.60	116.96	113.93	114.51	116.56	118.35	114.19	116.69	114.21	]
24.08	19.22	24.07	19.13	19.73	19.75	24.11	19.24	24.24	25.64	19.29	19.85	25.54	19.29	25.58	]
174.51	173.41	173.65	172.78	173.70	173.71	173.77	174.38	173.32	173.81	173.59	173.52	174.88	175.04	172.14	]

13C chem shifts: RMSD=1.65ppm (MAE=1.46) N=20 {-2.62 2.92}

Fractions: 0.104 0.090 0.088 0.088 0.085 0.079 0.069 0.066 0.064 0.030 0.025 0.018 0.018 0.016 0.016 0.015 0.015 0.013 0.012 0.011 0.010 0.009 0.009 0.008 0.007 0.007 0.007 0.006 0.005 0.004 0.004

NMR parameters calculated for 6*R*,7*S*,8*S* diastereomer vs experimental data of kallopterolide B (**2**)

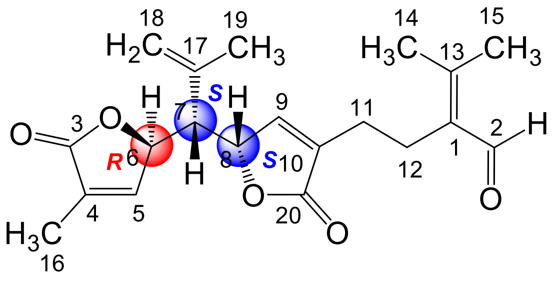

Parameter	RMSD
J_{HH}	0.59 Hz
δ_{1H}	0.20 ppm
δ_{13C}	1.49 ppm

	Conf1	Conf2	Conf3	Conf4	Conf5	Conf6	Conf7	Conf8	Conf9	Conf10	Conf11	Conf12	Conf13	Conf14	Conf15	Conf16
Rel energy (kcal/mol):	0.00	0.09	0.09	0.10	0.12	0.16	0.24	0.27	0.28	0.74	0.85	1.03	1.03	1.10	1.11	1.14

Conf17	Conf18	Conf19	Conf20	Conf21	Conf22	Conf23	Conf24	Conf25	Conf26	Conf27	Conf28	Conf29	Conf30	Conf31
1.15	1.25	1.26	1.35	1.37	1.42	1.43	1.55	1.58	1.59	1.64	1.68	1.81	1.89	1.93

iGau	jGau	Jexp	Jcalc	diff		1	2	3	4	5	6	7	8	9	10	11	12	13	14	15	16
22	30	1.65	2.01	0.36	[	2.05	1.96	1.96	2.03	2.03	2.05	1.99	2.03	1.99	2.02	2.05	2.05	2.03	2.03	2.05	1.94
30	31	1.50	1.52	0.02	[	-1.53	-1.52	-1.51	-1.52	-1.53	-1.53	-1.52	-1.53	-1.52	-1.51	-1.51	-1.51	-1.52	-1.52	-1.53	-1.52
22	29	7.00	6.06	-0.94	[	2.74	10.35	10.35	3.97	3.40	2.73	10.72	3.28	10.75	3.56	3.06	4.09	3.51	2.86	2.80	11.17
22	31	1.70	1.88	0.18	[	1.90	1.85	1.85	1.91	1.88	1.90	1.87	1.88	1.87	2.07	1.90	1.88	1.86	1.89	1.90	1.73
23	29	7.80	6.96	-0.84	[	10.36	2.72	2.76	1.72	10.71	10.33	3.37	10.84	3.37	10.70	9.35	9.96	10.56	10.03	10.28	3.78
23	28	1.40	1.99	0.59	[	1.94	2.02	2.03	2.05	1.96	1.94	1.99	1.94	2.00	1.98	2.04	2.04	2.00	1.97	1.92	2.04

17	18	19	20	21	22	23	24	25	26	27	28	29	30	31			
1.93	2.03	2.07	2.01	1.97	1.97	2.06	1.98	2.05	2.05	2.03	2.07	1.96	1.98	2.05	]	H6	H5
-1.52	-1.53	-1.53	-1.52	-1.53	-1.53	-1.53	-1.51	-1.53	-1.54	-1.53	-1.52	-1.51	-1.52	-1.53	]	H5	H16-Me
11.19	3.50	3.61	3.90	11.23	11.24	3.85	10.70	3.44	2.76	3.41	5.35	10.38	10.82	2.83	]	H6	H7
1.73	1.88	2.07	1.87	1.72	1.72	2.07	1.88	2.08	1.91	1.89	2.03	1.85	1.87	1.90	]	H6	H16-Me
3.80	10.73	11.18	10.25	5.21	5.33	11.23	3.43	10.96	10.30	10.75	11.26	2.72	3.30	10.14	]	H8	H7
2.03	1.93	1.90	2.00	2.04	2.03	1.91	1.96	2.08	1.97	1.98	1.96	2.09	2.07	1.98	]	H8	H9

For Js: RMSD=0.59Hz N=6 {-0.94 0.59}

H-nom	iGau	Exp	Calc	diff		1	2	3	4	5	6	7	8	9	10	11	12	13	14	15	16
H2	49	10.10	10.09	-0.01	[	10.10	10.09	10.09	10.09	10.10	10.10	10.09	10.10	10.09	10.14	9.95	9.96	9.96	10.00	10.13	10.10
H5	30	7.10	7.35	0.25	[	7.16	7.55	7.54	7.55	7.22	7.13	7.54	7.19	7.53	7.50	7.22	7.29	7.30	7.14	7.11	7.09
H6	22	5.05	5.10	0.05	[	5.04	5.26	5.27	4.94	5.07	4.99	5.40	5.02	5.42	4.80	5.27	5.24	5.14	4.76	4.91	5.00
H7	29	2.61	2.37	-0.24	[	2.14	2.12	2.13	2.90	2.48	2.08	2.48	2.45	2.49	2.66	1.80	2.20	2.34	2.21	2.06	2.63
H8	23	5.07	5.14	0.07	[	5.28	5.03	5.01	4.85	5.43	5.30	5.06	5.46	5.04	4.85	5.17	5.31	5.35	5.15	5.19	4.90
H9	28	7.13	7.39	0.26	[	7.60	7.22	7.22	7.20	7.60	7.62	7.28	7.60	7.28	6.58	7.80	7.77	7.71	7.16	7.36	7.56
H11	34	2.28	2.41	0.13	[	2.63	2.20	2.60	2.15	2.63	2.20	2.21	2.20	2.60	2.23	2.46	2.47	2.48	2.29	2.57	2.63

H11	35	2.28	2.46	0.18	[	2.25	2.57	2.17	2.57	2.25	2.64	2.58	2.64	2.17	2.57	2.63	2.63	2.63	2.72	2.69	2.26
H12	36	2.52	2.57	0.05	[	2.57	2.52	2.48	2.50	2.55	2.59	2.52	2.59	2.52	2.27	3.19	3.16	3.11	2.38	2.82	2.64
H12	37	2.52	2.56	0.04	[	2.53	2.54	2.51	2.48	2.52	2.56	2.53	2.56	2.55	2.97	2.27	2.26	2.28	3.07	2.75	2.57
H14-Me	41	2.03	2.31	0.28	[	2.35	2.33	2.33	2.31	2.34	2.35	2.33	2.34	2.33	2.24	2.32	2.32	2.33	2.22	1.94	2.37
H15-Me	38	2.21	2.28	0.07	[	2.28	2.27	2.27	2.27	2.28	2.28	2.27	2.28	2.27	2.59	2.27	2.27	2.27	2.27	2.23	2.30
H16-Me	31	1.95	2.05	0.10	[	2.03	2.08	2.07	2.10	2.04	2.02	2.08	2.03	2.07	2.10	2.03	2.05	2.04	2.01	2.01	1.99
H18a	44	4.83	5.35	0.52	[	5.41	5.38	5.41	5.42	5.21	5.42	5.18	5.20	5.26	5.61	5.36	5.16	5.16	5.37	5.39	5.70
H18b	45	5.08	5.26	0.18	[	5.38	5.35	5.38	5.43	5.07	5.38	5.05	5.04	5.12	5.35	5.30	5.04	5.02	5.30	5.34	5.47
H19-Me	46	1.81	1.91	0.10	[	1.88	1.84	1.91	1.91	1.92	1.86	1.90	1.93	1.93	2.09	1.83	1.82	1.87	1.94	1.85	2.03

**17**	**18**	**19**	**20**	**21**	**22**	**23**	**24**	**25**	**26**	**27**	**28**	**29**	**30**	**31**	
10.11	10.13	10.08	10.01	10.10	10.10	10.10	10.11	10.23	10.11	10.11	10.10	9.90	9.90	10.11	]
7.06	7.17	7.48	7.20	7.32	7.31	7.49	7.49	7.52	7.10	7.16	7.40	7.50	7.49	7.14	]
4.88	4.95	4.85	4.80	4.86	4.76	4.91	5.39	4.87	4.88	4.92	4.97	5.21	5.36	4.96	]
2.64	2.41	2.60	2.51	3.10	3.11	2.68	2.41	2.62	1.98	2.33	3.13	2.10	2.47	2.09	]
4.92	5.34	4.97	5.29	4.99	5.02	4.93	4.94	4.95	5.22	5.37	4.81	4.85	4.89	5.26	]
7.57	7.34	7.13	7.16	7.49	7.49	7.20	7.04	6.81	7.39	7.38	7.41	7.31	7.36	7.54	]
2.27	2.57	2.17	2.29	2.65	2.24	2.58	2.56	2.39	2.88	2.89	2.60	2.64	2.65	2.65	]
2.65	2.70	2.56	2.71	2.24	2.66	2.14	2.73	2.54	2.58	2.57	2.14	2.38	2.40	2.24	]
2.55	2.82	2.52	2.38	2.60	2.56	2.50	2.86	2.74	2.70	2.69	2.51	2.20	2.22	2.02	]
2.55	2.75	2.52	3.06	2.56	2.54	2.60	2.71	2.24	2.98	2.97	2.61	3.18	3.17	3.33	]
2.35	1.96	2.33	2.22	2.36	2.35	2.33	1.95	2.01	2.02	2.01	2.33	2.33	2.33	2.15	]
2.29	2.24	2.27	2.27	2.29	2.29	2.27	2.19	2.58	2.19	2.20	2.27	2.25	2.25	2.27	]
1.99	2.02	2.13	2.02	2.01	2.00	2.10	2.06	2.12	2.02	2.03	2.10	2.06	2.07	2.02	]
5.69	5.19	5.67	5.23	5.43	5.45	5.68	5.17	5.66	5.41	5.20	5.43	5.35	5.16	5.41	]
5.41	5.05	5.41	5.22	5.31	5.31	5.40	5.01	5.42	5.35	5.03	5.32	5.28	5.04	5.36	]
2.03	1.89	2.02	1.85	2.18	2.14	2.03	1.88	2.05	1.86	1.90	2.15	1.83	1.86	1.87	]

1H chem shifts: RMSD=0.20ppm (MAE=0.16) N=16 {-0.24 0.52}

m=1.000 b=0.00

**C-nom**	**iGau**	**Exp**	**Calc**	**diff**		**1**	**2**	**3**	**4**	**5**	**6**	**7**	**8**	**9**	**10**	**11**	**12**	**13**	**14**	**15**	**16**
C1-C**	18	135.40	136.32	0.92	[	136.57	136.63	136.71	136.73	136.55	136.77	136.61	136.74	136.70	134.65	134.63	134.35	134.16	136.53	134.64	136.49
C2-CH	26	190.70	189.68	-1.02	[	189.41	189.49	189.45	189.41	189.44	189.44	189.47	189.47	189.41	192.41	191.01	190.86	190.58	190.95	190.05	189.44
C3-C	10	173.50	173.25	-0.25	[	173.44	172.80	172.84	173.50	173.61	173.37	172.63	173.61	172.65	174.46	173.68	173.80	173.82	173.50	173.41	172.75
C4-C	11	130.90	132.50	1.60	[	133.60	133.56	133.52	131.41	130.68	133.60	133.07	130.75	133.04	133.05	132.95	130.29	130.24	133.30	133.53	131.23
C5-CH	12	147.00	144.60	-2.40	[	143.53	143.68	143.62	145.85	146.38	143.29	143.88	146.20	143.85	144.78	144.20	146.64	146.88	143.71	143.42	145.64
C6-CH	7	80.00	80.75	0.75	[	79.67	83.98	83.97	80.42	80.94	79.64	79.17	80.88	79.09	80.52	80.37	81.35	81.20	79.97	79.64	79.16
C7-CH	6	52.80	52.97	0.17	[	52.35	52.44	52.41	48.70	55.07	52.55	54.93	54.99	54.94	51.18	53.27	55.39	54.55	51.43	52.39	51.80
C8-CH	1	79.90	80.91	1.01	[	84.41	79.97	79.88	76.91	79.50	84.28	81.37	79.48	81.17	79.65	84.79	79.27	79.18	83.97	84.10	80.13
C9-CH	5	146.80	144.78	-2.02	[	143.90	143.69	143.60	145.17	144.16	143.95	146.58	144.16	146.57	143.68	147.86	147.81	146.97	141.81	145.79	144.32
C10-C	4	134.60	135.79	1.19	[	136.61	136.81	136.86	136.29	136.16	136.54	133.92	136.04	133.96	133.98	134.50	134.36	134.93	136.52	135.63	136.56
C11-CH2	16	24.60	26.70	2.10	[	27.26	27.03	27.02	26.92	27.18	27.26	26.96	27.16	26.94	26.68	22.38	22.62	23.01	27.77	25.98	27.24
C12-CH2	17	23.40	25.35	1.95	[	25.48	25.35	26.04	25.56	25.63	25.44	25.39	25.59	25.93	23.10	24.92	25.23	25.39	23.57	24.21	25.88
C13-C**	19	156.90	159.18	2.28	[	158.98	158.85	158.71	158.69	158.98	158.78	158.83	158.79	158.68	161.37	162.94	163.02	163.16	159.53	158.72	158.85
C14-CH3	21	23.40	21.81	-1.59	[	21.69	21.66	21.67	21.69	21.69	21.69	21.64	21.68	21.68	22.96	22.60	22.65	22.72	21.88	21.82	21.74

```
C15-CH3    20   19.40  17.08  -2.32   [  17.03  17.04  17.08  17.07  17.04  17.07  17.06  17.08  17.07  17.65  17.25  17.25  17.29  16.76  16.85  16.96
C16-CH3    14   10.70  11.19   0.49   [  11.09  11.31  11.33  11.32  11.06  11.11  11.30  11.06  11.31  11.15  11.14  11.06  11.11  11.08  11.11  11.07
C17-C       8  139.20 140.72   1.52   [ 140.15 140.19 140.35 137.83 141.77 139.95 141.56 141.73 141.32 142.95 141.15 142.16 142.06 141.12 140.19 141.17
C18-CH2    24  116.80 115.54  -1.26   [ 114.25 114.24 114.08 117.29 116.83 114.43 116.90 116.79 117.20 113.30 113.93 116.54 116.45 113.75 114.16 114.70
C19-CH3    25   23.60  23.04  -0.56   [  25.65  25.61  25.68  25.69  19.31  25.64  19.31  19.33  19.33  24.12  25.45  18.93  19.26  25.65  25.60  24.09
C20-C       3  173.00 174.00   1.00   [ 173.75 174.32 174.36 174.67 173.60 173.78 174.51 173.60 174.52 172.83 174.22 174.11 173.97 172.89 173.54 174.44

    17     18     19     20     21     22     23     24     25     26     27     28     29     30     31
136.50 134.64 136.80 136.54 136.48 136.49 136.88 134.92 133.88 135.11 135.04 136.90 134.08 134.09 136.91 ]
189.35 190.06 189.30 190.94 189.42 189.37 189.24 189.97 190.72 189.97 190.00 189.27 189.11 189.10 189.33 ]
172.66 173.60 173.46 173.68 172.48 172.45 173.44 172.58 173.94 173.28 173.48 172.69 172.85 172.66 173.41 ]
131.19 130.61 133.49 130.47 130.86 130.86 133.33 133.03 133.48 133.76 130.86 133.24 133.36 132.83 133.59 ]
145.63 146.31 143.69 146.42 146.66 146.77 143.88 143.76 144.27 143.11 146.02 143.63 143.80 143.96 143.43 ]
 79.14  80.79  79.68  81.18  77.86  77.87  79.72  79.05  80.10  79.55  80.71  84.11  83.77  79.12  79.84 ]
 51.68  54.83  51.69  54.09  53.43  53.35  51.52  54.95  51.84  52.67  55.20  53.22  52.37  54.75  52.59 ]
 80.19  79.23  79.54  78.96  84.56  84.57  79.45  81.07  79.73  84.06  79.22  78.30  79.60  80.94  83.92 ]
144.25 146.00 145.80 142.09 144.02 143.99 146.13 148.52 145.51 146.00 146.25 147.07 146.26 149.17 143.92 ]
136.45 135.09 134.38 135.98 136.39 136.29 134.20 132.98 134.43 135.72 135.20 133.97 135.53 132.74 137.37 ]
 27.09  25.94  27.16  27.72  27.27  27.08  27.08  25.65  23.81  25.74  25.68  27.11  22.47  22.56  27.46 ]
 25.44  24.22  24.95  23.60  25.83  25.38  25.40  24.08  25.91  24.28  24.30  25.43  24.19  24.33  25.17 ]
158.82 158.74 158.53 159.41 158.88 158.88 158.40 158.14 160.54 158.12 158.35 158.46 161.96 161.96 156.94 ]
 21.66  21.85  21.65  21.88  21.74  21.66  21.69  21.78  23.58  21.88  21.90  21.70  22.77  22.76  21.78 ]
 17.08  16.90  16.96  16.72  17.04  17.08  17.04  16.93  17.47  17.12  17.14  17.04  17.14  17.13  16.98 ]
 11.03  11.08  11.26  11.06  11.07  11.04  11.23  11.33  11.18  11.12  11.08  11.19  11.28  11.27  11.09 ]
141.12 141.58 141.40 141.86 141.25 141.10 141.16 141.38 141.97 139.75 141.90 141.02 139.95 141.77 140.35 ]
114.58 116.86 114.28 117.08 118.26 118.36 114.60 116.96 113.93 114.51 116.56 118.35 114.19 116.69 114.21 ]
 24.08  19.22  24.07  19.13  19.73  19.75  24.11  19.24  24.24  25.64  19.29  19.85  25.54  19.29  25.58 ]
174.51 173.41 173.65 172.78 173.70 173.71 173.77 174.38 173.32 173.81 173.59 173.52 174.88 175.04 172.14 ]

                  13C chem shifts: RMSD=1.49ppm (MAE=1.32) N=20 {-2.40 2.28}
                              Fractions:  0.104  0.090  0.088  0.088  0.085  0.079  0.069  0.066  0.064  0.030  0.025  0.018  0.018  0.016  0.016  0.015
0.015  0.013  0.012  0.011  0.010  0.009  0.009  0.008  0.007  0.007  0.007  0.006  0.005  0.004  0.004
```

Conformer 1

Energy: -1152.65932 Hartree (Rel: 0.0 kcal/mol)

XYZ coordinates for conf 1:

C	-0.98529	0.79949	-0.81010
O	-0.24419	2.00140	-0.49850
C	1.09621	1.73359	-0.50570
C	1.29471	0.30069	-0.84710
C	0.08121	-0.23821	-1.02590
C	-1.97619	0.53570	0.34970
C	-2.69069	-0.82840	0.17510
C	-2.93639	1.70769	0.55260
O	-3.41009	-0.87660	-1.07100
C	-4.72929	-1.17870	-0.83130
C	-4.91369	-1.35711	0.63240
C	-3.72579	-1.15160	1.21410
O	-5.53899	-1.27591	-1.72520
C	-6.24449	-1.70921	1.21260
O	1.92581	2.58730	-0.26970
C	2.66521	-0.29391	-0.93640
C	3.34081	-0.41591	0.45490
C	4.78461	-0.86931	0.36280
C	5.83821	-0.01131	0.26500
C	7.29221	-0.41651	0.19310
C	5.66011	1.48769	0.22540
H	-1.92929	-1.61820	0.13890
H	-1.54439	0.98950	-1.73260
C	-3.72759	2.16600	-0.42370
C	-2.92429	2.32340	1.92930
C	4.96741	-2.32981	0.33780
O	4.04021	-3.13201	0.37520
H	-0.13589	-1.26011	-1.31510
H	-1.36339	0.42500	1.25400
H	-3.49359	-1.21101	2.27170
H	-6.18859	-1.82031	2.29860
H	-6.98429	-0.93490	0.97750
H	-6.61849	-2.64550	0.78220
H	2.61821	-1.28161	-1.40530
H	3.28371	0.34689	-1.57720
H	3.26481	0.53989	0.97760
H	2.78451	-1.15421	1.04030
H	7.48661	-1.47791	0.34370
H	7.71041	-0.12821	-0.78050
H	7.86181	0.13989	0.94880
H	4.63361	1.81399	0.05340
H	6.00601	1.92859	1.17140
H	6.29371	1.91749	-0.56080
H	-4.40189	3.00069	-0.24940
H	-3.73659	1.74049	-1.42240
H	-3.15869	1.57990	2.70340
H	-1.92709	2.71950	2.16380
H	-3.64699	3.14070	2.01180
H	6.00231	-2.70971	0.27200

Conformer 2

Energy: -1152.65733 Hartree (Rel: 0.1 kcal/mol)

XYZ coordinates for conf 2:

C	-1.05490	-0.57931	0.05065
O	-0.58164	-0.21755	-1.26147
C	0.75208	0.09818	-1.19878
C	1.21402	-0.06901	0.20364
C	0.15897	-0.46466	0.92704
C	-2.26075	0.30100	0.46593
C	-3.43130	0.15948	-0.53757
C	-1.89288	1.76498	0.70677

O	-4.56732	0.86987	0.00210
C	-5.63682	0.02155	0.11102
C	-5.23069	-1.31933	-0.38595
C	-3.94900	-1.23397	-0.76504
O	-6.70753	0.38372	0.54628
C	-6.18920	-2.46463	-0.41490
O	1.37650	0.44076	-2.17976
C	2.64066	0.17545	0.58521
C	3.59245	-0.91147	0.02073
C	5.05345	-0.59076	0.26865
C	5.83186	0.10211	-0.60864
C	7.29576	0.42249	-0.41457
C	5.29924	0.63429	-1.91757
H	-3.16190	0.64026	-1.48420
H	-1.38779	-1.62367	-0.00422
C	-1.54922	2.59241	-0.28640
C	-1.95335	2.21942	2.14362
C	5.56799	-1.04738	1.57019
O	4.88323	-1.62762	2.40641
H	0.13743	-0.68769	1.98805
H	-2.61764	-0.12958	1.41078
H	-3.35023	-2.02472	-1.20251
H	-5.72107	-3.36678	-0.81716
H	-6.56165	-2.68200	0.59321
H	-7.06425	-2.21952	-1.02835
H	2.94460	1.15426	0.19368
H	2.73788	0.21216	1.67450
H	3.35873	-1.85902	0.51547
H	3.39769	-1.04394	-1.04555
H	7.76250	-0.04447	0.45213
H	7.85625	0.11210	-1.30597
H	7.43035	1.50906	-0.33017
H	4.21156	0.62026	-1.99887
H	5.64156	1.66612	-2.06713
H	5.71317	0.05087	-2.75260
H	-1.29092	3.62885	-0.08452
H	-1.51469	2.28185	-1.32605
H	-2.97139	2.10502	2.53941
H	-1.29586	1.61520	2.78378
H	-1.65982	3.26817	2.24751
H	6.62922	-0.84126	1.79612

Conformer 3
Energy: -1152.65751 Hartree (Rel: 0.1 kcal/mol)
XYZ coordinates for conf 3:

C	1.15950	-0.57881	-0.86540
O	0.67930	-1.58131	0.05180
C	-0.69260	-1.58450	0.05260
C	-1.17040	-0.55840	-0.91010
C	-0.08860	0.02540	-1.44130
C	2.11190	0.41369	-0.15210
C	3.32520	-0.32111	0.46850
C	1.40790	1.30909	0.86740
O	4.24430	0.67739	0.96310
C	5.47500	0.50099	0.38910
C	5.40880	-0.67051	-0.52350
C	4.15830	-1.14701	-0.47260
O	6.41550	1.22168	0.64090
C	6.60620	-1.13312	-1.28750
O	-1.33180	-2.34580	0.74630
C	-2.63220	-0.32330	-1.12830
C	-3.29650	0.38000	0.08470
C	-4.80340	0.47500	-0.05200
C	-5.66440	-0.46150	0.43510

C	-7.17130	-0.39499	0.34480
C	-5.18880	-1.70380	1.15010
H	2.99250	-0.91571	1.32620
H	1.72390	-1.10321	-1.64700
C	0.92500	0.84039	2.02330
C	1.30051	2.76399	0.48510
C	-5.27679	1.64820	-0.80470
O	-4.52879	2.48160	-1.30530
H	-0.06680	0.81200	-2.18750
H	2.51640	1.04659	-0.95270
H	3.77560	-2.01591	-0.99590
H	6.37440	-2.00611	-1.90320
H	6.98160	-0.33422	-1.93770
H	7.42290	-1.39302	-0.60370
H	-2.78740	0.28190	-2.02660
H	-3.12250	-1.29100	-1.29180
H	-3.02000	-0.14350	1.00230
H	-2.89039	1.39350	0.15370
H	-7.57240	0.54011	-0.04460
H	-7.54250	-1.21399	-0.28570
H	-7.60170	-0.55579	1.34180
H	-4.12450	-1.91070	1.03060
H	-5.40280	-1.61930	2.22540
H	-5.75060	-2.57680	0.79470
H	0.42701	1.50119	2.72830
H	1.00650	-0.20191	2.31600
H	2.29951	3.20009	0.35030
H	0.76921	2.88959	-0.46810
H	0.77201	3.34339	1.24790
H	-6.36820	1.76601	-0.92640

Conformer 4

Energy: -1152.65917 Hartree (Rel: 0.1 kcal/mol)

XYZ coordinates for conf 4:

C	-1.30580	0.33999	0.57160
O	-0.98280	-0.57831	-0.49100
C	0.36330	-0.53461	-0.74570
C	0.99220	0.43939	0.18260
C	0.01621	0.94589	0.94700
C	-2.38539	1.36600	0.15840
C	-3.79569	0.73460	0.01880
C	-2.03529	2.24520	-1.04110
O	-4.22680	0.26100	1.31350
C	-4.65380	-1.04150	1.21280
C	-4.47870	-1.48620	-0.19210
C	-3.96760	-0.45680	-0.88020
O	-5.08890	-1.65000	2.16490
C	-4.84700	-2.86770	-0.62630
O	0.87480	-1.21751	-1.60800
C	2.46850	0.68679	0.16500
C	3.26900	-0.51641	0.72930
C	4.76670	-0.34901	0.56360
C	5.46000	-0.80481	-0.51680
C	6.95330	-0.68262	-0.71340
C	4.78790	-1.51411	-1.66830
H	-4.48409	1.53810	-0.27450
H	-1.71020	-0.25500	1.40060
C	-1.77529	1.76269	-2.26260
C	-2.02849	3.72930	-0.76030
C	5.42440	0.39629	1.64950
O	4.82540	0.86909	2.60990
H	0.11681	1.68409	1.73520
H	-2.47849	2.02300	1.03280
H	-3.72870	-0.43360	-1.93580

H	-4.66400	-3.01050	-1.69440
H	-4.26800	-3.61470	-0.07050
H	-5.90490	-3.06920	-0.42060
H	2.78091	0.86849	-0.87090
H	2.70781	1.58419	0.74350
H	3.04660	-0.60211	1.79720
H	2.92470	-1.43581	0.25080
H	7.50470	-0.29652	0.14330
H	7.36790	-1.66772	-0.96370
H	7.16290	-0.03352	-1.57420
H	3.70210	-1.41121	-1.68630
H	5.18470	-1.13961	-2.62030
H	5.02960	-2.58641	-1.63680
H	-1.54569	2.43560	-3.08520
H	-1.76229	0.70259	-2.48820
H	-2.99739	4.06300	-0.36370
H	-1.27709	3.98049	0.00090
H	-1.80979	4.30980	-1.66130
H	6.51731	0.53828	1.57680

Conformer 5
Energy: -1152.65755 Hartree (Rel: 0.1 kcal/mol)
XYZ coordinates for conf 5:

C	-0.99040	0.77491	-0.72630
O	-0.25150	1.98241	-0.44150
C	1.09110	1.72411	-0.47950
C	1.29020	0.28971	-0.81390
C	0.07650	-0.25799	-0.96160
C	-1.95340	0.49271	0.45410
C	-2.72610	-0.82759	0.20390
C	-2.84490	1.68821	0.77830
O	-3.41410	-0.81159	-1.06490
C	-4.73500	-1.14599	-0.87660
C	-4.96540	-1.35979	0.57530
C	-3.79960	-1.15639	1.20130
O	-5.51420	-1.22629	-1.79890
C	-6.31300	-1.72869	1.10470
O	1.92000	2.58551	-0.27230
C	2.66200	-0.29829	-0.92470
C	3.35810	-0.41639	0.45680
C	4.80160	-0.86629	0.34540
C	5.85230	-0.00579	0.23820
C	7.30620	-0.40789	0.14960
C	5.67050	1.49291	0.20500
H	-1.99560	-1.64629	0.15730
H	-1.57090	0.95361	-1.63800
C	-2.94120	2.11181	2.04360
C	-3.58460	2.37721	-0.34440
C	4.98700	-2.32629	0.31350
O	4.06180	-3.13049	0.35910
H	-0.14110	-1.28379	-1.23620
H	-1.32780	0.29261	1.33330
H	-3.60510	-1.22679	2.26540
H	-6.29320	-1.85299	2.19050
H	-7.05150	-0.95819	0.85390
H	-6.66370	-2.66309	0.65080
H	2.61270	-1.28649	-1.39210
H	3.26790	0.34521	-1.57480
H	3.28680	0.53981	0.97950
H	2.81180	-1.15509	1.05110
H	7.50450	-1.46889	0.29720
H	7.71300	-0.11779	-0.82830
H	7.88290	0.14921	0.89950
H	4.64220	1.81721	0.03960

H	6.01990	1.93121	1.15100
H	6.29880	1.92731	-0.58280
H	-3.57780	2.95041	2.31450
H	-2.38710	1.64211	2.85300
H	-2.88340	2.85181	-1.04300
H	-4.19280	1.68131	-0.93200
H	-4.24000	3.15981	0.04890
H	6.02180	-2.70399	0.23430

Conformer 6
Energy: -1152.65918 Hartree (Rel: 0.2 kcal/mol)
XYZ coordinates for conf 6:

C	-0.92970	0.45809	-0.59620
O	-0.21710	1.71250	-0.69110
C	1.07790	1.54060	-0.28870
C	1.27690	0.11530	0.08330
C	0.10750	-0.51170	-0.10170
C	-2.15660	0.67789	0.32180
C	-2.87530	-0.66061	0.62780
C	-3.08950	1.75819	-0.22500
O	-3.31530	-1.29631	-0.58630
C	-4.66760	-1.53201	-0.52020
C	-5.16600	-1.05341	0.79560
C	-4.12030	-0.54681	1.46020
O	-5.27350	-2.05841	-1.42550
C	-6.60440	-1.18412	1.17680
O	1.87410	2.45630	-0.26920
C	2.61100	-0.38940	0.53690
C	3.64560	-0.42790	-0.61860
C	5.04250	-0.76929	-0.13800
C	5.96040	0.16651	0.23290
C	7.36910	-0.12549	0.69550
C	5.66100	1.64661	0.21150
H	-2.15800	-1.33961	1.10650
H	-1.26760	0.19540	-1.60430
C	-3.61630	1.69839	-1.45290
C	-3.37420	2.90419	0.71330
C	5.32650	-2.21129	-0.05400
O	4.50751	-3.08279	-0.32610
H	-0.09180	-1.56660	0.04860
H	-1.75610	1.01569	1.28670
H	-4.12020	-0.11711	2.45590
H	-6.78720	-0.79712	2.18260
H	-7.24090	-0.63842	0.47040
H	-6.91980	-2.23352	1.14110
H	2.98500	0.27370	1.32710
H	2.51010	-1.39050	0.96710
H	3.32910	-1.19360	-1.33320
H	3.63720	0.53020	-1.14250
H	7.67830	-1.16699	0.61420
H	8.07530	0.48101	0.11350
H	7.48820	0.18461	1.74220
H	4.60230	1.88941	0.11110
H	6.03620	2.11631	1.12950
H	6.20180	2.12341	-0.61870
H	-4.27970	2.48029	-1.81370
H	-3.41230	0.88499	-2.14240
H	-3.81890	2.55089	1.65380
H	-2.44391	3.42409	0.97900
H	-4.05851	3.63099	0.26560
H	6.33480	-2.51019	0.28350

Conformer 7
Energy: -1152.65686 Hartree (Rel: 0.2 kcal/mol)

XYZ coordinates for conf 7:

C	-1.03433	-0.56271	0.09506
O	-0.59208	-0.11387	-1.20514
C	0.74945	0.17530	-1.15884
C	1.23548	-0.04565	0.22731
C	0.19102	-0.45866	0.95675
C	-2.27428	0.22845	0.58336
C	-3.40661	0.14596	-0.47060
C	-1.97308	1.66411	1.00508
O	-4.56187	0.84427	0.03805
C	-5.64306	0.00280	0.05732
C	-5.21989	-1.32210	-0.46702
C	-3.91984	-1.23405	-0.77612
O	-6.73303	0.35939	0.44613
C	-6.18250	-2.45805	-0.58700
O	1.35729	0.55103	-2.13810
C	2.66966	0.18071	0.59237
C	3.60464	-0.89645	-0.01736
C	5.07158	-0.59534	0.21952
C	5.84488	0.11371	-0.64929
C	7.31414	0.41569	-0.46620
C	5.30000	0.68522	-1.93643
H	-3.09077	0.65702	-1.38704
H	-1.32375	-1.61605	-0.01738
C	-1.89714	1.96466	2.30632
C	-1.78383	2.71692	-0.06191
C	5.59905	-1.09149	1.50125
O	4.92031	-1.68863	2.33033
H	0.18548	-0.70875	2.01149
H	-2.62531	-0.31521	1.46968
H	-3.30377	-2.01375	-1.20937
H	-5.70077	-3.34628	-1.00394
H	-6.60230	-2.71394	0.39303
H	-7.02676	-2.18200	-1.22961
H	2.97452	1.16805	0.22372
H	2.78417	0.18588	1.68056
H	3.36955	-1.85631	0.45235
H	3.39439	-0.99591	-1.08446
H	7.78907	-0.08310	0.37799
H	7.85912	0.12985	-1.37525
H	7.45937	1.49787	-0.34860
H	4.21105	0.69069	-1.99952
H	5.65700	1.71448	-2.06806
H	5.68996	0.11453	-2.79160
H	-1.66890	2.97259	2.64366
H	-2.06335	1.21999	3.08123
H	-1.02016	2.43526	-0.79483
H	-2.71420	2.88027	-0.62099
H	-1.49521	3.67215	0.38619
H	6.66455	-0.89917	1.71913

Conformer 8

Energy: -1152.65643 Hartree (Rel: 0.3 kcal/mol)

XYZ coordinates for conf 8:

C	-0.93791	0.46070	-0.48500
O	-0.22781	1.71140	-0.61220
C	1.07809	1.54310	-0.24230
C	1.28229	0.12420	0.14990
C	0.10809	-0.50360	0.00180
C	-2.15131	0.67080	0.45510
C	-2.92051	-0.66330	0.63310
C	-3.02671	1.84470	0.02430
O	-3.32501	-1.21980	-0.63550
C	-4.67191	-1.49700	-0.60800

C	-5.21281	-1.08100	0.71160
C	-4.19511	-0.58130	1.42340
O	-5.24401	-1.99530	-1.55060
C	-6.65981	-1.24440	1.04630
O	1.87689	2.45610	-0.26260
C	2.62569	-0.37570	0.58100
C	3.62779	-0.44960	-0.60110
C	5.03599	-0.78640	-0.15140
C	5.97099	0.15340	0.16200
C	7.39209	-0.13500	0.58750
C	5.68029	1.63420	0.10560
H	-2.24031	-1.39150	1.09510
H	-1.29281	0.18270	-1.48370
C	-3.37951	2.76670	0.92700
C	-3.45921	1.94620	-1.41970
C	5.31229	-2.22720	-0.03000
O	4.47969	-3.10030	-0.25150
H	-0.08991	-1.55470	0.17730
H	-1.74831	0.90060	1.44970
H	-4.23141	-0.18270	2.43080
H	-6.87681	-0.89110	2.05770
H	-7.28521	-0.68700	0.33910
H	-6.95741	-2.29690	0.97050
H	3.02399	0.30570	1.34330
H	2.53199	-1.36490	1.03930
H	3.28759	-1.23160	-1.28660
H	3.60949	0.49470	-1.14930
H	7.69339	-1.18000	0.52230
H	8.08229	0.45280	-0.03160
H	7.54589	0.20040	1.62190
H	4.61999	1.88150	0.03780
H	6.09279	2.12940	0.99370
H	6.19189	2.08130	-0.75910
H	-4.01371	3.60850	0.66040
H	-3.04631	2.71920	1.96110
H	-2.59441	2.09830	-2.07830
H	-3.96561	1.03990	-1.76800
H	-4.13551	2.79440	-1.56050
H	6.32729	-2.52340	0.28930

Conformer 9

Energy: -1152.65625 Hartree (Rel: 0.3 kcal/mol)

XYZ coordinates for conf 9:

C	1.14040	-0.52801	-0.89830
O	0.67900	-1.51271	0.05310
C	-0.69340	-1.55011	0.04530
C	-1.19130	-0.53241	-0.91540
C	-0.12150	0.07089	-1.44950
C	2.13520	0.47040	-0.25320
C	3.31080	-0.29810	0.40100
C	1.48670	1.46829	0.70240
O	4.23830	0.65910	0.95280
C	5.48550	0.45780	0.42220
C	5.41740	-0.68700	-0.52350
C	4.15230	-1.12640	-0.53000
O	6.43730	1.14011	0.73040
C	6.62700	-1.16529	-1.25810
O	-1.31680	-2.32171	0.74220
C	-2.65780	-0.31481	-1.12270
C	-3.32340	0.37489	0.09710
C	-4.83200	0.45588	-0.03070
C	-5.68080	-0.49412	0.45170
C	-7.18880	-0.44232	0.36980
C	-5.18910	-1.73842	1.15210

H	2.93280	-0.90550	1.23100
H	1.67010	-1.07800	-1.68740
C	1.27899	2.72600	0.29720
C	1.10930	1.01299	2.09260
C	-5.32141	1.63178	-0.76900
O	-4.58481	2.47788	-1.26490
H	-0.11630	0.86429	-2.18810
H	2.55360	1.03610	-1.09530
H	3.76290	-1.96830	-1.09090
H	6.39110	-2.01359	-1.90600
H	7.04870	-0.36099	-1.87230
H	7.41100	-1.46889	-0.55430
H	-2.82590	0.29229	-2.01730
H	-3.13700	-1.28791	-1.28810
H	-3.03780	-0.15152	1.01050
H	-2.92691	1.39199	0.16950
H	-7.60150	0.49217	-0.00870
H	-7.55490	-1.25913	-0.26650
H	-7.61230	-0.61682	1.36740
H	-4.12260	-1.93152	1.02830
H	-5.40180	-1.66832	2.22870
H	-5.74120	-2.61412	0.78830
H	0.80519	3.45999	0.94420
H	1.57499	3.07039	-0.69090
H	0.46030	0.13069	2.08060
H	2.00080	0.74210	2.67270
H	0.59309	1.81209	2.63260
H	-6.41470	1.73958	-0.88340

Conformer 10
Energy: -1152.65713 Hartree (Rel: 0.7 kcal/mol)
XYZ coordinates for conf 10:

C	1.34475	-0.95993	-0.90874
O	1.16060	-2.39288	-0.89800
C	-0.15790	-2.69401	-1.12033
C	-0.89740	-1.42891	-1.35179
C	-0.01757	-0.42468	-1.25683
C	1.89350	-0.56554	0.48393
C	2.04718	0.95705	0.72679
C	3.22447	-1.24846	0.81279
O	0.75563	1.59607	0.85934
C	0.69558	2.68953	0.03878
C	1.97315	2.79313	-0.71205
C	2.74903	1.77215	-0.32191
O	-0.28590	3.40080	-0.02096
C	2.21603	3.89059	-1.69650
O	-0.56353	-3.83532	-1.13946
C	-2.37588	-1.40866	-1.58169
C	-3.18712	-1.48445	-0.25666
C	-2.94228	-0.31915	0.68494
C	-3.55421	0.89406	0.57730
C	-3.28606	2.08117	1.48026
C	-4.55214	1.20553	-0.50837
H	2.54996	1.07578	1.69548
H	2.07652	-0.73073	-1.69185
C	4.24039	-1.32740	-0.05320
C	3.32043	-1.80532	2.21132
C	-1.93746	-0.55304	1.73344
O	-1.30646	-1.59860	1.86353
H	-0.22888	0.62646	-1.39941
H	1.14490	-0.90729	1.20816
H	3.75432	1.54806	-0.65821
H	3.20646	3.80473	-2.15075
H	1.46213	3.87216	-2.49229

H	2.13752	4.86964	-1.20939
H	-2.64926	-0.50160	-2.13143
H	-2.65307	-2.26941	-2.20179
H	-4.24854	-1.56484	-0.51134
H	-2.90999	-2.40717	0.26054
H	-3.02848	1.81262	2.50626
H	-2.45823	2.67761	1.07296
H	-4.16875	2.72845	1.51783
H	-4.69727	0.39993	-1.22871
H	-5.52549	1.44715	-0.05955
H	-4.23736	2.10487	-1.05495
H	5.17766	-1.80213	0.22481
H	4.18218	-0.95147	-1.07119
H	3.09558	-1.03904	2.96597
H	2.58482	-2.60792	2.35306
H	4.31643	-2.20722	2.41917
H	-1.74499	0.27887	2.43204

Conformer 11

Energy: -1152.65716 Hartree (Rel: 0.8 kcal/mol)

XYZ coordinates for conf 11:

C	-0.42070	1.37291	-0.68330
O	0.52480	2.22310	0.00550
C	1.79830	1.90730	-0.38660
C	1.72770	0.83970	-1.41660
C	0.43390	0.54750	-1.60020
C	-1.21240	0.58271	0.39070
C	-1.99891	-0.59459	-0.23800
C	-2.06760	1.50471	1.25800
O	-2.88930	-0.12239	-1.26840
C	-4.17081	-0.53458	-0.99560
C	-4.15391	-1.32698	0.26110
C	-2.88931	-1.35649	0.69950
O	-5.10221	-0.26218	-1.71940
C	-5.40181	-1.93208	0.81700
O	2.77140	2.46539	0.07480
C	2.96000	0.23729	-2.02460
C	3.24029	-1.20381	-1.52150
C	3.29329	-1.35041	-0.00940
C	4.34179	-0.92651	0.75270
C	4.43319	-1.01131	2.25780
C	5.55149	-0.26232	0.14420
H	-1.27651	-1.27309	-0.70770
H	-1.10110	2.02311	-1.24220
C	-2.98460	2.32951	0.74040
C	-1.80820	1.42601	2.74170
C	2.11039	-1.97160	0.60680
O	1.10319	-2.31370	-0.00800
H	0.02229	-0.17570	-2.29400
H	-0.45481	0.10380	1.02330
H	-2.51171	-1.85839	1.58360
H	-5.19961	-2.48808	1.73620
H	-6.14381	-1.15458	1.03410
H	-5.85981	-2.61208	0.08910
H	2.86270	0.20779	-3.11650
H	3.80820	0.88919	-1.79380
H	2.44629	-1.85960	-1.88880
H	4.17589	-1.54911	-1.97640
H	3.54219	-1.38371	2.76160
H	5.27819	-1.65381	2.53890
H	4.65580	-0.01521	2.66200
H	5.68599	-0.48882	-0.91540
H	5.45400	0.82769	0.24450
H	6.46209	-0.55172	0.68130

H	-3.58419	2.97161	1.38070
H	-3.17530	2.40111	-0.32630
H	-1.96230	0.40661	3.12160
H	-0.76610	1.69151	2.96450
H	-2.46270	2.10021	3.30230
H	2.14869	-2.14230	1.69640

Conformer 12
Energy: -1152.65749 Hartree (Rel: 1.0 kcal/mol)
XYZ coordinates for conf 12:

C	-0.45210	1.20860	-0.70080
O	0.50959	2.10310	-0.10140
C	1.77069	1.77510	-0.52400
C	1.67130	0.63960	-1.47610
C	0.37410	0.32920	-1.59350
C	-1.20940	0.47039	0.43400
C	-2.09970	-0.65111	-0.15820
C	-1.93641	1.42949	1.37030
O	-2.93440	-0.16251	-1.23190
C	-4.24080	-0.52031	-0.99850
C	-4.30860	-1.23901	0.29970
C	-3.06480	-1.29771	0.79240
O	-5.12950	-0.25092	-1.77520
C	-5.60430	-1.75122	0.84000
O	2.75339	2.37571	-0.14410
C	2.88530	0.00141	-2.08330
C	3.25150	-1.35159	-1.41570
C	3.40100	-1.29479	0.09600
C	4.47820	-0.74569	0.72650
C	4.66220	-0.62509	2.22040
C	5.62350	-0.13369	-0.04060
H	-1.43350	-1.40961	-0.58910
H	-1.15301	1.81829	-1.28060
C	-1.73310	1.34229	2.68980
C	-2.86341	2.46689	0.78170
C	2.27590	-1.85300	0.86240
O	1.24381	-2.29380	0.36270
H	-0.05660	-0.43970	-2.22370
H	-0.44980	-0.06320	1.01740
H	-2.74770	-1.75481	1.72250
H	-5.46279	-2.25862	1.79790
H	-6.31700	-0.93012	0.98090
H	-6.06590	-2.45352	0.13600
H	2.71990	-0.17569	-3.15250
H	3.72150	0.70211	-1.99580
H	2.46170	-2.07300	-1.64240
H	4.17050	-1.72539	-1.88170
H	3.82050	-0.95979	2.82500
H	5.55100	-1.19159	2.52830
H	4.86160	0.42411	2.47460
H	5.70360	-0.49639	-1.06740
H	5.48780	0.95601	-0.07920
H	6.57510	-0.31978	0.47050
H	-2.24601	1.99869	3.38840
H	-1.04700	0.61660	3.12020
H	-2.31071	3.19069	0.16860
H	-3.62511	2.02279	0.13160
H	-3.37131	3.02529	1.57340
H	2.38370	-1.87460	1.96050

Conformer 13
Energy: -1152.65757 Hartree (Rel: 1.0 kcal/mol)
XYZ coordinates for conf 13:

C	-0.47459	1.19501	-0.57970

O	0.48141	1.97661	0.16810
C	1.74691	1.72401	-0.29030
C	1.65941	0.74891	-1.40770
C	0.36341	0.46491	-1.58940
C	-1.25179	0.28531	0.40690
C	-2.23729	-0.62340	-0.36990
C	-1.89059	1.07340	1.54610
O	-3.11509	0.14250	-1.22400
C	-4.42049	-0.21080	-0.97720
C	-4.43849	-1.22360	0.10910
C	-3.16899	-1.44770	0.47090
O	-5.34399	0.27630	-1.58980
C	-5.72049	-1.80250	0.61380
O	2.72291	2.26501	0.18420
C	2.88071	0.21391	-2.09370
C	3.32101	-1.17328	-1.55100
C	3.52721	-1.23528	-0.04640
C	4.60861	-0.70018	0.58850
C	4.84561	-0.69478	2.07970
C	5.70181	0.01482	-0.16500
H	-1.64849	-1.27859	-1.02510
H	-1.16529	1.89541	-1.06160
C	-1.69599	0.67761	2.80910
C	-2.71709	2.29500	1.21950
C	2.45061	-1.89309	0.71050
O	1.41912	-2.33469	0.21050
H	-0.05609	-0.19499	-2.33980
H	-0.51939	-0.40759	0.83900
H	-2.81358	-2.12890	1.23550
H	-5.54118	-2.53910	1.40120
H	-6.37039	-1.01490	1.01310
H	-6.27288	-2.28650	-0.20020
H	2.69161	0.11751	-3.16920
H	3.69201	0.93822	-1.96910
H	2.54942	-1.90379	-1.80910
H	4.23441	-1.47048	-2.07900
H	4.04321	-1.11648	2.68340
H	5.76911	-1.24338	2.30740
H	5.00901	0.33822	2.41340
H	5.75411	-0.25798	-1.22100
H	5.52711	1.09802	-0.10590
H	6.67851	-0.17728	0.29370
H	-2.14779	1.20511	3.64560
H	-1.07859	-0.18319	3.05470
H	-2.09659	3.08101	0.76970
H	-3.51689	2.07710	0.50360
H	-3.16959	2.70890	2.12540
H	2.59621	-1.99189	1.79990

Conformer 14

Energy: -1152.65887 Hartree (Rel: 1.1 kcal/mol)

XYZ coordinates for conf 14:

C	0.51608	-1.38895	-0.83374
O	-0.03778	-2.59768	-0.26879
C	-1.40262	-2.56463	-0.35145
C	-1.80605	-1.30824	-1.03627
C	-0.68654	-0.63860	-1.33715
C	1.33379	-0.68704	0.27972
C	1.79359	0.72434	-0.16024
C	2.47460	-1.56999	0.78453
O	2.59148	0.66198	-1.35812
C	3.80446	1.26734	-1.13681
C	3.83164	1.77381	0.25998
C	2.65995	1.45604	0.82414

O	4.65192	1.34332	-1.99765
C	5.02108	2.49645	0.80313
O	-2.09775	-3.46585	0.06398
C	-3.24888	-0.97956	-1.25954
C	-3.97246	-0.51866	0.03729
C	-3.53017	0.83891	0.55463
C	-4.05562	2.02026	0.12082
C	-3.66376	3.39428	0.61467
C	-5.12825	2.09167	-0.93821
H	0.90282	1.31985	-0.39885
H	1.18038	-1.68300	-1.65301
C	3.41751	-2.06123	-0.02727
C	2.46194	-1.85923	2.26460
C	-2.46391	0.80779	1.56950
O	-1.92133	-0.21882	1.96638
H	-0.61606	0.30313	-1.86951
H	0.62792	-0.51598	1.10263
H	2.33534	1.67820	1.83486
H	4.85744	2.81324	1.83645
H	5.90962	1.85498	0.77061
H	5.24711	3.38079	0.19584
H	-3.34061	-0.21041	-2.03398
H	-3.75823	-1.87961	-1.62535
H	-5.04911	-0.52282	-0.15418
H	-3.78594	-1.26438	0.81463
H	-2.93639	3.41045	1.42504
H	-3.25692	3.98357	-0.21785
H	-4.56004	3.92714	0.95784
H	-5.38795	1.12843	-1.37682
H	-6.04096	2.53427	-0.51594
H	-4.81034	2.76164	-1.74788
H	4.21851	-2.68550	0.36082
H	3.43123	-1.87049	-1.09603
H	2.49700	-0.93240	2.85349
H	1.53494	-2.37574	2.54670
H	3.31000	-2.48382	2.56124
H	-2.14044	1.77628	1.98882

Conformer 15

Energy: -1152.65671 Hartree (Rel: 1.1 kcal/mol)

XYZ coordinates for conf 15:

C	0.39580	0.64645	0.22256
O	-0.19000	1.94562	0.46204
C	-1.37513	2.05140	-0.21371
C	-1.62757	0.77775	-0.93741
C	-0.59713	-0.03789	-0.67484
C	1.81343	0.86228	-0.36066
C	2.44071	-0.47909	-0.81709
C	2.70434	1.65922	0.59196
O	2.51621	-1.40972	0.27889
C	3.81358	-1.83642	0.43368
C	4.65224	-1.18203	-0.60397
C	3.85003	-0.39228	-1.32833
O	4.13181	-2.62629	1.29327
C	6.11742	-1.45472	-0.70647
O	-2.05587	3.05432	-0.16702
C	-2.85177	0.56650	-1.77360
C	-4.17385	0.47545	-0.96294
C	-4.26807	-0.74444	-0.06613
C	-4.11345	-0.73113	1.28823
C	-4.22865	-1.93910	2.19141
C	-3.81343	0.52080	2.07592
H	1.78795	-0.92782	-1.57690
H	0.47224	0.13745	1.18943

C	2.93993	1.27033	1.84987
C	3.29777	2.92200	0.01940
C	-4.55825	-1.99466	-0.79158
O	-4.70586	-2.05532	-2.00772
H	-0.47465	-1.05621	-1.02588
H	1.67696	1.44399	-1.28168
H	4.12735	0.22734	-2.17405
H	6.56765	-0.90648	-1.53808
H	6.62835	-1.16710	0.21996
H	6.30210	-2.52575	-0.85020
H	-2.94732	1.41041	-2.46892
H	-2.73710	-0.34001	-2.37404
H	-4.31535	1.40150	-0.40401
H	-4.98539	0.41637	-1.69581
H	-4.50167	-2.86986	1.69654
H	-3.27629	-2.10039	2.71344
H	-4.97594	-1.74113	2.97087
H	-3.62957	1.40620	1.46872
H	-4.64758	0.73880	2.75725
H	-2.93505	0.35702	2.71408
H	3.58106	1.85583	2.50400
H	2.51080	0.36945	2.27749
H	3.89982	2.71209	-0.87546
H	2.50321	3.61422	-0.29005
H	3.93569	3.43329	0.74644
H	-4.64538	-2.92059	-0.19676

Conformer 16
Energy: -1152.65890 Hartree (Rel: 1.1 kcal/mol)
XYZ coordinates for conf 16:

C	-1.36889	-1.53021	-1.12460
O	-0.85689	-0.58670	-2.09250
C	0.48811	-0.40970	-1.89670
C	0.91291	-1.23340	-0.73540
C	-0.17429	-1.87040	-0.27860
C	-2.62269	-0.97101	-0.40300
C	-2.34009	0.22449	0.53600
C	-3.33448	-2.13481	0.29370
O	-3.58810	0.60669	1.15400
C	-3.85010	1.92748	0.90030
C	-2.73960	2.48119	0.08660
C	-1.86180	1.49179	-0.12220
O	-4.84570	2.47808	1.31650
C	-2.72501	3.91019	-0.34930
O	1.14691	0.32930	-2.59800
C	2.33251	-1.23579	-0.26050
C	2.72931	0.10801	0.40510
C	4.20031	0.16671	0.76730
C	5.16320	0.64792	-0.06740
C	6.63470	0.74762	0.26100
C	4.85120	1.15772	-1.45410
H	-1.65789	-0.07731	1.34000
H	-1.69338	-2.41161	-1.69250
C	-3.01118	-2.56301	1.51840
C	-4.41988	-2.79272	-0.52230
C	4.52611	-0.38859	2.09150
O	3.69271	-0.87839	2.84650
H	-0.22678	-2.56530	0.55090
H	-3.28119	-0.59461	-1.19540
H	-0.93100	1.56630	-0.66830
H	-1.82401	4.14249	-0.92310
H	-3.60141	4.13409	-0.96900
H	-2.77201	4.57909	0.51810
H	2.48941	-2.05259	0.45050

H	2.98981	-1.41559	-1.12040
H	2.45760	0.93301	-0.25700
H	2.14451	0.21861	1.32330
H	6.89820	0.50562	1.29010
H	7.20921	0.08732	-0.40210
H	6.98400	1.76802	0.05600
H	3.84870	0.91311	-1.80770
H	4.96870	2.25052	-1.48470
H	5.57640	0.75302	-2.17150
H	-3.52568	-3.40751	1.96920
H	-2.25049	-2.08741	2.13120
H	-5.24139	-2.08752	-0.70640
H	-4.05128	-3.10821	-1.50800
H	-4.82838	-3.67142	-0.01480
H	5.58481	-0.36488	2.40460

Conformer 17
Energy: -1152.65767 Hartree (Rel: 1.1 kcal/mol)
XYZ coordinates for conf 17:

C	-1.09950	1.12180	1.08430
O	-0.69450	-0.07910	1.77910
C	0.51810	-0.50180	1.30010
C	0.95070	0.42000	0.21820
C	-0.00220	1.35280	0.08340
C	-2.54881	0.99689	0.54670
C	-2.71910	-0.03121	-0.59550
C	-3.04791	2.39849	0.18240
O	-4.09910	-0.00041	-1.01960
C	-4.64580	-1.25311	-0.92320
C	-3.60550	-2.18501	-0.42200
C	-2.48590	-1.47481	-0.23420
O	-5.79710	-1.47821	-1.22530
C	-3.88940	-3.63701	-0.21450
O	1.06940	-1.49180	1.73360
C	2.25480	0.22720	-0.49090
C	3.46650	0.55720	0.42000
C	4.79360	0.22311	-0.23250
C	5.42190	-0.97719	-0.09100
C	6.75520	-1.34309	-0.70080
C	4.83790	-2.10579	0.72490
H	-2.10720	0.25739	-1.45880
H	-1.10391	1.92660	1.83090
C	-2.82071	2.97169	-1.00390
C	-3.79791	3.10859	1.28250
C	5.34199	1.29181	-1.08380
O	4.77729	2.36321	-1.27740
H	-0.00931	2.18780	-0.60690
H	-3.14980	0.63019	1.38790
H	-1.53560	-1.85731	0.11370
H	-3.00569	-4.16761	0.14940
H	-4.70150	-3.77211	0.50970
H	-4.21950	-4.10391	-1.15000
H	2.32740	-0.81820	-0.81580
H	2.29340	0.85470	-1.38650
H	3.44819	1.62990	0.63440
H	3.35680	0.03300	1.37180
H	7.28030	-0.52559	-1.19370
H	7.41410	-1.74249	0.08120
H	6.62060	-2.15159	-1.43160
H	3.79010	-1.96880	0.99550
H	4.92980	-3.05029	0.17370
H	5.41720	-2.23449	1.65050
H	-3.17221	3.97809	-1.21520
H	-2.30521	2.46759	-1.81670

H	-4.73111	2.57789	1.51400
H	-3.21671	3.14069	2.21430
H	-4.04711	4.13589	1.00150
H	6.31140	1.10001	-1.57700

Conformer 18
Energy: -1152.65665 Hartree (Rel: 1.2 kcal/mol)
XYZ coordinates for conf 18:

C	0.41441	0.67380	0.15860
O	-0.17699	1.95080	0.47830
C	-1.38649	2.07300	-0.15130
C	-1.64349	0.83510	-0.93300
C	-0.59559	0.02160	-0.74350
C	1.81511	0.91340	-0.45810
C	2.45910	-0.44130	-0.84950
C	2.69681	1.79460	0.42190
O	2.49270	-1.35830	0.26490
C	3.77020	-1.84281	0.42400
C	4.64710	-1.20371	-0.59050
C	3.88470	-0.37740	-1.31740
O	4.04680	-2.65300	1.27920
C	6.10610	-1.51691	-0.66830
O	-2.08029	3.06000	-0.02750
C	-2.88909	0.65011	-1.74370
C	-4.18539	0.49651	-0.90150
C	-4.23440	-0.76749	-0.06420
C	-4.04460	-0.81719	1.28480
C	-4.11470	-2.06959	2.13030
C	-3.74689	0.40081	2.12490
H	1.83190	-0.90630	-1.62190
H	0.51970	0.11760	1.09680
C	3.32271	2.84590	-0.11880
C	2.82851	1.46590	1.89050
C	-4.52120	-1.98619	-0.84260
O	-4.70000	-1.99049	-2.05610
H	-0.46930	-0.97430	-1.15280
H	1.65901	1.43870	-1.40880
H	4.19961	0.24999	-2.14330
H	6.58840	-0.96511	-1.47940
H	6.60690	-1.26341	0.27340
H	6.26280	-2.58961	-0.83150
H	-3.01639	1.52771	-2.39050
H	-2.78019	-0.22179	-2.39430
H	-4.32529	1.39121	-0.29320
H	-5.01679	0.46001	-1.61320
H	-4.39730	-2.97779	1.60010
H	-3.14190	-2.24569	2.60820
H	-4.83410	-1.91889	2.94560
H	-3.57629	1.31401	1.55630
H	-4.57649	0.58121	2.82270
H	-2.86130	0.21601	2.74720
H	3.97061	3.48699	0.47360
H	3.20941	3.10810	-1.16820
H	1.86581	1.57760	2.40580
H	3.15901	0.43530	2.05900
H	3.54241	2.13919	2.37370
H	-4.57540	-2.94149	-0.29200

Conformer 19
Energy: -1152.65813 Hartree (Rel: 1.3 kcal/mol)
XYZ coordinates for conf 19:

C	1.40960	-0.81690	-0.51981
O	0.65250	-2.03960	-0.66311
C	-0.65090	-1.82450	-0.30881

C	-0.80860	-0.40160	0.08179
C	0.39010	0.18340	-0.04231
C	2.59280	-1.10140	0.43329
C	3.53370	0.10959	0.66409
C	3.44600	-2.29780	0.00139
O	2.87720	1.14339	1.42809
C	3.02640	2.35240	0.79389
C	3.77810	2.13429	-0.46941
C	4.05570	0.82609	-0.55001
O	2.57580	3.37870	1.25149
C	4.09870	3.26509	-1.39151
O	-1.47940	-2.71090	-0.34151
C	-2.13700	0.14930	0.49849
C	-3.13820	0.22030	-0.68451
C	-4.53320	0.61931	-0.24431
C	-5.48810	-0.27569	0.13379
C	-6.89510	0.07361	0.56059
C	-5.23710	-1.76449	0.15889
H	4.36980	-0.24921	1.27849
H	1.78130	-0.54730	-1.51571
C	3.79610	-2.52791	-1.26831
C	3.89620	-3.19381	1.12909
C	-4.77460	2.07161	-0.21111
O	-3.92889	2.90930	-0.50581
H	0.61950	1.22220	0.15449
H	2.15530	-1.33140	1.41259
H	4.60410	0.31899	-1.33491
H	4.65700	2.91999	-2.26551
H	3.18111	3.75829	-1.73301
H	4.69291	4.02739	-0.87391
H	-2.55480	-0.49750	1.28029
H	-2.01200	1.14690	0.93059
H	-2.77400	0.96850	-1.39481
H	-3.15320	-0.74120	-1.20201
H	-7.16690	1.12291	0.45129
H	-7.61000	-0.52119	-0.02301
H	-7.04550	-0.20939	1.61099
H	-4.18830	-2.04569	0.05599
H	-5.61690	-2.19069	1.09629
H	-5.80320	-2.25069	-0.64881
H	4.42320	-3.37581	-1.53051
H	3.46080	-1.91000	-2.09671
H	4.40480	-2.62581	1.92009
H	3.03139	-3.68011	1.60009
H	4.57869	-3.97251	0.77659
H	-5.77640	2.41081	0.10709

Conformer 20

Energy: -1152.65704 Hartree (Rel: 1.4 kcal/mol)

XYZ coordinates for conf 20:

C	-0.53720	1.34230	-0.76670
O	0.01770	2.57290	-0.25800
C	1.38020	2.55380	-0.38440
C	1.77680	1.28110	-1.04260
C	0.65690	0.58950	-1.28620
C	-1.31170	0.64920	0.38440
C	-1.82350	-0.73910	-0.07320
C	-2.38900	1.54750	0.98440
O	-2.56530	-0.66070	-1.31050
C	-3.77870	-1.28890	-1.16040
C	-3.89030	-1.77230	0.24000
C	-2.75830	-1.43750	0.87200
O	-4.56910	-1.38520	-2.07210

C	-5.11050	-2.48710	0.72300
O	2.07670	3.47570	-0.02050
C	3.21600	0.96090	-1.29950
C	3.97940	0.53680	-0.01300
C	3.56760	-0.81420	0.54520
C	4.09040	-1.99950	0.11900
C	3.72860	-3.36640	0.65410
C	5.12670	-2.08410	-0.97460
H	-0.94890	-1.37290	-0.27260
H	-1.22740	1.60180	-1.57660
C	-2.42020	1.74130	2.30780
C	-3.38630	2.21290	0.06530
C	2.53280	-0.77180	1.59210
O	1.99170	0.25770	1.98330
H	0.58020	-0.36960	-1.78570
H	-0.57390	0.43830	1.16850
H	-2.50140	-1.63370	1.90660
H	-5.01420	-2.77330	1.77350
H	-5.99880	-1.85390	0.61310
H	-5.29050	-3.39030	0.12800
H	3.29370	0.17480	-2.05820
H	3.70590	1.85690	-1.70000
H	5.05050	0.54930	-0.23340
H	3.80460	1.29680	0.75310
H	2.99910	-3.37450	1.46260
H	3.33810	-3.99040	-0.16060
H	4.63600	-3.86740	1.01590
H	5.40240	-1.12130	-1.40430
H	6.03740	-2.56410	-0.59150
H	4.76060	-2.72750	-1.78600
H	-3.18060	2.36430	2.77240
H	-1.68500	1.29470	2.97280
H	-2.88980	2.92660	-0.60500
H	-3.90950	1.49350	-0.57400
H	-4.13330	2.76640	0.64180
H	2.23310	-1.73400	2.04230

Conformer 21
Energy: -1152.65706 Hartree (Rel: 1.4 kcal/mol)
XYZ coordinates for conf 21:

C	1.29799	-1.32841	1.42305
O	0.81397	-0.18605	2.16547
C	-0.52054	-0.00127	1.91643
C	-0.97597	-1.04677	0.96531
C	0.08782	-1.80666	0.66916
C	2.57893	-0.95682	0.62274
C	2.35641	0.14874	-0.43856
C	3.20860	-2.22695	0.04101
O	3.57673	0.29381	-1.19901
C	4.01478	1.59171	-1.14221
C	3.06005	2.37352	-0.31894
C	2.09672	1.53656	0.08700
O	5.02194	1.95630	-1.70732
C	3.25550	3.83382	-0.07033
O	-1.14898	0.90155	2.42854
C	-2.39765	-1.11102	0.50152
C	-2.75696	0.05994	-0.45062
C	-4.23070	0.08867	-0.80548
C	-5.16164	0.79071	-0.10116
C	-6.63436	0.86494	-0.43114
C	-4.80862	1.60375	1.12134
H	1.57279	-0.15773	-1.14311
H	1.60538	-2.07878	2.16125
C	4.10043	-2.90166	0.77529

C	2.80619	-2.69760	-1.33801
C	-4.59821	-0.75082	-1.95779
O	-3.79582	-1.43838	-2.58065
H	0.09904	-2.67862	0.02663
H	3.27468	-0.54952	1.36480
H	1.24132	1.78903	0.69905
H	2.45453	4.24241	0.55119
H	4.21412	4.01566	0.42982
H	3.28285	4.38690	-1.01663
H	-2.58517	-2.06180	-0.00655
H	-3.05435	-1.07021	1.37951
H	-2.44661	1.00322	0.00376
H	-2.18252	-0.06335	-1.37372
H	-6.92341	0.39951	-1.37278
H	-7.21905	0.39867	0.37307
H	-6.94567	1.91704	-0.46674
H	-3.81293	1.40593	1.52046
H	-4.87784	2.67597	0.88784
H	-5.54078	1.41710	1.91711
H	4.54799	-3.82545	0.41781
H	4.42159	-2.55410	1.75470
H	1.72063	-2.80222	-1.45280
H	3.13805	-1.98641	-2.10288
H	3.26014	-3.66788	-1.55909
H	-5.66018	-0.75946	-2.26057

Conformer 22
Energy: -1152.65731 Hartree (Rel: 1.4 kcal/mol)
XYZ coordinates for conf 22:

C	-1.06050	1.03700	1.21080
O	-0.67210	-0.24600	1.75230
C	0.52750	-0.63320	1.21540
C	0.98510	0.41510	0.26830
C	0.05250	1.37780	0.25870
C	-2.51650	0.98211	0.66610
C	-2.71660	-0.03569	-0.48400
C	-2.98149	2.39661	0.30370
O	-4.05830	0.12151	-0.99700
C	-4.72850	-1.07299	-0.93520
C	-3.81650	-2.09369	-0.36360
C	-2.64810	-1.49239	-0.10510
O	-5.87260	-1.18909	-1.31490
C	-4.25160	-3.50989	-0.16950
O	1.05220	-1.68630	1.51170
C	2.28920	0.29300	-0.45630
C	3.50390	0.46120	0.49410
C	4.82500	0.18369	-0.19630
C	5.41830	-1.04221	-0.22210
C	6.74360	-1.35851	-0.87550
C	4.79840	-2.25530	0.42920
H	-2.02850	0.18871	-1.30910
H	-1.07350	1.74460	2.04840
C	-3.56399	3.14701	1.24570
C	-2.76499	2.92061	-1.09760
C	5.40790	1.34379	-0.89040
O	4.87461	2.44749	-0.93500
H	0.08461	2.30200	-0.30510
H	-3.12720	0.63531	1.50730
H	-1.76360	-1.95579	0.31080
H	-3.44791	-4.11889	0.25240
H	-5.11740	-3.55969	0.50130
H	-4.56440	-3.95239	-1.12270

H	2.33830	-0.69780	-0.92510
H	2.34790	1.03870	-1.25490
H	3.51330	1.49410	0.85470
H	3.37520	-0.18620	1.36410
H	7.29610	-0.49591	-1.24650
H	7.38540	-1.88511	-0.15730
H	6.58880	-2.05061	-1.71410
H	3.75160	-2.12940	0.70890
H	4.87280	-3.11881	-0.24390
H	5.36460	-2.52261	1.33310
H	-3.87849	4.16781	1.04460
H	-3.75389	2.76931	2.24790
H	-1.72269	2.83531	-1.42780
H	-3.37259	2.36261	-1.81900
H	-3.04919	3.97501	-1.15990
H	6.37450	1.19439	-1.40340

Conformer 23
Energy: -1152.65679 Hartree (Rel: 1.4 kcal/mol)
XYZ coordinates for conf 23:

C	1.44442	-0.89691	-0.64296
O	0.64824	-2.09795	-0.75465
C	-0.67854	-1.77272	-0.81773
C	-0.81026	-0.29627	-0.75277
C	0.42517	0.21228	-0.65381
C	2.31318	-1.01099	0.63129
C	3.29373	0.17347	0.83731
C	3.11126	-2.31579	0.71160
O	2.58340	1.39915	1.11500
C	3.03553	2.39451	0.28344
C	4.06965	1.82411	-0.61828
C	4.20326	0.52822	-0.30600
O	2.59823	3.52234	0.33279
C	4.75737	2.66265	-1.64576
O	-1.54276	-2.61892	-0.92050
C	-2.15226	0.36621	-0.79218
C	-2.96305	0.12346	0.50820
C	-4.38919	0.62918	0.40946
C	-5.43036	-0.13598	-0.02184
C	-6.86933	0.31661	-0.11421
C	-5.25355	-1.57046	-0.45974
H	3.88585	-0.05470	1.73327
H	2.08279	-0.84900	-1.53350
C	3.73831	-2.85658	-0.33807
C	3.16441	-2.94217	2.08342
C	-4.56586	2.04461	0.77483
O	-3.64609	2.77779	1.12143
H	0.68507	1.26097	-0.59780
H	1.62327	-0.98187	1.48353
H	4.87854	-0.18885	-0.75710
H	5.48085	2.07691	-2.21863
H	4.02951	3.09852	-2.34012
H	5.28157	3.50055	-1.17123
H	-2.03765	1.44252	-0.95280
H	-2.71583	-0.03679	-1.64312
H	-2.94482	-0.94120	0.75108
H	-2.46364	0.65376	1.32453
H	-7.07470	1.30039	0.30645
H	-7.19093	0.32113	-1.16430
H	-7.51208	-0.40941	0.40059
H	-4.21642	-1.86530	-0.62511
H	-5.68558	-2.24545	0.29322
H	-5.81376	-1.74901	-1.38634
H	4.31315	-3.77309	-0.23497

H	3.68875	-2.43588	-1.33858
H	3.51880	-2.23035	2.84141
H	2.16147	-3.26035	2.39817
H	3.82325	-3.81515	2.10192
H	-5.58880	2.45772	0.71972

Conformer 24
Energy: -1152.65768 Hartree (Rel: 1.5 kcal/mol)
XYZ coordinates for conf 24:

C	-0.47060	0.03410	-0.05090
O	-0.20850	-0.11980	1.36090
C	1.02660	-0.69510	1.53930
C	1.61570	-0.98030	0.20570
C	0.73470	-0.56630	-0.71490
C	-1.83770	-0.57750	-0.45080
C	-2.96520	0.02620	0.42350
C	-1.86110	-2.10350	-0.46150
O	-4.22770	-0.52021	-0.01010
C	-5.08071	0.49539	-0.35430
C	-4.38321	1.79079	-0.14140
C	-3.15481	1.51400	0.31460
O	-6.20561	0.28859	-0.75190
C	-5.05841	3.09510	-0.41460
O	1.48290	-0.89780	2.64360
C	2.95040	-1.64230	0.04810
C	4.15740	-0.78590	0.51840
C	4.39229	0.46760	-0.30350
C	4.11249	1.73560	0.11130
C	4.37639	2.99340	-0.68700
C	3.50789	2.05290	1.45760
H	-2.81820	-0.27340	1.46730
H	-0.51040	1.11380	-0.24800
C	-1.80830	-2.75310	-1.62960
C	-1.96090	-2.84430	0.85130
C	4.98499	0.20980	-1.62880
O	5.26600	-0.91050	-2.04100
H	0.83680	-0.62820	-1.79230
H	-2.01330	-0.23240	-1.47760
H	-2.39361	2.23150	0.59900
H	-4.40021	3.93929	-0.19320
H	-5.36801	3.15739	-1.46450
H	-5.96861	3.19189	0.18880
H	2.95120	-2.56710	0.63940
H	3.09760	-1.92550	-0.99740
H	4.04360	-0.55790	1.57890
H	5.04480	-1.42060	0.42190
H	4.88339	2.84650	-1.63950
H	3.42829	3.51120	-0.88410
H	4.98349	3.68400	-0.08730
H	3.17100	1.18330	2.02050
H	4.23819	2.59710	2.07280
H	2.65289	2.73000	1.33160
H	-1.80710	-3.83920	-1.67550
H	-1.76820	-2.22770	-2.58090
H	-1.17610	-2.55090	1.55670
H	-2.92080	-2.64230	1.34390
H	-1.89230	-3.92410	0.69060
H	5.18149	1.08150	-2.27730

Conformer 25
Energy: -1152.65679 Hartree (Rel: 1.6 kcal/mol)
XYZ coordinates for conf 25:

C	1.32530	-1.06249	-0.78270
O	1.17670	-2.49499	-0.67940

C	-0.14309	-2.83689	-0.81050
C	-0.93510	-1.60140	-1.04210
C	-0.07930	-0.56919	-1.01410
C	2.05150	-0.58799	0.49650
C	2.25459	0.94342	0.60750
C	3.40780	-1.27288	0.69680
O	1.00079	1.61651	0.86040
C	0.85039	2.65821	-0.01780
C	2.01619	2.67561	-0.93820
C	2.82199	1.66572	-0.58170
O	-0.11422	3.39191	0.01270
C	2.14158	3.69591	-2.02230
O	-0.51689	-3.98789	-0.74990
C	-2.41480	-1.66500	-1.27360
C	-3.28370	-1.43370	-0.00460
C	-3.34620	0.01329	0.44530
C	-2.69391	0.53630	1.52220
C	-2.77191	1.98290	1.96330
C	-1.77850	-0.27010	2.40820
H	2.88319	1.11912	1.49030
H	1.94660	-0.86179	-1.66400
C	4.30060	-1.42858	-0.28620
C	3.68270	-1.73408	2.10640
C	-4.20711	0.86129	-0.39830
O	-4.81771	0.44979	-1.37990
H	-0.32400	0.47181	-1.17870
H	1.40540	-0.86379	1.33850
H	3.77079	1.39572	-1.02950
H	3.06078	3.55452	-2.59630
H	1.28768	3.64041	-2.70750
H	2.14318	4.70782	-1.60050
H	-2.71070	-0.93850	-2.03540
H	-2.64659	-2.66190	-1.66500
H	-4.30150	-1.74441	-0.26320
H	-2.94510	-2.09540	0.79590
H	-1.88561	2.52330	1.60440
H	-3.65541	2.51949	1.61700
H	-2.75901	2.03440	3.05830
H	-1.58270	-1.28060	2.05010
H	-0.81910	0.25340	2.50630
H	-2.20300	-0.33680	3.41960
H	5.26210	-1.89887	-0.09860
H	4.11390	-1.12328	-1.31220
H	3.56690	-0.91418	2.82860
H	2.96760	-2.51418	2.39980
H	4.69410	-2.13778	2.20890
H	-4.29311	1.92679	-0.12230

Conformer 26
Energy: -1152.65916 Hartree (Rel: 1.6 kcal/mol)
XYZ coordinates for conf 26:

C	-0.47791	1.29081	-0.78510
O	0.24459	2.37391	-0.15780
C	1.58419	2.23971	-0.40080
C	1.79959	1.02861	-1.23550
C	0.59639	0.48041	-1.45430
C	-1.30571	0.57211	0.30760
C	-1.96661	-0.71529	-0.24730
C	-2.29431	1.51801	0.98880
O	-2.82681	-0.41209	-1.36080
C	-4.09111	-0.89019	-1.11120
C	-4.08951	-1.55709	0.21700
C	-2.85201	-1.45229	0.71640
O	-4.99641	-0.75729	-1.90260

C	-5.32241	-2.19469	0.76900
O	2.39849	3.02771	0.03350
C	3.15519	0.62181	-1.72680
C	4.15679	0.20821	-0.61480
C	3.76999	-1.04349	0.15100
C	3.34179	-1.06149	1.44500
C	3.00509	-2.30679	2.23570
C	3.17609	0.18601	2.27970
H	-1.17641	-1.37879	-0.62170
H	-1.15471	1.73211	-1.52470
C	-3.21211	2.21131	0.30620
C	-2.16341	1.62121	2.48770
C	3.92799	-2.29079	-0.61910
O	4.33799	-2.32929	-1.77420
H	0.38799	-0.39609	-2.05730
H	-0.58241	0.22371	1.05670
H	-2.49231	-1.83519	1.66530
H	-5.13391	-2.64839	1.74540
H	-6.12391	-1.45419	0.87550
H	-5.69541	-2.96859	0.08790
H	3.05369	-0.19869	-2.44170
H	3.59629	1.47071	-2.26560
H	5.11119	0.01151	-1.11570
H	4.31739	1.05461	0.05360
H	3.20129	-3.25109	1.73010
H	3.57479	-2.30499	3.17410
H	1.94509	-2.28839	2.52210
H	3.19309	1.11631	1.71300
H	2.23099	0.14151	2.83610
H	3.97229	0.23221	3.03640
H	-3.90521	2.87391	0.81840
H	-3.31031	2.15401	-0.77340
H	-2.27761	0.63961	2.96800
H	-1.16731	1.99401	2.76220
H	-2.91191	2.29671	2.91230
H	3.65699	-3.23639	-0.11750

Conformer 27

Energy: -1152.65906 Hartree (Rel: 1.6 kcal/mol)

XYZ coordinates for conf 27:

C	-0.49070	1.21671	-0.72210
O	0.22490	2.31841	-0.12470
C	1.55970	2.21471	-0.41000
C	1.77670	0.99941	-1.23780
C	0.58000	0.42321	-1.41660
C	-1.27580	0.47191	0.38620
C	-2.02700	-0.74069	-0.22140
C	-2.15970	1.40851	1.20500
O	-2.87630	-0.34769	-1.31990
C	-4.14750	-0.83200	-1.11530
C	-4.17660	-1.54590	0.18730
C	-2.94680	-1.47519	0.71160
O	-5.03760	-0.65650	-1.91580
C	-5.42840	-2.17990	0.70050
O	2.36720	3.02801	-0.01220
C	3.12660	0.61431	-1.76110
C	4.16400	0.23051	-0.67150
C	3.81670	-1.01629	0.12090
C	3.42300	-1.02289	1.42610
C	3.12530	-2.26199	2.24160
C	3.25940	0.23291	2.24860
H	-1.27990	-1.43299	-0.63220
H	-1.19570	1.63791	-1.44750
C	-2.10470	1.36421	2.54080

C	-3.06620	2.37761	0.48280
C	3.97170	-2.27139	-0.63650
O	4.34930	-2.31989	-1.80240
H	0.37330	-0.46499	-2.00280
H	-0.53200	0.03331	1.06390
H	-2.61150	-1.88839	1.65610
H	-5.26220	-2.66440	1.66600
H	-6.22140	-1.43170	0.81600
H	-5.80070	-2.92870	-0.00860
H	3.02260	-0.21499	-2.46540
H	3.53740	1.46611	-2.31910
H	5.10760	0.04161	-1.19530
H	4.32940	1.08791	-0.01850
H	3.32160	-3.21029	1.74360
H	3.71930	-2.23939	3.16460
H	2.07300	-2.25429	2.55570
H	3.25550	1.15631	1.67070
H	2.32500	0.18551	2.82230
H	4.06860	0.29641	2.99000
H	-2.73040	2.00551	3.15640
H	-1.43310	0.69131	3.06900
H	-2.48170	3.11431	-0.08310
H	-3.72340	1.87651	-0.23580
H	-3.68940	2.92611	1.19500
H	3.72970	-3.21399	-0.11480

Conformer 28

Energy: -1152.65894 Hartree (Rel: 1.7 kcal/mol)

XYZ coordinates for conf 28:

C	1.41310	-0.82610	-0.53421
O	0.62440	-2.03030	-0.67751
C	-0.70360	-1.71270	-0.76681
C	-0.84350	-0.23820	-0.68781
C	0.38630	0.27650	-0.55521
C	2.25970	-0.95150	0.75659
C	3.28900	0.20010	0.94339
C	2.97250	-2.30240	0.88019
O	2.61540	1.47440	1.02849
C	3.18120	2.36120	0.14649
C	4.28030	1.67171	-0.57651
C	4.33070	0.41281	-0.12051
O	2.78179	3.49860	0.03339
C	5.10800	2.37951	-1.59901
O	-1.55880	-2.56310	-0.90031
C	-2.18820	0.41710	-0.75211
C	-3.03010	0.15370	0.52419
C	-4.45830	0.64560	0.39229
C	-5.47970	-0.12671	-0.07251
C	-6.92040	0.31179	-0.19961
C	-5.27630	-1.55570	-0.51681
H	3.76860	0.02670	1.91429
H	2.07000	-0.76850	-1.41131
C	3.08930	-2.86340	2.08909
C	3.53830	-2.96840	-0.35361
C	-4.65910	2.05600	0.76409
O	-3.75620	2.79550	1.14059
H	0.64140	1.32500	-0.47871
H	1.56750	-0.86110	1.60109
H	5.03800	-0.35279	-0.41461
H	5.87100	1.71991	-2.02021
H	4.47820	2.75371	-2.41461
H	5.60330	3.25191	-1.15701
H	-2.07620	1.49590	-0.89701
H	-2.72770	0.02140	-1.62191

H	-3.00710	-0.91300	0.75779
H	-2.55780	0.68120	1.35839
H	-7.14720	1.29019	0.22249
H	-7.21400	0.32079	-1.25781
H	-7.56880	-0.42481	0.29259
H	-4.23220	-1.83780	-0.65891
H	-5.71880	-2.24120	0.22039
H	-5.81180	-1.73321	-1.45801
H	3.61700	-3.80309	2.23059
H	2.65800	-2.41030	2.97889
H	2.73440	-3.27530	-1.03211
H	4.20260	-2.31049	-0.92681
H	4.10820	-3.86079	-0.07911
H	-5.68440	2.45930	0.68549

Conformer 29

Energy: -1152.65630 Hartree (Rel: 1.8 kcal/mol)

XYZ coordinates for conf 29:

C	0.52040	0.06210	0.29020
O	-0.04140	0.00770	-1.03470
C	-1.27920	-0.58469	-0.98590
C	-1.58230	-0.93339	0.42620
C	-0.52990	-0.55820	1.16440
C	1.90940	-0.62350	0.33050
C	2.89820	0.05229	-0.65020
C	1.84419	-2.13660	0.12340
O	4.20330	-0.52941	-0.43840
C	5.10670	0.45389	-0.13540
C	4.40531	1.76419	-0.15420
C	3.12250	1.52669	-0.45680
O	6.27180	0.21348	0.09290
C	5.12921	3.04169	0.12070
O	-1.94710	-0.75219	-1.98280
C	-2.88980	-1.55659	0.81960
C	-3.89830	-0.55079	1.43680
C	-4.13300	0.70631	0.61610
C	-4.85640	0.73092	-0.53870
C	-5.08339	1.94102	-1.41310
C	-5.49640	-0.51188	-1.10480
H	2.60190	-0.17051	-1.68090
H	0.64410	1.12240	0.54520
C	1.54559	-2.68850	-1.05780
C	2.14189	-2.97031	1.34450
C	-3.50299	1.92581	1.14990
O	-2.82109	1.96341	2.16900
H	-0.41300	-0.64890	2.23860
H	2.29840	-0.42471	1.33770
H	2.33961	2.26629	-0.57870
H	4.45811	3.90219	0.05740
H	5.58141	3.02208	1.11930
H	5.94871	3.18228	-0.59390
H	-3.32341	-2.02049	-0.07170
H	-2.71441	-2.35699	1.54780
H	-4.84090	-1.08218	1.61270
H	-3.52090	-0.23709	2.41360
H	-4.55509	2.84371	-1.10880
H	-4.77899	1.70382	-2.44080
H	-6.15639	2.17062	-1.45380
H	-5.66340	-1.29238	-0.35940
H	-6.45520	-0.27198	-1.57860
H	-4.84500	-0.92658	-1.88620
H	1.50249	-3.76850	-1.17380
H	1.33819	-2.10050	-1.94650
H	3.15719	-2.76761	1.71100

H	1.45509	-2.73230	2.16840
H	2.06019	-4.04040	1.13170
H	-3.67299	2.86121	0.58870

Conformer 30
Energy: -1152.65913 Hartree (Rel: 1.9 kcal/mol)
XYZ coordinates for conf 30:

C	0.50400	0.03571	0.35931
O	-0.02580	-0.03510	-0.98189
C	-1.28050	-0.59500	-0.95119
C	-1.60750	-0.94820	0.45371
C	-0.55760	-0.59960	1.20861
C	1.91440	-0.59989	0.45121
C	2.86610	0.07001	-0.57129
C	1.92030	-2.12249	0.34371
O	4.17500	-0.52339	-0.44539
C	5.10490	0.44982	-0.19019
C	4.41269	1.76441	-0.14539
C	3.11190	1.54001	-0.37149
O	6.28050	0.19832	-0.04429
C	5.16159	3.03272	0.10421
O	-1.93500	-0.74810	-1.95929
C	-2.92730	-1.55790	0.82661
C	-3.93830	-0.53820	1.41631
C	-4.15150	0.71080	0.57741
C	-4.85930	0.72740	-0.58709
C	-5.06381	1.92849	-1.47939
C	-5.50280	-0.51641	-1.14689
H	2.51020	-0.13279	-1.58769
H	0.58900	1.10141	0.61071
C	2.07390	-2.85849	1.45021
C	1.76990	-2.76259	-1.01649
C	-3.51701	1.93130	1.10391
O	-2.84631	1.97530	2.13011
H	-0.45290	-0.71160	2.28171
H	2.28050	-0.33299	1.45061
H	2.32799	2.28631	-0.43189
H	4.49429	3.89851	0.09421
H	5.67259	2.99502	1.07351
H	5.93769	3.17882	-0.65629
H	-3.34900	-2.02560	-0.06859
H	-2.77180	-2.35330	1.56461
H	-4.88740	-1.06090	1.58371
H	-3.57340	-0.21590	2.39521
H	-4.53501	2.83149	-1.17689
H	-4.74341	1.67710	-2.49889
H	-6.13441	2.16429	-1.54129
H	-5.67900	-1.29001	-0.39649
H	-6.45730	-0.27481	-1.62869
H	-4.84920	-0.94081	-1.92109
H	2.06811	-3.94509	1.41441
H	2.21490	-2.40629	2.42911
H	0.87260	-2.41869	-1.54199
H	2.62590	-2.52139	-1.65979
H	1.72011	-3.85159	-0.92639
H	-3.67251	2.86120	0.52961

Conformer 31
Energy: -1152.65797 Hartree (Rel: 1.9 kcal/mol)
XYZ coordinates for conf 31:

C	-0.93571	0.57349	-0.61660
O	-0.33611	1.87659	-0.78130
C	0.96589	1.84889	-0.35460
C	1.28459	0.46859	0.10160

C	0.17629	-0.26791	-0.05600
C	-2.19181	0.73589	0.27420
C	-2.77261	-0.64161	0.68170
C	-3.22241	1.66839	-0.36190
O	-3.11271	-1.42071	-0.48040
C	-4.43211	-1.79951	-0.41240
C	-5.01051	-1.26941	0.84970
C	-4.04181	-0.59681	1.48300
O	-4.95441	-2.46631	-1.27660
C	-6.43471	-1.52521	1.22120
O	1.67159	2.83279	-0.38160
C	2.64129	0.07830	0.60360
C	3.73259	0.15210	-0.49670
C	5.09049	-0.31120	-0.00230
C	5.55709	-1.58380	-0.13500
C	6.91509	-2.07120	0.31650
C	4.74799	-2.68570	-0.77550
H	-1.99851	-1.19701	1.22680
H	-1.23511	0.21989	-1.60920
C	-3.72841	1.45399	-1.58140
C	-3.62891	2.85419	0.47670
C	5.87759	0.72840	0.68520
O	5.47859	1.87230	0.86900
H	0.06859	-1.32550	0.15690
H	-1.84141	1.18819	1.21140
H	-4.11141	-0.08851	2.43840
H	-6.68091	-1.07821	2.18780
H	-7.11051	-1.11311	0.46260
H	-6.63401	-2.60211	1.27090
H	2.93389	0.75230	1.41750
H	2.59379	-0.93531	1.01700
H	3.41319	-0.42770	-1.36670
H	3.81689	1.19500	-0.81410
H	7.59289	-1.29620	0.67200
H	6.79679	-2.81330	1.11740
H	7.40849	-2.59200	-0.51450
H	3.70049	-2.43060	-0.93810
H	5.18779	-2.96040	-1.74460
H	4.78769	-3.58880	-0.15330
H	-4.46311	2.13219	-2.00790
H	-3.43671	0.61049	-2.19970
H	-4.04241	2.53799	1.44440
H	-2.75801	3.48599	0.69680
H	-4.37971	3.46779	-0.03000
H	6.87639	0.44220	1.05940

LIST OF FILES (technical info - delete in the final SI version
kallopterolideB-epi8-du8ml-chloroform_1013.log kallopterolideB-epi8-du8ml-chloroform_1025.log
kallopterolideB-epi8-du8ml-chloroform_1019.log kallopterolideB-epi8-du8ml-chloroform_1065.log
kallopterolideB-epi8-du8ml-chloroform_12.log kallopterolideB-epi8-du8ml-chloroform_1066.log
kallopterolideB-epi8-du8ml-chloroform_1069.log kallopterolideB-epi8-du8ml-chloroform_1042.log
kallopterolideB-epi8-du8ml-chloroform_103.log kallopterolideB-epi8-du8ml-chloroform_1047.log
kallopterolideB-epi8-du8ml-chloroform_35.log kallopterolideB-epi8-du8ml-chloroform_105.log
kallopterolideB-epi8-du8ml-chloroform_1045.log kallopterolideB-epi8-du8ml-chloroform_1039.log
kallopterolideB-epi8-du8ml-chloroform_1023.log kallopterolideB-epi8-du8ml-chloroform_1018.log
kallopterolideB-epi8-du8ml-chloroform_1038.log kallopterolideB-epi8-du8ml-chloroform_1015.log
kallopterolideB-epi8-du8ml-chloroform_1055.log kallopterolideB-epi8-du8ml-chloroform_1037.log
kallopterolideB-epi8-du8ml-chloroform_1033.log kallopterolideB-epi8-du8ml-chloroform_1050.log
kallopterolideB-epi8-du8ml-chloroform_1049.log kallopterolideB-epi8-du8ml-chloroform_1026.log
kallopterolideB-epi8-du8ml-chloroform_1061.log kallopterolideB-epi8-du8ml-chloroform_1057.log
kallopterolideB-epi8-du8ml-chloroform_1040.log kallopterolideB-epi8-du8ml-chloroform_1046.log
kallopterolideB-epi8-du8ml-chloroform_1027.log kallopterolideB-epi8-du8ml-chloroform_106.log
kallopterolideB-epi8-du8ml-chloroform_1028.log

DU8ML data for 2-(2-((*S*)-5-((*S*,*E*)-2-hydroxy-2-methyl-3-(4-methyl-5-oxofuran-2(5*H*)-ylidene)propyl)-2-oxo-2,5-dihydrofuran-3-yl)ethyl)-3-methylbut-2-enal (**10*S*,8*S*,6*E* diastereomer**)

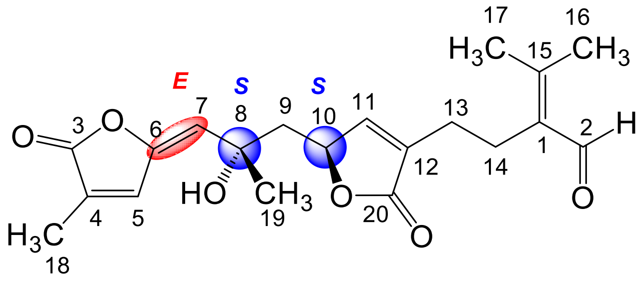

NMR parameters calculated for 10*S*,8*S*,6*E* diastereomer vs experimental data of kallopterolide C (**3**):

	Conf1	Conf2	Conf3	Conf4	Conf5	Conf6	Conf7	Conf8
Rel energy (kcal/mol):	0.00	1.10	1.13	1.19	1.23	1.61	1.84	2.15

C-nom	iGau	Exp	Calc	diff	1	2	3	4	5	6	7	8
C1-C	2	135.35	135.97	0.61	[136.52	134.62	136.30	134.73	134.09	136.08	136.79	134.69]
C2-CH	1	190.66	189.73	-0.93	[189.45	189.94	190.89	189.95	190.03	189.89	189.24	190.26]
C3-C	22	170.02	169.22	-0.80	[169.20	169.22	169.36	169.17	169.26	169.27	169.27	169.24]
C4-C	21	131.27	131.51	0.24	[131.54	131.57	131.21	131.75	131.28	131.46	131.60	131.47]
C5-CH	20	136.50	137.08	0.58	[137.05	137.06	137.37	136.90	137.13	137.13	137.11	137.14]
C6-C	19	149.51	150.07	0.56	[150.09	150.10	149.94	150.15	149.89	150.08	150.17	150.09]
C7-CH	18	117.38	116.72	-0.66	[116.63	116.69	117.37	116.29	117.22	116.74	116.53	116.80]
C8-C	15	72.72	74.53	1.81	[74.54	74.51	74.64	74.44	74.49	74.59	74.47	74.53]
C9-CH2	14	46.30	44.87	-1.42	[44.98	44.98	44.05	45.29	44.29	44.93	45.09	45.03]
C10-CH	11	78.77	80.06	1.28	[80.22	80.00	79.74	79.93	79.97	79.35	79.50	79.70]
C11-CH	12	148.56	147.71	-0.85	[147.43	149.39	145.24	149.43	150.04	145.46	147.42	148.35]
C12-C	8	133.63	133.77	0.14	[134.01	133.05	133.87	133.03	132.92	133.71	134.70	135.13]
C13-CH2	7	24.39	26.47	2.08	[26.93	25.61	27.67	25.63	23.07	27.63	27.26	23.43]
C14-CH2	3	23.39	24.99	1.60	[25.51	24.21	23.31	24.29	25.15	22.61	25.22	28.00]
C15-C	4	157.06	158.88	1.83	[158.75	158.60	159.00	158.46	162.50	158.11	156.77	155.43]
C16-CH3**	6	23.31	21.81	-1.50	[21.69	21.75	21.84	21.97	22.72	21.73	21.76	21.77]
C17-CH3**	5	19.38	16.98	-2.40	[17.03	16.88	16.76	16.96	17.25	16.62	16.83	16.92]
C18-CH3	25	10.83	11.10	0.27	[11.11	11.12	11.09	11.05	11.08	11.16	11.12	11.11]
C19-CH3	16	30.80	29.67	-1.13	[29.68	29.65	29.66	29.70	29.60	29.70	29.74	29.63]
C20-C	9	172.67	172.60	-0.07	[172.77	172.60	171.86	172.64	173.19	171.52	171.16	172.35]

13C chem shifts: RMSD=1.23ppm (MAE=1.04) N=20 {-2.40 2.08}

Fractions:	0.586	0.092	0.087	0.079	0.074	0.039	0.026	0.016

NMR parameters calculated for 10*S*,8*S*,6*E* diastereomer vs experimental data of kallopterolide D (**4**):

C-nom	iGau	Exp	Calc	diff	1	2	3	4	5	6	7	8
C1-C	2	135.49	135.97	0.47	[136.52	134.62	136.30	134.73	134.09	136.08	136.79	134.69]
C2-CH	1	190.65	189.73	-0.92	[189.45	189.94	190.89	189.95	190.03	189.89	189.24	190.26]
C3-C	22	169.95	169.22	-0.73	[169.20	169.22	169.36	169.17	169.26	169.27	169.27	169.24]
C4-C	21	129.26	131.51	2.25	[131.54	131.57	131.21	131.75	131.28	131.46	131.60	131.47]
C5-CH	20	138.63	137.08	-1.55	[137.05	137.06	137.37	136.90	137.13	137.13	137.11	137.14]
C6-C	19	146.88	150.07	3.19	[150.09	150.10	149.94	150.15	149.89	150.08	150.17	150.09]
C7-CH	18	118.26	116.72	-1.54	[116.63	116.69	117.37	116.29	117.22	116.74	116.53	116.80]
C8-C	15	71.78	74.53	2.75	[74.54	74.51	74.64	74.44	74.49	74.59	74.47	74.53]
C9-CH2	14	45.85	44.87	-0.97	[44.98	44.98	44.05	45.29	44.29	44.93	45.09	45.03]
C10-CH	11	78.34	80.06	1.71	[80.22	80.00	79.74	79.93	79.97	79.35	79.50	79.70]
C11-CH	12	149.56	147.71	-1.85	[147.43	149.39	145.24	149.43	150.04	145.46	147.42	148.35]

C12-C	8	133.05	133.77	0.72	[	134.01	133.05	133.87	133.03	132.92	133.71	134.70	135.13	]
C13-CH2	7	24.37	26.47	2.09	[	26.93	25.61	27.67	25.63	23.07	27.63	27.26	23.43	]
C14-CH2	3	23.38	24.99	1.61	[	25.51	24.21	23.31	24.29	25.15	22.61	25.22	28.00	]
C15-C	4	156.72	158.88	2.16	[	158.75	158.60	159.00	158.46	162.50	158.11	156.77	155.43	]
C16-CH3**	6	23.42	21.81	-1.61	[	21.69	21.75	21.84	21.97	22.72	21.73	21.76	21.77	]
C17-CH3**	5	19.35	16.98	-2.37	[	17.03	16.88	16.76	16.96	17.25	16.62	16.83	16.92	]
C18-CH3	25	10.51	11.10	0.59	[	11.11	11.12	11.09	11.05	11.08	11.16	11.12	11.11	]
C19-CH3	16	30.14	29.67	-0.47	[	29.68	29.65	29.66	29.70	29.60	29.70	29.74	29.63	]
C20-C	9	173.51	172.60	-0.91	[	172.77	172.60	171.86	172.64	173.19	171.52	171.16	172.35	]

13C chem shifts: RMSD=1.71ppm (MAE=1.52) N=20 {-2.37 3.19}

Fractions: 0.586 0.092 0.087 0.079 0.074 0.039 0.026 0.016

NMR parameters calculated for 10*S*,8*S*,6*E* diastereomer vs experimental data of kallopterolide E (**5**):

**C-nom**	**iGau**	**Exp**	**Calc**	**diff**		**1**	**2**	**3**	**4**	**5**	**6**	**7**	**8**	
C1-C	2	135.53	135.97	0.43	[	136.52	134.62	136.30	134.73	134.09	136.08	136.79	134.69	]
C2-CH	1	190.64	189.73	-0.91	[	189.45	189.94	190.89	189.95	190.03	189.89	189.24	190.26	]
C3-C	22	170.05	169.22	-0.82	[	169.20	169.22	169.36	169.17	169.26	169.27	169.27	169.24	]
C4-C	21	129.50	131.51	2.00	[	131.54	131.57	131.21	131.75	131.28	131.46	131.60	131.47	]
C5-CH	20	138.53	137.08	-1.45	[	137.05	137.06	137.37	136.90	137.13	137.13	137.11	137.14	]
C6-C	19	145.96	150.07	4.11	[	150.09	150.10	149.94	150.15	149.89	150.08	150.17	150.09	]
C7-CH	18	119.20	116.72	-2.48	[	116.63	116.69	117.37	116.29	117.22	116.74	116.53	116.80	]
C8-C	15	72.05	74.53	2.49	[	74.54	74.51	74.64	74.44	74.49	74.59	74.47	74.53	]
C9-CH2	14	45.10	44.87	-0.23	[	44.98	44.98	44.05	45.29	44.29	44.93	45.09	45.03	]
C10-CH	11	78.82	80.06	1.23	[	80.22	80.00	79.74	79.93	79.97	79.35	79.50	79.70	]
C11-CH	12	149.11	147.71	-1.40	[	147.43	149.39	145.24	149.43	150.04	145.46	147.42	148.35	]
C12-C	8	133.19	133.77	0.59	[	134.01	133.05	133.87	133.03	132.92	133.71	134.70	135.13	]
C13-CH2	7	24.37	26.47	2.09	[	26.93	25.61	27.67	25.63	23.07	27.63	27.26	23.43	]
C14-CH2	3	23.41	24.99	1.58	[	25.51	24.21	23.31	24.29	25.15	22.61	25.22	28.00	]
C15-C	4	156.79	158.88	2.09	[	158.75	158.60	159.00	158.46	162.50	158.11	156.77	155.43	]
C16-CH3**	6	23.35	21.81	-1.54	[	21.69	21.75	21.84	21.97	22.72	21.73	21.76	21.77	]
C17-CH3**	5	19.36	16.98	-2.38	[	17.03	16.88	16.76	16.96	17.25	16.62	16.83	16.92	]
C18-CH3	25	10.52	11.10	0.59	[	11.11	11.12	11.09	11.05	11.08	11.16	11.12	11.11	]
C19-CH3	16	28.92	29.67	0.75	[	29.68	29.65	29.66	29.70	29.60	29.70	29.74	29.63	]
C20-C	9	173.15	172.60	-0.56	[	172.77	172.60	171.86	172.64	173.19	171.52	171.16	172.35	]

13C chem shifts: RMSD=1.75ppm (MAE=1.49) N=20 {-2.48 4.11}

Fractions: 0.586 0.092 0.087 0.079 0.074 0.039 0.026 0.016

Conformer 1

Energy: -1227.88979 Hartree (Rel: 0.0 kcal/mol)

XYZ coordinates for conf 1:

C	-6.20219	0.16219	1.62650
C	-5.45459	-0.53631	0.56780
C	-3.94669	-0.43961	0.69320
C	-6.08639	-1.17511	-0.45600
C	-7.58319	-1.31941	-0.60550
C	-5.33309	-1.83791	-1.58450
C	-3.37339	0.83909	0.02780
C	-1.87609	0.85019	0.02670
C	-1.10459	-0.06541	-0.85050
O	0.23641	0.14329	-0.62210
C	0.40961	1.18979	0.37090
C	-0.99519	1.57239	0.73260
O	-1.49639	-0.88171	-1.65470
C	1.25211	2.33339	-0.20810
C	2.66811	1.92169	-0.69960
C	3.42531	3.17829	-1.16630
O	2.56551	1.08410	-1.85160
C	3.43921	1.27609	0.43570
C	4.17561	0.15360	0.39810
C	4.48541	-0.80940	-0.64760
C	5.29821	-1.75950	-0.13740
C	5.52901	-1.41930	1.28320
O	4.83241	-0.25410	1.56010

O	6.18611	-1.98370	2.12920
C	5.91441	-2.96140	-0.77270
O	-5.66779	0.79109	2.53390
H	-7.30479	0.11719	1.58250
H	-3.46269	-1.31941	0.26360
H	-3.69009	-0.41341	1.75650
H	-7.92199	-0.77651	-1.49790
H	-8.17019	-0.97321	0.24440
H	-7.83029	-2.37531	-0.77630
H	-5.80199	-1.58621	-2.54420
H	-5.40259	-2.93091	-1.48750
H	-4.27759	-1.56811	-1.63920
H	-3.72569	0.89059	-1.00990
H	-3.75519	1.72159	0.54990
H	0.93261	0.73040	1.21910
H	-1.21709	2.33869	1.46740
H	0.71681	2.78369	-1.05160
H	1.34521	3.09950	0.57140
H	3.61441	3.86190	-0.33290
H	2.84061	3.70240	-1.92980
H	4.38541	2.88470	-1.60100
H	1.84711	0.44599	-1.68760
H	3.42431	1.81239	1.38300
H	4.11161	-0.72010	-1.65680
H	5.62931	-3.04360	-1.82470
H	5.60491	-3.87690	-0.25400
H	7.00841	-2.91740	-0.70900

Conformer 2
Energy: -1227.88898 Hartree (Rel: 1.1 kcal/mol)
XYZ coordinates for conf 2:

C	-5.52087	0.71467	-1.17141
C	-4.57395	0.98687	-0.07475
C	-4.55730	-0.08254	1.00155
C	-3.81245	2.11755	-0.06213
C	-3.84508	3.20350	-1.11492
C	-2.83088	2.44437	1.03712
C	-3.83621	-1.39455	0.58810
C	-2.36690	-1.24417	0.33712
C	-1.40853	-0.94532	1.43049
O	-0.14302	-0.87379	0.89099
C	-0.20042	-1.13730	-0.53592
C	-1.66183	-1.34935	-0.79816
O	-1.60878	-0.77038	2.61154
C	0.69011	-2.33542	-0.88857
C	2.18889	-2.17129	-0.51044
C	2.97160	-3.40048	-1.00682
O	2.34092	-2.16505	0.90939
C	2.75306	-0.92823	-1.17142
C	3.52700	0.02003	-0.61868
C	4.07220	0.22019	0.71533
C	4.80755	1.35286	0.71948
C	4.74309	1.92005	-0.64479
O	3.96098	1.07824	-1.41882
O	5.24228	2.92376	-1.10259
C	5.58350	2.00959	1.81230
O	-6.23271	-0.28283	-1.22238
H	-5.58475	1.45758	-1.98548
H	-5.59532	-0.35502	1.22054
H	-4.12313	0.28724	1.93133
H	-4.03486	4.17150	-0.63336
H	-4.58642	3.07199	-1.90159
H	-2.85850	3.28604	-1.58989
H	-3.17052	3.33553	1.58323

H	-1.85796	2.70758	0.60191
H	-2.67334	1.64360	1.75859
H	-3.97853	-2.12016	1.39932
H	-4.31981	-1.80323	-0.30294
H	0.17249	-0.23377	-1.03490
H	-2.04797	-1.56787	-1.78791
H	0.30762	-3.22932	-0.38302
H	0.60320	-2.50440	-1.96880
H	2.97122	-3.45821	-2.09977
H	2.52321	-4.31442	-0.60300
H	4.00767	-3.33669	-0.66125
H	1.62908	-1.61094	1.27892
H	2.53501	-0.81524	-2.23193
H	3.89837	-0.46788	1.52929
H	5.50391	1.44870	2.74710
H	5.22302	3.03088	1.98510
H	6.64348	2.09079	1.54255

Conformer 3

Energy: -1227.89154 Hartree (Rel: 1.1 kcal/mol)

XYZ coordinates for conf 3:

C	-3.87080	0.96970	-1.08800
C	-4.70840	0.03020	-0.32270
C	-4.31980	-1.43210	-0.45170
C	-5.74400	0.47260	0.44600
C	-6.18040	1.91260	0.59190
C	-6.60320	-0.45840	1.26590
C	-3.36400	-1.94410	0.66210
C	-1.99280	-1.34890	0.57750
C	-1.04100	-1.77450	-0.47920
O	0.12180	-1.05150	-0.32820
C	-0.02060	-0.11430	0.77160
C	-1.39110	-0.40370	1.31070
O	-1.16560	-2.61380	-1.34040
C	0.16060	1.32030	0.25710
C	1.54420	1.61180	-0.38820
C	1.61410	3.09890	-0.78170
O	1.68670	0.88220	-1.60680
C	2.64860	1.31630	0.60870
C	3.77630	0.61530	0.40820
C	4.33230	-0.08820	-0.73740
C	5.52120	-0.62310	-0.38470
C	5.75690	-0.26780	1.03100
O	4.67790	0.48320	1.46660
O	6.68510	-0.53170	1.76310
C	6.50080	-1.42971	-1.17080
O	-2.89960	0.63330	-1.75790
H	-4.14330	2.03820	-1.04520
H	-3.82640	-1.57590	-1.41660
H	-5.21030	-2.06690	-0.45270
H	-6.10610	2.21630	1.64450
H	-7.24070	2.00110	0.32150
H	-5.62300	2.63270	-0.00520
H	-7.64800	-0.39370	0.93280
H	-6.59780	-0.14260	2.31770
H	-6.29570	-1.50320	1.22430
H	-3.79510	-1.74250	1.64850
H	-3.27780	-3.03260	0.55630
H	0.76530	-0.36040	1.49650
H	-1.79990	0.12190	2.16690
H	-0.61640	1.52710	-0.48740
H	0.00450	2.00060	1.10350
H	1.58260	3.74720	0.09970
H	0.77280	3.34810	-1.43690

H	2.54650	3.28990	-1.32140
H	1.28990	0.00190	-1.47260
H	2.52510	1.75830	1.59590
H	3.83730	-0.12950	-1.69610
H	7.47940	-0.93520	-1.19780
H	6.15710	-1.57980	-2.19750
H	6.65650	-2.41170	-0.70760

Conformer 4

Energy: -1227.88974 Hartree (Rel: 1.2 kcal/mol)

XYZ coordinates for conf 4:

C	5.71490	0.07290	-1.00510
C	4.81420	-0.05040	0.15570
C	4.43330	-1.48110	0.48790
C	4.40140	1.04750	0.85030
C	4.80240	2.47430	0.54620
C	3.48690	0.97510	2.04930
C	3.40810	-2.11490	-0.49170
C	2.05660	-1.46820	-0.48480
C	1.15090	-1.54990	0.68830
O	-0.01670	-0.88650	0.38230
C	0.06750	-0.33340	-0.95790
C	1.42310	-0.76430	-1.43360
O	1.32010	-2.07890	1.76410
C	-0.14190	1.18580	-0.91760
C	-1.49050	1.64590	-0.29600
C	-1.60500	3.17650	-0.41420
O	-1.50550	1.36580	1.10430
C	-2.64650	1.00690	-1.04130
C	-3.73850	0.42220	-0.52250
C	-4.19670	0.17650	0.83650
C	-5.39140	-0.45081	0.78630
C	-5.73130	-0.62831	-0.64210
O	-4.70400	-0.08250	-1.39470
O	-6.69860	-1.13851	-1.16150
C	-6.29380	-0.92190	1.87770
O	6.12280	-0.88530	-1.65290
H	6.03320	1.08870	-1.29750
H	4.05760	-1.57220	1.50770
H	5.33980	-2.09260	0.42600
H	5.22210	2.93470	1.45010
H	3.91050	3.06140	0.28970
H	5.52730	2.59670	-0.25710
H	4.03520	1.28190	2.95110
H	3.05500	-0.00790	2.23260
H	2.66410	1.69310	1.93580
H	3.81890	-2.09230	-1.50430
H	3.29050	-3.16930	-0.21020
H	-0.73280	-0.80630	-1.54090
H	1.79540	-0.52120	-2.42290
H	0.67000	1.64610	-0.34290
H	-0.06830	1.55630	-1.94740
H	-1.66980	3.49130	-1.46040
H	-0.73220	3.65200	0.04610
H	-2.50430	3.51600	0.10810
H	-1.14710	0.46740	1.22650
H	-2.60110	1.06710	-2.12730
H	-3.63810	0.48260	1.70850
H	-7.27610	-0.43951	1.80600
H	-5.87070	-0.70691	2.86240
H	-6.46650	-2.00211	1.79950

Conformer 5
Energy: -1227.88959 Hartree (Rel: 1.2 kcal/mol)
XYZ coordinates for conf 5:

C	3.87011	1.23469	1.05687
C	4.48989	0.15646	0.26915
C	4.58474	0.41846	-1.22490
C	4.91702	-0.99435	0.86155
C	4.80930	-1.32422	2.33104
C	5.52528	-2.12261	0.06681
C	3.54682	-0.34470	-2.09242
C	2.12870	-0.11664	-1.65909
C	1.42815	-1.06134	-0.75477
O	0.17088	-0.56003	-0.49609
C	-0.00402	0.70850	-1.18196
C	1.28772	0.89343	-1.91968
O	1.80206	-2.11347	-0.28769
C	-0.30226	1.82482	-0.17152
C	-1.55330	1.58835	0.71891
C	-1.77415	2.82117	1.61430
O	-1.32450	0.49793	1.61280
C	-2.77705	1.38925	-0.15443
C	-3.72973	0.44984	-0.03816
C	-3.94082	-0.65901	0.87960
C	-5.08740	-1.28921	0.54481
C	-5.64743	-0.58238	-0.62724
O	-4.79428	0.46246	-0.94135
O	-6.65489	-0.79443	-1.26503
C	-5.76675	-2.47001	1.15491
O	3.42061	2.27157	0.57732
H	3.81789	1.08819	2.14936
H	5.58460	0.16981	-1.59878
H	4.44120	1.49099	-1.38139
H	5.81346	-1.47009	2.75060
H	4.28364	-2.28105	2.44669
H	4.29184	-0.58441	2.94023
H	4.75847	-2.88871	-0.11300
H	6.33119	-2.60392	0.63281
H	5.92007	-1.81067	-0.90224
H	3.67011	-0.02641	-3.13393
H	3.74927	-1.41988	-2.05471
H	-0.85289	0.57718	-1.86448
H	1.47723	1.75892	-2.54509
H	0.56831	1.95983	0.47994
H	-0.43119	2.75406	-0.74004
H	-2.02308	3.70580	1.01975
H	-0.86711	3.02676	2.19247
H	-2.59639	2.62507	2.30899
H	-0.88279	-0.20725	1.10480
H	-2.91717	2.12228	-0.94689
H	-3.26501	-0.88513	1.69098
H	-6.77485	-2.20570	1.49664
H	-5.20058	-2.85664	2.00626
H	-5.88408	-3.27441	0.41870

Conformer 6
Energy: -1227.88811 Hartree (Rel: 1.6 kcal/mol)
XYZ coordinates for conf 6:

C	-3.25361	2.03129	0.31350
C	-4.38620	1.09229	0.21540
C	-4.36610	-0.04121	1.22530
C	-5.35800	1.25179	-0.72690
C	-5.41941	2.36469	-1.74830
C	-6.52220	0.30139	-0.86320
C	-3.74330	-1.36081	0.69210

C	-2.28130	-1.24110	0.39140
C	-1.27670	-1.14660	1.48090
O	-0.03300	-0.99890	0.90620
C	-0.15090	-1.02180	-0.53990
C	-1.62470	-1.18100	-0.77430
O	-1.42530	-1.20360	2.67900
C	0.71490	-2.14750	-1.11900
C	2.23140	-2.03939	-0.79590
C	2.98121	-3.18829	-1.49490
O	2.45350	-2.22849	0.60170
C	2.77200	-0.71869	-1.30940
C	3.55910	0.15871	-0.66590
C	4.13840	0.19951	0.66800
C	4.86900	1.32841	0.79090
C	4.76949	2.05461	-0.49340
O	3.96940	1.30851	-1.34310
O	5.25799	3.10691	-0.84060
C	5.66779	1.85542	1.93630
O	-2.33861	1.91890	1.12130
H	-3.24471	2.88469	-0.38650
H	-5.37720	-0.26251	1.57910
H	-3.78740	0.28179	2.09450
H	-5.46921	1.93559	-2.75770
H	-4.58741	3.06699	-1.72440
H	-6.34701	2.93619	-1.61170
H	-6.50170	-0.17461	-1.85310
H	-7.46830	0.85658	-0.81090
H	-6.54570	-0.48631	-0.11000
H	-3.88100	-2.13231	1.46010
H	-4.27680	-1.69191	-0.20540
H	0.20740	-0.04860	-0.89910
H	-2.05140	-1.23030	-1.77020
H	0.35271	-3.10990	-0.73960
H	0.57370	-2.14720	-2.20690
H	2.92771	-3.09559	-2.58420
H	2.54741	-4.14919	-1.19860
H	4.03331	-3.17229	-1.19520
H	1.75110	-1.74950	1.07930
H	2.52070	-0.47479	-2.34010
H	3.98720	-0.58229	1.39740
H	5.61900	1.18251	2.79630
H	5.30129	2.84241	2.24350
H	6.71909	1.98372	1.65150

Conformer 7
Energy: -1227.88861 Hartree (Rel: 1.8 kcal/mol)
XYZ coordinates for conf 7:

C	-5.98871	-1.86189	-0.25680
C	-5.57471	-0.45089	-0.14870
C	-4.09441	-0.21329	-0.38250
C	-6.46400	0.52951	0.17120
C	-7.94810	0.33202	0.38080
C	-6.05660	1.97201	0.35020
C	-3.24921	-0.42719	0.90040
C	-1.79191	-0.14390	0.69220
C	-0.94071	-0.97670	-0.19690
O	0.33919	-0.46230	-0.17180
C	0.38660	0.70630	0.68650
C	-1.01760	0.82500	1.20050
O	-1.22141	-1.94830	-0.85870
C	0.87970	1.92450	-0.10500
C	2.28080	1.75859	-0.75760
C	2.68390	3.08329	-1.43050
O	2.22160	0.79500	-1.80960

C	3.30670	1.41289	0.30470
C	4.26130	0.46969	0.25150
C	4.64639	-0.51451	-0.74860
C	5.70299	-1.21581	-0.28460
C	6.02259	-0.68601	1.05850
O	5.12670	0.33319	1.33810
O	6.88359	-1.00962	1.84600
C	6.48669	-2.32502	-0.90350
O	-5.21341	-2.77949	-0.49840
H	-7.05771	-2.08948	-0.09870
H	-3.91420	0.79101	-0.77540
H	-3.74711	-0.92559	-1.13570
H	-8.50100	1.05362	-0.23470
H	-8.20920	0.55382	1.42420
H	-8.32231	-0.66298	0.14340
H	-6.50190	2.37521	1.26860
H	-6.45240	2.57971	-0.47580
H	-4.97830	2.12631	0.39910
H	-3.62650	0.20981	1.70810
H	-3.37331	-1.46879	1.21940
H	1.09220	0.47340	1.49410
H	-1.31760	1.60540	1.89160
H	0.16070	2.15180	-0.90020
H	0.89500	2.78010	0.58120
H	2.81870	3.88019	-0.69250
H	1.91110	3.38789	-2.14430
H	3.62500	2.94589	-1.97130
H	1.68959	0.04370	-1.48850
H	3.28980	2.03179	1.20000
H	4.14319	-0.61311	-1.69900
H	6.45229	-3.22282	-0.27470
H	7.54349	-2.04802	-1.00040
H	6.10019	-2.57831	-1.89410

Conformer 8
Energy: -1227.88965 Hartree (Rel: 2.1 kcal/mol)
XYZ coordinates for conf 8:

C	-5.23330	1.01021	1.04051
C	-4.45520	0.97701	-0.21289
C	-4.61150	-0.31619	-0.99079
C	-3.68910	2.03371	-0.60059
C	-3.55850	3.33741	0.15521
C	-2.86310	2.04141	-1.86469
C	-3.86690	-1.53219	-0.37359
C	-2.37490	-1.38659	-0.32969
C	-1.67710	-0.75029	0.81771
O	-0.32310	-0.74899	0.55181
C	-0.06880	-1.40009	-0.71969
C	-1.43790	-1.76529	-1.21099
O	-2.12290	-0.29639	1.84541
C	0.87450	-2.59389	-0.52319
C	2.25530	-2.24199	0.09771
C	3.12390	-3.51199	0.15101
O	2.09410	-1.82769	1.45451
C	2.95350	-1.20379	-0.75959
C	3.58290	-0.08859	-0.35509
C	3.81460	0.51801	0.94691
C	4.52930	1.65141	0.77801
C	4.77210	1.80211	-0.67289
O	4.18460	0.72511	-1.31669
O	5.36070	2.66511	-1.28529
C	5.03960	2.64161	1.77141
O	-5.98110	0.10651	1.39601
H	-5.12600	1.90121	1.68261

H	-4.30360	-0.19649	-2.03179
H	-5.67600	-0.57449	-1.00009
H	-4.22530	3.44981	1.00871
H	-3.74480	4.17571	-0.52849
H	-2.52700	3.45391	0.51351
H	-1.84540	2.38681	-1.64129
H	-3.28090	2.76291	-2.58079
H	-2.79060	1.07261	-2.35829
H	-4.24040	-1.68769	0.64291
H	-4.12380	-2.42209	-0.95879
H	0.40570	-0.65119	-1.36679
H	-1.59650	-2.27689	-2.15449
H	0.38880	-3.33349	0.12341
H	1.02440	-3.06019	-1.50469
H	3.36160	-3.87529	-0.85369
H	2.59590	-4.30039	0.69791
H	4.06010	-3.29129	0.67221
H	1.31390	-1.24419	1.49271
H	2.97820	-1.41159	-1.82789
H	3.46330	0.07931	1.86901
H	6.13180	2.72291	1.71351
H	4.76460	2.35841	2.79071
H	4.63780	3.64111	1.56571

LIST OF FILES (technical info - delete in the final SI version
abimael-PkC-SS-E-du8ml-chloroform_154.log abimael-PkC-SS-E-du8ml-chloroform_148.log abimael-PkC-SS-E-du8ml-chloroform_219.log abimael-PkC-SS-E-du8ml-chloroform_31.log abimael-PkC-SS-E-du8ml-chloroform_246.log abimael-PkC-SS-E-du8ml-chloroform_153.log abimael-PkC-SS-E-du8ml-chloroform_261.log abimael-PkC-SS-E-du8ml-chloroform_249.log

DU8ML data for 2-(2-((*S*)-5-((*S*,*Z*)-2-hydroxy-2-methyl-3-(4-methyl-5-oxofuran-2(5*H*)-ylidene)propyl)-2-oxo-2,5-dihydrofuran-3-yl)ethyl)-3-methylbut-2-enal (**10*S*,8*S*,6*Z* diastereomer**)

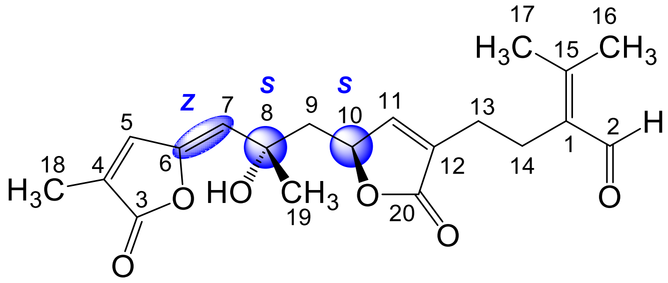

NMR parameters calculated for 10*S*,8*S*,6*Z* diastereomer vs experimental data of kallopterolide C (**3**):

	Conf1	Conf2	Conf3	Conf4	Conf5	Conf6	Conf7	Conf8	Conf9	Conf10	Conf11	Conf12	Conf13
Rel energy (kcal/mol):	0.00	0.04	0.17	0.18	0.37	0.39	0.46	0.46	0.48	0.61	0.68	0.70	0.84

Conf14	Conf15	Conf16	Conf17	Conf18	Conf19	Conf20	Conf21
1.02	1.17	1.19	1.22	1.31	1.47	1.58	1.63

C-nom	iGau	Exp	Calc	diff		1	2	3	4	5	6	7	8	9	10	11	12	13
C1-C	2	135.35	135.78	0.43	[	136.74	134.86	134.80	136.81	134.22	134.19	136.06	136.83	136.68	137.03	137.08	134.90	136.82
C2-CH	1	190.66	190.60	-0.06	[	193.26	189.93	189.87	193.25	190.50	189.92	189.89	189.47	189.53	189.31	189.26	190.28	189.49
C3-C	22	170.02	169.08	-0.94	[	169.30	169.53	169.60	169.75	168.62	169.61	169.65	168.40	168.21	168.21	168.02	168.73	168.38
C4-C	21	131.27	129.81	-1.46	[	130.34	130.28	130.11	128.42	129.03	129.94	129.90	130.09	130.46	128.84	128.96	130.60	129.49
C5-CH	20	136.50	139.10	2.60	[	139.16	138.98	139.05	139.67	139.53	139.07	139.11	138.65	138.50	139.21	139.18	138.91	139.28
C6-C	19	149.51	145.50	-4.01	[	146.86	144.88	144.63	143.89	146.85	144.51	144.66	145.92	146.97	145.03	145.08	147.00	145.67
C7-CH	18	117.38	120.54	3.16	[	120.97	119.36	120.48	121.50	122.79	120.82	121.30	120.97	120.06	118.68	118.86	120.17	120.27
C8-C	15	72.72	72.78	0.06	[	72.74	72.55	73.09	71.55	72.77	73.05	73.22	72.84	72.79	73.02	72.92	72.19	73.52
C9-CH2	14	46.30	44.24	-2.06	[	45.59	43.76	42.84	44.60	47.16	42.10	42.96	44.01	46.62	42.85	43.07	45.75	43.51
C10-CH	11	78.77	79.13	0.36	[	78.04	79.90	80.36	79.50	78.74	80.36	79.70	77.36	78.74	79.45	79.33	78.29	78.76
C11-CH	12	148.56	149.19	0.64	[	147.54	150.06	149.96	151.83	149.41	150.60	146.19	148.10	146.78	149.49	149.82	150.25	147.90
C12-C	8	133.63	132.33	-1.30	[	132.19	132.00	132.48	131.30	132.40	132.38	133.22	134.09	133.84	129.97	129.78	131.99	133.99
C13-CH2	7	24.39	26.05	1.66	[	27.26	25.72	25.74	26.67	22.10	23.15	27.45	27.07	27.13	27.00	26.72	26.01	27.06
C14-CH2	3	23.39	24.75	1.36	[	23.21	24.15	24.14	26.21	24.09	25.04	22.75	25.59	25.62	25.13	25.94	24.45	25.35
C15-C	4	157.06	159.64	2.58	[	159.19	158.73	158.11	163.41	164.55	162.09	158.24	158.78	159.04	158.27	157.61	159.08	158.64
C16-CH3**	6	23.31	22.07	-1.24	[	22.12	22.08	21.76	22.27	23.19	22.65	21.79	21.74	21.68	21.62	21.53	22.15	21.72
C17-CH3**	5	19.38	17.19	-2.18	[	17.16	17.03	16.84	17.53	17.50	17.20	16.64	16.97	16.99	16.90	16.99	16.79	17.02
C18-CH3	25	10.83	10.82	-0.01	[	10.84	10.80	10.82	10.80	10.89	10.79	10.81	10.89	10.90	10.74	10.69	10.84	10.87
C19-CH3	16	30.80	28.50	-2.30	[	24.74	28.90	29.11	29.56	30.89	29.01	28.91	27.59	31.27	30.88	30.94	24.64	27.41
C20-C	9	172.67	173.86	1.20	[	173.48	172.96	173.02	174.68	174.99	173.57	171.94	174.58	174.23	175.33	175.36	174.22	174.19

14	15	16	17	18	19	20	21	
137.02	136.97	134.76	134.86	131.74	135.08	136.92	134.80	]
189.36	188.78	190.00	190.17	193.15	189.90	189.43	189.93	]
168.19	169.17	168.06	169.66	169.57	168.02	169.35	169.80	]
130.32	128.65	130.27	130.05	130.28	130.45	130.52	130.34	]
138.59	140.06	138.64	139.11	138.94	138.56	138.76	138.67	]
146.30	144.60	146.43	144.51	144.82	146.65	145.55	148.33	]
120.11	119.67	120.53	120.72	120.41	120.45	122.51	117.03	]
72.95	73.40	73.21	73.10	73.20	73.11	72.99	74.51	]
43.99	43.05	45.47	42.90	42.99	45.53	42.15	44.51	]
78.50	78.59	77.49	80.05	80.65	77.44	77.80	79.59	]
150.13	149.42	150.03	148.92	148.10	150.02	147.79	148.89	]
132.16	131.02	133.10	134.63	133.28	133.12	134.17	133.17	]
27.11	25.74	25.66	23.36	28.97	25.66	27.03	25.73	]
25.44	26.15	24.18	27.85	30.14	24.38	25.85	24.18	]
158.44	158.51	158.64	155.11	159.05	157.95	158.72	158.51	]
21.65	21.96	21.81	21.76	23.79	21.99	21.77	21.79	]
17.02	18.51	16.91	16.93	24.45	16.90	17.03	16.89	]
10.86	10.67	10.84	10.76	10.84	10.87	10.85	10.78	]
26.54	31.09	25.72	29.15	29.03	25.76	26.25	29.11	]
174.66	173.76	174.46	172.75	172.97	174.49	174.59	172.90	]

13C chem shifts: RMSD=1.83ppm (MAE=1.48) N=20 {-4.01 3.16}

Fractions: 0.125 0.117 0.094 0.093 0.068 0.065 0.058 0.058 0.055 0.045 0.040 0.038 0.030 0.022 0.017 0.017 0.016 0.014 0.010 0.009 0.008

NMR parameters calculated for 10*S*,8*S*,6*Z* diastereomer vs experimental data of kallopterolide D (**4**):

C-nom	iGau	Exp	Calc	diff		1	2	3	4	5	6	7	8	9	10	11	12	13
C1-C	2	135.49	135.78	0.28	[	136.74	134.86	134.80	136.81	134.22	134.19	136.06	136.83	136.68	137.03	137.08	134.90	136.82
C2-CH	1	190.65	190.60	-0.05	[	193.26	189.93	189.87	193.25	190.50	189.92	189.89	189.47	189.53	189.31	189.26	190.28	189.49
C3-C	22	169.95	169.08	-0.87	[	169.30	169.53	169.60	169.75	168.62	169.61	169.65	168.40	168.21	168.21	168.02	168.73	168.38
C4-C	21	129.26	129.81	0.55	[	130.34	130.28	130.11	128.42	129.03	129.94	129.90	130.09	130.46	128.84	128.96	130.60	129.49
C5-CH	20	138.63	139.10	0.47	[	139.16	138.98	139.05	139.67	139.53	139.07	139.11	138.65	138.50	139.21	139.18	138.91	139.28
C6-C	19	146.88	145.50	-1.37	[	146.86	144.88	144.63	143.89	146.85	144.51	144.66	145.92	146.97	145.03	145.08	147.00	145.67
C7-CH	18	118.26	120.54	2.28	[	120.97	119.36	120.48	121.50	122.79	120.82	121.30	120.97	120.06	118.68	118.86	120.17	120.27
C8-C	15	71.78	72.78	1.00	[	72.74	72.55	73.09	71.55	72.77	73.05	73.22	72.84	72.79	73.02	72.92	72.19	73.52
C9-CH2	14	45.85	44.24	-1.61	[	45.59	43.76	42.84	44.60	47.16	42.10	42.96	44.01	46.62	42.85	43.07	45.75	43.51
C10-CH	11	78.34	79.13	0.79	[	78.04	79.90	80.36	79.50	78.74	80.36	79.70	77.36	78.74	79.45	79.33	78.29	78.76
C11-CH	12	149.56	149.19	-0.37	[	147.54	150.06	149.96	151.83	149.41	150.60	146.19	148.10	146.78	149.49	149.82	150.25	147.90
C12-C	8	133.05	132.33	-0.72	[	132.19	132.00	132.48	131.30	132.40	132.38	133.22	134.09	133.84	129.97	129.78	131.99	133.99
C13-CH2	7	24.37	26.05	1.68	[	27.26	25.72	25.74	26.67	22.10	23.15	27.45	27.07	27.13	27.00	26.72	26.01	27.06
C14-CH2	3	23.38	24.75	1.36	[	23.21	24.15	24.14	26.21	24.09	25.04	22.75	25.59	25.62	25.13	25.94	24.45	25.35
C15-C	4	156.72	159.64	2.92	[	159.19	158.73	158.11	163.41	164.55	162.09	158.24	158.78	159.04	158.27	157.61	159.08	158.64
C16-CH3**	6	23.42	22.07	-1.35	[	22.12	22.08	21.76	22.27	23.19	22.65	21.79	21.74	21.68	21.62	21.53	22.15	21.72
C17-CH3**	5	19.35	17.19	-2.16	[	17.16	17.03	16.84	17.53	17.50	17.20	16.64	16.97	16.99	16.90	16.99	16.79	17.02

C18-CH3	25	10.51	10.82	0.31	[	10.84	10.80	10.82	10.80	10.89	10.79	10.81	10.89	10.90	10.74	10.69	10.84	10.87
C19-CH3	16	30.14	28.50	-1.64	[	24.74	28.90	29.11	29.56	30.89	29.01	28.91	27.59	31.27	30.88	30.94	24.64	27.41
C20-C	9	173.51	173.86	0.35	[	173.48	172.96	173.02	174.68	174.99	173.57	171.94	174.58	174.23	175.33	175.36	174.22	174.19

**14**	**15**	**16**	**17**	**18**	**19**	**20**	**21**	
137.02	136.97	134.76	134.86	131.74	135.08	136.92	134.80	]
189.36	188.78	190.00	190.17	193.15	189.90	189.43	189.93	]
168.19	169.17	168.06	169.66	169.57	168.02	169.35	169.80	]
130.32	128.65	130.27	130.05	130.28	130.45	130.52	130.34	]
138.59	140.06	138.64	139.11	138.94	138.56	138.76	138.67	]
146.30	144.60	146.43	144.51	144.82	146.65	145.55	148.33	]
120.11	119.67	120.53	120.72	120.41	120.45	122.51	117.03	]
72.95	73.40	73.21	73.10	73.20	73.11	72.99	74.51	]
43.99	43.05	45.47	42.90	42.99	45.53	42.15	44.51	]
78.50	78.59	77.49	80.05	80.65	77.44	77.80	79.59	]
150.13	149.42	150.03	148.92	148.10	150.02	147.79	148.89	]
132.16	131.02	133.10	134.63	133.28	133.12	134.17	133.17	]
27.11	25.74	25.66	23.36	28.97	25.66	27.03	25.73	]
25.44	26.15	24.18	27.85	30.14	24.38	25.85	24.18	]
158.44	158.51	158.64	155.11	159.05	157.95	158.72	158.51	]
21.65	21.96	21.81	21.76	23.79	21.99	21.77	21.79	]
17.02	18.51	16.91	16.93	24.45	16.90	17.03	16.89	]
10.86	10.67	10.84	10.76	10.84	10.87	10.85	10.78	]
26.54	31.09	25.72	29.15	29.03	25.76	26.25	29.11	]
174.66	173.76	174.46	172.75	172.97	174.49	174.59	172.90	]

13C chem shifts: RMSD=1.34ppm (MAE=1.11) N=20 {-2.16 2.92}

Fractions: 0.125 0.117 0.094 0.093 0.068 0.065 0.058 0.058 0.055 0.045 0.040 0.038 0.030 0.022 0.017 0.017 0.016 0.014 0.010 0.009 0.008

NMR parameters calculated for 10*S*,8*S*,6*Z* diastereomer vs experimental data of kallopterolide E (**5**):

**C-nom**	**iGau**	**Exp**	**Calc**	**diff**		**1**	**2**	**3**	**4**	**5**	**6**	**7**	**8**	**9**	**10**	**11**	**12**	**13**
C1-C	2	135.53	135.78	0.25	[	136.74	134.86	134.80	136.81	134.22	134.19	136.06	136.83	136.68	137.03	137.08	134.90	136.82
C2-CH	1	190.64	190.60	-0.04	[	193.26	189.93	189.87	193.25	190.50	189.92	189.89	189.47	189.53	189.31	189.26	190.28	189.49
C3-C	22	170.05	169.08	-0.96	[	169.30	169.53	169.60	169.75	168.62	169.61	169.65	168.40	168.21	168.21	168.02	168.73	168.38
C4-C	21	129.50	129.81	0.31	[	130.34	130.28	130.11	128.42	129.03	129.94	129.90	130.09	130.46	128.84	128.96	130.60	129.49
C5-CH	20	138.53	139.10	0.57	[	139.16	138.98	139.05	139.67	139.53	139.07	139.11	138.65	138.50	139.21	139.18	138.91	139.28
C6-C	19	145.96	145.50	-0.45	[	146.86	144.88	144.63	143.89	146.85	144.51	144.66	145.92	146.97	145.03	145.08	147.00	145.67
C7-CH	18	119.20	120.54	1.35	[	120.97	119.36	120.48	121.50	122.79	120.82	121.30	120.97	120.06	118.68	118.86	120.17	120.27
C8-C	15	72.05	72.78	0.73	[	72.74	72.55	73.09	71.55	72.77	73.05	73.22	72.84	72.79	73.02	72.92	72.19	73.52
C9-CH2	14	45.10	44.24	-0.86	[	45.59	43.76	42.84	44.60	47.16	42.10	42.96	44.01	46.62	42.85	43.07	45.75	43.51
C10-CH	11	78.82	79.13	0.31	[	78.04	79.90	80.36	79.50	78.74	80.36	79.70	77.36	78.74	79.45	79.33	78.29	78.76
C11-CH	12	149.11	149.19	0.08	[	147.54	150.06	149.96	151.83	149.41	150.60	146.19	148.10	146.78	149.49	149.82	150.25	147.90
C12-C	8	133.19	132.33	-0.85	[	132.19	132.00	132.48	131.30	132.40	132.38	133.22	134.09	133.84	129.97	129.78	131.99	133.99

C13-CH2	7	24.37	26.05	1.67	[	27.26	25.72	25.74	26.67	22.10	23.15	27.45	27.07	27.13	27.00	26.72	26.01	27.06
C14-CH2	3	23.41	24.75	1.33	[	23.21	24.15	24.14	26.21	24.09	25.04	22.75	25.59	25.62	25.13	25.94	24.45	25.35
C15-C	4	156.79	159.64	2.85	[	159.19	158.73	158.11	163.41	164.55	162.09	158.24	158.78	159.04	158.27	157.61	159.08	158.64
C16-CH3**	6	23.35	22.07	-1.28	[	22.12	22.08	21.76	22.27	23.19	22.65	21.79	21.74	21.68	21.62	21.53	22.15	21.72
C17-CH3**	5	19.36	17.19	-2.16	[	17.16	17.03	16.84	17.53	17.50	17.20	16.64	16.97	16.99	16.90	16.99	16.79	17.02
C18-CH3	25	10.52	10.82	0.30	[	10.84	10.80	10.82	10.80	10.89	10.79	10.81	10.89	10.90	10.74	10.69	10.84	10.87
C19-CH3	16	28.92	28.50	-0.42	[	24.74	28.90	29.11	29.56	30.89	29.01	28.91	27.59	31.27	30.88	30.94	24.64	27.41
C20-C	9	173.15	173.86	0.71	[	173.48	172.96	173.02	174.68	174.99	173.57	171.94	174.58	174.23	175.33	175.36	174.22	174.19

**14**	**15**	**16**	**17**	**18**	**19**	**20**	**21**	
137.02	136.97	134.76	134.86	131.74	135.08	136.92	134.80	]
189.36	188.78	190.00	190.17	193.15	189.90	189.43	189.93	]
168.19	169.17	168.06	169.66	169.57	168.02	169.35	169.80	]
130.32	128.65	130.27	130.05	130.28	130.45	130.52	130.34	]
138.59	140.06	138.64	139.11	138.94	138.56	138.76	138.67	]
146.30	144.60	146.43	144.51	144.82	146.65	145.55	148.33	]
120.11	119.67	120.53	120.72	120.41	120.45	122.51	117.03	]
72.95	73.40	73.21	73.10	73.20	73.11	72.99	74.51	]
43.99	43.05	45.47	42.90	42.99	45.53	42.15	44.51	]
78.50	78.59	77.49	80.05	80.65	77.44	77.80	79.59	]
150.13	149.42	150.03	148.92	148.10	150.02	147.79	148.89	]
132.16	131.02	133.10	134.63	133.28	133.12	134.17	133.17	]
27.11	25.74	25.66	23.36	28.97	25.66	27.03	25.73	]
25.44	26.15	24.18	27.85	30.14	24.38	25.85	24.18	]
158.44	158.51	158.64	155.11	159.05	157.95	158.72	158.51	]
21.65	21.96	21.81	21.76	23.79	21.99	21.77	21.79	]
17.02	18.51	16.91	16.93	24.45	16.90	17.03	16.89	]
10.86	10.67	10.84	10.76	10.84	10.87	10.85	10.78	]
26.54	31.09	25.72	29.15	29.03	25.76	26.25	29.11	]
174.66	173.76	174.46	172.75	172.97	174.49	174.59	172.90	]

13C chem shifts: RMSD=1.12ppm (MAE=0.87) N=20 {-2.16 2.85}

Fractions: 0.125 0.117 0.094 0.093 0.068 0.065 0.058 0.058 0.055 0.045 0.040 0.038 0.030 0.022 0.017 0.017 0.016 0.014 0.010 0.009 0.008

Conformer 1
Energy: -1227.88917 Hartree (Rel: 0.0 kcal/mol)
XYZ coordinates for conf 1:

C	-0.87626	1.62584	-1.02473
C	-2.03598	2.08663	-0.23991
C	-3.36982	1.48138	-0.64025
C	-1.88356	2.98726	0.77214
C	-0.57602	3.60942	1.20882
C	-3.04490	3.47969	1.60072
C	-3.81250	0.27066	0.22835
C	-2.91768	-0.91893	0.06785
C	-2.97844	-1.77508	-1.14362
O	-2.02110	-2.74815	-1.03926
C	-1.27768	-2.57172	0.19028
C	-1.94998	-1.40370	0.85616
O	-3.73259	-1.71226	-2.09110
C	0.19825	-2.35441	-0.19273
C	1.20707	-2.29339	0.98793
C	1.23436	-3.59533	1.79911
O	0.84520	-1.27139	1.92576
C	2.58719	-2.04022	0.39492
C	3.08297	-0.84135	0.05398
C	4.36102	-0.47161	-0.52312
C	4.39792	0.86781	-0.69941
C	3.10506	1.40244	-0.22641
O	2.34221	0.32847	0.22641
O	2.67803	2.53433	-0.19524
C	5.46333	1.75430	-1.25347
O	-0.94678	0.78382	-1.91607
H	0.10239	2.07507	-0.79142
H	-3.29742	1.14661	-1.67833
H	-4.16270	2.23469	-0.60179
H	-0.47054	3.50597	2.29684
H	-0.60087	4.68938	1.00783
H	0.32191	3.20181	0.74605
H	-2.93577	3.13412	2.63810
H	-4.02065	3.15496	1.23818
H	-3.03866	4.57683	1.64210
H	-3.85345	0.56285	1.28336
H	-4.82872	-0.00823	-0.07691
H	-1.39781	-3.49626	0.76713
H	-1.63048	-1.03209	1.82067
H	0.48942	-3.16969	-0.86560
H	0.25965	-1.42525	-0.77051
H	1.44702	-4.45435	1.15469
H	0.27416	-3.75900	2.29616
H	2.00702	-3.53592	2.57187
H	0.96621	-0.41036	1.48961
H	3.22036	-2.90196	0.20236
H	5.13581	-1.18944	-0.76312
H	5.09446	2.30503	-2.12691
H	6.34182	1.17625	-1.55103
H	5.77105	2.50228	-0.51301

Conformer 2
Energy: -1227.88727 Hartree (Rel: 0.0 kcal/mol)
XYZ coordinates for conf 2:

C	4.82470	-1.09920	-1.67020
C	4.36590	-0.28890	-0.52680
C	4.35480	-1.04360	0.78960
C	3.99999	1.01560	-0.68540
C	4.01189	1.76940	-1.99760
C	3.53569	1.89630	0.44980
C	3.16670	-2.03520	0.94110

C	1.82770	-1.36540	0.92840
C	1.40549	-0.44060	2.00830
O	0.16670	0.06650	1.68720
C	-0.27901	-0.48750	0.41910
C	0.83740	-1.40930	0.02600
O	1.97989	-0.12090	3.02570
C	-0.53511	0.62440	-0.60940
C	-1.37141	1.82920	-0.10410
C	-1.66721	2.77330	-1.28220
O	-0.58991	2.60210	0.81470
C	-2.67771	1.44210	0.57150
C	-3.58970	0.53730	0.18200
C	-4.86660	0.19790	0.78510
C	-5.45740	-0.77040	0.05210
C	-4.54261	-1.08270	-1.06650
O	-3.42220	-0.26180	-0.94360
O	-4.64390	-1.87840	-1.97070
C	-6.76330	-1.47310	0.22320
O	5.16370	-2.27420	-1.57670
H	4.86740	-0.60910	-2.65840
H	4.35930	-0.36490	1.64340
H	5.27109	-1.64030	0.84720
H	4.71549	2.60980	-1.93110
H	3.02279	2.21170	-2.17420
H	4.27549	1.18060	-2.87490
H	4.14349	2.81040	0.48170
H	3.57219	1.42560	1.43130
H	2.50149	2.22580	0.27520
H	3.21440	-2.77950	0.14190
H	3.29410	-2.56520	1.89360
H	-1.20830	-1.02900	0.62720
H	0.81360	-1.99060	-0.88940
H	0.42369	1.02920	-0.95120
H	-1.02651	0.15890	-1.46900
H	-2.29411	2.27830	-2.02920
H	-0.72691	3.08460	-1.75040
H	-2.18541	3.66700	-0.92160
H	-0.27021	1.98490	1.49730
H	-2.92171	2.02410	1.45590
H	-5.24631	0.67340	1.68140
H	-7.40261	-1.32460	-0.65530
H	-7.29521	-1.11030	1.10640
H	-6.61260	-2.55450	0.32550

Conformer 3
Energy: -1227.88754 Hartree (Rel: 0.2 kcal/mol)
XYZ coordinates for conf 3:

C	-4.68709	1.74101	-1.23000
C	-4.23230	1.03181	-0.01970
C	-4.48340	-0.46379	-0.05580
C	-3.65449	1.70121	1.01750
C	-3.42629	3.19581	1.07280
C	-3.17280	1.02221	2.27670
C	-3.51011	-1.25029	-0.97560
C	-2.08301	-1.24530	-0.51900
C	-1.64861	-1.95890	0.70780
O	-0.29191	-1.78251	0.85670
C	0.22359	-0.98151	-0.24090
C	-0.99610	-0.67560	-1.05850
O	-2.29991	-2.59950	1.50320
C	1.31429	-1.74681	-0.99900
C	2.50249	-2.24202	-0.13280
C	3.57009	-2.87052	-1.04580
O	2.07029	-3.29832	0.73080

C	3.15069	-1.15812	0.71600
C	3.47080	0.10258	0.38280
C	4.14960	1.11698	1.17000
C	4.26541	2.24318	0.43360
C	3.64011	1.96668	-0.87710
O	3.17390	0.65308	-0.85940
O	3.50111	2.67138	-1.84960
C	4.87521	3.56767	0.75340
O	-5.22050	1.18351	-2.18320
H	-4.53299	2.83351	-1.26860
H	-5.48960	-0.62519	-0.45680
H	-4.46510	-0.90169	0.94320
H	-3.88389	3.60051	1.98490
H	-3.81459	3.76071	0.22670
H	-2.35069	3.40170	1.15200
H	-2.13860	1.32320	2.48940
H	-3.20950	-0.06610	2.24820
H	-3.77100	1.36291	3.13340
H	-3.85931	-2.29009	-1.01520
H	-3.57220	-0.84749	-1.99000
H	0.64470	-0.07381	0.20670
H	-0.95160	-0.08120	-1.96460
H	0.86849	-2.62931	-1.47160
H	1.68220	-1.08901	-1.79190
H	3.99589	-2.12182	-1.72010
H	3.12068	-3.67502	-1.63800
H	4.37449	-3.29692	-0.43860
H	1.24099	-3.00701	1.15030
H	3.44729	-1.48252	1.70970
H	4.49410	0.95078	2.18360
H	5.68481	3.80247	0.05200
H	5.27921	3.58237	1.76890
H	4.13382	4.37028	0.66050

Conformer 4

Energy: -1227.88839 Hartree (Rel: 0.2 kcal/mol)

XYZ coordinates for conf 4:

C	-2.39873	1.10651	1.26345
C	-3.66790	0.67874	0.67477
C	-3.84513	-0.81977	0.50755
C	-4.60407	1.59756	0.29776
C	-4.48343	3.09510	0.45644
C	-5.90667	1.20632	-0.35369
C	-3.41152	-1.39111	-0.87246
C	-1.92900	-1.45199	-1.09967
C	-1.13278	-0.30025	-1.59152
O	0.17950	-0.68228	-1.68742
C	0.30583	-2.08128	-1.34503
C	-1.08110	-2.47633	-0.92948
O	-1.49864	0.81786	-1.89016
C	1.45704	-2.35524	-0.37117
C	1.40121	-1.70823	1.04111
C	2.47405	-2.37703	1.92426
O	0.15300	-2.00510	1.67188
C	1.62977	-0.20593	1.00017
C	2.70389	0.44671	0.52891
C	2.96615	1.87293	0.49399
C	4.17385	2.08683	-0.07292
C	4.72535	0.76170	-0.42129
O	3.79709	-0.20172	-0.03689
O	5.77415	0.45570	-0.94229
C	4.91731	3.35108	-0.35304
O	-1.49622	0.34030	1.60559
H	-2.24342	2.18538	1.41137

H	-4.89254	-1.09098	0.66682
H	-3.27028	-1.32660	1.28856
H	-4.42355	3.56406	-0.53461
H	-3.63077	3.43728	1.04108
H	-5.39360	3.48536	0.92928
H	-5.98257	0.14848	-0.60418
H	-6.04913	1.78620	-1.27460
H	-6.74654	1.46595	0.30567
H	-3.81583	-2.40633	-0.95591
H	-3.86906	-0.79320	-1.66977
H	0.55950	-2.60531	-2.27866
H	-1.32166	-3.47712	-0.58934
H	1.47903	-3.44449	-0.23596
H	2.39582	-2.07529	-0.85838
H	3.46961	-2.27040	1.48594
H	2.24176	-3.44215	2.03216
H	2.47303	-1.92083	2.91862
H	-0.49117	-1.29586	1.48801
H	0.84279	0.41341	1.42132
H	2.27231	2.61438	0.87145
H	5.89200	3.35007	0.14972
H	4.35316	4.22496	-0.01651
H	5.11529	3.45703	-1.42656

Conformer 5

Energy: -1227.88843 Hartree (Rel: 0.4 kcal/mol)

XYZ coordinates for conf 5:

C	-1.97932	-1.53324	1.47350
C	-2.96966	-1.63271	0.39261
C	-2.40047	-1.93329	-0.98417
C	-4.29956	-1.45468	0.63861
C	-4.91440	-1.11281	1.97425
C	-5.32897	-1.54889	-0.45920
C	-2.42621	-0.74510	-1.98311
C	-1.84083	0.52551	-1.43792
C	-2.69934	1.64750	-0.97889
O	-1.89728	2.66448	-0.54303
C	-0.50668	2.27242	-0.65402
C	-0.56669	0.89922	-1.25519
O	-3.91080	1.73519	-0.97286
C	0.12700	2.40053	0.74179
C	1.60653	1.96877	0.83015
C	2.22279	2.51132	2.13897
O	2.25247	2.56497	-0.29956
C	1.72391	0.45333	0.83551
C	2.76978	-0.26406	0.39728
C	2.96889	-1.70036	0.38854
C	4.17859	-1.97583	-0.14661
C	4.80205	-0.68437	-0.50277
O	3.90465	0.32888	-0.15416
O	5.87321	-0.43145	-1.00085
C	4.86963	-3.27662	-0.39067
O	-0.76903	-1.69112	1.32356
H	-2.36169	-1.31102	2.48406
H	-2.93008	-2.77523	-1.44625
H	-1.36281	-2.24811	-0.84544
H	-5.58156	-1.92553	2.29070
H	-5.54560	-0.22201	1.86025
H	-4.20804	-0.92092	2.78089
H	-6.27011	-1.95754	-0.07463
H	-5.00406	-2.15940	-1.30480
H	-5.54153	-0.53895	-0.83537
H	-1.87860	-1.04784	-2.88352
H	-3.45589	-0.53676	-2.28919

H	-0.01995	2.97713	-1.33597
H	0.32438	0.33475	-1.49985
H	0.04976	3.45310	1.03484
H	-0.46632	1.81824	1.45511
H	1.70657	2.11542	3.02051
H	2.15380	3.60430	2.15066
H	3.27785	2.22485	2.20471
H	3.15242	2.20123	-0.35812
H	0.90835	-0.11776	1.27168
H	2.22837	-2.39871	0.75911
H	5.08960	-3.40703	-1.45710
H	4.25701	-4.11779	-0.05628
H	5.83012	-3.31204	0.13728

Conformer 6
Energy: -1227.88657 Hartree (Rel: 0.4 kcal/mol)
XYZ coordinates for conf 6:

C	-3.58287	0.79165	1.60486
C	-4.28271	-0.22242	0.79840
C	-3.88178	-1.66124	1.07911
C	-5.20366	0.13501	-0.14040
C	-5.60770	1.54783	-0.48828
C	-5.91156	-0.88652	-0.99466
C	-2.95612	-2.30503	0.01101
C	-1.71412	-1.50766	-0.25953
C	-1.63369	-0.54767	-1.38773
O	-0.40147	0.06492	-1.34837
C	0.36152	-0.43628	-0.21701
C	-0.54920	-1.45401	0.40067
O	-2.44515	-0.29056	-2.24918
C	0.73777	0.70857	0.73206
C	1.49313	1.89358	0.07534
C	1.91641	2.89381	1.16555
O	0.60905	2.61949	-0.78563
C	2.71812	1.48234	-0.72827
C	3.68014	0.59904	-0.41665
C	4.88003	0.24867	-1.15603
C	5.56536	-0.68919	-0.46710
C	4.79388	-0.96938	0.76242
O	3.65823	-0.16151	0.74743
O	5.01141	-1.73388	1.67351
C	6.84980	-1.38691	-0.77036
O	-2.71688	0.53709	2.43657
H	-3.87118	1.84448	1.44285
H	-4.76843	-2.29693	1.18462
H	-3.36026	-1.67618	2.03990
H	-5.47555	1.70390	-1.56689
H	-5.05939	2.33261	0.03100
H	-6.67764	1.68664	-0.28405
H	-6.95467	-0.59391	-1.16234
H	-5.89739	-1.89365	-0.57334
H	-5.42829	-0.92690	-1.98051
H	-2.68242	-3.30893	0.35552
H	-3.50139	-2.42219	-0.93119
H	1.26548	-0.89669	-0.63192
H	-0.26572	-2.02447	1.27840
H	-0.17487	1.10932	1.18675
H	1.34824	0.27818	1.53140
H	2.63351	2.43845	1.85458
H	1.03441	3.21988	1.72735
H	2.37809	3.77294	0.70534
H	0.14584	1.96381	-1.33723
H	2.85203	2.03031	-1.65696
H	5.14454	0.69574	-2.10669

```
H          7.58806       -1.20358        0.01955
H          7.26769       -1.04984       -1.72251
H          6.70126       -2.47234       -0.81864
```

Conformer 7

```
Energy: -1227.88708 Hartree (Rel:  0.5 kcal/mol)
XYZ coordinates for conf 7:
C         -3.14601        1.26238        1.50120
C         -4.08601        0.93228        0.41440
C         -4.39330       -0.54682        0.26310
C         -4.61461        1.90298       -0.38310
C         -4.34231        3.38528       -0.26450
C         -5.56381        1.59618       -1.51530
C         -3.53350       -1.27132       -0.80890
C         -2.07750       -1.31712       -0.46170
C         -1.56610       -2.21942        0.60070
O         -0.21090       -2.01161        0.72640
C          0.22660       -1.01381       -0.23350
C         -1.03570       -0.63011       -0.94810
O         -2.15340       -3.03782        1.26990
C          1.32060       -1.58891       -1.13960
C          2.57020       -2.13721       -0.40000
C          3.62810       -2.56581       -1.43210
O          2.23170       -3.32801        0.31690
C          3.18890       -1.14211        0.57210
C          3.48580        0.15209        0.37290
C          4.12540        1.09989        1.26800
C          4.22609        2.29499        0.64680
C          3.63209        2.13299       -0.69700
O          3.19840        0.81419       -0.81610
O          3.49409        2.92549       -1.60000
C          4.79539        3.59589        1.10740
O         -2.61950        0.42828        2.22900
H         -2.91431        2.32998        1.65930
H         -5.44720       -0.69972        0.01260
H         -4.21990       -1.03362        1.22630
H         -3.97891        3.77108       -1.22580
H         -3.62301        3.66788        0.50270
H         -5.28201        3.91428       -0.05690
H         -6.48141        2.18988       -1.40870
H         -5.84240        0.54498       -1.59090
H         -5.11361        1.89838       -2.47090
H         -3.90540       -2.29952       -0.90010
H         -3.66330       -0.78732       -1.78280
H          0.62370       -0.17331        0.34810
H         -1.05040        0.11048       -1.74030
H          0.90130       -2.41321       -1.72760
H          1.62050       -0.79751       -1.83290
H          3.98700       -1.70491       -2.00320
H          3.19530       -3.30021       -2.12000
H          4.47760       -3.03001       -0.92170
H          1.39750       -3.15421        0.78930
H          3.47260       -1.56041        1.53380
H          4.45470        0.84479        2.26800
H          5.61079        3.91869        0.44880
H          5.18109        3.51999        2.12730
H          4.03479        4.38539        1.07940
```

Conformer 8

```
Energy: -1227.88730 Hartree (Rel:  0.5 kcal/mol)
XYZ coordinates for conf 8:
C         -6.14799        2.23929        0.31000
C         -5.79409        0.92439       -0.24930
C         -4.30559        0.64209       -0.30710
```

C	-6.74559	0.02909	-0.63550
C	-8.23679	0.27339	-0.61400
C	-6.39919	-1.34721	-1.15160
C	-3.76409	0.02729	1.01010
C	-2.33339	-0.39701	0.88970
C	-1.94279	-1.56381	0.05700
O	-0.58569	-1.71260	0.11760
C	-0.01639	-0.68470	0.96580
C	-1.21039	0.10709	1.41730
O	-2.64549	-2.31191	-0.59230
C	1.02251	0.14940	0.20630
C	2.39361	-0.53560	-0.00060
C	2.31701	-1.78140	-0.90830
O	2.82151	-0.90490	1.31330
C	3.33021	0.47890	-0.63980
C	4.66031	0.54430	-0.47870
C	5.62961	1.45760	-1.05510
C	6.86281	1.13410	-0.60920
C	6.70931	-0.03040	0.28830
O	5.35081	-0.35090	0.33560
O	7.52761	-0.66429	0.91040
C	8.19721	1.74371	-0.88520
O	-5.32249	3.05169	0.71410
H	-7.22159	2.49079	0.37190
H	-3.78169	1.58649	-0.48310
H	-4.06709	-0.02311	-1.13980
H	-8.66149	0.01319	-1.59220
H	-8.53689	1.29459	-0.38130
H	-8.71259	-0.39601	0.11520
H	-5.36589	-1.64741	-0.97260
H	-6.58789	-1.39681	-2.23380
H	-7.05879	-2.09461	-0.69260
H	-3.87519	0.75319	1.82150
H	-4.36989	-0.85041	1.26800
H	0.47351	-1.19220	1.80440
H	-1.12389	0.96149	2.08010
H	0.60041	0.45400	-0.75780
H	1.19961	1.06030	0.79100
H	1.95641	-1.52420	-1.91030
H	1.64071	-2.52380	-0.47850
H	3.31431	-2.22340	-1.00860
H	3.74231	-1.21110	1.25660
H	2.89231	1.20140	-1.32380
H	5.36151	2.25990	-1.73180
H	8.87871	1.00511	-1.32400
H	8.66441	2.09311	0.04330
H	8.11101	2.58971	-1.57180

Conformer 9

Energy: -1227.88809 Hartree (Rel: 0.5 kcal/mol)

XYZ coordinates for conf 9:

C	4.00114	2.78432	0.47669
C	4.40587	1.37551	0.34242
C	3.27509	0.37662	0.49044
C	5.69131	1.02090	0.06308
C	6.85298	1.97758	-0.07394
C	6.10804	-0.41655	-0.13759
C	2.54730	0.09556	-0.85027
C	1.53161	-0.99625	-0.71994
C	1.94024	-2.41173	-0.53022
O	0.81793	-3.18320	-0.43548
C	-0.36257	-2.34776	-0.53780
C	0.19195	-0.96570	-0.73049
O	3.05761	-2.88497	-0.46916

C	-1.21772	-2.59295	0.71581
C	-2.60077	-1.90573	0.70988
C	-3.46192	-2.48581	1.85542
O	-3.18381	-2.23169	-0.55331
C	-2.47101	-0.40647	0.92787
C	-3.28570	0.54781	0.45073
C	-3.25677	1.98572	0.64734
C	-4.28585	2.54546	-0.02484
C	-5.02405	1.44784	-0.68459
O	-4.37418	0.25163	-0.36596
O	-6.00884	1.46125	-1.38241
C	-4.70908	3.97039	-0.16025
O	2.84647	3.14449	0.68225
H	4.79049	3.55003	0.37499
H	2.54500	0.78509	1.19565
H	3.63831	-0.56539	0.90689
H	7.67854	1.64015	0.56612
H	6.63441	3.01369	0.18224
H	7.23366	1.95433	-1.10381
H	5.27719	-1.11220	-0.26226
H	6.71066	-0.75192	0.71883
H	6.75853	-0.49674	-1.01781
H	2.07008	1.01495	-1.20389
H	3.28917	-0.20096	-1.60232
H	-0.92214	-2.67658	-1.41940
H	-0.44002	-0.09704	-0.87103
H	-1.37785	-3.67429	0.78346
H	-0.65472	-2.28873	1.60536
H	-3.01018	-2.29698	2.83562
H	-3.57060	-3.56690	1.71915
H	-4.45698	-2.02862	1.84452
H	-3.98556	-1.69400	-0.66827
H	-1.67809	-0.07062	1.59109
H	-2.50730	2.48936	1.24569
H	-5.73115	4.10910	0.21189
H	-4.71118	4.27630	-1.21323
H	-4.04225	4.63525	0.39452

Conformer 10
Energy: -1227.88819 Hartree (Rel: 0.6 kcal/mol)
XYZ coordinates for conf 10:

C	-4.90124	-0.58513	1.56522
C	-4.25267	-0.21022	0.29767
C	-3.11001	-1.11449	-0.12139
C	-4.63568	0.89187	-0.40543
C	-5.77741	1.81174	-0.03913
C	-3.94112	1.32151	-1.67577
C	-1.74555	-0.66410	0.46263
C	-0.60182	-1.45388	-0.09573
C	-0.17255	-1.29596	-1.50533
O	0.87671	-2.14291	-1.74534
C	1.17441	-2.89692	-0.54533
C	0.20039	-2.36769	0.46785
O	-0.61332	-0.56374	-2.36929
C	2.67173	-2.83355	-0.21321
C	3.22987	-1.50859	0.34866
C	4.76114	-1.64736	0.52629
O	2.61674	-1.35134	1.62899
C	2.96643	-0.35097	-0.59607
C	2.68632	0.92002	-0.26945
C	2.46209	2.07271	-1.12091
C	2.21294	3.15315	-0.34894
C	2.27624	2.70826	1.05792
O	2.56524	1.34083	1.05436

O	2.12759	3.31647	2.09079
C	1.91531	4.57008	-0.71325
O	-4.55842	-1.54429	2.24837
H	-5.73718	0.04782	1.91254
H	-3.03593	-1.16749	-1.20991
H	-3.31946	-2.12538	0.24090
H	-5.38862	2.81221	0.19324
H	-6.38754	1.48315	0.80153
H	-6.43892	1.93325	-0.90686
H	-2.99208	0.81684	-1.86169
H	-3.75694	2.40326	-1.65208
H	-4.59935	1.14293	-2.53834
H	-1.58994	0.39626	0.22675
H	-1.76887	-0.75520	1.55313
H	0.94139	-3.94613	-0.77528
H	0.17535	-2.72169	1.49076
H	2.87036	-3.60708	0.53790
H	3.22538	-3.11484	-1.11605
H	5.27123	-1.79046	-0.43265
H	4.97892	-2.50436	1.17283
H	5.16280	-0.74433	0.99746
H	2.69182	-0.42045	1.89940
H	3.06622	-0.55930	-1.65671
H	2.49281	2.02726	-2.20268
H	0.94018	4.87698	-0.31648
H	1.90810	4.70579	-1.79775
H	2.66091	5.24805	-0.28081

Conformer 11
Energy: -1227.88805 Hartree (Rel: 0.7 kcal/mol)
XYZ coordinates for conf 11:

C	-3.63450	-0.51072	2.02390
C	-3.23251	-0.51501	0.60730
C	-1.74060	-0.64851	0.37330
C	-4.14921	-0.45222	-0.39810
C	-5.64111	-0.29622	-0.21400
C	-3.75751	-0.52702	-1.85510
C	-1.27070	-2.12651	0.34060
C	0.16800	-2.25230	-0.05490
C	0.60590	-2.01760	-1.45070
O	1.96450	-2.18019	-1.52110
C	2.47770	-2.53319	-0.21380
C	1.26270	-2.52529	0.66850
O	-0.05520	-1.72940	-2.42870
C	3.65870	-1.63329	0.17660
C	3.35239	-0.16959	0.55790
C	4.68609	0.56212	0.84350
O	2.58499	-0.24159	1.76000
C	2.64649	0.56001	-0.56970
C	1.71779	1.52221	-0.45980
C	1.04448	2.28790	-1.49130
C	0.19038	3.15940	-0.91120
C	0.29968	2.97110	0.54980
O	1.23869	1.95921	0.77440
O	-0.25792	3.52660	1.46540
C	-0.73412	4.16880	-1.50710
O	-2.84570	-0.63111	2.95500
H	-4.71171	-0.40342	2.24420
H	-1.21301	-0.14231	1.18730
H	-1.44831	-0.15731	-0.55760
H	-6.15600	-1.20213	-0.56120
H	-6.00151	0.52397	-0.84850
H	-5.96391	-0.09433	0.80680
H	-2.74110	-0.88341	-2.02810

H	-3.85711	0.46508	-2.31910
H	-4.44910	-1.18662	-2.39430
H	-1.43240	-2.57731	1.32460
H	-1.88510	-2.67991	-0.38070
H	2.86971	-3.55609	-0.30290
H	1.31201	-2.74139	1.72830
H	4.14520	-2.09528	1.04370
H	4.38220	-1.65628	-0.64620
H	5.31659	0.62452	-0.05030
H	5.23599	0.02932	1.62690
H	4.48229	1.58012	1.19140
H	2.12569	0.60551	1.88990
H	2.96619	0.31921	-1.57870
H	1.22678	2.14771	-2.54980
H	-0.49713	5.17570	-1.14330
H	-1.77212	3.96419	-1.21860
H	-0.66942	4.16790	-2.59820

Conformer 12
Energy: -1227.88783 Hartree (Rel: 0.7 kcal/mol)
XYZ coordinates for conf 12:

C	-3.06648	-2.57143	-1.55822
C	-2.84646	-2.00972	-0.21326
C	-3.94929	-1.07490	0.24688
C	-1.74921	-2.34786	0.52198
C	-0.68621	-3.33760	0.10092
C	-1.47107	-1.78229	1.89377
C	-3.92757	0.32686	-0.41934
C	-2.79268	1.21134	0.00004
C	-2.69872	1.77082	1.37230
O	-1.60149	2.58600	1.44195
C	-0.92038	2.59600	0.16414
C	-1.74891	1.68417	-0.69588
O	-3.42450	1.58860	2.32855
C	0.54105	2.18896	0.41226
C	1.47612	2.22110	-0.82836
C	1.48765	3.59049	-1.52003
O	1.02919	1.29513	-1.82588
C	2.88511	1.89120	-0.35206
C	3.36396	0.66086	-0.11728
C	4.67440	0.22490	0.32644
C	4.68448	-1.12269	0.42170
C	3.33890	-1.59497	0.03479
O	2.57238	-0.47440	-0.28857
O	2.87554	-2.70988	-0.01941
C	5.76531	-2.06906	0.82689
O	-4.04683	-2.31606	-2.25019
H	-2.29572	-3.25403	-1.95667
H	-3.95472	-0.95617	1.33140
H	-4.90474	-1.53507	-0.02732
H	0.29384	-2.84793	0.03588
H	-0.87187	-3.84535	-0.84467
H	-0.58817	-4.10862	0.87701
H	-1.53230	-2.58144	2.64581
H	-2.14169	-0.97854	2.19635
H	-0.44112	-1.40412	1.93630
H	-3.92086	0.20802	-1.50626
H	-4.86687	0.83018	-0.15462
H	-0.96541	3.62732	-0.20664
H	-1.49809	1.48395	-1.72943
H	0.94279	2.85845	1.18195
H	0.55148	1.17623	0.83233
H	1.74193	4.38578	-0.81165
H	0.51038	3.80946	-1.95873

H	2.22558	3.59138	-2.32806
H	1.14539	0.39574	-1.47351
H	3.56242	2.72027	-0.16670
H	5.48904	0.90735	0.53597
H	5.45401	-2.66688	1.69178
H	6.68259	-1.53367	1.08449
H	5.98772	-2.77437	0.01733

Conformer 13

Energy: -1227.88682 Hartree (Rel: 0.8 kcal/mol)

XYZ coordinates for conf 13:

C	6.76410	-0.90862	-0.22310
C	5.86290	0.21988	0.06320
C	4.47860	-0.18202	0.53330
C	6.24750	1.51258	-0.12840
C	7.62060	1.95997	-0.57350
C	5.31310	2.67668	0.10000
C	3.49940	-0.43251	-0.64330
C	2.10030	-0.67921	-0.17050
C	1.28390	0.41109	0.42200
O	0.06400	-0.09951	0.77930
C	0.00419	-1.50811	0.44550
C	1.35429	-1.79131	-0.15210
O	1.56690	1.57799	0.59560
C	-1.15731	-1.81090	-0.50550
C	-2.60031	-1.62660	0.02670
C	-2.82131	-2.35390	1.37050
O	-3.39171	-2.25230	-0.98990
C	-2.94440	-0.15460	0.19050
C	-4.16010	0.39470	0.04940
C	-4.58610	1.77281	0.20370
C	-5.91340	1.85711	-0.03330
C	-6.38620	0.49441	-0.35270
O	-5.28320	-0.36029	-0.28930
O	-7.48770	0.08091	-0.62680
C	-6.84009	3.02731	-0.01160
O	6.43669	-2.08552	-0.11210
H	7.78590	-0.66673	-0.56520
H	4.05760	0.57488	1.19860
H	4.56519	-1.10952	1.10730
H	7.55380	2.43967	-1.55920
H	8.37010	1.17137	-0.63160
H	7.99381	2.72757	0.11690
H	5.61371	3.22318	1.00560
H	4.26410	2.39648	0.20450
H	5.39721	3.39058	-0.72920
H	3.49840	0.44659	-1.29990
H	3.85229	-1.28352	-1.23390
H	-0.12321	-2.05180	1.38950
H	1.63569	-2.77571	-0.51050
H	-1.03701	-1.21780	-1.41900
H	-1.07281	-2.86520	-0.79470
H	-2.26831	-1.88780	2.19300
H	-2.51351	-3.40220	1.28350
H	-3.88521	-2.32590	1.62800
H	-4.32211	-2.01839	-0.83130
H	-2.14070	0.51150	0.48650
H	-3.91050	2.57721	0.46800
H	-7.31709	3.16411	-0.98940
H	-6.30869	3.94641	0.24820
H	-7.64640	2.87211	0.71530

Conformer 14
Energy: -1227.88911 Hartree (Rel: 1.0 kcal/mol)
XYZ coordinates for conf 14:

C	-5.59239	2.43290	0.60550
C	-5.54880	1.04730	0.10960
C	-4.36070	0.23920	0.59370
C	-6.48780	0.56300	-0.75040
C	-7.69850	1.32190	-1.24270
C	-6.42190	-0.83580	-1.31600
C	-3.11520	0.40360	-0.31630
C	-2.00590	-0.52450	0.07210
C	-2.11280	-1.98780	-0.15280
O	-0.97210	-2.58750	0.30370
C	-0.07370	-1.58320	0.83840
C	-0.81920	-0.29240	0.64930
O	-3.01920	-2.62600	-0.65020
C	1.26720	-1.71591	0.09980
C	2.45260	-0.93251	0.70530
C	2.81280	-1.41761	2.12720
O	2.04740	0.43889	0.74240
C	3.65810	-1.14241	-0.20090
C	4.68290	-0.29201	-0.36140
C	5.87500	-0.40341	-1.18140
C	6.63190	0.70259	-1.01520
C	5.92331	1.57549	-0.05530
O	4.74230	0.92519	0.31370
O	6.21381	2.65649	0.39670
C	7.94041	1.09429	-1.61710
O	-4.74149	2.91950	1.34280
H	-6.44049	3.06090	0.27930
H	-4.61510	-0.82030	0.66780
H	-4.09640	0.58230	1.59840
H	-8.59850	0.71450	-1.08040
H	-7.62420	1.47700	-2.32730
H	-7.86159	2.29130	-0.77310
H	-7.20800	-1.45740	-0.86350
H	-5.46440	-1.33650	-1.16650
H	-6.63350	-0.81280	-2.39260
H	-3.40320	0.19350	-1.35420
H	-2.77009	1.44130	-0.27540
H	0.05190	-1.81290	1.90320
H	-0.39710	0.65860	0.94570
H	1.52730	-2.78011	0.08350
H	1.12380	-1.39581	-0.93850
H	3.07850	-2.48031	2.13420
H	1.97550	-1.25971	2.81430
H	3.67060	-0.85081	2.50400
H	2.82470	0.97719	0.97010
H	3.71800	-2.07921	-0.74850
H	6.08460	-1.25781	-1.81340
H	7.84491	2.03119	-2.17890
H	8.31190	0.31929	-2.29240
H	8.69080	1.26788	-0.83670

Conformer 15
Energy: -1227.88722 Hartree (Rel: 1.2 kcal/mol)
XYZ coordinates for conf 15:

C	4.09554	-1.29699	0.85509
C	3.19960	-0.73967	-0.16878
C	1.72114	-0.83142	0.15306
C	3.67437	-0.19140	-1.32175
C	5.13082	-0.03010	-1.68313
C	2.73664	0.35685	-2.37684
C	1.12583	-2.24066	-0.10946

C	-0.36193	-2.26648	0.07265
C	-1.00904	-2.07445	1.39380
O	-2.37171	-2.10492	1.22337
C	-2.68217	-2.33465	-0.16983
C	-1.33856	-2.39592	-0.83586
O	-0.50794	-1.91181	2.48597
C	-3.69229	-1.30169	-0.68956
C	-3.19881	0.14571	-0.90409
C	-4.39684	1.01736	-1.35421
O	-2.24360	0.07309	-1.96338
C	-2.64631	0.74077	0.37667
C	-1.59784	1.56690	0.51479
C	-1.06891	2.20897	1.70222
C	0.00175	2.96090	1.36276
C	0.19424	2.81164	-0.09226
O	-0.80441	1.95698	-0.56556
O	1.02289	3.28164	-0.83770
C	0.90462	3.81059	2.19462
O	3.70219	-1.80853	1.89812
H	5.18188	-1.24942	0.65724
H	1.15560	-0.09453	-0.42199
H	1.57614	-0.60130	1.21302
H	5.32818	1.02262	-1.92593
H	5.35052	-0.60072	-2.59556
H	5.83873	-0.33509	-0.91307
H	1.87895	-0.29911	-2.55406
H	3.25887	0.49741	-3.32818
H	2.33946	1.33499	-2.07158
H	1.36798	-2.56417	-1.12827
H	1.59899	-2.94654	0.58248
H	-3.17386	-3.31644	-0.22067
H	-1.22518	-2.54490	-1.90249
H	-4.05514	-1.66198	-1.65953
H	-4.55044	-1.30068	-0.00822
H	-5.16603	1.08590	-0.57720
H	-4.84427	0.58567	-2.25605
H	-4.05226	2.03029	-1.58647
H	-1.68457	0.86777	-1.93606
H	-3.20051	0.52304	1.28445
H	-1.49330	2.07404	2.68954
H	0.89514	4.84942	1.84367
H	1.94119	3.46027	2.12412
H	0.60340	3.79426	3.24508

Conformer 16

Energy: -1227.88844 Hartree (Rel: 1.2 kcal/mol)

XYZ coordinates for conf 16:

C	-5.03470	2.45461	-0.39400
C	-4.90020	1.03561	-0.01820
C	-4.81930	0.09161	-1.20310
C	-4.86680	0.65001	1.28890
C	-4.97770	1.58071	2.47650
C	-4.72730	-0.78819	1.72540
C	-3.45200	0.10831	-1.93960
C	-2.30030	-0.39449	-1.12400
C	-2.17690	-1.81909	-0.72070
O	-1.02220	-1.97569	-0.00410
C	-0.33580	-0.70559	0.10400
C	-1.22940	0.24671	-0.63640
O	-2.92850	-2.74679	-0.94060
C	1.08560	-0.79319	-0.46710
C	2.12230	-1.47599	0.47500
C	1.77650	-2.93829	0.78230
O	2.12700	-0.80979	1.74180

C	3.47910	-1.41729	-0.21050
C	4.35030	-0.39809	-0.16530
C	5.65590	-0.24729	-0.77990
C	6.16460	0.95991	-0.45070
C	5.16760	1.63441	0.40680
O	4.08030	0.76711	0.55090
O	5.17850	2.72111	0.93230
C	7.46000	1.60871	-0.81000
O	-5.07840	2.84941	-1.55450
H	-5.09740	3.19421	0.42340
H	-5.57180	0.40631	-1.93410
H	-5.05990	-0.93379	-0.91950
H	-5.78680	1.23670	3.13390
H	-5.16590	2.62621	2.23670
H	-4.05650	1.53021	3.07190
H	-4.51690	-1.48909	0.91870
H	-5.64750	-1.11260	2.23130
H	-3.92470	-0.87479	2.46960
H	-3.55030	-0.52389	-2.83160
H	-3.24340	1.12511	-2.28290
H	-0.27230	-0.45899	1.17090
H	-0.99220	1.29961	-0.74600
H	1.05170	-1.29649	-1.44030
H	1.43390	0.23151	-0.64760
H	1.69320	-3.52229	-0.13950
H	0.82590	-3.00199	1.31530
H	2.56010	-3.37579	1.40900
H	2.54920	0.05911	1.62690
H	3.76560	-2.27159	-0.81790
H	6.11450	-1.00989	-1.39740
H	8.04240	1.83821	0.09030
H	7.28770	2.56021	-1.32700
H	8.05890	0.96301	-1.45720

Conformer 17

Energy: -1227.88889 Hartree (Rel: 1.2 kcal/mol)

XYZ coordinates for conf 17:

C	5.53140	0.24871	-0.35090
C	4.37850	1.02411	0.14670
C	3.99610	0.69621	1.57790
C	3.75200	1.94471	-0.63680
C	4.15280	2.30061	-2.05140
C	2.56050	2.75171	-0.17900
C	3.27761	-0.66979	1.75600
C	1.95921	-0.77390	1.04820
C	1.85421	-1.24540	-0.35700
O	0.52621	-1.21560	-0.72480
C	-0.28669	-0.77450	0.39460
C	0.71681	-0.50800	1.47700
O	2.71661	-1.61859	-1.11830
C	-1.33029	-1.83911	0.75260
C	-2.26149	-2.27331	-0.41000
C	-3.32988	-3.24141	0.12860
O	-1.51919	-3.02381	-1.37630
C	-2.95059	-1.11781	-1.12100
C	-3.54230	-0.03161	-0.59880
C	-4.25780	1.03798	-1.27200
C	-4.69200	1.92638	-0.35200
C	-4.24580	1.43189	0.96780
O	-3.55320	0.23919	0.76530
O	-4.39450	1.89359	2.07530
C	-5.47140	3.19098	-0.49910
O	6.16401	-0.54108	0.34050
H	5.82830	0.39702	-1.40340

H	3.38230	1.48521	2.01780
H	4.91820	0.64291	2.16700
H	4.25600	3.39011	-2.13810
H	3.35350	2.00931	-2.74570
H	5.08100	1.85021	-2.39980
H	2.84339	3.80801	-0.06840
H	2.12600	2.41400	0.76140
H	1.77580	2.72370	-0.94610
H	3.94011	-1.46059	1.39170
H	3.12441	-0.83639	2.82810
H	-0.78690	0.14760	0.07590
H	0.42491	-0.15950	2.46210
H	-0.81529	-2.73870	1.10800
H	-1.92839	-1.44401	1.57890
H	-3.98129	-2.74061	0.85050
H	-2.84258	-4.09441	0.61340
H	-3.94158	-3.61731	-0.69730
H	-0.69689	-2.53230	-1.55280
H	-3.01989	-1.23081	-2.19940
H	-4.39930	1.07458	-2.34540
H	-6.40950	3.13748	0.06620
H	-5.70770	3.39058	-1.54750
H	-4.90951	4.04358	-0.09920

Conformer 18
Energy: -1227.88854 Hartree (Rel: 1.3 kcal/mol)
XYZ coordinates for conf 18:

C	5.48850	2.28561	0.44760
C	5.15280	0.97721	-0.14330
C	3.67300	0.76431	-0.41109
C	6.10440	0.03661	-0.40690
C	7.57880	0.25411	-0.15760
C	5.77360	-1.31909	-0.97640
C	2.93990	0.06601	0.76470
C	1.52100	-0.27830	0.43030
C	1.20190	-1.33380	-0.56380
O	-0.16480	-1.42020	-0.68119
C	-0.79780	-0.47110	0.22010
C	0.36020	0.22040	0.87701
O	1.94560	-2.04299	-1.20569
C	-1.73270	-1.19830	1.19311
C	-2.83700	-2.06340	0.52941
C	-3.77010	-2.62100	1.61881
O	-2.25080	-3.21070	-0.09340
C	-3.66610	-1.31850	-0.50709
C	-4.20930	-0.09300	-0.43029
C	-5.05310	0.60230	-1.38650
C	-5.37280	1.81740	-0.89129
C	-4.71741	1.93030	0.42911
O	-4.02390	0.74420	0.66441
O	-4.71371	2.83220	1.23411
C	-6.20601	2.92019	-1.45520
O	6.58969	2.68061	0.81100
H	4.60909	2.95941	0.55980
H	3.52090	0.18081	-1.32239
H	3.19340	1.73561	-0.58730
H	8.16310	-0.57269	-0.57300
H	7.78960	0.32011	0.91641
H	7.93080	1.19611	-0.58659
H	4.70740	-1.52389	-1.08150
H	6.21220	-2.09929	-0.33949
H	6.24910	-1.43249	-1.96110
H	3.47740	-0.85709	1.01460
H	2.96950	0.70511	1.65410

H	-1.37260	0.21920	-0.40819
H	0.22370	1.00390	1.61441
H	-1.13840	-1.86240	1.83071
H	-2.19200	-0.43850	1.83221
H	-4.30410	-1.81230	2.12581
H	-3.18360	-3.18260	2.35411
H	-4.50130	-3.29990	1.16941
H	-1.48670	-2.90070	-0.61140
H	-3.89140	-1.89070	-1.40280
H	-5.35530	0.17400	-2.33449
H	-5.62001	3.84249	-1.54689
H	-7.05061	3.14669	-0.79329
H	-6.59741	2.65649	-2.44109

Conformer 19

Energy: -1227.88665 Hartree (Rel: 1.5 kcal/mol)

XYZ coordinates for conf 19:

C	4.16394	2.72832	-0.76879
C	4.06310	1.59466	0.16857
C	4.77081	0.33917	-0.30544
C	3.39915	1.71416	1.35287
C	2.72371	2.97262	1.85198
C	3.27074	0.58574	2.34770
C	4.03608	-0.41978	-1.44366
C	2.71564	-1.01049	-1.05314
C	2.60399	-2.13389	-0.08715
O	1.28543	-2.47310	0.04392
C	0.47329	-1.61987	-0.79780
C	1.47304	-0.71659	-1.45981
O	3.47980	-2.70643	0.52877
C	-0.58907	-0.87992	0.02556
C	-1.82803	-1.74229	0.41255
C	-1.46743	-2.94349	1.29545
O	-2.40910	-2.29868	-0.77091
C	-2.80254	-0.84586	1.16254
C	-3.72177	-0.03751	0.61339
C	-4.69126	0.84573	1.23418
C	-5.41110	1.46117	0.27104
C	-4.90414	0.97589	-1.02991
O	-3.87543	0.06565	-0.76815
O	-5.23245	1.24529	-2.15979
C	-6.52382	2.45204	0.36079
O	4.76580	2.67647	-1.83601
H	3.66305	3.67032	-0.48430
H	4.96324	-0.35023	0.51760
H	5.74535	0.63663	-0.70734
H	2.86429	3.85701	1.23236
H	3.09147	3.20743	2.85928
H	1.64426	2.79755	1.95274
H	3.85878	0.81703	3.24718
H	3.58959	-0.38665	1.97438
H	2.22823	0.49847	2.68061
H	3.90003	0.25399	-2.29382
H	4.69513	-1.23290	-1.77500
H	-0.03231	-2.26563	-1.52628
H	1.18253	0.04881	-2.17160
H	-0.11810	-0.45136	0.91810
H	-0.95268	-0.04042	-0.58015
H	-0.95904	-2.61946	2.20906
H	-0.80566	-3.62658	0.75965
H	-2.37963	-3.48250	1.57023
H	-2.84003	-1.57978	-1.26498
H	-2.73923	-0.83895	2.24731
H	-4.79013	0.96070	2.30676

H	-6.26135	3.37962	-0.16191
H	-6.75664	2.69142	1.40148
H	-7.42954	2.06325	-0.11953

Conformer 20
Energy: -1227.88859 Hartree (Rel: 1.6 kcal/mol)
XYZ coordinates for conf 20:

C	-5.26060	1.63740	1.69430
C	-4.86311	1.13360	0.36960
C	-3.43451	0.63320	0.28570
C	-5.73841	1.08810	-0.67330
C	-7.16100	1.59721	-0.64450
C	-5.36381	0.51360	-2.01880
C	-3.29691	-0.85470	0.70310
C	-1.92101	-1.38620	0.44730
C	-1.43741	-1.63710	-0.93500
O	-0.15241	-2.09840	-0.87410
C	0.27679	-2.17500	0.50760
C	-0.92221	-1.70520	1.28070
O	-2.02171	-1.49200	-1.98960
C	1.53219	-1.32700	0.75070
C	2.85489	-1.93671	0.22210
C	2.87979	-2.11501	-1.30570
O	2.95379	-3.21761	0.86570
C	4.01439	-1.07181	0.69710
C	4.31919	0.18159	0.32520
C	5.40569	1.03499	0.77430
C	5.32330	2.22349	0.13880
C	4.14700	2.15279	-0.75530
O	3.57210	0.89259	-0.60530
O	3.68330	2.97249	-1.51290
C	6.17570	3.44579	0.22570
O	-4.51790	1.64540	2.67050
H	-6.29210	2.01721	1.80300
H	-2.81720	1.23420	0.96020
H	-3.03540	0.76320	-0.72250
H	-7.32410	2.26591	-1.49980
H	-7.44310	2.13681	0.25900
H	-7.85881	0.75891	-0.77280
H	-6.17591	-0.12330	-2.39170
H	-4.44351	-0.07210	-2.01630
H	-5.25070	1.32560	-2.75160
H	-3.55181	-0.95860	1.76230
H	-4.01611	-1.45300	0.12990
H	0.50799	-3.22600	0.71580
H	-0.92911	-1.64690	2.36390
H	1.38829	-0.32991	0.32440
H	1.63919	-1.20931	1.83560
H	2.75509	-1.15741	-1.81650
H	2.08019	-2.78791	-1.62290
H	3.84389	-2.53901	-1.61270
H	3.63269	-3.73291	0.40040
H	4.65359	-1.52261	1.45130
H	6.14239	0.72539	1.50570
H	6.59930	3.69469	-0.75470
H	6.99590	3.30719	0.93480
H	5.58220	4.31179	0.54270

Conformer 21
Energy: -1227.88889 Hartree (Rel: 1.6 kcal/mol)
XYZ coordinates for conf 21:

C	-5.30540	0.57349	-1.21991
C	-4.34720	0.89659	-0.14721
C	-4.31590	-0.12311	0.97579

C	-3.59001	2.02940	-0.19301
C	-3.63891	3.06750	-1.29261
C	-2.59891	2.40880	0.88030
C	-3.59740	-1.45151	0.61379
C	-2.12930	-1.31360	0.34899
C	-1.16490	-0.95760	1.42149
O	0.09670	-0.92180	0.87819
C	0.03540	-1.26069	-0.53021
C	-1.42770	-1.48020	-0.78070
O	-1.36660	-0.71620	2.59130
C	0.91391	-2.48119	-0.82820
C	2.41831	-2.32719	-0.45291
C	3.17301	-3.59239	-0.89750
O	2.58071	-2.27389	0.95759
C	3.01400	-1.12949	-1.17000
C	3.41480	0.04902	-0.66500
C	4.00780	1.17452	-1.37061
C	4.25159	2.16742	-0.49001
C	3.80039	1.68532	0.83430
O	3.30530	0.39481	0.67459
O	3.81219	2.22972	1.91349
C	4.84939	3.52242	-0.67531
O	-6.01550	-0.42661	-1.21870
H	-5.37940	1.27859	-2.06641
H	-5.35100	-0.38581	1.21900
H	-3.87070	0.28840	1.88240
H	-2.65671	3.13600	-1.77880
H	-3.83201	4.05450	-0.85250
H	-4.38551	2.89619	-2.06650
H	-2.42730	1.64060	1.63309
H	-2.93881	3.32010	1.39200
H	-1.63251	2.65980	0.42400
H	-3.73519	-2.14131	1.45650
H	-4.08680	-1.89841	-0.25561
H	0.41240	-0.38819	-1.07910
H	-1.81610	-1.75180	-1.75640
H	0.52241	-3.34779	-0.28320
H	0.82291	-2.69469	-1.90071
H	3.13571	-3.71939	-1.98411
H	2.72661	-4.47209	-0.42311
H	4.21981	-3.52478	-0.58640
H	2.02680	-1.55309	1.30309
H	3.14040	-1.23889	-2.24520
H	4.20850	1.17002	-2.43540
H	5.75349	3.63472	-0.06481
H	4.15099	4.30322	-0.35111
H	5.11159	3.70082	-1.72141

LIST OF FILES (technical info - delete in the final SI version
abimael-PkC-SS-Z-du8ml-chloroform_112.log abimael-PkC-SS-Z-du8ml-chloroform_35.log abimael-PkC-SS-Z-du8ml-chloroform_38.log abimael-PkC-SS-Z-du8ml-chloroform_80.log abimael-PkC-SS-Z-du8ml-chloroform_6.log abimael-PkC-SS-Z-du8ml-chloroform_41.log abimael-PkC-SS-Z-du8ml-chloroform_37.log abimael-PkC-SS-Z-du8ml-chloroform_129.log abimael-PkC-SS-Z-du8ml-chloroform_128.log abimael-PkC-SS-Z-du8ml-chloroform_220.log abimael-PkC-SS-Z-du8ml-chloroform_197.log abimael-PkC-SS-Z-du8ml-chloroform_228.log abimael-PkC-SS-Z-du8ml-chloroform_240.log abimael-PkC-SS-Z-du8ml-chloroform_127.log abimael-PkC-SS-Z-du8ml-chloroform_196.log abimael-PkC-SS-Z-du8ml-chloroform_124.log abimael-PkC-SS-Z-du8ml-chloroform_36.log abimael-PkC-SS-Z-du8ml-chloroform_148.log abimael-PkC-SS-Z-du8ml-chloroform_270.log abimael-PkC-SS-Z-du8ml-chloroform_52.log abimael-PkC-SS-Z-du8ml-chloroform_143.log

DU8ML data for 2-(2-((*S*)-5-((*R*,*Z*)-2-hydroxy-2-methyl-3-(4-methyl-5-oxofuran-2(5*H*)-ylidene)propyl)-2-oxo-2,5-dihydrofuran-3-yl)ethyl)-3-methylbut-2-enal (**10*S*,8*R*,6*Z* diastereomer**)

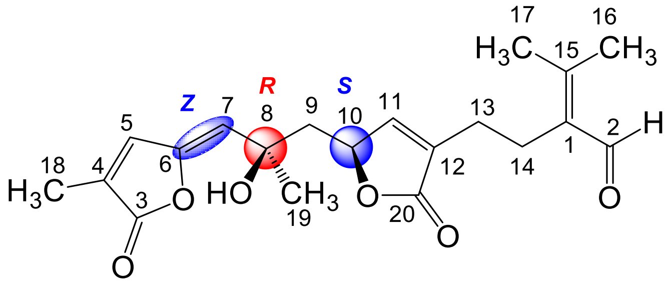

NMR parameters calculated for 10*S*,8*R*,6*Z* diastereomer vs experimental data of kallopterolide C (**3**):

	Conf1	Conf2	Conf3	Conf4	Conf5	Conf6	Conf7	Conf8	Conf9	Conf10	Conf11	Conf12	Conf13	Conf14
Rel energy (kcal/mol):	0.00	0.00	0.31	0.37	0.92	0.96	1.36	1.36	1.36	1.62	1.65	1.69	1.91	1.96

C-nom	iGau	Exp	Calc	diff		1	2	3	4	5	6	7	8	9	10	11	12	13	14	
C1-C	2	135.35	135.91	0.55	[	135.42	134.95	136.83	136.86	137.06	136.66	136.51	136.50	134.81	135.13	138.54	135.12	134.20	134.87	]
C2-CH	1	190.66	191.18	0.53	[	192.64	192.63	189.40	189.39	189.41	189.47	190.84	190.83	190.01	189.98	193.83	192.41	189.54	189.86	]
C3-C	22	170.02	168.94	-1.09	[	169.40	169.39	168.27	168.27	168.16	169.42	168.43	168.45	168.33	168.28	169.39	170.62	168.32	169.33	]
C4-C	21	131.27	129.89	-1.39	[	130.31	130.32	129.34	129.24	130.39	129.90	128.87	128.89	129.14	129.48	130.37	129.94	129.06	130.18	]
C5-CH	20	136.50	138.95	2.45	[	138.74	138.73	139.21	139.20	138.42	139.19	139.52	139.54	139.25	139.14	138.60	139.77	139.20	139.11	]
C6-C	19	149.51	145.80	-3.71	[	145.42	145.44	146.20	146.02	148.01	145.09	145.74	145.73	145.90	146.28	145.46	147.08	145.72	145.24	]
C7-CH	18	117.38	120.16	2.78	[	120.32	120.22	119.82	119.87	118.33	123.24	120.72	120.72	119.94	119.43	120.10	116.25	120.49	122.63	]
C8-C	15	72.72	72.88	0.16	[	72.50	72.44	73.12	73.15	74.21	73.74	73.22	73.22	73.18	73.13	72.51	72.15	73.07	73.78	]
C9-CH2	14	46.30	46.64	0.35	[	48.01	48.17	45.53	45.59	45.10	42.08	45.14	45.14	45.60	45.64	48.33	49.14	45.35	42.95	]
C10-CH	11	78.77	78.68	-0.09	[	78.89	78.86	78.30	78.26	79.81	79.45	77.66	77.66	77.99	78.04	78.84	78.69	77.90	78.88	]
C11-CH	12	148.56	150.63	2.07	[	153.00	152.53	148.19	148.14	150.47	147.70	146.14	146.17	150.11	150.25	150.49	153.51	150.61	149.73	]
C12-C	8	133.63	131.82	-1.81	[	130.15	130.25	133.94	133.88	131.95	133.67	133.56	133.56	132.90	132.92	131.36	130.25	132.93	132.18	]
C13-CH2	7	24.39	24.44	0.04	[	22.26	21.75	27.12	27.04	27.11	26.94	27.56	27.57	25.71	25.66	25.39	22.43	22.95	25.73	]
C14-CH2	3	23.39	25.02	1.63	[	24.77	24.87	25.62	25.53	25.47	25.59	23.40	23.39	24.17	24.41	24.80	25.69	25.07	24.18	]
C15-C	4	157.06	162.58	5.52	[	165.93	166.63	158.74	158.59	158.40	158.67	158.87	158.86	158.55	157.98	164.37	166.43	162.19	158.70	]
C16-CH3**	6	23.31	22.31	-1.00	[	22.73	22.97	21.71	21.65	21.77	21.65	21.85	21.85	21.79	22.01	22.46	22.86	22.82	22.06	]
C17-CH3**	5	19.38	17.30	-2.07	[	17.54	17.68	17.01	17.04	16.97	17.07	16.69	16.69	16.90	16.93	17.46	17.60	17.23	16.95	]
C18-CH3	25	10.83	10.83	0.00	[	10.85	10.84	10.82	10.82	10.86	10.77	10.81	10.81	10.84	10.81	10.85	10.78	10.82	10.78	]

C19-CH3	16	30.80	30.12	-0.68	[	30.20	30.18	30.71	30.70	30.57	24.90	30.76	30.76	30.71	30.63	30.25	31.46	30.76	24.66	]
C20-C	9	172.67	174.57	1.90	[	174.63	174.86	174.71	174.71	174.64	173.14	173.64	173.65	174.53	174.62	174.18	174.80	175.03	172.80	]

13C chem shifts: RMSD=2.02ppm (MAE=1.49) N=20 {-3.71 5.52}

Fractions: 0.243 0.242 0.145 0.131 0.052 0.048 0.025 0.025 0.025 0.016 0.015 0.014 0.010 0.009

NMR parameters calculated for 10*S*,8*R*,6*Z* diastereomer vs experimental data of kallopterolide D (**4**):

**C-nom**	**iGau**	**Exp**	**Calc**	**diff**		**1**	**2**	**3**	**4**	**5**	**6**	**7**	**8**	**9**	**10**	**11**	**12**	**13**	**14**	
C1-C	2	135.49	135.91	0.41	[	135.42	134.95	136.83	136.86	137.06	136.66	136.51	136.50	134.81	135.13	138.54	135.12	134.20	134.87	]
C2-CH	1	190.65	191.18	0.54	[	192.64	192.63	189.40	189.39	189.41	189.47	190.84	190.83	190.01	189.98	193.83	192.41	189.54	189.86	]
C3-C	22	169.95	168.94	-1.02	[	169.40	169.39	168.27	168.27	168.16	169.42	168.43	168.45	168.33	168.28	169.39	170.62	168.32	169.33	]
C4-C	21	129.26	129.89	0.62	[	130.31	130.32	129.34	129.24	130.39	129.90	128.87	128.89	129.14	129.48	130.37	129.94	129.06	130.18	]
C5-CH	20	138.63	138.95	0.32	[	138.74	138.73	139.21	139.20	138.42	139.19	139.52	139.54	139.25	139.14	138.60	139.77	139.20	139.11	]
C6-C	19	146.88	145.80	-1.08	[	145.42	145.44	146.20	146.02	148.01	145.09	145.74	145.73	145.90	146.28	145.46	147.08	145.72	145.24	]
C7-CH	18	118.26	120.16	1.90	[	120.32	120.22	119.82	119.87	118.33	123.24	120.72	120.72	119.94	119.43	120.10	116.25	120.49	122.63	]
C8-C	15	71.78	72.88	1.10	[	72.50	72.44	73.12	73.15	74.21	73.74	73.22	73.22	73.18	73.13	72.51	72.15	73.07	73.78	]
C9-CH2	14	45.85	46.64	0.80	[	48.01	48.17	45.53	45.59	45.10	42.08	45.14	45.14	45.60	45.64	48.33	49.14	45.35	42.95	]
C10-CH	11	78.34	78.68	0.34	[	78.89	78.86	78.30	78.26	79.81	79.45	77.66	77.66	77.99	78.04	78.84	78.69	77.90	78.88	]
C11-CH	12	149.56	150.63	1.07	[	153.00	152.53	148.19	148.14	150.47	147.70	146.14	146.17	150.11	150.25	150.49	153.51	150.61	149.73	]
C12-C	8	133.05	131.82	-1.23	[	130.15	130.25	133.94	133.88	131.95	133.67	133.56	133.56	132.90	132.92	131.36	130.25	132.93	132.18	]
C13-CH2	7	24.37	24.44	0.06	[	22.26	21.75	27.12	27.04	27.11	26.94	27.56	27.57	25.71	25.66	25.39	22.43	22.95	25.73	]
C14-CH2	3	23.38	25.02	1.63	[	24.77	24.87	25.62	25.53	25.47	25.59	23.40	23.39	24.17	24.41	24.80	25.69	25.07	24.18	]
C15-C	4	156.72	162.58	5.86	[	165.93	166.63	158.74	158.59	158.40	158.67	158.87	158.86	158.55	157.98	164.37	166.43	162.19	158.70	]
C16-CH3**	6	23.42	22.31	-1.11	[	22.73	22.97	21.71	21.65	21.77	21.65	21.85	21.85	21.79	22.01	22.46	22.86	22.82	22.06	]
C17-CH3**	5	19.35	17.30	-2.05	[	17.54	17.68	17.01	17.04	16.97	17.07	16.69	16.69	16.90	16.93	17.46	17.60	17.23	16.95	]
C18-CH3	25	10.51	10.83	0.32	[	10.85	10.84	10.82	10.82	10.86	10.77	10.81	10.81	10.84	10.81	10.85	10.78	10.82	10.78	]
C19-CH3	16	30.14	30.12	-0.02	[	30.20	30.18	30.71	30.70	30.57	24.90	30.76	30.76	30.71	30.63	30.25	31.46	30.76	24.66	]
C20-C	9	173.51	174.57	1.06	[	174.63	174.86	174.71	174.71	174.64	173.14	173.64	173.65	174.53	174.62	174.18	174.80	175.03	172.80	]

13C chem shifts: RMSD=1.66ppm (MAE=1.13) N=20 {-2.05 5.86}

Fractions: 0.243 0.242 0.145 0.131 0.052 0.048 0.025 0.025 0.025 0.016 0.015 0.014 0.010 0.009

NMR parameters calculated for 10*S*,8*R*,6*Z* diastereomer vs experimental data of kallopterolide E (**5**):

**C-nom**	**iGau**	**Exp**	**Calc**	**diff**		**1**	**2**	**3**	**4**	**5**	**6**	**7**	**8**	**9**	**10**	**11**	**12**	**13**	**14**	
C1-C	2	135.53	135.91	0.37	[	135.42	134.95	136.83	136.86	137.06	136.66	136.51	136.50	134.81	135.13	138.54	135.12	134.20	134.87	]
C2-CH	1	190.64	191.18	0.55	[	192.64	192.63	189.40	189.39	189.41	189.47	190.84	190.83	190.01	189.98	193.83	192.41	189.54	189.86	]
C3-C	22	170.05	168.94	-1.11	[	169.40	169.39	168.27	168.27	168.16	169.42	168.43	168.45	168.33	168.28	169.39	170.62	168.32	169.33	]
C4-C	21	129.50	129.89	0.38	[	130.31	130.32	129.34	129.24	130.39	129.90	128.87	128.89	129.14	129.48	130.37	129.94	129.06	130.18	]
C5-CH	20	138.53	138.95	0.42	[	138.74	138.73	139.21	139.20	138.42	139.19	139.52	139.54	139.25	139.14	138.60	139.77	139.20	139.11	]
C6-C	19	145.96	145.80	-0.16	[	145.42	145.44	146.20	146.02	148.01	145.09	145.74	145.73	145.90	146.28	145.46	147.08	145.72	145.24	]
C7-CH	18	119.20	120.16	0.96	[	120.32	120.22	119.82	119.87	118.33	123.24	120.72	120.72	119.94	119.43	120.10	116.25	120.49	122.63	]
C8-C	15	72.05	72.88	0.84	[	72.50	72.44	73.12	73.15	74.21	73.74	73.22	73.22	73.18	73.13	72.51	72.15	73.07	73.78	]
C9-CH2	14	45.10	46.64	1.54	[	48.01	48.17	45.53	45.59	45.10	42.08	45.14	45.14	45.60	45.64	48.33	49.14	45.35	42.95	]
C10-CH	11	78.82	78.68	-0.14	[	78.89	78.86	78.30	78.26	79.81	79.45	77.66	77.66	77.99	78.04	78.84	78.69	77.90	78.88	]
C11-CH	12	149.11	150.63	1.52	[	153.00	152.53	148.19	148.14	150.47	147.70	146.14	146.17	150.11	150.25	150.49	153.51	150.61	149.73	]

C12-C	8	133.19	131.82	-1.36	[	130.15	130.25	133.94	133.88	131.95	133.67	133.56	133.56	132.90	132.92	131.36	130.25	132.93	132.18]
C13-CH2	7	24.37	24.44	0.06	[	22.26	21.75	27.12	27.04	27.11	26.94	27.56	27.57	25.71	25.66	25.39	22.43	22.95	25.73]
C14-CH2	3	23.41	25.02	1.60	[	24.77	24.87	25.62	25.53	25.47	25.59	23.40	23.39	24.17	24.41	24.80	25.69	25.07	24.18]
C15-C	4	156.79	162.58	5.79	[	165.93	166.63	158.74	158.59	158.40	158.67	158.87	158.86	158.55	157.98	164.37	166.43	162.19	158.70]
C16-CH3**	6	23.35	22.31	-1.04	[	22.73	22.97	21.71	21.65	21.77	21.65	21.85	21.85	21.79	22.01	22.46	22.86	22.82	22.06]
C17-CH3**	5	19.36	17.30	-2.05	[	17.54	17.68	17.01	17.04	16.97	17.07	16.69	16.69	16.90	16.93	17.46	17.60	17.23	16.95]
C18-CH3	25	10.52	10.83	0.31	[	10.85	10.84	10.82	10.82	10.86	10.77	10.81	10.81	10.84	10.81	10.85	10.78	10.82	10.78]
C19-CH3	16	28.92	30.12	1.20	[	30.20	30.18	30.71	30.70	30.57	24.90	30.76	30.76	30.71	30.63	30.25	31.46	30.76	24.66]
C20-C	9	173.15	174.57	1.41	[	174.63	174.86	174.71	174.71	174.64	173.14	173.64	173.65	174.53	174.62	174.18	174.80	175.03	172.80]

13C chem shifts: RMSD=1.66ppm (MAE=1.14) N=20 {-2.05 5.79}

Fractions:	0.243	0.242	0.145	0.131	0.052	0.048	0.025	0.025	0.025	0.016	0.015	0.014	0.010	0.009

Conformer 1

Energy: -1227.89027 Hartree (Rel: 0.0 kcal/mol)

XYZ coordinates for conf 1:

C	3.18574	1.70247	0.41365
C	4.15305	0.70120	-0.04849
C	4.04719	0.30885	-1.51398
C	5.06199	0.15971	0.81477
C	5.19479	0.48851	2.28247
C	6.05016	-0.89091	0.37541
C	3.36912	-1.06331	-1.76884
C	2.00510	-1.17075	-1.14948
C	1.72730	-2.08037	-0.01141
O	0.42481	-1.91374	0.37545
C	-0.18813	-0.86919	-0.41965
C	0.87823	-0.49304	-1.40599
O	2.46606	-2.87262	0.53706
C	-0.62027	0.25000	0.54468
C	-1.30684	1.48369	-0.09746
C	-1.64868	2.50366	1.01037
O	-0.45304	2.10534	-1.06499
C	-2.54993	1.10882	-0.87889
C	-3.65237	0.49124	-0.42631
C	-4.86703	0.13934	-1.14053
C	-5.71742	-0.47198	-0.28812
C	-5.04432	-0.52483	1.02763
O	-3.79254	0.07202	0.89087
O	-5.41670	-0.97559	2.08565
C	-7.08668	-1.02880	-0.49650
O	2.31451	2.20350	-0.29812
H	3.25466	2.02328	1.46538
H	5.03843	0.29559	-1.98124
H	3.46778	1.08363	-2.02338
H	5.18332	-0.44247	2.86348
H	4.42641	1.14469	2.68881
H	6.17233	0.95413	2.46539
H	7.01814	-0.73713	0.86634
H	6.20747	-0.91956	-0.70414
H	5.69058	-1.88106	0.68872
H	3.30409	-1.21652	-2.85318
H	3.98951	-1.87167	-1.36888
H	-1.06682	-1.30669	-0.90880
H	0.71789	0.26811	-2.15699
H	-1.29607	-0.18860	1.28471
H	0.27169	0.59568	1.08070
H	-2.34529	2.08402	1.74153
H	-2.09867	3.39441	0.56198
H	-0.73217	2.80448	1.53201
H	0.41618	2.27819	-0.65269
H	-2.54908	1.40008	-1.92509
H	-5.02339	0.35374	-2.19091
H	-7.41451	-0.89772	-1.53101
H	-7.81092	-0.53764	0.16442
H	-7.11250	-2.09812	-0.25463

Conformer 2

Energy: -1227.88933 Hartree (Rel: 0.0 kcal/mol)

XYZ coordinates for conf 2:

C	3.17143	1.70601	0.47643
C	4.12439	0.70568	-0.01492
C	4.04883	0.39816	-1.50239
C	4.98559	0.08213	0.84188
C	5.08461	0.32090	2.32899
C	5.93513	-0.98936	0.36917
C	3.35833	-0.94695	-1.85507

C	2.00918	-1.09564	-1.21396
C	1.77743	-2.02132	-0.07873
O	0.47978	-1.89000	0.33634
C	-0.17303	-0.84993	-0.43319
C	0.86230	-0.44107	-1.43946
O	2.54644	-2.80073	0.44692
C	-0.60461	0.24934	0.55444
C	-1.29931	1.49303	-0.05892
C	-1.64854	2.48416	1.07260
O	-0.44840	2.14312	-1.01018
C	-2.53932	1.12918	-0.85032
C	-3.63629	0.49023	-0.41447
C	-4.84801	0.14694	-1.13778
C	-5.69262	-0.49474	-0.30205
C	-5.01861	-0.57693	1.01176
O	-3.77224	0.03459	0.89100
O	-5.38676	-1.05884	2.05743
C	-7.05682	-1.05811	-0.52537
O	2.32075	2.25594	-0.22454
H	3.22853	1.97605	1.54315
H	5.05112	0.39826	-1.94640
H	3.49055	1.20890	-1.97813
H	4.99237	-0.63829	2.85459
H	4.34544	1.00308	2.74660
H	6.08265	0.70994	2.57025
H	6.87098	-0.95797	0.93803
H	6.17060	-0.92212	-0.69500
H	5.48088	-1.97364	0.54997
H	3.26797	-1.00928	-2.94624
H	3.98267	-1.78719	-1.53609
H	-1.05356	-1.30060	-0.90645
H	0.66965	0.32306	-2.17986
H	-1.27291	-0.20583	1.29124
H	0.28971	0.58870	1.09030
H	-2.34408	2.04317	1.79220
H	-2.10267	3.38295	0.64493
H	-0.73449	2.77736	1.60283
H	0.41974	2.31098	-0.59337
H	-2.54128	1.44876	-1.88822
H	-5.00656	0.38806	-2.18198
H	-7.38558	-0.90300	-1.55624
H	-7.78554	-0.59079	0.14781
H	-7.07313	-2.13355	-0.31143

Conformer 3
Energy: -1227.89037 Hartree (Rel: 0.3 kcal/mol)
XYZ coordinates for conf 3:

C	-6.48729	-1.07770	0.14481
C	-5.62750	0.11060	0.27121
C	-4.14770	-0.19080	0.40601
C	-6.13650	1.37340	0.22341
C	-7.60250	1.72240	0.11121
C	-5.26020	2.60150	0.28871
C	-3.43740	-0.32130	-0.96679
C	-1.95280	-0.45240	-0.82419
C	-1.11660	0.69340	-0.38319
O	0.18860	0.29110	-0.32999
C	0.28680	-1.10410	-0.71209
C	-1.13079	-1.49260	-1.01569
O	-1.45380	1.82620	-0.10249
C	0.93551	-1.92870	0.40551
C	2.46731	-1.77070	0.51851
C	2.99371	-2.68970	1.64351
O	2.97461	-2.21000	-0.74449

C	2.85490	-0.33690	0.83831
C	3.99270	0.27740	0.48121
C	4.46070	1.62090	0.76241
C	5.67490	1.79601	0.19631
C	6.02740	0.53100	-0.48009
O	4.97240	-0.36270	-0.27799
O	7.00760	0.21431	-1.11079
C	6.58610	2.97851	0.17781
O	-6.06179	-2.22800	0.13491
H	-7.57409	-0.90770	0.04501
H	-4.03569	-1.13950	0.93941
H	-3.64640	0.57750	0.99881
H	-7.86620	2.43950	0.89951
H	-8.28790	0.87879	0.18501
H	-7.79110	2.23200	-0.84309
H	-5.57580	3.32540	-0.47329
H	-4.19650	2.40230	0.15121
H	-5.39310	3.10190	1.25881
H	-3.84230	-1.18430	-1.50439
H	-3.65480	0.57180	-1.56609
H	0.92310	-1.15170	-1.60339
H	-1.40249	-2.49090	-1.34199
H	0.45260	-1.68680	1.35871
H	0.74161	-2.98740	0.19751
H	2.59351	-2.40050	2.62171
H	4.08631	-2.63400	1.69091
H	2.70541	-3.72610	1.43701
H	3.90600	-1.93870	-0.81049
H	2.16670	0.24090	1.44661
H	3.89100	2.34060	1.33741
H	6.15850	3.81501	0.73621
H	6.77640	3.30671	-0.85109
H	7.55950	2.72661	0.61541

Conformer 4

Energy: -1227.88869 Hartree (Rel: 0.4 kcal/mol)

XYZ coordinates for conf 4:

C	6.46491	-0.73540	1.09140
C	5.60812	0.32794	0.54139
C	4.12240	0.11857	0.75955
C	6.12602	1.38524	-0.14391
C	7.59759	1.64839	-0.36508
C	5.25445	2.44891	-0.76827
C	3.46990	-0.74072	-0.35526
C	1.97915	-0.79229	-0.22642
C	1.13967	0.39682	-0.52296
O	-0.17104	0.07551	-0.30636
C	-0.27172	-1.30408	0.12708
C	1.15298	-1.77620	0.15298
O	1.47860	1.50016	-0.90162
C	-1.17946	-2.10437	-0.81347
C	-2.69023	-1.83914	-0.63304
C	-3.48665	-2.74771	-1.59641
O	-2.96728	-2.20624	0.72083
C	-3.03712	-0.39086	-0.93188
C	-4.02673	0.32392	-0.37575
C	-4.44456	1.69132	-0.61822
C	-5.49504	1.98308	0.17975
C	-5.78811	0.77312	0.97481
O	-4.86755	-0.20951	0.60110
O	-6.63349	0.55726	1.81004
C	-6.29220	3.23632	0.33140
O	6.03220	-1.72648	1.67014
H	7.55613	-0.62528	0.96046

H	3.60073	1.07552	0.82880
H	3.98283	-0.39869	1.71348
H	7.83083	2.67863	-0.06616
H	7.83114	1.57901	-1.43600
H	8.27390	0.98441	0.17225
H	5.33288	3.38095	-0.19004
H	4.19949	2.18047	-0.83843
H	5.61746	2.68414	-1.77678
H	3.72302	-0.30851	-1.33151
H	3.88743	-1.75182	-0.32500
H	-0.71776	-1.29756	1.12851
H	1.42496	-2.78614	0.44079
H	-0.87811	-1.91982	-1.85053
H	-1.01850	-3.16915	-0.60785
H	-3.27039	-2.51275	-2.64460
H	-4.56116	-2.61621	-1.43133
H	-3.23025	-3.79620	-1.40954
H	-3.84272	-1.85715	0.96004
H	-2.45515	0.11023	-1.69844
H	-3.95804	2.34335	-1.33339
H	-7.35097	3.05317	0.11210
H	-5.92779	4.01970	-0.33815
H	-6.24170	3.60670	1.36224

Conformer 5

Energy: -1227.88827 Hartree (Rel: 0.9 kcal/mol)

XYZ coordinates for conf 5:

C	-5.08275	2.61144	-0.55541
C	-5.12673	1.28459	0.08102
C	-3.77675	0.74391	0.51007
C	-6.29337	0.59564	0.22281
C	-7.66236	1.10246	-0.16841
C	-6.34646	-0.79239	0.81522
C	-3.05539	-0.03139	-0.62327
C	-1.80625	-0.70283	-0.14181
C	-1.86421	-1.87211	0.77078
O	-0.58810	-2.27587	1.04985
C	0.35575	-1.41834	0.36253
C	-0.51327	-0.44039	-0.37554
O	-2.83338	-2.43749	1.23694
C	1.27549	-2.29919	-0.49807
C	2.56958	-1.60486	-0.96898
C	3.37561	-2.57094	-1.86581
O	2.15337	-0.46988	-1.73196
C	3.42477	-1.22529	0.22931
C	4.23503	-0.16139	0.34129
C	5.10622	0.23719	1.43205
C	5.74320	1.37946	1.09550
C	5.28625	1.74990	-0.26076
O	4.36756	0.78122	-0.67505
O	5.58175	2.68022	-0.97069
C	6.73364	2.20779	1.84437
O	-4.04811	3.24295	-0.74246
H	-6.04274	3.04642	-0.88600
H	-3.87695	0.09469	1.38258
H	-3.14400	1.58763	0.80162
H	-8.34829	0.98689	0.68094
H	-8.07038	0.48387	-0.97896
H	-7.69891	2.14377	-0.48673
H	-6.80907	-0.75429	1.81208
H	-5.37437	-1.27809	0.90939
H	-6.99355	-1.43396	0.20355
H	-3.73554	-0.79589	-1.01984
H	-2.82206	0.65642	-1.44196

H	0.94763	-0.91119	1.13651
H	-0.09146	0.33416	-1.00238
H	1.54455	-3.17969	0.09560
H	0.72248	-2.65072	-1.37575
H	3.68846	-3.46669	-1.31830
H	4.27359	-2.06950	-2.24213
H	2.76131	-2.87583	-2.71962
H	2.92666	0.10022	-1.88142
H	3.43134	-1.91905	1.06639
H	5.19890	-0.32335	2.35445
H	7.67182	2.29313	1.28319
H	6.95087	1.77480	2.82406
H	6.35684	3.22743	1.98843

Conformer 6
Energy: -1227.88781 Hartree (Rel: 1.0 kcal/mol)
XYZ coordinates for conf 6:

C	-5.93507	2.34726	0.17697
C	-5.66199	0.90435	0.07387
C	-4.30683	0.48027	0.60518
C	-6.55024	0.04389	-0.49721
C	-7.92004	0.41740	-1.01465
C	-6.25465	-1.42680	-0.67110
C	-3.18350	0.61026	-0.45675
C	-1.88445	0.04152	0.02393
C	-1.70043	-1.42323	0.17705
O	-0.43134	-1.65453	0.65330
C	0.27021	-0.39220	0.81652
C	-0.74230	0.63386	0.39835
O	-2.47201	-2.32858	-0.05326
C	1.55875	-0.37995	-0.01620
C	2.53367	-1.56477	0.24745
C	2.86491	-1.74194	1.74413
O	1.97852	-2.77977	-0.27086
C	3.80145	-1.36135	-0.55715
C	4.68594	-0.35705	-0.46064
C	5.91282	-0.13691	-1.20517
C	6.48847	1.00767	-0.77799
C	5.61457	1.56118	0.27810
O	4.53386	0.69527	0.43522
O	5.71967	2.56557	0.94297
C	7.74759	1.68996	-1.19922
O	-5.13775	3.15863	0.63574
H	-6.91211	2.70586	-0.19271
H	-4.33774	-0.54652	0.97598
H	-4.05074	1.12489	1.45143
H	-7.95831	0.27457	-2.10288
H	-8.23700	1.43657	-0.79590
H	-8.66701	-0.26520	-0.58892
H	-6.85266	-2.01370	0.04096
H	-5.20569	-1.69289	-0.53394
H	-6.56396	-1.75381	-1.67182
H	-3.48680	0.07408	-1.36462
H	-3.05757	1.66403	-0.72283
H	0.50505	-0.29911	1.88339
H	-0.52912	1.69723	0.41017
H	1.29651	-0.39173	-1.07990
H	2.06963	0.56576	0.19147
H	3.29359	-0.83035	2.17146
H	3.58549	-2.55658	1.86107
H	1.96502	-2.00862	2.30921
H	1.06961	-2.84286	0.07142
H	4.01284	-2.13353	-1.29076
H	6.27248	-0.81134	-1.97278

H	8.25532	1.13061	-1.98929
H	8.43421	1.79763	-0.35093
H	7.53804	2.70170	-1.56679

Conformer 7
Energy: -1227.88773 Hartree (Rel: 1.4 kcal/mol)
XYZ coordinates for conf 7:

C	3.59421	0.52749	1.55100
C	4.60400	-0.09130	0.67470
C	4.31411	-1.52530	0.26760
C	5.70360	0.60380	0.26620
C	6.04220	2.02550	0.65320
C	6.74540	0.01070	-0.65050
C	3.58891	-1.66951	-1.09980
C	2.19171	-1.13311	-1.07520
C	1.10071	-1.86121	-0.37720
O	-0.04729	-1.12371	-0.47250
C	0.21701	0.10479	-1.19230
C	1.66660	-0.00541	-1.56960
O	1.13341	-2.93611	0.18290
C	-0.08740	1.32229	-0.31030
C	-1.58890	1.61879	-0.10920
C	-1.74600	2.89519	0.74790
O	-2.10020	1.85279	-1.42470
C	-2.28899	0.47189	0.59860
C	-3.57130	0.10679	0.45160
C	-4.32499	-0.94721	1.10220
C	-5.59309	-0.93991	0.63570
C	-5.68799	0.15319	-0.35260
O	-4.43269	0.76119	-0.43030
O	-6.61690	0.54069	-1.02110
C	-6.76289	-1.80741	0.96460
O	2.56990	-0.03501	1.92450
H	3.78360	1.56259	1.88380
H	3.68401	-1.98181	1.03540
H	5.23901	-2.10790	0.22680
H	7.03230	2.04870	1.12700
H	5.33750	2.50660	1.32980
H	6.11990	2.64510	-0.25000
H	6.84950	0.63280	-1.54950
H	6.53191	-1.00990	-0.96790
H	7.72691	0.01980	-0.15720
H	4.16581	-1.16881	-1.88500
H	3.55111	-2.73761	-1.34790
H	-0.44420	0.11709	-2.06600
H	2.17841	0.76239	-2.14000
H	0.41520	1.19379	0.65450
H	0.34600	2.20489	-0.79590
H	-1.33230	2.75939	1.75320
H	-2.80600	3.15219	0.84550
H	-1.22760	3.72949	0.26270
H	-3.07120	1.82519	-1.38440
H	-1.70989	-0.08771	1.32580
H	-3.89619	-1.61621	1.83830
H	-7.60179	-1.20601	1.33480
H	-6.50309	-2.54891	1.72450
H	-7.12019	-2.33411	0.07150

Conformer 8
Energy: -1227.88816 Hartree (Rel: 1.4 kcal/mol)
XYZ coordinates for conf 8:

C	3.59420	0.52719	1.55079
C	4.60400	-0.09172	0.67449
C	4.31380	-1.52551	0.26699

C	5.70390	0.60308	0.26649
C	6.04310	2.02448	0.65409
C	6.74570	0.00968	-0.65001
C	3.58850	-1.66921	-1.10041
C	2.19130	-1.13261	-1.07551
C	1.10040	-1.86061	-0.37741
O	-0.04760	-1.12301	-0.47261
C	0.21660	0.10539	-1.19241
C	1.66620	-0.00491	-1.57001
O	1.13289	-2.93551	0.18279
C	-0.08760	1.32299	-0.31051
C	-1.58910	1.61949	-0.10921
C	-1.74600	2.89599	0.74769
O	-2.10050	1.85339	-1.42461
C	-2.28910	0.47269	0.59889
C	-3.57120	0.10709	0.45179
C	-4.32450	-0.94681	1.10289
C	-5.59260	-0.94031	0.63609
C	-5.68770	0.15229	-0.35271
O	-4.43270	0.76069	-0.43061
O	-6.61670	0.53899	-1.02171
C	-6.76220	-1.80791	0.96549
O	2.56980	-0.03511	1.92419
H	3.78380	1.56229	1.88359
H	3.68370	-1.98211	1.03469
H	5.23850	-2.10821	0.22599
H	7.03460	2.04738	1.12509
H	5.34020	2.50448	1.33329
H	6.11810	2.64508	-0.24861
H	6.85420	0.63478	-1.54651
H	6.52960	-1.00901	-0.97161
H	7.72610	0.01368	-0.15451
H	4.16530	-1.16822	-1.88551
H	3.55050	-2.73711	-1.34891
H	-0.44470	0.11769	-2.06611
H	2.17800	0.76279	-2.14051
H	0.41510	1.19459	0.65419
H	0.34570	2.20549	-0.79631
H	-1.33220	2.76029	1.75309
H	-2.80600	3.15299	0.84549
H	-1.22770	3.73029	0.26249
H	-3.07160	1.82529	-1.38431
H	-1.70990	-0.08631	1.32649
H	-3.89560	-1.61531	1.83929
H	-6.50160	-2.55031	1.72419
H	-7.12071	-2.33341	0.07219
H	-7.60050	-1.20691	1.33749

Conformer 9

Energy: -1227.88823 Hartree (Rel: 1.4 kcal/mol)

XYZ coordinates for conf 9:

C	5.83840	-0.07860	-0.97940
C	4.81090	-0.81410	-0.22020
C	4.60260	-0.29660	1.19050
C	4.14100	-1.86540	-0.77170
C	4.36050	-2.40460	-2.16800
C	3.08250	-2.65120	-0.03630
C	3.81730	1.04110	1.26820
C	2.39230	0.95130	0.81450
C	1.36700	0.19000	1.57350
O	0.16610	0.30390	0.92840
C	0.32180	1.12410	-0.25570
C	1.77820	1.48610	-0.24970
O	1.48990	-0.45540	2.59470

C	-0.62210	2.33070	-0.21060
C	-2.10300	2.00320	-0.50260
C	-2.92670	3.31050	-0.48710
O	-2.10320	1.44520	-1.81920
C	-2.67520	1.05640	0.53790
C	-3.63600	0.14080	0.34370
C	-4.25940	-0.77380	1.28070
C	-5.18540	-1.51260	0.63120
C	-5.18060	-1.07590	-0.77970
O	-4.22140	-0.06740	-0.90530
O	-5.83440	-1.44300	-1.72680
C	-6.09800	-2.58900	1.11860
O	6.47330	0.86680	-0.52360
H	6.03860	-0.40590	-2.01470
H	5.58910	-0.10580	1.62650
H	4.11170	-1.03640	1.82410
H	4.56060	-3.48270	-2.11500
H	5.17310	-1.94220	-2.72640
H	3.43930	-2.29220	-2.75500
H	2.18500	-2.74310	-0.66180
H	2.78510	-2.22630	0.92160
H	3.43780	-3.67650	0.13870
H	3.83010	1.37100	2.31520
H	4.34340	1.79930	0.68200
H	0.05760	0.50130	-1.11830
H	2.22530	2.10900	-1.01720
H	-0.52380	2.83080	0.75920
H	-0.29640	3.03920	-0.98120
H	-2.91710	3.78240	0.50170
H	-3.96730	3.09990	-0.75530
H	-2.51320	4.01420	-1.21770
H	-2.96300	1.01700	-1.97030
H	-2.30300	1.15300	1.55270
H	-3.99050	-0.82530	2.32880
H	-5.95940	-2.76980	2.18760
H	-5.91620	-3.52650	0.57960
H	-7.14630	-2.32010	0.94210

Conformer 10

Energy: -1227.89085 Hartree (Rel: 1.6 kcal/mol)

XYZ coordinates for conf 10:

C	5.84541	0.63397	-0.42291
C	4.85344	-0.07093	0.40964
C	4.54295	-1.47893	-0.06240
C	4.30196	0.52245	1.50569
C	4.63175	1.91186	2.00547
C	3.28370	-0.15648	2.38979
C	3.65052	-1.54582	-1.33151
C	2.25537	-1.03221	-1.14490
C	1.26214	-1.72870	-0.28731
O	0.07752	-1.04682	-0.34143
C	0.21812	0.11236	-1.19953
C	1.64145	0.03767	-1.66872
O	1.39681	-2.73090	0.38510
C	-0.12904	1.39549	-0.43574
C	-1.64167	1.64126	-0.24466
C	-1.85284	2.97502	0.50595
O	-2.16388	1.74359	-1.57204
C	-2.28758	0.52220	0.55434
C	-3.55402	0.09393	0.44444
C	-4.25875	-0.94317	1.17338
C	-5.52758	-1.02345	0.71645
C	-5.67390	-0.00699	-0.34552
O	-4.44568	0.64739	-0.47423

O	-6.62061	0.29235	-1.03306
C	-6.65753	-1.91515	1.11257
O	6.38294	0.13731	-1.40696
H	6.10928	1.66684	-0.13536
H	4.08756	-2.07826	0.72700
H	5.49160	-1.96094	-0.32241
H	3.73868	2.54811	1.94477
H	5.44125	2.41816	1.48173
H	4.90060	1.86160	3.06856
H	3.72962	-0.36966	3.37165
H	2.88424	-1.08727	1.98889
H	2.44270	0.52339	2.57955
H	4.13817	-0.99666	-2.14135
H	3.59468	-2.59815	-1.63931
H	-0.48900	-0.00809	-2.02864
H	2.06769	0.76844	-2.34781
H	0.38474	1.38780	0.53207
H	0.25854	2.24563	-1.00910
H	-1.43374	2.94013	1.51775
H	-2.92293	3.19262	0.58678
H	-1.37202	3.78899	-0.04748
H	-3.13310	1.67997	-1.52532
H	-1.68142	0.04026	1.31442
H	-3.79848	-1.54010	1.95126
H	-6.36029	-2.59768	1.91277
H	-7.00213	-2.50887	0.25746
H	-7.51636	-1.32605	1.45593

Conformer 11

Energy: -1227.89085 Hartree (Rel: 1.6 kcal/mol)

XYZ coordinates for conf 11:

C	-3.32504	-1.41965	0.85719
C	-4.31462	-0.58300	0.17299
C	-4.20475	0.91726	0.39665
C	-5.25965	-1.14990	-0.63437
C	-5.44545	-2.63001	-0.87590
C	-6.26910	-0.32696	-1.39552
C	-3.40231	1.68749	-0.69663
C	-1.91543	1.65532	-0.49071
C	-1.20583	2.81838	0.10134
O	0.11861	2.50474	0.23358
C	0.33653	1.13496	-0.18183
C	-1.00381	0.69564	-0.69745
O	-1.64709	3.90267	0.42116
C	0.85928	0.36820	1.04648
C	1.18947	-1.13184	0.83823
C	1.72141	-1.71894	2.16375
O	0.02400	-1.86035	0.43336
C	2.18440	-1.35697	-0.28223
C	3.43504	-0.87959	-0.38116
C	4.42033	-1.09263	-1.42678
C	5.54765	-0.41867	-1.11324
C	5.29910	0.25900	0.17774
O	4.00143	-0.05137	0.57961
O	6.01428	0.96877	0.84541
C	6.85106	-0.29702	-1.83062
O	-2.43351	-0.98296	1.58789
H	-3.37591	-2.50578	0.68216
H	-3.73030	1.08796	1.36726
H	-5.20553	1.35399	0.45775
H	-4.82960	-3.28703	-0.26369
H	-5.24465	-2.86005	-1.93070
H	-6.49545	-2.89559	-0.69843
H	-6.30047	-0.64878	-2.44427

H	-6.07995	0.74592	-1.37326
H	-7.27469	-0.50644	-0.98985
H	-3.65891	1.29299	-1.68630
H	-3.71428	2.73730	-0.67483
H	1.09777	1.15267	-0.97089
H	-1.15735	-0.28962	-1.11585
H	1.75662	0.88440	1.40051
H	0.10125	0.44457	1.83550
H	2.65030	-1.23224	2.47388
H	1.90572	-2.79052	2.04186
H	0.97474	-1.58321	2.95532
H	-0.70129	-1.65745	1.05918
H	1.84340	-2.00122	-1.08731
H	4.23739	-1.70434	-2.30203
H	6.84256	-0.86257	-2.76603
H	7.67426	-0.66584	-1.20695
H	7.07292	0.75244	-2.05857

Conformer 12
Energy: -1227.88939 Hartree (Rel: 1.7 kcal/mol)
XYZ coordinates for conf 12:

C	2.97010	1.09991	-1.36123
C	3.63571	-0.18056	-1.09913
C	2.83004	-1.42814	-1.42584
C	4.89471	-0.21427	-0.57147
C	5.73205	0.98473	-0.19667
C	5.59049	-1.51477	-0.25874
C	2.23168	-2.15931	-0.19364
C	1.38286	-1.26028	0.65673
C	1.85851	-0.71349	1.94997
O	0.89582	0.12218	2.45290
C	-0.19776	0.22268	1.50786
C	0.16733	-0.74732	0.42277
O	2.89721	-0.92571	2.54221
C	-0.28701	1.69596	1.06777
C	-1.31558	2.02375	-0.05109
C	-1.26263	3.54172	-0.33640
O	-1.02229	1.31338	-1.24732
C	-2.71069	1.66580	0.40739
C	-3.49255	0.61806	0.09557
C	-4.84286	0.35509	0.57188
C	-5.28292	-0.80195	0.03493
C	-4.18739	-1.32015	-0.81278
O	-3.12923	-0.42127	-0.74691
O	-4.11872	-2.32897	-1.47706
C	-6.58029	-1.52536	0.18182
O	1.81646	1.21556	-1.77575
H	3.55389	2.01608	-1.17701
H	3.44299	-2.14329	-1.98652
H	2.00820	-1.13093	-2.08298
H	5.24169	1.95181	-0.29820
H	6.64450	0.99851	-0.80734
H	6.06014	0.88240	0.84579
H	6.66514	-1.43551	-0.45856
H	5.19405	-2.36650	-0.81458
H	5.48039	-1.73282	0.81270
H	1.63629	-3.00582	-0.55573
H	3.03391	-2.56995	0.42757
H	-1.11200	-0.07313	2.03541
H	-0.48077	-0.92704	-0.42408
H	-0.50667	2.30056	1.95515
H	0.70456	1.99871	0.71058
H	-1.51107	4.13618	0.54980
H	-1.96687	3.78987	-1.13623

H	-0.25465	3.81835	-0.66556
H	-0.07301	1.42481	-1.45033
H	-3.15278	2.36762	1.11064
H	-5.37394	1.01504	1.24744
H	-7.25650	-0.99418	0.85678
H	-6.41922	-2.53711	0.57295
H	-7.07505	-1.63776	-0.79044

Conformer 13
Energy: -1227.88869 Hartree (Rel: 1.9 kcal/mol)
XYZ coordinates for conf 13:

C	-3.70770	-0.53190	1.73130
C	-4.41240	0.15021	0.63360
C	-4.75030	-0.72559	-0.56160
C	-4.70740	1.47890	0.70290
C	-4.35420	2.40481	1.84220
C	-5.40260	2.20091	-0.42380
C	-3.85600	-0.50650	-1.81250
C	-2.38790	-0.62200	-1.52640
C	-1.54540	0.56880	-1.24620
O	-0.27180	0.15000	-0.97190
C	-0.20200	-1.29460	-1.05540
C	-1.59610	-1.69850	-1.43290
O	-1.84720	1.74460	-1.25070
C	0.26920	-1.90100	0.27130
C	1.77110	-1.71280	0.57450
C	2.11760	-2.43250	1.89750
O	2.44950	-2.34570	-0.51410
C	2.12990	-0.24330	0.71370
C	3.31300	0.31920	0.42540
C	3.75260	1.69490	0.55480
C	5.03510	1.79260	0.14110
C	5.46350	0.44130	-0.27380
O	4.38130	-0.42110	-0.08210
O	6.51810	0.04050	-0.70580
C	5.95300	2.96750	0.06360
O	-3.37380	-1.71270	1.71340
H	-3.47710	0.07190	2.62630
H	-5.79460	-0.58189	-0.86330
H	-4.64880	-1.76700	-0.24440
H	-3.78090	3.25430	1.44830
H	-3.77430	1.95630	2.64770
H	-5.27180	2.82461	2.27510
H	-6.08550	2.96381	-0.03260
H	-5.96470	1.53761	-1.08440
H	-4.65010	2.72221	-1.03100
H	-4.14400	-1.24400	-2.57070
H	-4.04580	0.48510	-2.23590
H	0.52720	-1.53710	-1.83690
H	-1.87970	-2.73560	-1.57680
H	-0.34520	-1.50660	1.08780
H	0.08900	-2.98140	0.22300
H	1.58440	-1.99300	2.74780
H	3.19270	-2.35940	2.09250
H	1.84700	-3.49130	1.82160
H	3.38410	-2.07870	-0.48840
H	1.37200	0.41580	1.12440
H	3.11680	2.48940	0.92600
H	5.46130	3.87690	0.41860
H	6.28820	3.13200	-0.96750
H	6.85340	2.79860	0.66640

Conformer 14
Energy: -1227.88869 Hartree (Rel: 2.0 kcal/mol)
XYZ coordinates for conf 14:

C	3.91770	2.79470	-0.60950
C	3.77390	1.60340	0.24780
C	4.62840	0.42920	-0.19280
C	2.94170	1.60560	1.32860
C	2.09130	2.77740	1.76930
C	2.76310	0.42110	2.24790
C	4.07490	-0.32820	-1.43310
C	2.73780	-0.95870	-1.19600
C	2.55930	-2.07230	-0.23260
O	1.22060	-2.38790	-0.17600
C	0.47300	-1.52900	-1.07880
C	1.53210	-0.66940	-1.70510
O	3.38050	-2.66010	0.43570
C	-0.58780	-0.71230	-0.32140
C	-1.45120	-1.50940	0.69760
C	-2.08120	-2.77580	0.08220
O	-0.64800	-1.86470	1.83190
C	-2.52210	-0.60480	1.27180
C	-3.51710	0.01130	0.61550
C	-4.56820	0.86950	1.13270
C	-5.35420	1.26890	0.10970
C	-4.80430	0.65880	-1.11970
O	-3.68960	-0.09650	-0.75950
O	-5.17000	0.73369	-2.26950
C	-6.56230	2.14540	0.08430
O	4.65260	2.84030	-1.59030
H	3.33150	3.69060	-0.34050
H	4.78140	-0.28460	0.61750
H	5.61430	0.81350	-0.47420
H	2.11760	3.64820	1.11600
H	2.40150	3.09850	2.77250
H	1.04540	2.45660	1.86040
H	2.93750	0.72960	3.28730
H	3.41380	-0.42400	2.02770
H	1.72450	0.06270	2.20770
H	4.01330	0.36160	-2.27900
H	4.79880	-1.10970	-1.69680
H	-0.00540	-2.18990	-1.81060
H	1.29940	0.09220	-2.44140
H	-0.09450	0.09530	0.22980
H	-1.23480	-0.25140	-1.07470
H	-2.71010	-2.52910	-0.77850
H	-2.69700	-3.27800	0.83400
H	-1.30280	-3.47630	-0.23880
H	0.11320	-2.36190	1.48390
H	-2.47560	-0.45580	2.34650
H	-4.66970	1.12110	2.18150
H	-6.81580	2.49839	1.08720
H	-7.42520	1.60620	-0.32470
H	-6.39820	3.01609	-0.56180

LIST OF FILES (technical info - delete in the final SI version
abimael-PkC-SR-Z-du8ml-chloroform_46.log abimael-PkC-SR-Z-du8ml-chloroform_65.log abimael-PkC-SR-Z-du8ml-chloroform_155.log abimael-PkC-SR-Z-du8ml-chloroform_12.log abimael-PkC-SR-Z-du8ml-chloroform_7.log abimael-PkC-SR-Z-du8ml-chloroform_143.log abimael-PkC-SR-Z-du8ml-chloroform_17.log abimael-PkC-SR-Z-du8ml-chloroform_87.log abimael-PkC-SR-Z-du8ml-chloroform_83.log abimael-PkC-SR-Z-du8ml-chloroform_185.log abimael-PkC-SR-Z-du8ml-chloroform_244.log abimael-PkC-SR-Z-du8ml-chloroform_233.log abimael-PkC-SR-Z-du8ml-chloroform_199.log abimael-PkC-SR-Z-du8ml-chloroform_222.log

DU8ML data for 2-(2-((*S*)-5-((*R*,*E*)-2-hydroxy-2-methyl-3-(4-methyl-5-oxofuran-2(5*H*)-ylidene)propyl)-2-oxo-2,5-dihydrofuran-3-yl)ethyl)-3-methylbut-2-enal (**10*S*,8*R*,6*E* diastereomer**)

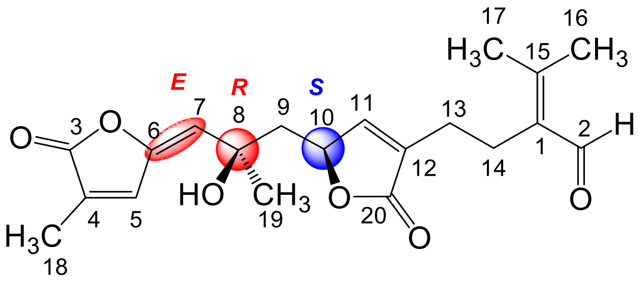

NMR parameters calculated for 10*S*,8*R*,6*E* diastereomer vs experimental data of kallopterolide C (**3**):

	Conf1	Conf2	Conf3	Conf4	Conf5	Conf6	Conf7	Conf8	Conf9	Conf10	Conf11	Conf12	Conf13	Conf14	Conf15	Conf16	Conf17	Conf18
Rel energy (kcal/mol):	0.00	0.17	0.29	1.08	1.10	1.12	1.18	1.24	1.39	1.45	1.53	1.69	1.74	1.77	1.82	1.90	1.95	1.98

C-nom	iGau	Exp	Calc	diff		1	2	3	4	5	6	7	8	9	10	11	12	13
C1-C	2	135.35	136.12	0.77	[	136.52	134.28	138.47	136.10	134.75	134.64	136.72	134.72	136.84	136.53	136.00	133.98	136.45
C2-CH	1	190.66	192.69	2.03	[	189.47	200.85	193.97	190.96	189.92	190.03	189.50	189.93	189.52	189.18	190.03	188.96	189.01
C3-C	22	170.02	169.37	-0.66	[	169.46	169.27	169.27	169.64	169.36	169.35	169.21	169.43	169.01	169.89	169.48	169.45	169.90
C4-C	21	131.27	131.13	-0.15	[	131.07	131.20	131.01	130.59	131.21	131.13	132.12	131.01	130.84	130.75	130.85	130.86	130.82
C5-CH	20	136.50	138.07	1.57	[	138.43	137.96	138.03	138.88	138.31	138.27	137.66	138.32	137.12	137.54	138.57	138.46	137.71
C6-C	19	149.51	148.29	-1.22	[	148.20	148.46	148.41	148.51	148.06	148.11	149.29	148.38	147.82	148.06	148.08	148.11	147.64
C7-CH	18	117.38	116.76	-0.62	[	117.21	115.96	116.18	117.68	117.05	117.18	114.10	117.03	118.02	118.04	117.51	117.31	118.91
C8-C	15	72.72	73.75	1.03	[	74.23	73.17	73.11	74.10	74.33	74.27	74.33	74.17	73.66	73.85	74.21	74.21	73.59
C9-CH2	14	46.30	45.80	-0.49	[	43.78	47.94	49.48	43.13	44.39	44.19	47.37	43.91	41.74	41.67	43.67	43.65	41.87
C10-CH	11	78.77	78.79	0.02	[	79.07	78.76	78.55	78.54	78.69	78.76	79.21	78.83	80.27	79.19	78.20	78.69	79.73
C11-CH	12	148.56	149.32	0.76	[	147.41	153.28	150.06	145.22	149.43	149.47	148.55	149.42	148.06	148.53	145.39	149.88	147.72
C12-C	8	133.63	132.49	-1.14	[	133.95	129.25	131.64	133.87	132.61	132.90	133.05	132.96	134.93	135.36	133.62	132.89	136.05
C13-CH2	7	24.39	26.30	1.91	[	27.04	26.38	25.33	27.67	25.76	25.66	27.07	25.62	26.67	24.31	27.54	22.70	25.98
C14-CH2	3	23.39	25.66	2.27	[	25.39	28.15	24.78	23.28	24.23	24.32	25.59	24.19	26.31	28.54	22.83	24.25	25.23
C15-C	4	157.06	159.86	2.81	[	158.83	158.20	165.26	159.09	158.58	158.48	158.86	158.43	157.79	159.89	158.30	162.11	159.32
C16-CH3**	6	23.31	22.01	-1.30	[	21.62	22.29	22.50	21.81	22.08	21.90	21.73	21.80	21.58	21.89	21.81	22.81	22.45
C17-CH3**	5	19.38	18.22	-1.16	[	17.06	22.22	17.60	16.75	16.88	16.89	16.99	16.99	17.11	18.22	16.66	17.18	18.63
C18-CH3	25	10.83	11.06	0.23	[	11.03	11.10	11.09	11.02	11.05	11.04	11.10	10.99	11.02	11.03	10.96	10.96	11.01
C19-CH3	16	30.80	28.58	-2.22	[	25.66	30.92	31.50	25.86	25.43	25.50	31.21	25.74	30.97	30.53	25.62	25.67	30.87
C20-C	9	172.67	173.22	0.56	[	172.87	173.21	173.97	171.96	172.57	172.72	174.17	172.78	173.78	173.95	171.72	173.41	172.31

14	15	16	17	18	
136.79	136.76	136.81	136.76	135.42	]
189.50	189.49	189.47	189.45	192.58	]
169.40	169.40	169.07	169.10	168.92	]
130.97	130.98	131.89	132.02	133.49	]
137.97	138.04	135.89	135.85	133.29	]
147.59	147.58	148.23	148.27	147.25	]
115.93	115.83	117.84	117.74	120.59	]
74.19	74.15	73.45	73.40	71.11	]
47.55	47.67	45.32	45.22	51.51	]
77.44	77.35	77.12	77.09	78.40	]
147.05	147.08	147.29	147.29	152.93	]
134.61	134.55	134.43	134.39	130.43	]
27.07	27.09	27.08	27.00	22.33	]
25.73	25.44	25.57	25.60	25.09	]
158.96	158.90	158.85	158.83	166.11	]
21.71	21.69	21.68	21.67	22.80	]
17.05	17.02	16.98	17.03	17.54	]
11.09	11.11	11.13	11.11	11.12	]
31.20	31.16	31.41	31.45	33.20	]
174.36	174.35	174.41	174.42	174.56	]

13C chem shifts: RMSD=1.37ppm (MAE=1.15) N=20 {-2.22 2.81}

Fractions: 0.273 0.204 0.166 0.044 0.043 0.041 0.037 0.034 0.026 0.024 0.021 0.016 0.014 0.014 0.013 0.011 0.010 0.010

NMR parameters calculated for 10*S*,8*R*,6*E* diastereomer vs experimental data of kallopterolide D (**4**):

C-nom	iGau	Exp	Calc	diff		1	2	3	4	5	6	7	8	9	10	11	12	13
C1-C	2	135.49	136.12	0.63	[	136.52	134.28	138.47	136.10	134.75	134.64	136.72	134.72	136.84	136.53	136.00	133.98	136.45
C2-CH	1	190.65	192.69	2.04	[	189.47	200.85	193.97	190.96	189.92	190.03	189.50	189.93	189.52	189.18	190.03	188.96	189.01
C3-C	22	169.95	169.37	-0.58	[	169.46	169.27	169.27	169.64	169.36	169.35	169.21	169.43	169.01	169.89	169.48	169.45	169.90
C4-C	21	129.26	131.13	1.86	[	131.07	131.20	131.01	130.59	131.21	131.13	132.12	131.01	130.84	130.75	130.85	130.86	130.82
C5-CH	20	138.63	138.07	-0.56	[	138.43	137.96	138.03	138.88	138.31	138.27	137.66	138.32	137.12	137.54	138.57	138.46	137.71
C6-C	19	146.88	148.29	1.41	[	148.20	148.46	148.41	148.51	148.06	148.11	149.29	148.38	147.82	148.06	148.08	148.11	147.64
C7-CH	18	118.26	116.76	-1.50	[	117.21	115.96	116.18	117.68	117.05	117.18	114.10	117.03	118.02	118.04	117.51	117.31	118.91
C8-C	15	71.78	73.75	1.97	[	74.23	73.17	73.11	74.10	74.33	74.27	74.33	74.17	73.66	73.85	74.21	74.21	73.59
C9-CH2	14	45.85	45.80	-0.04	[	43.78	47.94	49.48	43.13	44.39	44.19	47.37	43.91	41.74	41.67	43.67	43.65	41.87
C10-CH	11	78.34	78.79	0.45	[	79.07	78.76	78.55	78.54	78.69	78.76	79.21	78.83	80.27	79.19	78.20	78.69	79.73
C11-CH	12	149.56	149.32	-0.24	[	147.41	153.28	150.06	145.22	149.43	149.47	148.55	149.42	148.06	148.53	145.39	149.88	147.72
C12-C	8	133.05	132.49	-0.56	[	133.95	129.25	131.64	133.87	132.61	132.90	133.05	132.96	134.93	135.36	133.62	132.89	136.05
C13-CH2	7	24.37	26.30	1.93	[	27.04	26.38	25.33	27.67	25.76	25.66	27.07	25.62	26.67	24.31	27.54	22.70	25.98
C14-CH2	3	23.38	25.66	2.28	[	25.39	28.15	24.78	23.28	24.23	24.32	25.59	24.19	26.31	28.54	22.83	24.25	25.23
C15-C	4	156.72	159.86	3.14	[	158.83	158.20	165.26	159.09	158.58	158.48	158.86	158.43	157.79	159.89	158.30	162.11	159.32
C16-CH3**	6	23.42	22.01	-1.41	[	21.62	22.29	22.50	21.81	22.08	21.90	21.73	21.80	21.58	21.89	21.81	22.81	22.45
C17-CH3**	5	19.35	18.22	-1.13	[	17.06	22.22	17.60	16.75	16.88	16.89	16.99	16.99	17.11	18.22	16.66	17.18	18.63

C18-CH3	25	10.51	11.06	0.55	[	11.03	11.10	11.09	11.02	11.05	11.04	11.10	10.99	11.02	11.03	10.96	10.96	11.01
C19-CH3	16	30.14	28.58	-1.57	[	25.66	30.92	31.50	25.86	25.43	25.50	31.21	25.74	30.97	30.53	25.62	25.67	30.87
C20-C	9	173.51	173.22	-0.29	[	172.87	173.21	173.97	171.96	172.57	172.72	174.17	172.78	173.78	173.95	171.72	173.41	172.31

**14**	**15**	**16**	**17**	**18**	
136.79	136.76	136.81	136.76	135.42	]
189.50	189.49	189.47	189.45	192.58	]
169.40	169.40	169.07	169.10	168.92	]
130.97	130.98	131.89	132.02	133.49	]
137.97	138.04	135.89	135.85	133.29	]
147.59	147.58	148.23	148.27	147.25	]
115.93	115.83	117.84	117.74	120.59	]
74.19	74.15	73.45	73.40	71.11	]
47.55	47.67	45.32	45.22	51.51	]
77.44	77.35	77.12	77.09	78.40	]
147.05	147.08	147.29	147.29	152.93	]
134.61	134.55	134.43	134.39	130.43	]
27.07	27.09	27.08	27.00	22.33	]
25.73	25.44	25.57	25.60	25.09	]
158.96	158.90	158.85	158.83	166.11	]
21.71	21.69	21.68	21.67	22.80	]
17.05	17.02	16.98	17.03	17.54	]
11.09	11.11	11.13	11.11	11.12	]
31.20	31.16	31.41	31.45	33.20	]
174.36	174.35	174.41	174.42	174.56	]

13C chem shifts: RMSD=1.45ppm (MAE=1.21) N=20 {-1.57 3.14}

Fractions: 0.273 0.204 0.166 0.044 0.043 0.041 0.037 0.034 0.026 0.024 0.021 0.016 0.014 0.014 0.013 0.011 0.010 0.010

NMR parameters calculated for 10*S*,8*R*,6*E* diastereomer vs experimental data of kallopterolide E (**5**):

**C-nom**	**iGau**	**Exp**	**Calc**	**diff**		**1**	**2**	**3**	**4**	**5**	**6**	**7**	**8**	**9**	**10**	**11**	**12**	**13**
C1-C	2	135.53	136.12	0.59	[	136.52	134.28	138.47	136.10	134.75	134.64	136.72	134.72	136.84	136.53	136.00	133.98	136.45
C2-CH	1	190.64	192.69	2.05	[	189.47	200.85	193.97	190.96	189.92	190.03	189.50	189.93	189.52	189.18	190.03	188.96	189.01
C3-C	22	170.05	169.37	-0.68	[	169.46	169.27	169.27	169.64	169.36	169.35	169.21	169.43	169.01	169.89	169.48	169.45	169.90
C4-C	21	129.50	131.13	1.62	[	131.07	131.20	131.01	130.59	131.21	131.13	132.12	131.01	130.84	130.75	130.85	130.86	130.82
C5-CH	20	138.53	138.07	-0.47	[	138.43	137.96	138.03	138.88	138.31	138.27	137.66	138.32	137.12	137.54	138.57	138.46	137.71
C6-C	19	145.96	148.29	2.33	[	148.20	148.46	148.41	148.51	148.06	148.11	149.29	148.38	147.82	148.06	148.08	148.11	147.64
C7-CH	18	119.20	116.76	-2.44	[	117.21	115.96	116.18	117.68	117.05	117.18	114.10	117.03	118.02	118.04	117.51	117.31	118.91
C8-C	15	72.05	73.75	1.70	[	74.23	73.17	73.11	74.10	74.33	74.27	74.33	74.17	73.66	73.85	74.21	74.21	73.59
C9-CH2	14	45.10	45.80	0.70	[	43.78	47.94	49.48	43.13	44.39	44.19	47.37	43.91	41.74	41.67	43.67	43.65	41.87
C10-CH	11	78.82	78.79	-0.03	[	79.07	78.76	78.55	78.54	78.69	78.76	79.21	78.83	80.27	79.19	78.20	78.69	79.73
C11-CH	12	149.11	149.32	0.21	[	147.41	153.28	150.06	145.22	149.43	149.47	148.55	149.42	148.06	148.53	145.39	149.88	147.72
C12-C	8	133.19	132.49	-0.69	[	133.95	129.25	131.64	133.87	132.61	132.90	133.05	132.96	134.93	135.36	133.62	132.89	136.05
C13-CH2	7	24.37	26.30	1.93	[	27.04	26.38	25.33	27.67	25.76	25.66	27.07	25.62	26.67	24.31	27.54	22.70	25.98
C14-CH2	3	23.41	25.66	2.25	[	25.39	28.15	24.78	23.28	24.23	24.32	25.59	24.19	26.31	28.54	22.83	24.25	25.23

C15-C	4	156.79	159.86	3.07	[	158.83	158.20	165.26	159.09	158.58	158.48	158.86	158.43	157.79	159.89	158.30	162.11	159.32
C16-CH3**	6	23.35	22.01	-1.34	[	21.62	22.29	22.50	21.81	22.08	21.90	21.73	21.80	21.58	21.89	21.81	22.81	22.45
C17-CH3**	5	19.36	18.22	-1.14	[	17.06	22.22	17.60	16.75	16.88	16.89	16.99	16.99	17.11	18.22	16.66	17.18	18.63
C18-CH3	25	10.52	11.06	0.54	[	11.03	11.10	11.09	11.02	11.05	11.04	11.10	10.99	11.02	11.03	10.96	10.96	11.01
C19-CH3	16	28.92	28.58	-0.35	[	25.66	30.92	31.50	25.86	25.43	25.50	31.21	25.74	30.97	30.53	25.62	25.67	30.87
C20-C	9	173.15	173.22	0.07	[	172.87	173.21	173.97	171.96	172.57	172.72	174.17	172.78	173.78	173.95	171.72	173.41	172.31

**14**	**15**	**16**	**17**	**18**	
136.79	136.76	136.81	136.76	135.42	]
189.50	189.49	189.47	189.45	192.58	]
169.40	169.40	169.07	169.10	168.92	]
130.97	130.98	131.89	132.02	133.49	]
137.97	138.04	135.89	135.85	133.29	]
147.59	147.58	148.23	148.27	147.25	]
115.93	115.83	117.84	117.74	120.59	]
74.19	74.15	73.45	73.40	71.11	]
47.55	47.67	45.32	45.22	51.51	]
77.44	77.35	77.12	77.09	78.40	]
147.05	147.08	147.29	147.29	152.93	]
134.61	134.55	134.43	134.39	130.43	]
27.07	27.09	27.08	27.00	22.33	]
25.73	25.44	25.57	25.60	25.09	]
158.96	158.90	158.85	158.83	166.11	]
21.71	21.69	21.68	21.67	22.80	]
17.05	17.02	16.98	17.03	17.54	]
11.09	11.11	11.13	11.11	11.12	]
31.20	31.16	31.41	31.45	33.20	]
174.36	174.35	174.41	174.42	174.56	]

13C chem shifts: RMSD=1.50ppm (MAE=1.21) N=20 {-2.44 3.07}

Fractions: 0.273 0.204 0.166 0.044 0.043 0.041 0.037 0.034 0.026 0.024 0.021 0.016 0.014 0.014 0.013 0.011 0.010 0.010

Conformer 1
Energy: -1227.88792 Hartree (Rel: 0.0 kcal/mol)
XYZ coordinates for conf 1:

C	6.48912	-0.54647	-1.48218
C	5.80318	0.19175	-0.40885
C	4.52266	-0.45550	0.08119
C	6.27447	1.38040	0.06079
C	7.55597	2.04868	-0.38034
C	5.54678	2.18042	1.11458
C	3.28760	-0.04451	-0.76253
C	2.00922	-0.56844	-0.18512
C	1.45792	-0.01944	1.07868
O	0.29970	-0.70078	1.37918
C	0.02757	-1.68620	0.34720
C	1.18019	-1.53490	-0.60249
O	1.87844	0.86776	1.78741
C	-1.34696	-1.42766	-0.28574
C	-2.54311	-1.35686	0.71267
C	-2.60875	-2.59414	1.63476
O	-2.45013	-0.17416	1.50491
C	-3.80740	-1.30030	-0.10907
C	-4.65463	-0.27070	-0.26525
C	-4.70015	1.08922	0.24899
C	-5.79552	1.70485	-0.24614
C	-6.49432	0.72785	-1.10777
O	-5.76882	-0.45133	-1.08857
O	-7.51385	0.83738	-1.75276
C	-6.31219	3.09044	-0.04203
O	6.06689	-1.59209	-1.96484
H	7.42972	-0.11782	-1.87113
H	4.34067	-0.21461	1.13077
H	4.63663	-1.54137	0.01058
H	8.18581	1.45456	-1.04161
H	8.15097	2.31127	0.50402
H	7.32601	2.99505	-0.88782
H	4.52178	1.85525	1.29724
H	5.52535	3.23989	0.82955
H	6.09681	2.13062	2.06533
H	3.23140	1.05044	-0.80355
H	3.41231	-0.40629	-1.78758
H	0.04573	-2.66745	0.83567
H	1.28121	-2.14366	-1.49433
H	-1.31653	-0.48092	-0.83613
H	-1.52208	-2.22637	-1.01635
H	-2.66394	-3.52646	1.06129
H	-3.49518	-2.52446	2.27176
H	-1.72950	-2.63845	2.28617
H	-1.53314	-0.12083	1.82838
H	-4.05179	-2.21461	-0.64439
H	-3.95091	1.48578	0.91696
H	-5.66500	3.65925	0.63067
H	-7.32401	3.07018	0.38067
H	-6.38013	3.62574	-0.99695

Conformer 2
Energy: -1227.88786 Hartree (Rel: 0.2 kcal/mol)
XYZ coordinates for conf 2:

C	3.01148	0.51853	1.43811
C	3.24243	1.34855	0.24477
C	4.06603	0.66453	-0.83657
C	2.76183	2.62072	0.14717
C	1.94684	3.30497	1.21686
C	3.01933	3.49342	-1.05399
C	3.19688	0.01574	-1.95318

C	2.20721	-0.97275	-1.41336
C	2.54745	-2.39996	-1.18761
O	1.46184	-3.02475	-0.63328
C	0.40268	-2.06080	-0.41342
C	0.94898	-0.78759	-0.98966
O	3.57808	-2.99058	-1.43318
C	0.10659	-2.03276	1.09699
C	-0.96721	-1.01434	1.57009
C	-1.14016	-1.16023	3.09992
O	-0.57609	0.32387	1.26477
C	-2.28475	-1.31104	0.89871
C	-3.11214	-0.46371	0.26559
C	-3.07339	0.96213	-0.01875
C	-4.19548	1.30311	-0.68872
C	-4.99594	0.07129	-0.85555
O	-4.30229	-0.96979	-0.26212
O	-6.06743	-0.10167	-1.39315
C	-4.65841	2.62297	-1.21022
O	2.08910	0.60426	2.25054
H	3.76996	-0.27567	1.58965
H	4.67569	-0.12348	-0.37751
H	4.77097	1.35776	-1.30519
H	0.87547	3.17916	1.00621
H	2.14818	4.38200	1.21170
H	2.12210	2.90407	2.21532
H	3.52992	2.98962	-1.87499
H	3.61663	4.36662	-0.75812
H	2.06777	3.88979	-1.43265
H	2.66522	0.80194	-2.49986
H	3.86370	-0.48753	-2.66232
H	-0.47677	-2.41221	-0.96644
H	0.37719	0.12986	-0.99990
H	-0.19990	-3.04201	1.39462
H	1.04065	-1.81217	1.62496
H	-1.48803	-2.16099	3.37623
H	-1.86701	-0.42492	3.45707
H	-0.18090	-0.97880	3.59880
H	0.28126	0.50392	1.70400
H	-2.61454	-2.34498	0.96988
H	-2.25669	1.59697	0.28932
H	-3.93370	3.41177	-0.99187
H	-5.62155	2.90117	-0.76547
H	-4.81305	2.58069	-2.29523

Conformer 3
Energy: -1227.88720 Hartree (Rel: 0.3 kcal/mol)
XYZ coordinates for conf 3:

C	2.37181	1.19129	1.51809
C	3.40271	1.34929	0.49019
C	4.00060	0.07038	-0.07511
C	3.77121	2.59508	0.06669
C	3.24442	3.90509	0.60429
C	4.79761	2.80598	-1.01831
C	3.33580	-0.43981	-1.39071
C	2.09299	-1.25061	-1.16301
C	2.13379	-2.73451	-1.22191
O	0.89228	-3.22090	-0.91591
C	0.01309	-2.12330	-0.57371
C	0.84279	-0.89910	-0.83431
O	3.06088	-3.46441	-1.50391
C	-0.41301	-2.32959	0.89149
C	-1.39431	-1.28489	1.48409
C	-1.73481	-1.69679	2.93589
O	-0.81430	0.01761	1.48689

C	-2.66941	-1.26018	0.67849
C	-3.30690	-0.20198	0.15209
C	-3.04459	1.22852	0.13429
C	-4.03919	1.84522	-0.54011
C	-4.98180	0.79623	-0.98381
O	-4.49940	-0.42357	-0.54101
O	-6.00990	0.88263	-1.61831
C	-4.26438	3.28912	-0.84481
O	1.97720	0.10339	1.94449
H	1.90871	2.10909	1.91279
H	3.92350	-0.71232	0.68499
H	5.06760	0.21628	-0.26511
H	2.60012	3.82939	1.47889
H	2.68982	4.43079	-0.18441
H	4.09032	4.55278	0.86769
H	5.06301	1.90148	-1.56501
H	5.71572	3.22367	-0.58131
H	4.43522	3.54998	-1.73851
H	3.12160	0.41029	-2.04821
H	4.05509	-1.08042	-1.91221
H	-0.85761	-2.18279	-1.23761
H	0.44360	0.09750	-0.70471
H	-0.86352	-3.32589	0.96589
H	0.49419	-2.33840	1.50739
H	-2.24151	-2.66669	2.97569
H	-2.38611	-0.94168	3.38559
H	-0.81511	-1.76579	3.52879
H	0.07570	-0.03530	1.89459
H	-3.14361	-2.22888	0.53849
H	-2.18139	1.66961	0.60909
H	-3.46318	3.91052	-0.43601
H	-5.21888	3.63093	-0.42631
H	-4.31798	3.45522	-1.92761

Conformer 4
Energy: -1227.88776 Hartree (Rel: 1.1 kcal/mol)
XYZ coordinates for conf 4:

C	2.07739	1.80340	0.46900
C	3.52569	1.59690	0.29730
C	4.11069	0.44260	1.09190
C	4.25889	2.39460	-0.53020
C	3.72519	3.56220	-1.32830
C	5.73899	2.19671	-0.74870
C	4.19179	-0.89920	0.31150
C	2.84780	-1.48730	0.01140
C	2.03670	-2.12440	1.07890
O	0.84900	-2.54910	0.52300
C	0.80810	-2.18980	-0.88260
C	2.14260	-1.54560	-1.12540
O	2.29790	-2.30750	2.24480
C	-0.38470	-1.26031	-1.15150
C	-1.77900	-1.82161	-0.73550
C	-2.05560	-3.21251	-1.34650
O	-1.87550	-1.89981	0.68590
C	-2.81890	-0.85381	-1.24680
C	-3.55271	0.02339	-0.54380
C	-3.63981	0.36539	0.86700
C	-4.55481	1.34689	1.02070
C	-5.08971	1.66009	-0.32100
O	-4.45561	0.83569	-1.23510
O	-5.92511	2.46719	-0.66550
C	-5.02441	2.05959	2.24560
O	1.36039	1.08950	1.16290
H	1.62249	2.65420	-0.06650

```
H          3.49199        0.28420        1.97920
H          5.11539        0.69020        1.44570
H          4.30929        4.46150       -1.09370
H          2.67379        3.79610       -1.16760
H          3.86989        3.37330       -2.40030
H          6.15059        1.31771       -0.25270
H          6.28949        3.07961       -0.39570
H          5.95099        2.11401       -1.82290
H          4.74899       -0.75740       -0.62080
H          4.75480       -1.61190        0.92690
H          0.70650       -3.12490       -1.44560
H          2.43899       -1.17690       -2.10130
H         -0.22641       -0.32190       -0.60940
H         -0.38621       -1.03110       -2.22410
H         -1.97140       -3.20171       -2.43940
H         -3.06700       -3.53231       -1.07890
H         -1.35410       -3.95411       -0.95060
H         -1.03020       -2.25901        1.01030
H         -2.97681       -0.86691       -2.32260
H         -3.04181       -0.12171        1.62210
H         -4.51881        1.68489        3.13940
H         -6.10611        1.93559        2.37760
H         -4.83781        3.13749        2.16530
```

Conformer 5

```
Energy: -1227.88742 Hartree (Rel:  1.1 kcal/mol)
XYZ coordinates for conf 5:
C         -4.01560       -2.50189       -1.21579
C         -3.40530       -1.88779       -0.02190
C         -4.31540       -0.90529        0.69151
C         -2.13820       -2.20739        0.36880
C         -1.23040       -3.19009       -0.33809
C         -1.46670       -1.61580        1.58500
C         -4.44809        0.46991       -0.02179
C         -3.15739        1.22281       -0.11689
C         -2.46929        1.75241        1.08580
O         -1.29079        2.34531        0.68610
C         -1.14919        2.23670       -0.75519
C         -2.40049        1.52581       -1.18080
O         -2.79949        1.71581        2.24960
C          0.13441        1.47410       -1.12339
C          1.43121        1.94260       -0.39659
C          1.65291        3.46570       -0.52340
O          1.36931        1.57470        0.98081
C          2.59101        1.22790       -1.04370
C          3.41961        0.32030       -0.50319
C          3.53471       -0.28960        0.81261
C          4.57140       -1.15520        0.80161
C          5.15790       -1.11780       -0.55490
O          4.43011       -0.21190       -1.30790
O          6.09640       -1.72490       -1.02180
C          5.12130       -2.03310        1.87651
O         -5.14830       -2.24269       -1.60800
H         -3.41240       -3.23729       -1.77599
H         -4.00190       -0.74069        1.72321
H         -5.32090       -1.33759        0.72620
H         -0.98470       -4.01620        0.34220
H         -0.27850       -2.70010       -0.58110
H         -1.62850       -3.61999       -1.25589
H         -2.09560       -0.94439        2.16800
H         -0.56880       -1.05700        1.28510
H         -1.11690       -2.42140        2.24431
H         -4.85989        0.31731       -1.02290
H         -5.17129        1.07061        0.54421
```

H	-1.11029	3.26080	-1.14369
H	-2.60869	1.29001	-2.21880
H	0.00081	0.41030	-0.89950
H	0.25901	1.56480	-2.20910
H	1.69641	3.78240	-1.57160
H	2.59481	3.73740	-0.03830
H	0.84561	4.01590	-0.02859
H	0.52871	1.92690	1.32441
H	2.77151	1.48970	-2.08350
H	2.87241	-0.04630	1.62921
H	4.55900	-1.91990	2.80710
H	6.17350	-1.79460	2.07321
H	5.08830	-3.08680	1.57401

Conformer 6

Energy: -1227.88732 Hartree (Rel: 1.1 kcal/mol)

XYZ coordinates for conf 6:

C	4.30280	2.30211	-1.26680
C	3.62700	1.80581	-0.05390
C	4.41280	0.74561	0.69500
C	2.41360	2.29491	0.33000
C	1.63810	3.37241	-0.39570
C	1.68200	1.82541	1.56440
C	4.41430	-0.64999	0.01260
C	3.06750	-1.30119	-0.06550
C	2.34680	-1.76539	1.14580
O	1.14990	-2.32389	0.75230
C	1.01930	-2.24069	-0.69140
C	2.29720	-1.58459	-1.12540
O	2.66790	-1.70469	2.31140
C	-0.24420	-1.45519	-1.07290
C	-1.56860	-1.94729	-0.41270
C	-1.79930	-3.45959	-0.62440
O	-1.55480	-1.65409	0.98310
C	-2.69900	-1.18819	-1.06270
C	-3.50800	-0.26679	-0.51600
C	-3.62250	0.32171	0.80950
C	-4.63010	1.22101	0.80050
C	-5.19810	1.22771	-0.56440
O	-4.48930	0.31261	-1.32430
O	-6.10930	1.87401	-1.03260
C	-5.16620	2.09721	1.88370
O	5.39770	1.90051	-1.64630
H	3.78520	3.07791	-1.85740
H	4.06840	0.63971	1.72440
H	5.45780	1.07031	0.73970
H	1.40600	4.18691	0.30280
H	0.67210	2.97051	-0.72910
H	2.13670	3.80761	-1.26040
H	2.13960	0.97471	2.06760
H	0.64990	1.55281	1.30670
H	1.60690	2.65101	2.28590
H	4.83470	-0.55699	-0.99210
H	5.08470	-1.30009	0.58930
H	0.95530	-3.27049	-1.06190
H	2.52210	-1.38519	-2.16750
H	-0.11010	-0.40259	-0.80050
H	-0.33410	-1.50179	-2.16480
H	-1.80020	-3.72639	-1.68730
H	-2.76390	-3.74289	-0.19360
H	-1.02100	-4.04279	-0.12100
H	-0.70620	-1.98159	1.33120
H	-2.86920	-1.42139	-2.11100
H	-2.97950	0.04211	1.62990

H	-4.61780	1.95291	2.81820
H	-6.22700	1.88631	2.06540
H	-5.09970	3.15421	1.59860

Conformer 7
Energy: -1227.88936 Hartree (Rel: 1.2 kcal/mol)
XYZ coordinates for conf 7:

C	5.01600	2.22290	1.06690
C	5.05010	0.93850	0.34840
C	3.77940	0.11900	0.45960
C	6.13080	0.55600	-0.38760
C	7.42290	1.32970	-0.51260
C	6.15670	-0.73060	-1.17760
C	2.73910	0.47840	-0.63330
C	1.55100	-0.43210	-0.60190
C	1.65750	-1.85340	-1.01900
O	0.43380	-2.44610	-0.87440
C	-0.51840	-1.48220	-0.36340
C	0.28320	-0.21900	-0.22460
O	2.62280	-2.46170	-1.43520
C	-1.12800	-2.05321	0.92590
C	-2.33080	-1.25721	1.49750
C	-2.83540	-1.95091	2.78080
O	-1.94290	0.08749	1.80410
C	-3.45120	-1.22061	0.48830
C	-4.15080	-0.16211	0.04810
C	-4.10500	1.26659	0.32090
C	-5.06260	1.87919	-0.40780
C	-5.75860	0.83049	-1.18460
O	-5.17310	-0.38621	-0.87540
O	-6.67730	0.91409	-1.96850
C	-5.44730	3.31809	-0.50220
O	4.04570	2.62210	1.70250
H	5.91930	2.85620	1.01510
H	3.32820	0.31480	1.43700
H	3.99860	-0.94980	0.40990
H	7.50390	2.20590	0.12980
H	7.56130	1.65570	-1.55210
H	8.26720	0.66631	-0.28400
H	5.18410	-1.21280	-1.28310
H	6.84420	-1.44510	-0.70240
H	6.55820	-0.54340	-2.18170
H	2.42060	1.51750	-0.50340
H	3.21650	0.40100	-1.61820
H	-1.30220	-1.37061	-1.12360
H	-0.15560	0.70239	0.13550
H	-1.44970	-3.07911	0.71330
H	-0.34580	-2.11501	1.69290
H	-3.19080	-2.96581	2.57680
H	-3.65690	-1.37251	3.21330
H	-2.02450	-2.01831	3.51710
H	-1.32730	0.05219	2.55480
H	-3.74290	-2.19101	0.09400
H	-3.40060	1.71089	1.00770
H	-4.81970	3.93959	0.14180
H	-6.49530	3.46029	-0.21190
H	-5.35440	3.67829	-1.53400

Conformer 8
Energy: -1227.88694 Hartree (Rel: 1.2 kcal/mol)
XYZ coordinates for conf 8:

C	-5.86240	-0.83590	-1.54619
C	-5.28990	-0.47190	-0.23719
C	-4.57380	-1.61410	0.45901

C	-5.43060	0.78480	0.27171
C	-6.17290	1.91980	-0.39879
C	-4.86750	1.20630	1.60751
C	-3.19480	-1.97490	-0.15719
C	-2.15710	-0.90040	-0.03849
C	-1.58390	-0.49970	1.27051
O	-0.65300	0.49310	1.05211
C	-0.55570	0.77140	-0.36939
C	-1.56220	-0.15750	-0.98239
O	-1.83000	-0.90730	2.38351
C	0.88200	0.55000	-0.85999
C	1.98340	1.33840	-0.08669
C	1.67400	2.84940	-0.00919
O	2.12800	0.81230	1.23171
C	3.27920	1.14780	-0.83659
C	4.34730	0.41750	-0.47869
C	4.66420	-0.41240	0.67311
C	5.90060	-0.93040	0.50881
C	6.41710	-0.42790	-0.78199
O	5.44410	0.38230	-1.34299
O	7.47610	-0.62680	-1.33539
C	6.70140	-1.83990	1.38061
O	-5.77230	-1.95520	-2.03919
H	-6.39880	-0.04870	-2.10429
H	-5.19860	-2.50890	0.36521
H	-4.45140	-1.42310	1.52591
H	-5.47590	2.74120	-0.61199
H	-6.92080	2.32520	0.29501
H	-6.68370	1.66330	-1.32559
H	-4.19550	0.48200	2.06611
H	-5.68960	1.40250	2.31011
H	-4.32580	2.15520	1.50121
H	-2.82680	-2.87190	0.35761
H	-3.33100	-2.23630	-1.20959
H	-0.84840	1.81910	-0.50649
H	-1.73880	-0.19700	-2.05169
H	1.12530	-0.51590	-0.79099
H	0.90730	0.82400	-1.92149
H	1.53310	3.28780	-1.00379
H	2.50320	3.36460	0.48421
H	0.76890	3.02850	0.58071
H	1.23140	0.72190	1.60101
H	3.34230	1.66430	-1.79129
H	3.98020	-0.55320	1.49591
H	7.64970	-1.36910	1.66671
H	6.95630	-2.76470	0.84891
H	6.15380	-2.10060	2.29001

Conformer 9
Energy: -1227.88661 Hartree (Rel: 1.4 kcal/mol)
XYZ coordinates for conf 9:

C	3.90550	-0.60831	-1.81690
C	3.40620	-0.46381	-0.43950
C	1.90310	-0.59950	-0.29860
C	4.24850	-0.26911	0.61280
C	5.74590	-0.09211	0.51310
C	3.75790	-0.19681	2.03960
C	1.44780	-2.07950	-0.20900
C	-0.01780	-2.21140	0.06650
C	-0.58600	-1.87260	1.39320
O	-1.94870	-2.08759	1.35660
C	-2.32570	-2.59819	0.04950
C	-1.03540	-2.58869	-0.71940
O	-0.03570	-1.46720	2.39290

C	-3.53230	-1.83409	-0.52700
C	-3.54820	-0.28799	-0.38780
C	-4.80000	0.25422	-1.10470
O	-3.71430	0.06871	0.98660
C	-2.32309	0.34291	-1.01970
C	-1.61399	1.38521	-0.55560
C	-1.70139	2.20161	0.64490
C	-0.73019	3.13951	0.60560
C	0.02341	2.93550	-0.64980
O	-0.54739	1.86350	-1.31860
O	0.97242	3.53630	-1.10210
C	-0.38218	4.22541	1.56870
O	3.18560	-0.86391	-2.77640
H	4.99200	-0.49051	-1.97790
H	1.42880	-0.14540	-1.17340
H	1.54370	-0.05720	0.57840
H	6.25450	-0.93812	0.99490
H	6.04241	0.80419	1.07330
H	6.13500	0.00468	-0.49990
H	2.74720	-0.58120	2.18440
H	3.78091	0.84529	2.38980
H	4.43540	-0.75601	2.69670
H	1.70010	-2.59090	-1.14250
H	2.00100	-2.57400	0.59950
H	-2.64171	-3.63949	0.20060
H	-0.97660	-2.90839	-1.75440
H	-3.61840	-2.11579	-1.58360
H	-4.43650	-2.19658	-0.02670
H	-4.74330	0.08682	-2.18460
H	-4.88489	1.32962	-0.92260
H	-5.69670	-0.23988	-0.71550
H	-3.08120	-0.46789	1.49750
H	-2.03060	-0.04829	-1.99150
H	-2.45179	2.04641	1.40590
H	-1.05008	4.21661	2.43410
H	-0.44708	5.20850	1.08690
H	0.64992	4.11530	1.92290

Conformer 10

Energy: -1227.88917 Hartree (Rel: 1.5 kcal/mol)

XYZ coordinates for conf 10:

C	4.36637	-1.51040	-1.16916
C	3.53535	-0.57444	-0.39972
C	2.05134	-0.87943	-0.41251
C	4.06859	0.47306	0.28872
C	5.52783	0.85680	0.30797
C	3.19969	1.40405	1.10578
C	1.65705	-1.98422	0.60411
C	0.17180	-2.14156	0.72436
C	-0.60349	-1.38299	1.73622
O	-1.93423	-1.72976	1.62183
C	-2.09824	-2.68385	0.53995
C	-0.70386	-2.86233	0.01067
O	-0.22451	-0.58181	2.56027
C	-3.19003	-2.22828	-0.45608
C	-3.36759	-0.69987	-0.67726
C	-4.42663	-0.48520	-1.77511
O	-3.91825	-0.11235	0.50361
C	-2.07460	-0.03689	-1.11056
C	-1.62055	1.16991	-0.73201
C	-2.12663	2.19644	0.16566
C	-1.25929	3.23201	0.17860
C	-0.14247	2.87514	-0.72004
O	-0.41102	1.62779	-1.25948

O	0.87023	3.48202	-0.99575
C	-1.29856	4.52906	0.91617
O	3.91756	-2.48231	-1.76978
H	5.45470	-1.32119	-1.19278
H	1.77615	-1.22906	-1.41300
H	1.46874	0.02364	-0.22234
H	5.91918	0.77327	1.33074
H	5.62612	1.91401	0.02770
H	6.17070	0.27398	-0.35114
H	2.37496	0.88904	1.60620
H	2.75934	2.17641	0.45894
H	3.79389	1.91964	1.86714
H	2.11362	-2.92823	0.29218
H	2.05877	-1.73036	1.59149
H	-2.43231	-3.62321	0.99954
H	-0.48272	-3.53248	-0.81343
H	-2.99646	-2.72676	-1.41338
H	-4.15885	-2.59139	-0.09890
H	-4.07399	-0.85336	-2.74343
H	-4.64459	0.58290	-1.86631
H	-5.35052	-1.01097	-1.51113
H	-3.33657	-0.37761	1.23982
H	-1.47903	-0.57277	-1.84564
H	-3.05892	2.09878	0.70186
H	-2.19566	4.60462	1.53604
H	-1.28325	5.37513	0.21846
H	-0.41809	4.63532	1.56129

Conformer 11

Energy: -1227.88654 Hartree (Rel: 1.5 kcal/mol)

XYZ coordinates for conf 11:

C	4.89580	-0.51230	1.20550
C	5.24380	0.09920	-0.09010
C	4.63760	1.47000	-0.33260
C	6.04500	-0.54550	-0.98460
C	6.67740	-1.90350	-0.78080
C	6.40460	0.04420	-2.32620
C	3.31180	1.44970	-1.14240
C	2.19050	0.77230	-0.41690
C	1.51040	1.42550	0.73000
O	0.56210	0.55360	1.22120
C	0.55630	-0.66370	0.43240
C	1.62760	-0.42990	-0.59330
O	1.67250	2.52580	1.20290
C	-0.83960	-0.90880	-0.15670
C	-2.00810	-0.93530	0.87580
C	-1.74270	-1.92580	2.03090
O	-2.22280	0.37140	1.40540
C	-3.24740	-1.37760	0.13760
C	-4.34570	-0.66230	-0.15250
C	-4.75780	0.70870	0.10480
C	-5.98570	0.90020	-0.42410
C	-6.39800	-0.37580	-1.04590
O	-5.37610	-1.29070	-0.85690
O	-7.41500	-0.67070	-1.63460
C	-6.86150	2.10900	-0.44470
O	4.15260	0.00680	2.03060
H	5.34100	-1.49630	1.43390
H	5.34500	2.11840	-0.85760
H	4.43850	1.93410	0.63670
H	7.76970	-1.81070	-0.84320
H	6.37950	-2.57520	-1.59650
H	6.43710	-2.39390	0.16140
H	5.99420	1.03880	-2.50080

H	6.05230	-0.61580	-3.13060
H	7.49640	0.09810	-2.43210
H	3.02300	2.48920	-1.34130
H	3.47120	0.96200	-2.11010
H	0.83590	-1.48020	1.10870
H	1.87750	-1.16720	-1.34840
H	-1.06180	-0.12670	-0.89110
H	-0.80060	-1.86340	-0.69530
H	-1.55730	-2.94080	1.66120
H	-2.61220	-1.95040	2.69410
H	-0.87750	-1.60740	2.62170
H	-1.34890	0.72390	1.65240
H	-3.23840	-2.41050	-0.20200
H	-4.13920	1.41310	0.63960
H	-7.82480	1.89990	0.03620
H	-7.08300	2.41261	-1.47510
H	-6.38940	2.94880	0.07180

Conformer 12
Energy: -1227.88648 Hartree (Rel: 1.7 kcal/mol)
XYZ coordinates for conf 12:

C	-5.05600	-1.44595	-0.33347
C	-5.14334	-0.11542	0.29254
C	-4.68358	-0.05457	1.73965
C	-5.59738	0.96526	-0.40251
C	-6.02496	0.96755	-1.85077
C	-5.68443	2.33551	0.22163
C	-3.30734	0.62831	1.96368
C	-2.21324	0.07820	1.09541
C	-1.79268	0.76325	-0.15204
O	-0.80741	0.00781	-0.75025
C	-0.52269	-1.16076	0.06256
C	-1.47294	-1.03235	1.21613
O	-2.17771	1.80346	-0.63633
C	0.95877	-1.18057	0.46408
C	1.97374	-1.09413	-0.71672
C	1.70032	-2.16106	-1.79963
O	1.93165	0.20514	-1.30425
C	3.34886	-1.33074	-0.14271
C	4.36421	-0.46062	-0.02380
C	4.54226	0.94072	-0.37046
C	5.79014	1.32035	-0.01996
C	6.45562	0.14100	0.57296
O	5.55623	-0.91134	0.54909
O	7.57251	0.01325	1.02450
C	6.48271	2.63627	-0.15174
O	-4.64220	-2.45158	0.23404
H	-5.39830	-1.52803	-1.37973
H	-4.62264	-1.08277	2.10602
H	-5.42265	0.46428	2.36133
H	-7.08550	1.24317	-1.92187
H	-5.88316	0.02722	-2.38189
H	-5.46689	1.74493	-2.38872
H	-5.73150	2.31396	1.31252
H	-6.56050	2.87670	-0.15383
H	-4.79816	2.91743	-0.06580
H	-3.38745	1.70227	1.76820
H	-3.03440	0.51110	3.01873
H	-0.76655	-2.03860	-0.54731
H	-1.51910	-1.76840	2.01121
H	1.15958	-0.34069	1.13836
H	1.12831	-2.10424	1.03020
H	1.70192	-3.17441	-1.38235
H	2.47311	-2.10073	-2.57131

H	0.73170	-1.98474	-2.27901
H	0.99241	0.42379	-1.44064
H	3.53006	-2.33909	0.22162
H	3.76587	1.53010	-0.83376
H	5.83040	3.38275	-0.61245
H	7.38873	2.53993	-0.76228
H	6.80178	3.00876	0.82926

Conformer 13

Energy: -1227.88681 Hartree (Rel: 1.7 kcal/mol)

XYZ coordinates for conf 13:

C	4.27393	-1.31137	0.96568
C	3.43720	-0.60544	-0.01551
C	1.94217	-0.74517	0.19888
C	3.97946	0.11545	-1.03650
C	5.45492	0.33751	-1.26577
C	3.11199	0.81637	-2.05985
C	1.39167	-2.12315	-0.25512
C	-0.09924	-2.22580	-0.13241
C	-0.80494	-2.11487	1.16931
O	-2.16344	-2.24897	0.94325
C	-2.40240	-2.49829	-0.46591
C	-1.03378	-2.40338	-1.07689
O	-0.36925	-1.93067	2.28178
C	-3.52149	-1.59413	-1.01511
C	-3.49563	-0.09000	-0.63252
C	-4.65519	0.62192	-1.35601
O	-3.77016	0.05799	0.76222
C	-2.19346	0.56424	-1.04943
C	-1.49032	1.48418	-0.36910
C	-1.66079	2.12676	0.92328
C	-0.64384	2.99539	1.11726
C	0.22975	2.91451	-0.07129
O	-0.32466	1.99481	-0.94645
O	1.26400	3.49211	-0.33062
C	-0.34307	3.90472	2.26212
O	3.82285	-1.98780	1.88431
H	5.36961	-1.22469	0.85211
H	1.40261	0.04886	-0.32131
H	1.73023	-0.63945	1.26721
H	5.64897	1.41595	-1.33879
H	5.74758	-0.09208	-2.23333
H	6.11352	-0.07023	-0.49959
H	2.26141	0.20587	-2.37667
H	3.69418	1.07653	-2.94943
H	2.70327	1.74960	-1.64804
H	1.67674	-2.31854	-1.29517
H	1.86020	-2.89594	0.36498
H	-2.75672	-3.53540	-0.54605
H	-0.86637	-2.52809	-2.14162
H	-3.51777	-1.70301	-2.10675
H	-4.48065	-1.98627	-0.66043
H	-4.50648	0.62601	-2.44031
H	-4.71736	1.65762	-1.00924
H	-5.60125	0.11868	-1.12946
H	-3.21970	-0.59509	1.23193
H	-1.82069	0.30294	-2.03721
H	-2.49210	1.91258	1.57866
H	-1.10012	3.81681	3.04559
H	-0.30217	4.94933	1.93050
H	0.63679	3.67162	2.69585

Conformer 14
Energy: -1227.88673 Hartree (Rel: 1.8 kcal/mol)
XYZ coordinates for conf 14:

C	-6.39189	0.99491	0.07350
C	-5.50109	-0.14559	-0.19510
C	-4.03869	0.21961	-0.35920
C	-5.96670	-1.42498	-0.24320
C	-7.41520	-1.83428	-0.10930
C	-5.05440	-2.61009	-0.45210
C	-3.27949	0.26391	0.99290
C	-1.80689	0.45781	0.80690
C	-0.95179	-0.62409	0.25250
O	0.33711	-0.17349	0.18060
C	0.40521	1.18991	0.66140
C	-1.01179	1.50891	1.04430
O	-1.26500	-1.74409	-0.09580
C	0.98581	2.11451	-0.41420
C	2.53021	2.03921	-0.56530
C	2.98771	3.05861	-1.62980
O	3.14061	2.34591	0.69370
C	2.96491	0.66161	-0.99410
C	3.94261	-0.10210	-0.48150
C	4.87191	0.04990	0.62680
C	5.65850	-1.04620	0.68890
C	5.23940	-1.94670	-0.40610
O	4.19980	-1.33550	-1.08430
O	5.66330	-3.03340	-0.73360
C	6.76530	-1.40260	1.62510
O	-6.00229	2.15372	0.17520
H	-7.46779	0.77892	0.19810
H	-3.97969	1.21231	-0.81560
H	-3.53629	-0.47889	-1.03180
H	-7.55030	-2.43038	0.80320
H	-7.68560	-2.49008	-0.94710
H	-8.12999	-1.01238	-0.08510
H	-3.99380	-2.38849	-0.32570
H	-5.20100	-3.01889	-1.46230
H	-5.32280	-3.41459	0.24420
H	-3.69089	1.06581	1.61360
H	-3.44349	-0.68209	1.52400
H	1.07181	1.19411	1.53260
H	-1.30249	2.47071	1.45340
H	0.49221	1.90601	-1.37030
H	0.73641	3.14951	-0.14500
H	2.57261	2.82141	-2.61450
H	4.07921	3.05500	-1.70120
H	2.65401	4.06841	-1.35800
H	3.01841	3.29591	0.85580
H	2.43251	0.25731	-1.85020
H	4.88571	0.91990	1.26550
H	6.92781	-0.61500	2.36570
H	7.70150	-1.56580	1.07760
H	6.54200	-2.33760	2.15310

Conformer 15
Energy: -1227.88686 Hartree (Rel: 1.8 kcal/mol)
XYZ coordinates for conf 15:

C	-6.29560	0.58469	1.21090
C	-5.43460	-0.39681	0.53120
C	-3.94800	-0.18431	0.73980
C	-5.95110	-1.38291	-0.25420
C	-7.42310	-1.64611	-0.47280
C	-5.07790	-2.35701	-1.00860
C	-3.33690	0.80519	-0.28690

C	-1.84540	0.87709	-0.18090
C	-0.98700	-0.25700	-0.61260
O	0.32130	0.07130	-0.38930
C	0.40150	1.39960	0.17840
C	-1.03231	1.83570	0.28100
O	-1.31220	-1.32340	-1.09310
C	1.26879	2.31130	-0.69630
C	2.79949	2.09300	-0.54410
C	3.54739	3.12060	-1.41970
O	3.16469	2.26900	0.82940
C	3.19460	0.70550	-0.97910
C	3.96710	-0.18319	-0.33470
C	4.64470	-0.19179	0.95220
C	5.29710	-1.36589	1.09280
C	5.04560	-2.15439	-0.13220
O	4.23700	-1.40040	-0.96410
O	5.43070	-3.25919	-0.44680
C	6.13780	-1.88789	2.21050
O	-5.86610	1.51649	1.88340
H	-7.38760	0.46898	1.09200
H	-3.40850	-1.13251	0.69180
H	-3.79510	0.22749	1.74200
H	-8.09750	-1.05712	0.14800
H	-7.63390	-2.70692	-0.28480
H	-7.68130	-1.46352	-1.52450
H	-4.02760	-2.06811	-1.06530
H	-5.45580	-2.48251	-2.03120
H	-5.13490	-3.34921	-0.53790
H	-3.60090	0.47499	-1.29940
H	-3.77501	1.79709	-0.13940
H	0.86890	1.30350	1.16630
H	-1.31951	2.80500	0.67480
H	0.96389	2.19980	-1.74310
H	1.06129	3.35130	-0.41160
H	3.32469	2.97770	-2.48190
H	4.62629	3.01711	-1.27260
H	3.24899	4.14150	-1.14760
H	3.08389	3.21470	1.03620
H	2.82530	0.40760	-1.95630
H	4.59820	0.63431	1.64530
H	6.20900	-1.16259	3.02540
H	7.15090	-2.12069	1.86050
H	5.72010	-2.82039	2.60910

Conformer 16
Energy: -1227.88789 Hartree (Rel: 1.9 kcal/mol)
XYZ coordinates for conf 16:

C	-6.23298	0.97447	0.14607
C	-5.32924	-0.13381	-0.20265
C	-3.87299	0.26095	-0.35196
C	-5.77872	-1.41341	-0.33143
C	-7.22069	-1.85014	-0.21406
C	-4.85290	-2.56999	-0.62345
C	-3.10155	0.21614	0.99314
C	-1.63410	0.44842	0.81002
C	-0.76751	-0.56817	0.15799
O	0.51270	-0.09142	0.11072
C	0.56486	1.22676	0.70584
C	-0.85299	1.48798	1.12965
O	-1.06611	-1.66092	-0.27895
C	1.10445	2.25947	-0.29103
C	2.63891	2.24792	-0.49783
C	3.02342	3.41080	-1.43497
O	3.19276	2.48008	0.80360

C	3.11256	0.95005	-1.11512
C	3.83102	-0.03009	-0.54456
C	4.35000	-0.25357	0.79621
C	4.99839	-1.43779	0.82182
C	4.91469	-2.00936	-0.53954
O	4.19800	-1.12561	-1.32726
O	5.35736	-3.04564	-0.98316
C	5.69558	-2.14598	1.93517
O	-5.85801	2.12913	0.32181
H	-7.30496	0.73596	0.26374
H	-3.83056	1.28496	-0.73516
H	-3.36836	-0.38003	-1.07805
H	-7.33992	-2.50939	0.65608
H	-7.49057	-2.45025	-1.09282
H	-7.94563	-1.04140	-0.12731
H	-3.79374	-2.33962	-0.50185
H	-5.01101	-2.92253	-1.65296
H	-5.09758	-3.41725	0.02970
H	-3.51956	0.96209	1.67618
H	-3.24577	-0.76952	1.45316
H	1.23627	1.16663	1.57044
H	-1.15268	2.40711	1.62174
H	0.59469	2.12475	-1.25079
H	0.84452	3.25661	0.08384
H	2.56761	3.29479	-2.42370
H	4.11096	3.44843	-1.56965
H	2.69125	4.36044	-1.00327
H	4.14069	2.66376	0.69346
H	2.84161	0.80476	-2.15829
H	4.19533	0.43295	1.61577
H	5.64976	-1.56967	2.86295
H	6.74867	-2.32287	1.68481
H	5.24431	-3.12966	2.11230

Conformer 17

Energy: -1227.88964 Hartree (Rel: 1.9 kcal/mol)

XYZ coordinates for conf 17:

C	-6.12160	0.57881	1.16510
C	-5.25001	-0.36539	0.44710
C	-3.76641	-0.15749	0.68000
C	-5.75481	-1.31439	-0.38990
C	-7.22361	-1.57419	-0.63290
C	-4.87021	-2.24569	-1.18410
C	-3.15020	0.88680	-0.28790
C	-1.66040	0.95950	-0.16060
C	-0.79121	-0.14250	-0.65010
O	0.51250	0.17600	-0.39100
C	0.57950	1.46440	0.26420
C	-0.85800	1.88960	0.37240
O	-1.10591	-1.17920	-1.19810
C	1.45070	2.44310	-0.53220
C	2.97960	2.23669	-0.39720
C	3.70340	3.37839	-1.14010
O	3.24760	2.33549	1.00720
C	3.42390	0.91649	-0.98860
C	3.86489	-0.17651	-0.34610
C	4.02029	-0.53041	1.05660
C	4.49729	-1.79111	1.13850
C	4.67239	-2.28111	-0.24580
O	4.27339	-1.27321	-1.10610
O	5.08249	-3.34461	-0.65490
C	4.81899	-2.63941	2.32350
O	-5.70230	1.47851	1.88580
H	-7.21190	0.46511	1.02970

H	-3.22201	-1.09970	0.58780
H	-3.62451	0.20241	1.70350
H	-7.90581	-1.02309	0.01360
H	-7.42911	-2.64469	-0.50270
H	-7.47591	-1.33769	-1.67520
H	-3.82181	-1.94680	-1.22060
H	-5.24121	-2.32469	-2.21380
H	-4.92281	-3.25959	-0.76170
H	-3.40130	0.60800	-1.31890
H	-3.59550	1.86740	-0.09360
H	1.02310	1.30330	1.25380
H	-1.15350	2.83060	0.82410
H	1.15850	2.40200	-1.58680
H	1.23480	3.45490	-0.16850
H	3.46350	3.37519	-2.20850
H	4.79000	3.27039	-1.04040
H	3.40710	4.34309	-0.71570
H	4.21150	2.38109	1.12240
H	3.39060	0.85739	-2.07400
H	3.75889	0.13099	1.86960
H	4.63589	-2.10261	3.25810
H	5.86869	-2.95631	2.30120
H	4.21369	-3.55391	2.32180

Conformer 18

Energy: -1227.88766 Hartree (Rel: 2.0 kcal/mol)

XYZ coordinates for conf 18:

C	3.22760	1.69223	0.43645
C	4.18626	0.70699	-0.07536
C	4.02402	0.32468	-1.53836
C	5.13848	0.17076	0.74360
C	5.33041	0.48985	2.20689
C	6.11887	-0.86491	0.25411
C	3.35094	-1.05294	-1.77747
C	2.00794	-1.17281	-1.11648
C	1.77629	-2.07001	0.04130
O	0.48238	-1.91467	0.46422
C	-0.16617	-0.88943	-0.32670
C	0.86523	-0.51351	-1.34949
O	2.54008	-2.84467	0.57966
C	-0.57828	0.23833	0.63694
C	-1.29497	1.45955	0.00321
C	-1.61508	2.49450	1.10347
O	-0.45792	2.07902	-0.98020
C	-2.54266	1.07262	-0.77068
C	-3.65291	0.47028	-0.31452
C	-4.06701	-0.02620	0.98859
C	-5.30608	-0.55298	0.88544
C	-5.72428	-0.40083	-0.52638
O	-4.69440	0.22190	-1.20925
O	-6.75159	-0.72658	-1.07724
C	-6.18660	-1.18934	1.90789
O	2.31604	2.18381	-0.22986
H	3.34175	2.00947	1.48542
H	4.99548	0.32602	-2.04544
H	3.41612	1.09665	-2.01799
H	5.34975	-0.44540	2.78088
H	4.57552	1.13778	2.64999
H	6.31155	0.96100	2.35230
H	7.10522	-0.70348	0.70424
H	6.23027	-0.88415	-0.83133
H	5.78432	-1.86141	0.57496
H	3.25229	-1.20200	-2.85969
H	3.99085	-1.85712	-1.40054

H	-1.05473	-1.34176	-0.78271
H	0.67250	0.23311	-2.10746
H	-1.21679	-0.20457	1.40851
H	0.32620	0.59733	1.14192
H	-2.28545	2.09236	1.86954
H	-2.08879	3.37137	0.65299
H	-0.68740	2.81414	1.59264
H	0.41884	2.25253	-0.58440
H	-2.52501	1.34072	-1.82327
H	-3.46333	0.02960	1.88470
H	-5.70293	-1.21701	2.88773
H	-6.44248	-2.21449	1.61431
H	-7.13262	-0.64216	1.99937

LIST OF FILES (technical info - delete in the final SI version
abimael-PkC-SR-E-du8ml-chloroform_94.log abimael-PkC-SR-E-du8ml-chloroform_193.log abimael-PkC-SR-E-du8ml-chloroform_2.log abimael-PkC-SR-E-du8ml-chloroform_104.log abimael-PkC-SR-E-du8ml-chloroform_86.log abimael-PkC-SR-E-du8ml-chloroform_133.log abimael-PkC-SR-E-du8ml-chloroform_13.log abimael-PkC-SR-E-du8ml-chloroform_97.log abimael-PkC-SR-E-du8ml-chloroform_14.log abimael-PkC-SR-E-du8ml-chloroform_18.log abimael-PkC-SR-E-du8ml-chloroform_138.log abimael-PkC-SR-E-du8ml-chloroform_19.log abimael-PkC-SR-E-du8ml-chloroform_77.log abimael-PkC-SR-E-du8ml-chloroform_63.log abimael-PkC-SR-E-du8ml-chloroform_68.log abimael-PkC-SR-E-du8ml-chloroform_27.log abimael-PkC-SR-E-du8ml-chloroform_31.log abimael-PkC-SR-E-du8ml-chloroform_4.log

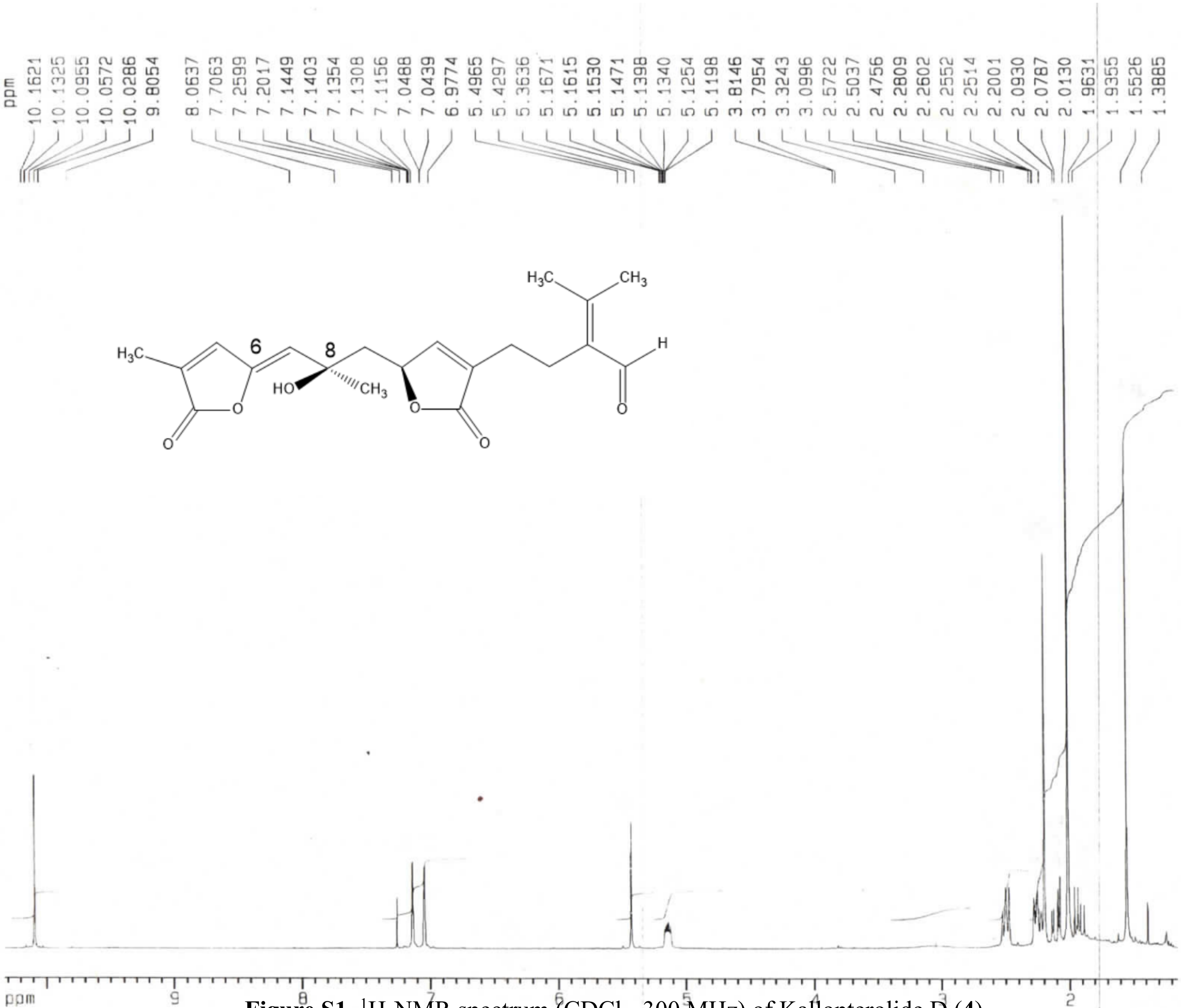

Figure S1. ^{1}H-NMR spectrum ($CDCl_3$, 300 MHz) of Kallopterolide D (**4**)

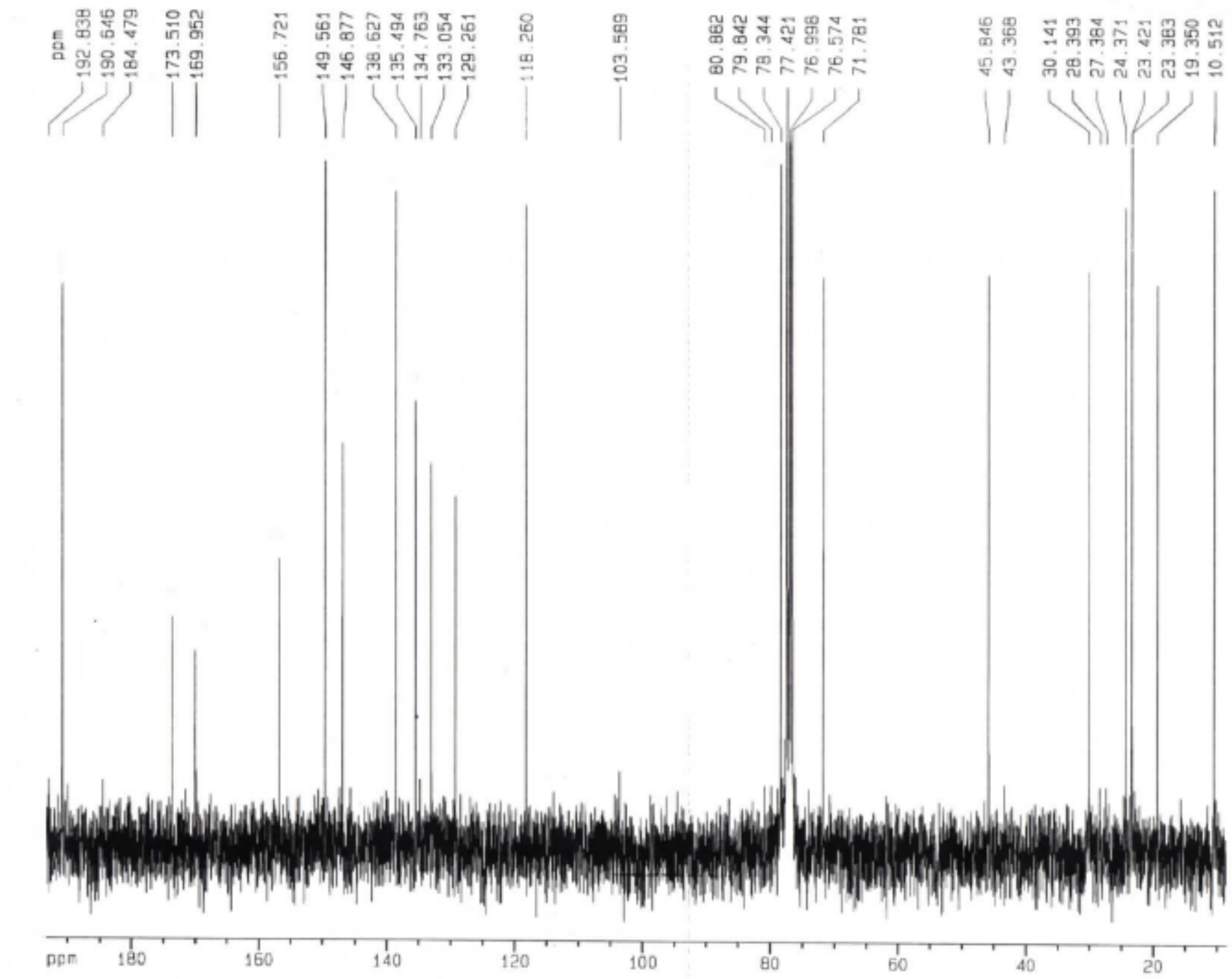

Figure S2. ^{13}C-NMR spectrum ($CDCl_3$, 75 MHz) of Kallopterolide D (**4**)

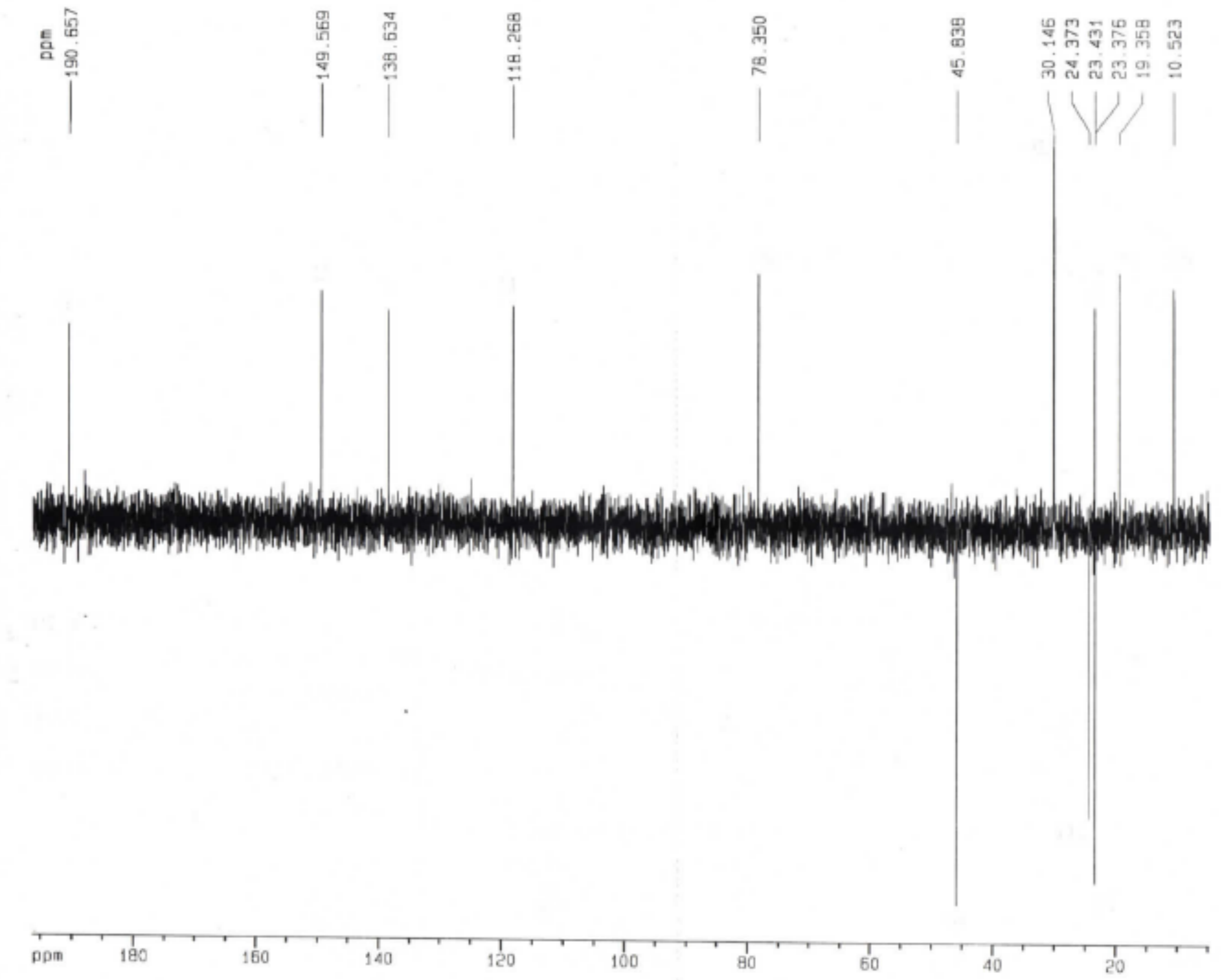

Figure S3. DEPT-135 spectrum ($CDCl_3$, 75 MHz) of Kallopterolide D (**4**)

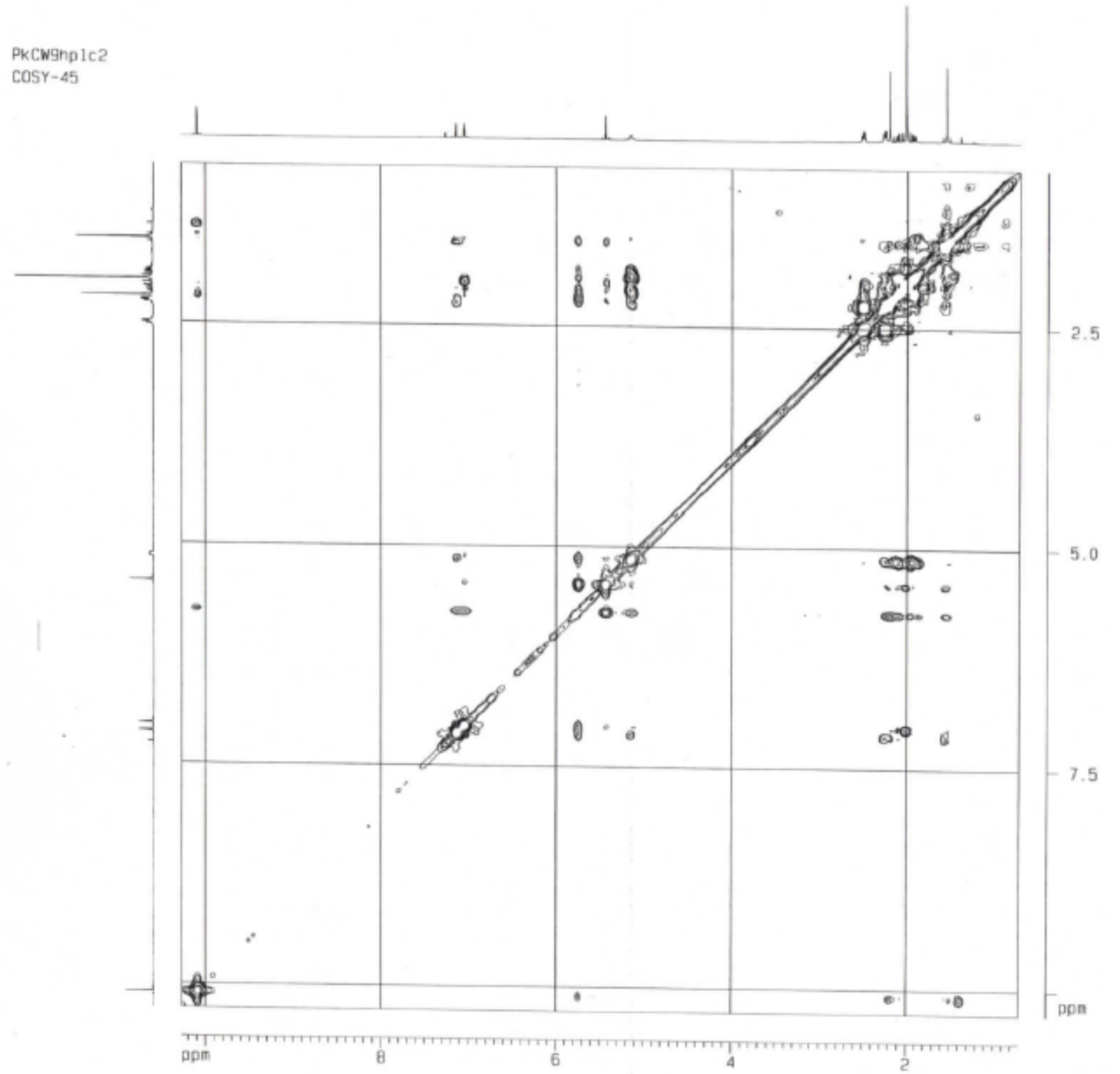

Figure S4. ^{1}H-^{1}H-COSY spectrum ($CDCl_3$) of Kallopterolide D (**4**)

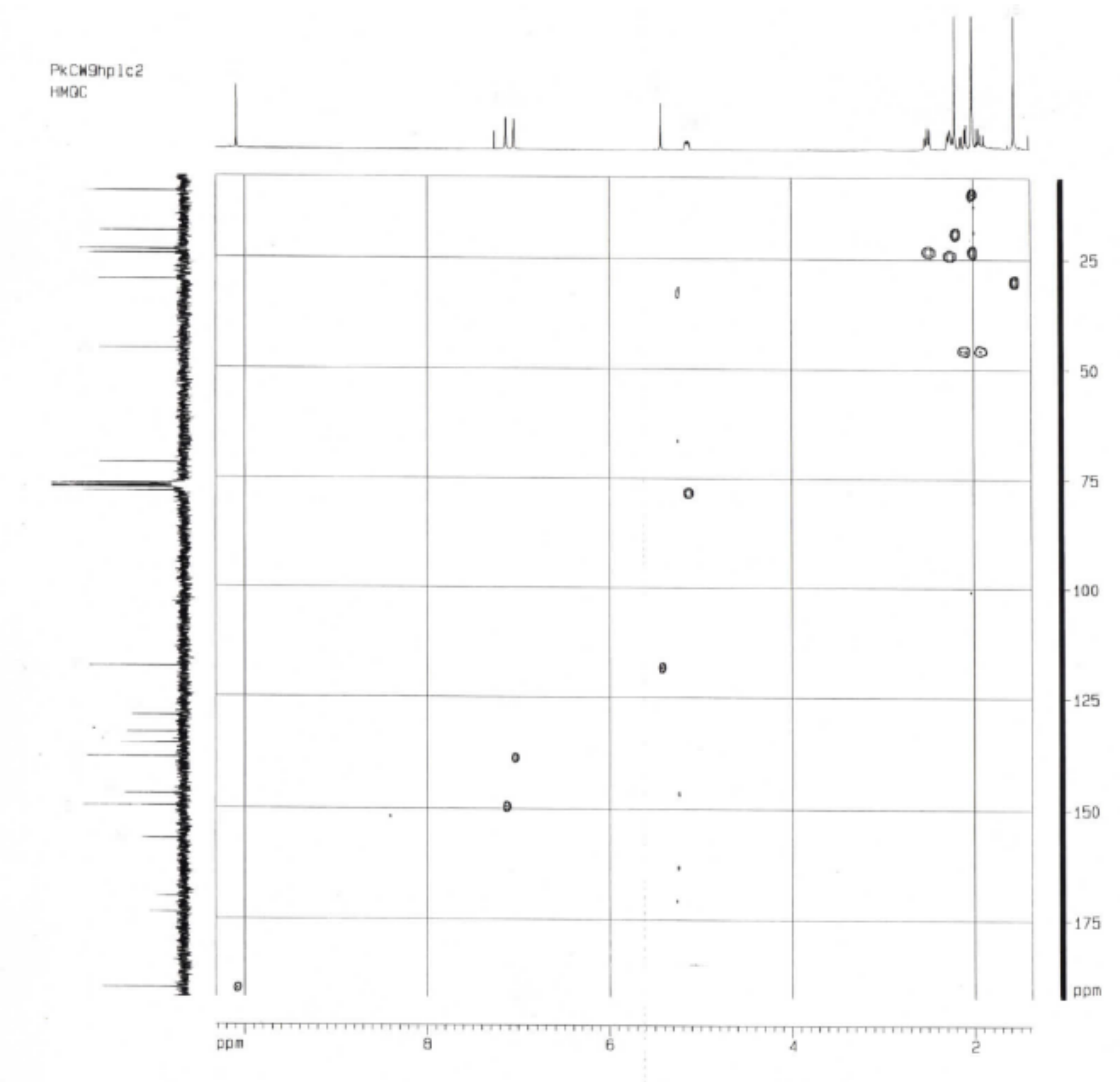

Figure S5. ^{1}H-^{13}C-HMQC spectrum ($CDCl_3$) of Kallopterolide D (**4**)

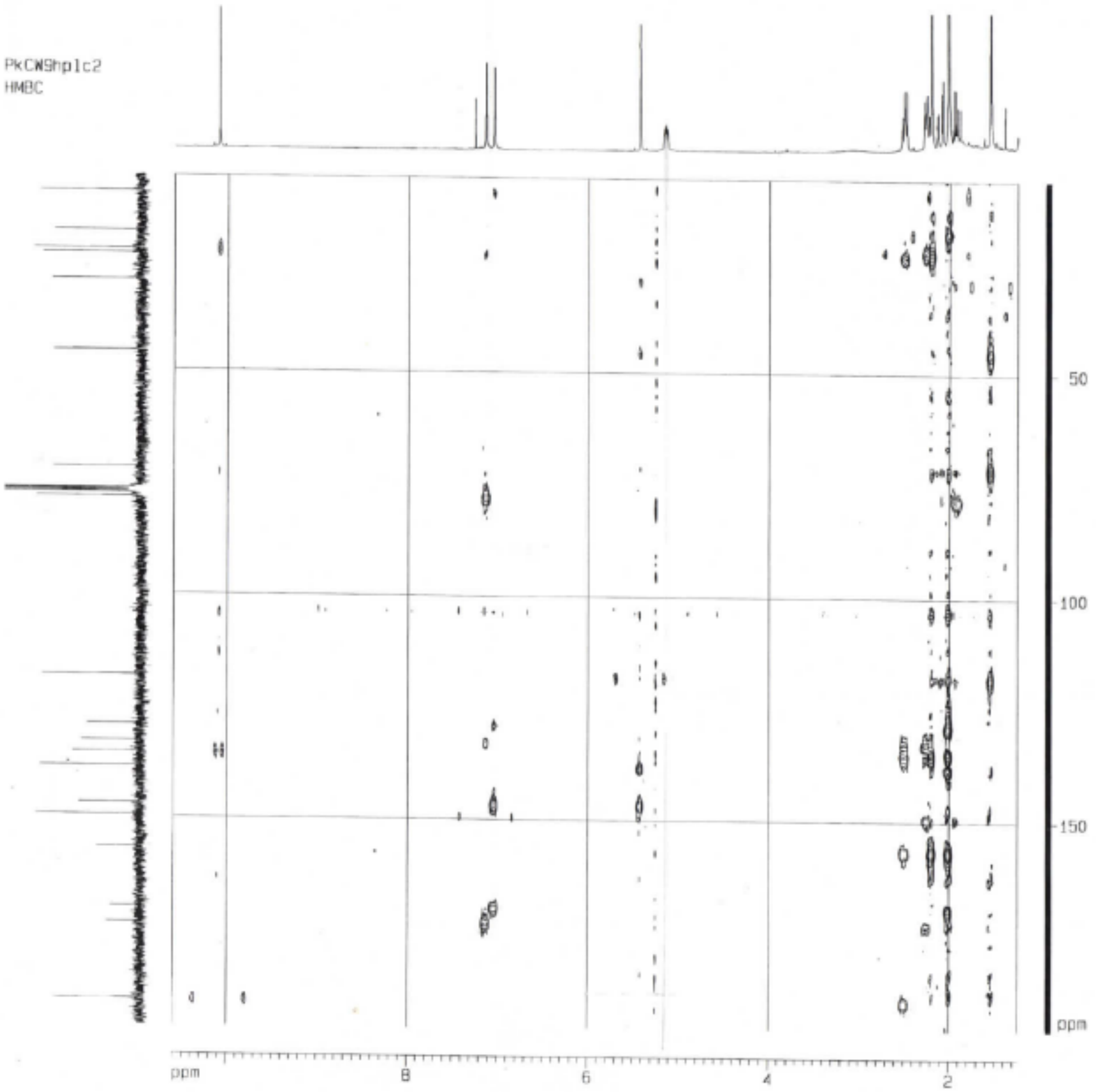

Figure S6. ¹H-¹³C-HMBC spectrum ($CDCl_3$) of Kallopterolide D (**4**)

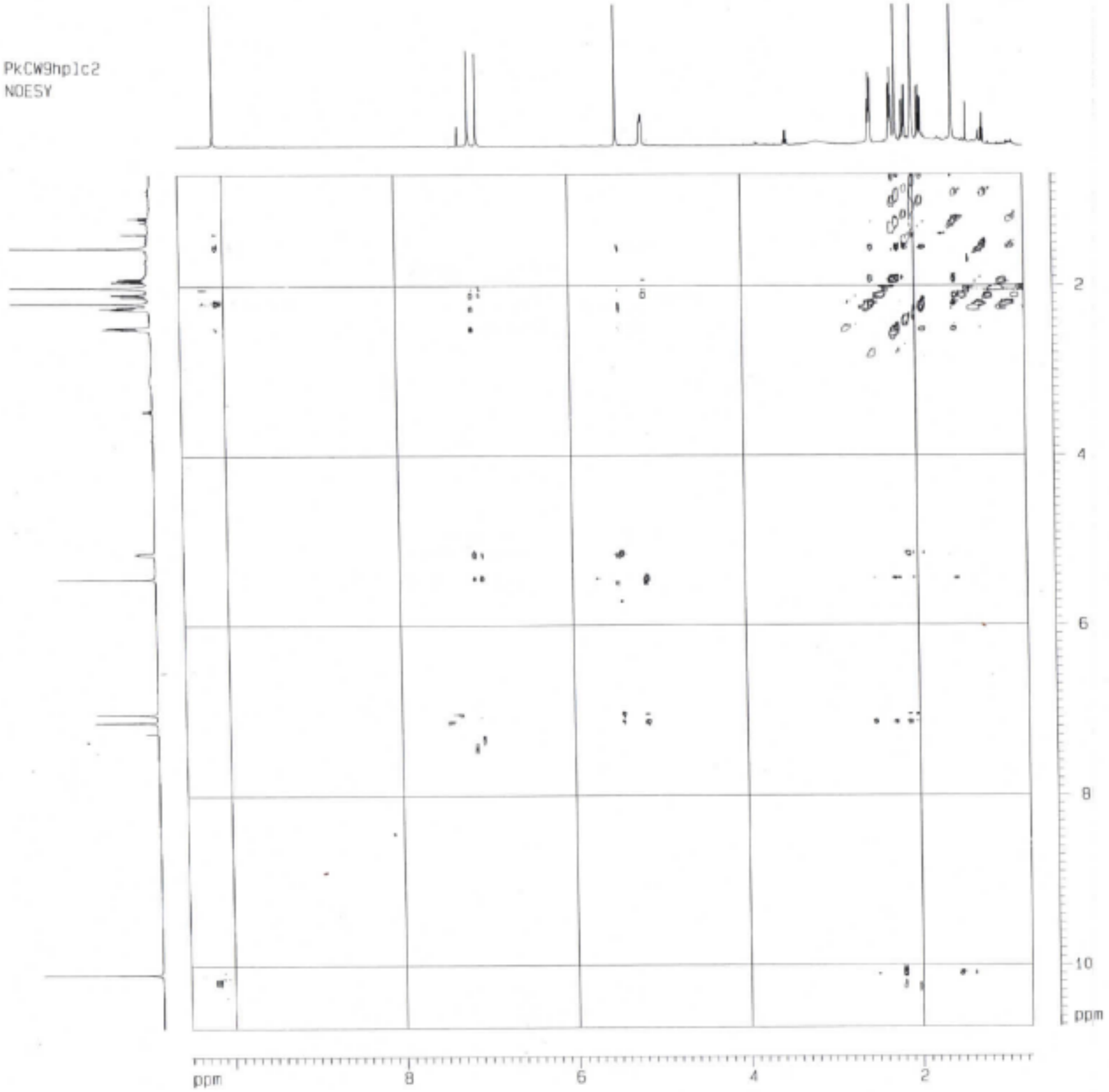

Figure S7. ^{1}H-^{1}H-NOESY spectrum ($CDCl_3$) of Kallopterolide D (**4**)

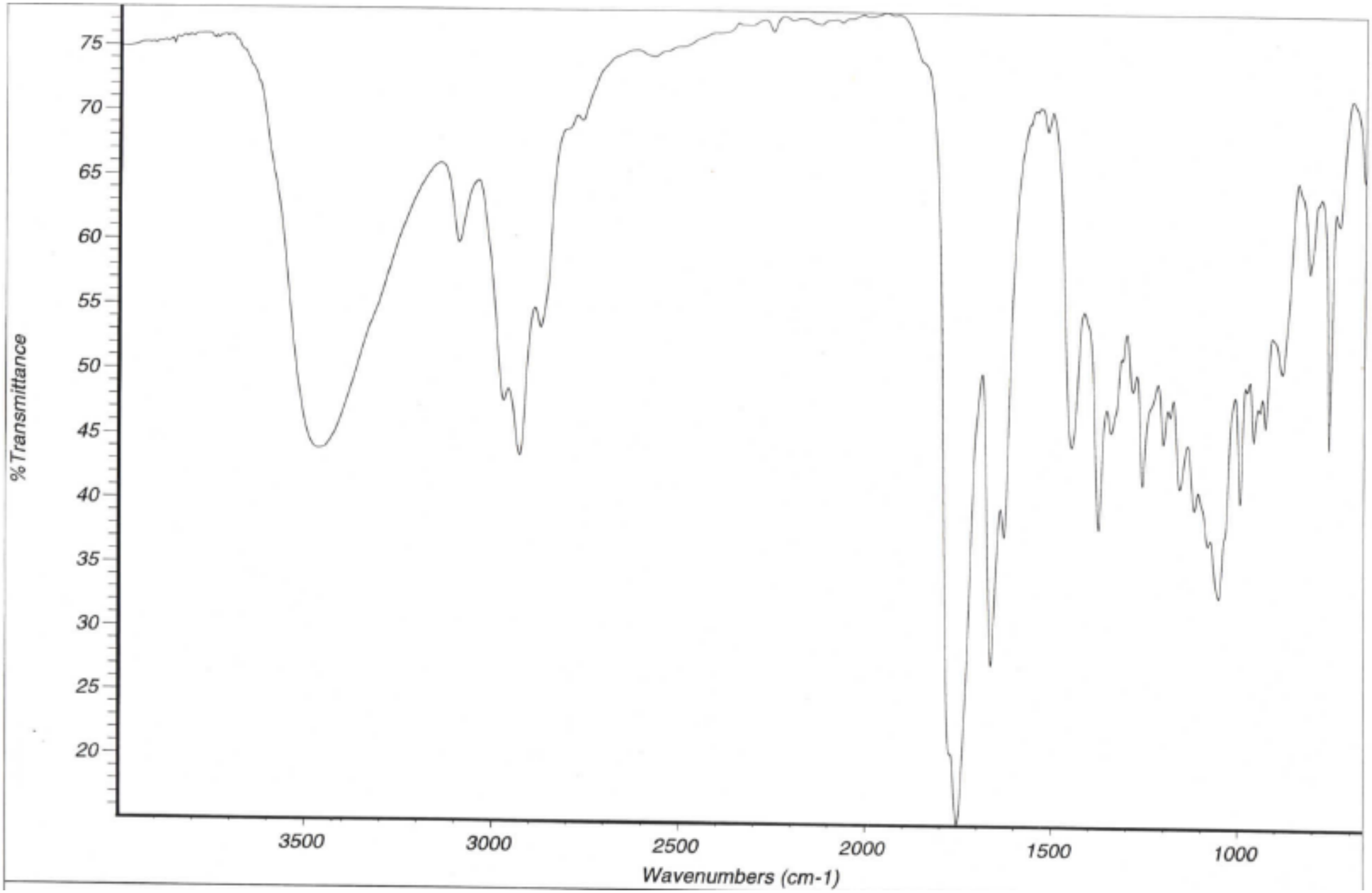

Figure S8. FTIR spectrum of Kallopterolide D (**4**)

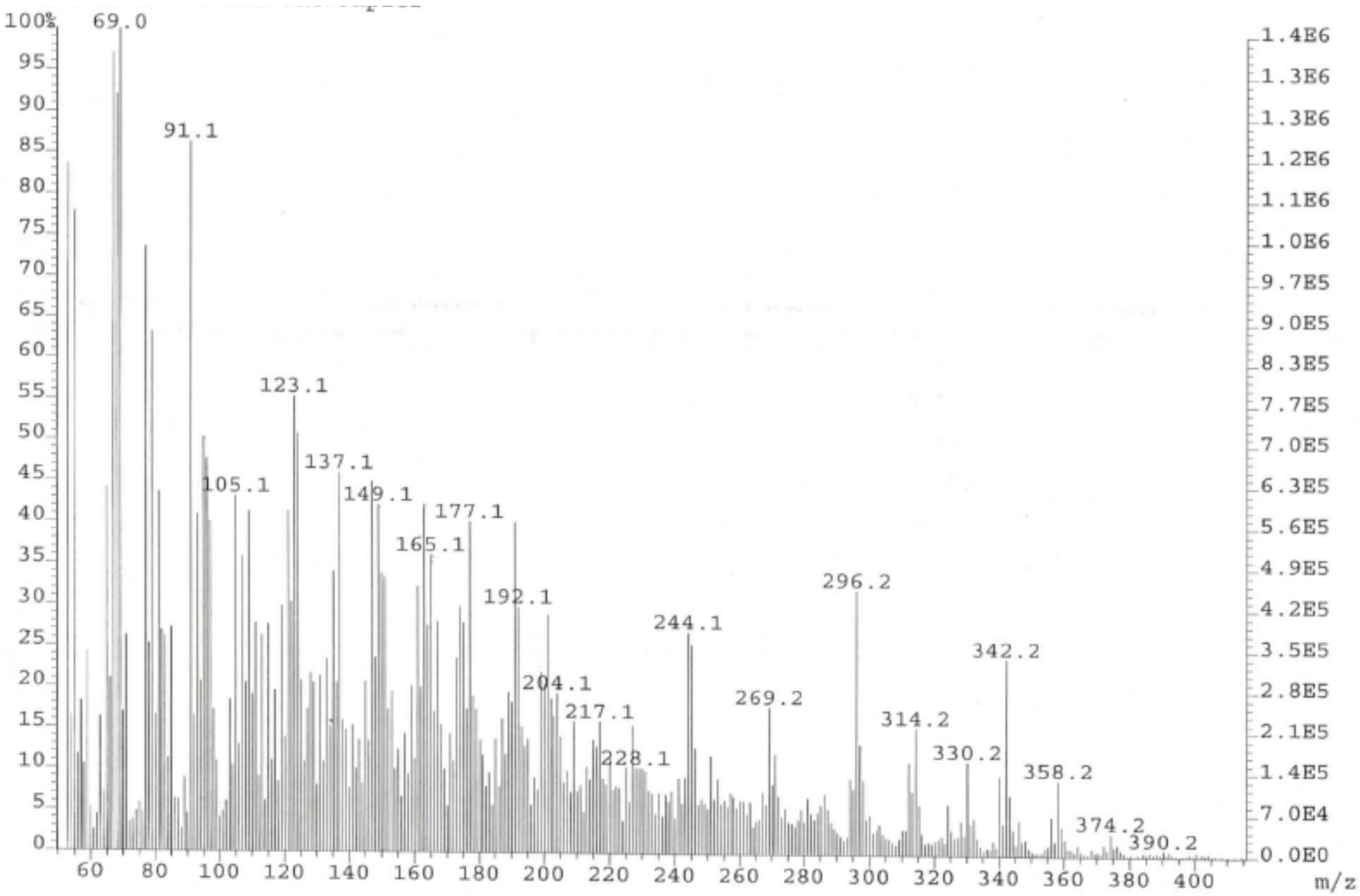

Figure S9. HRFAB-MS (glycerol) spectrum of Kallopterolide D (**4**)

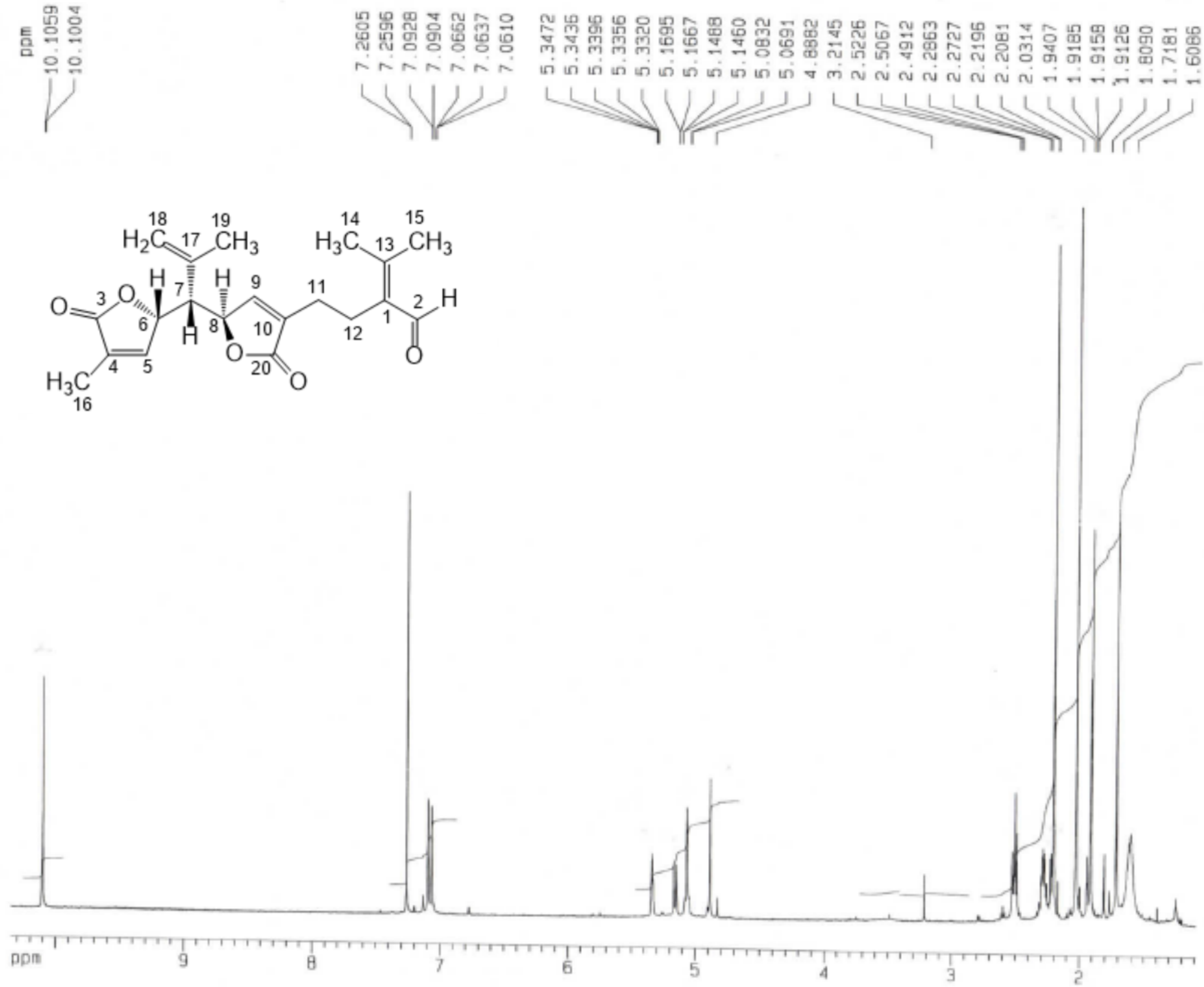

Figure S10. ^{1}H-NMR spectrum ($CDCl_3$, 300 MHz) of Kallopterolide A (**1**)

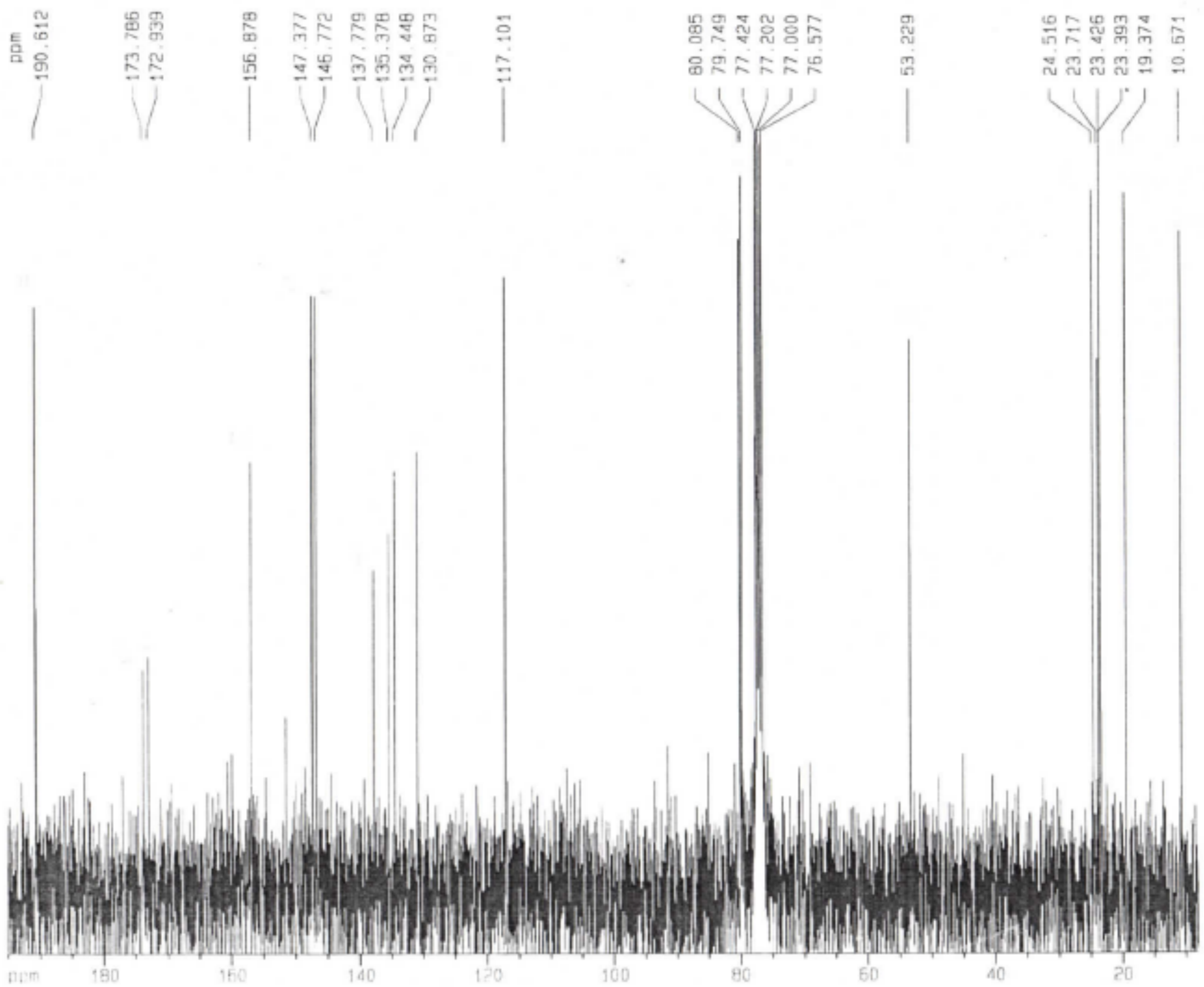

Figure S11. ^{13}C-NMR spectrum ($CDCl_3$, 75 MHz) of Kallopterolide A (**1**)

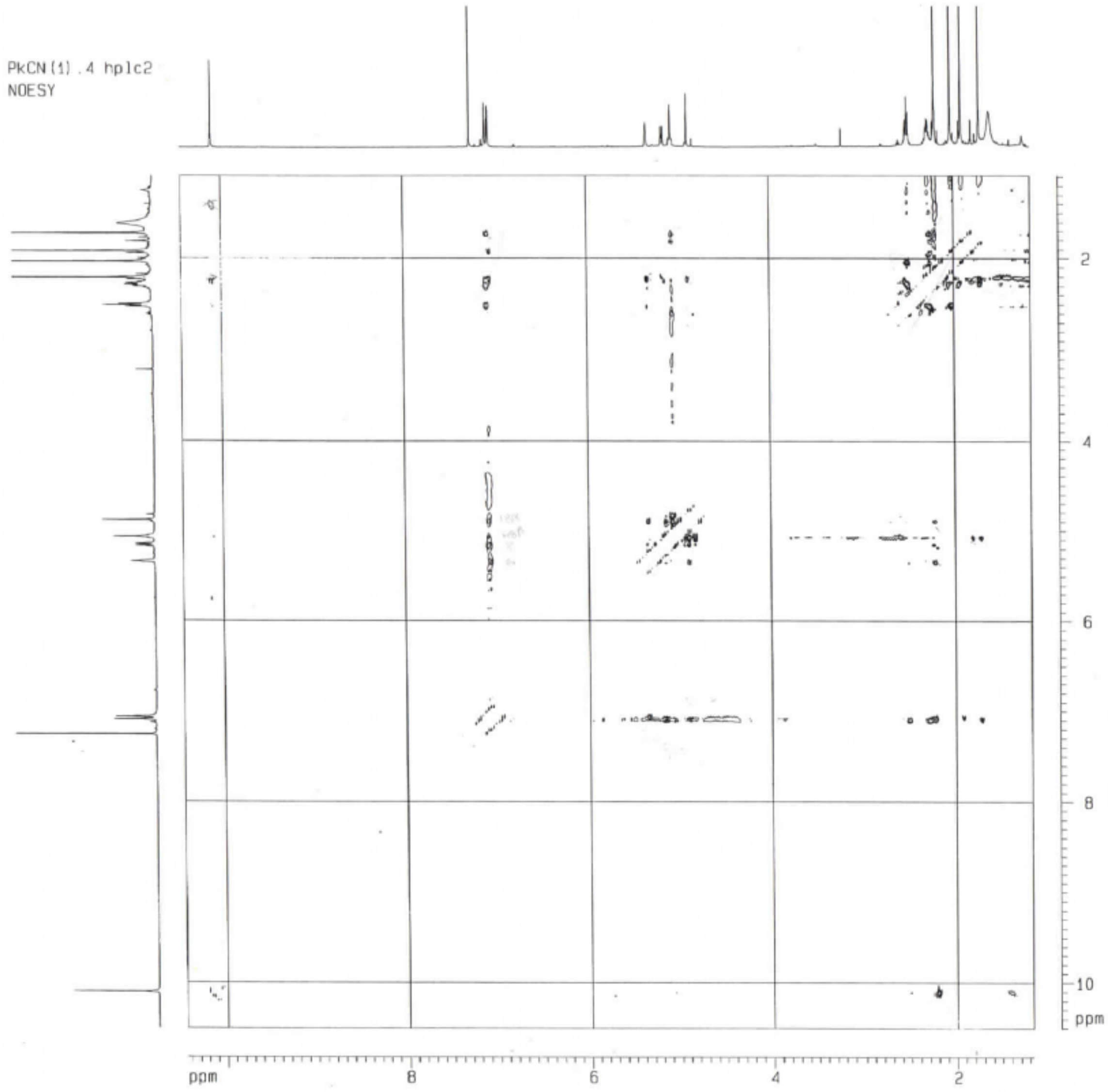

Figure S12. ^{1}H-^{1}H-NOESY spectrum ($CDCl_3$) of Kallopterolide A (**1**)

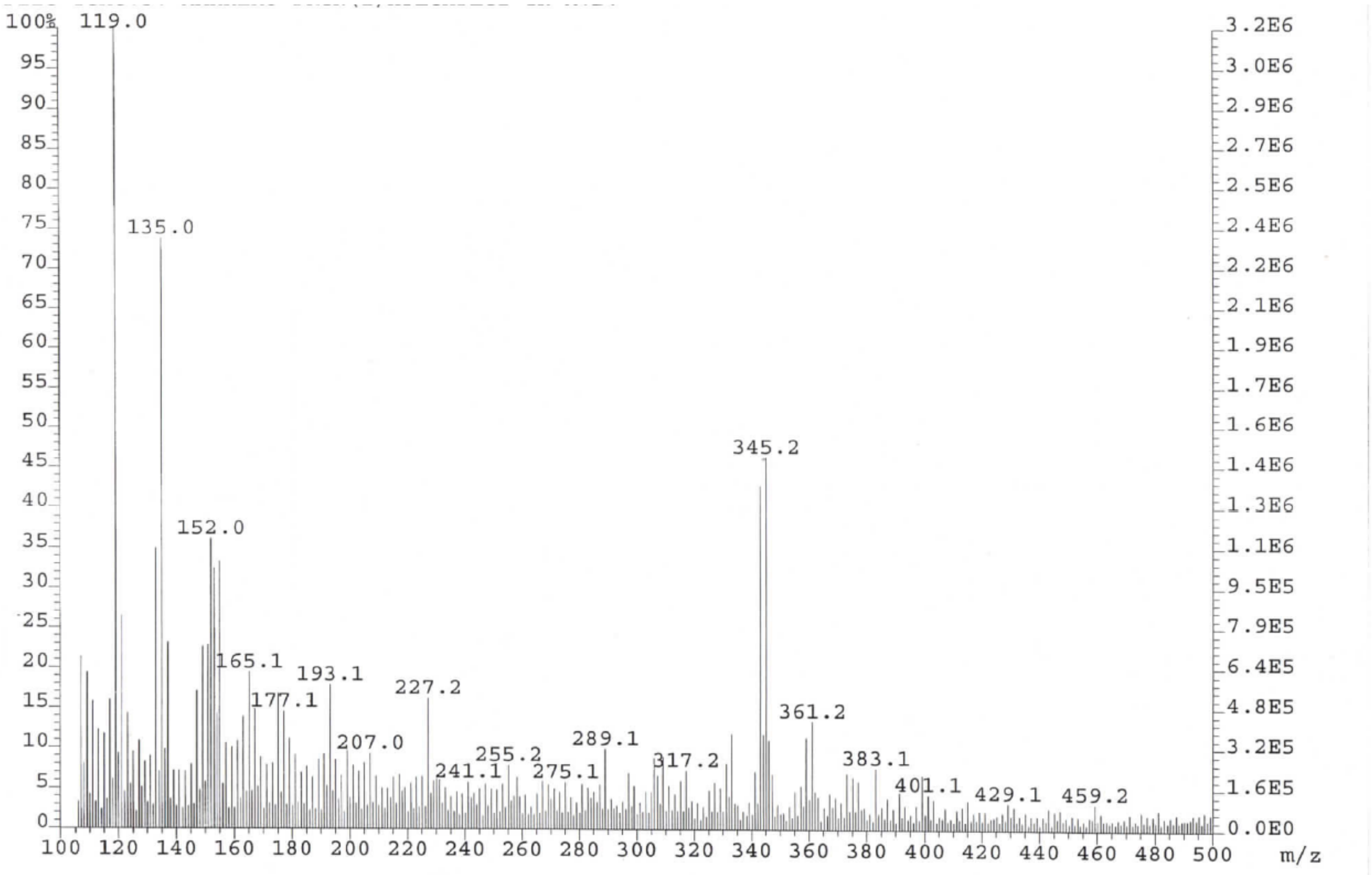

Figure S13. HRFAB-MS (glycerol) spectrum of Kallopterolide A (**1**)|

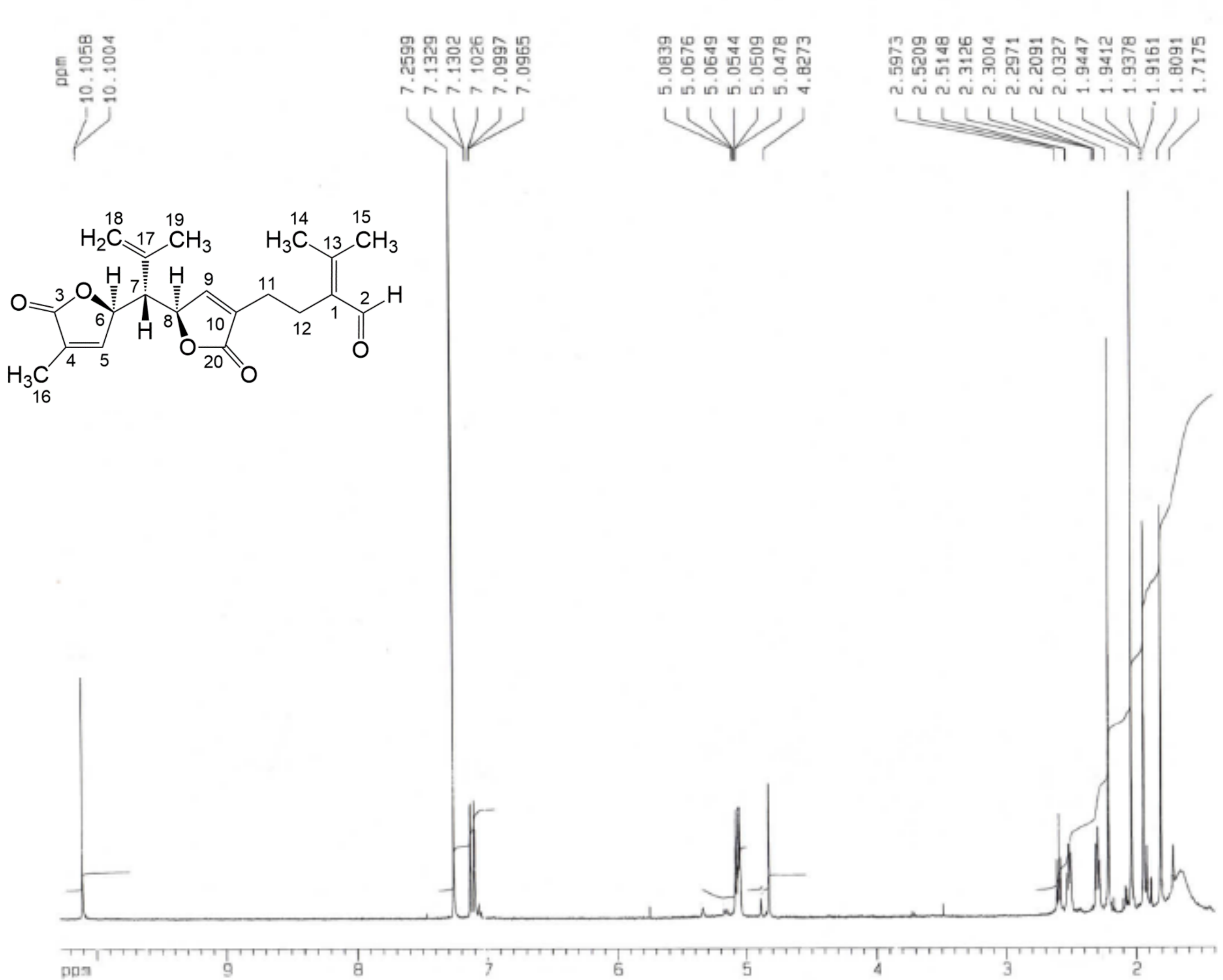

Figure S14. ^{1}H-NMR spectrum ($CDCl_3$, 300 MHz) of Kallopterolide B (**2**)

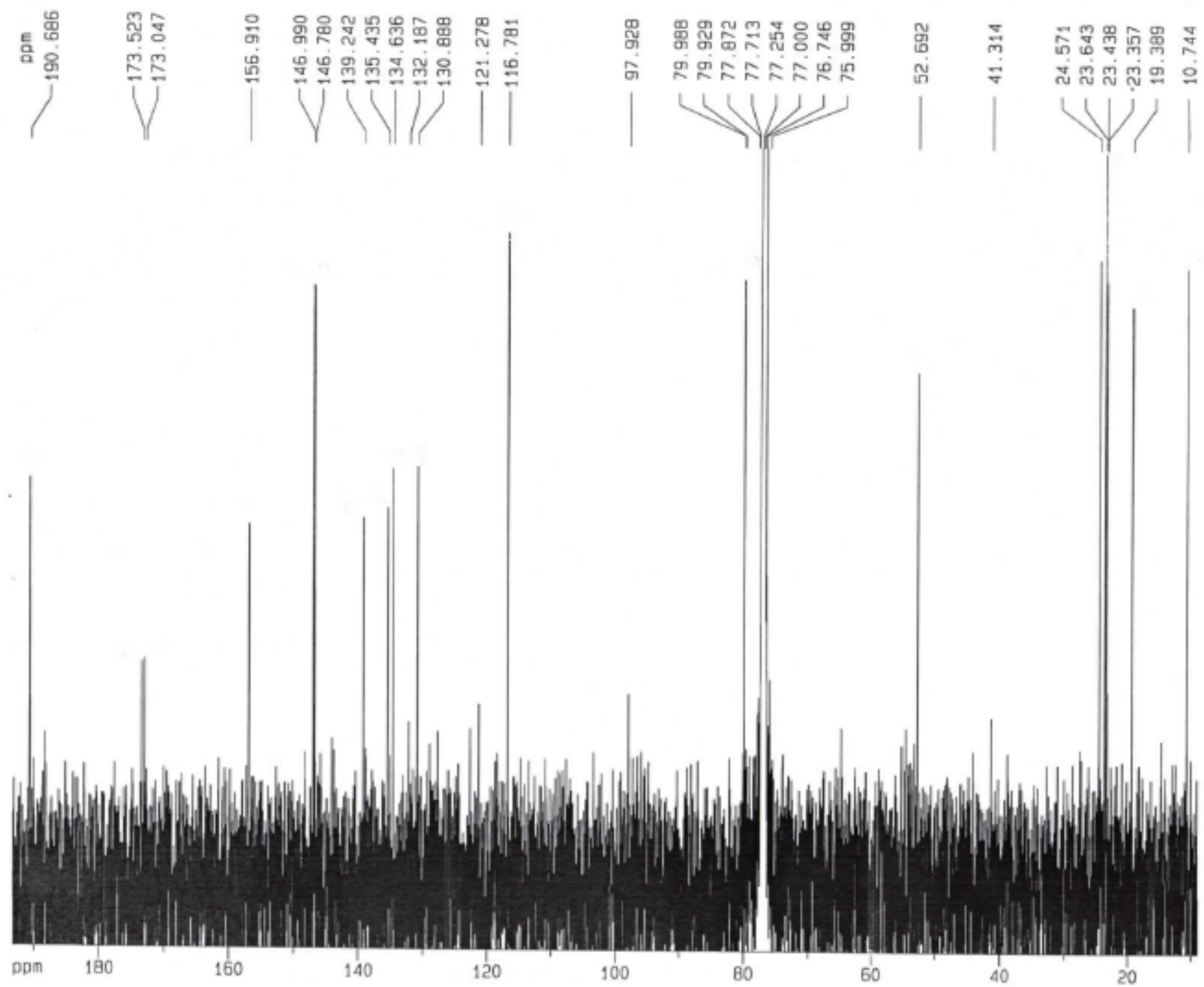

Figure S15. ^{13}C-NMR spectrum ($CDCl_3$, 75 MHz) of Kallopterolide B (**2**)

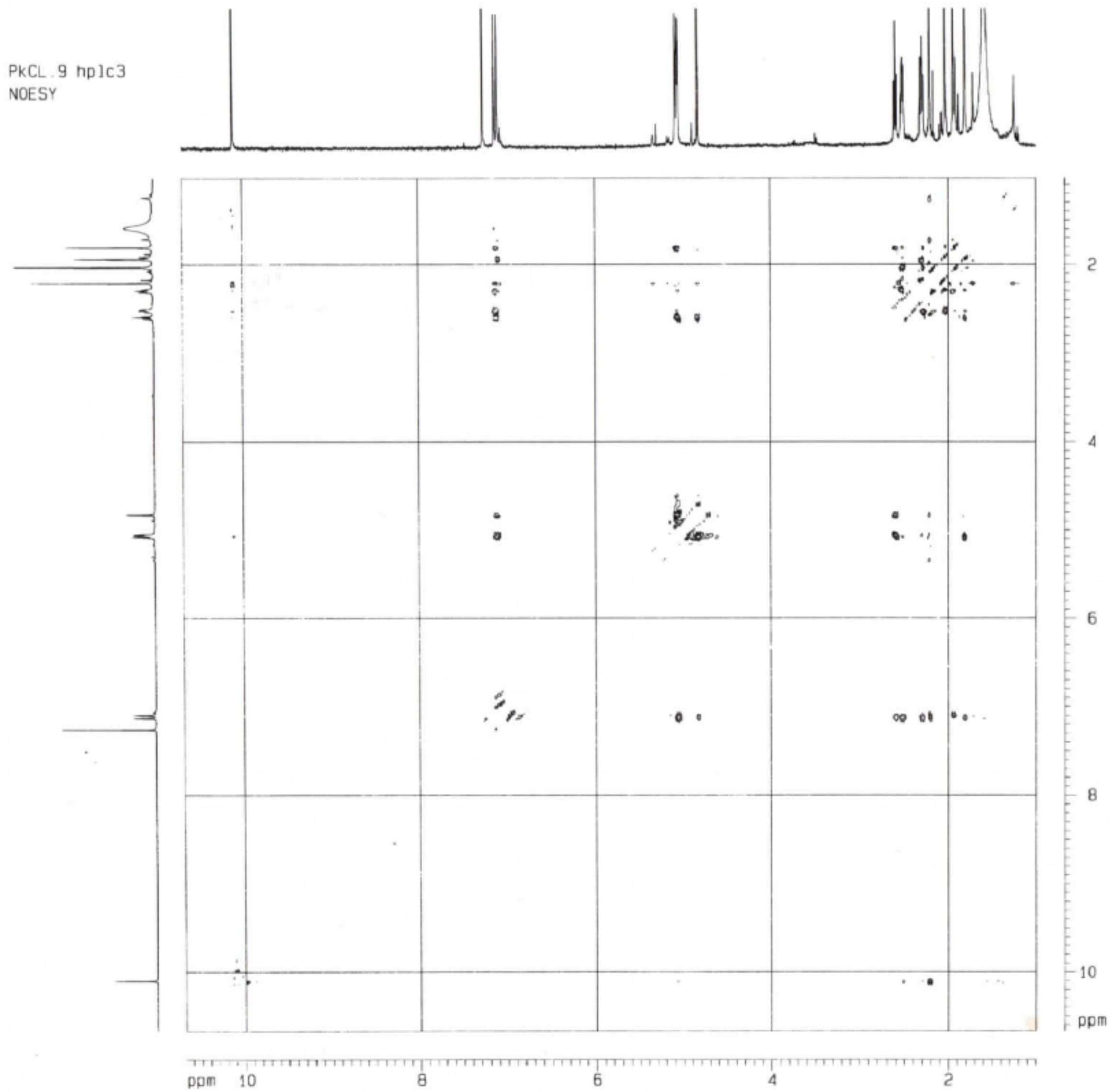

Figure S16. ^{1}H-^{1}H-NOESY spectrum ($CDCl_3$) of Kallopterolide B (**2**)

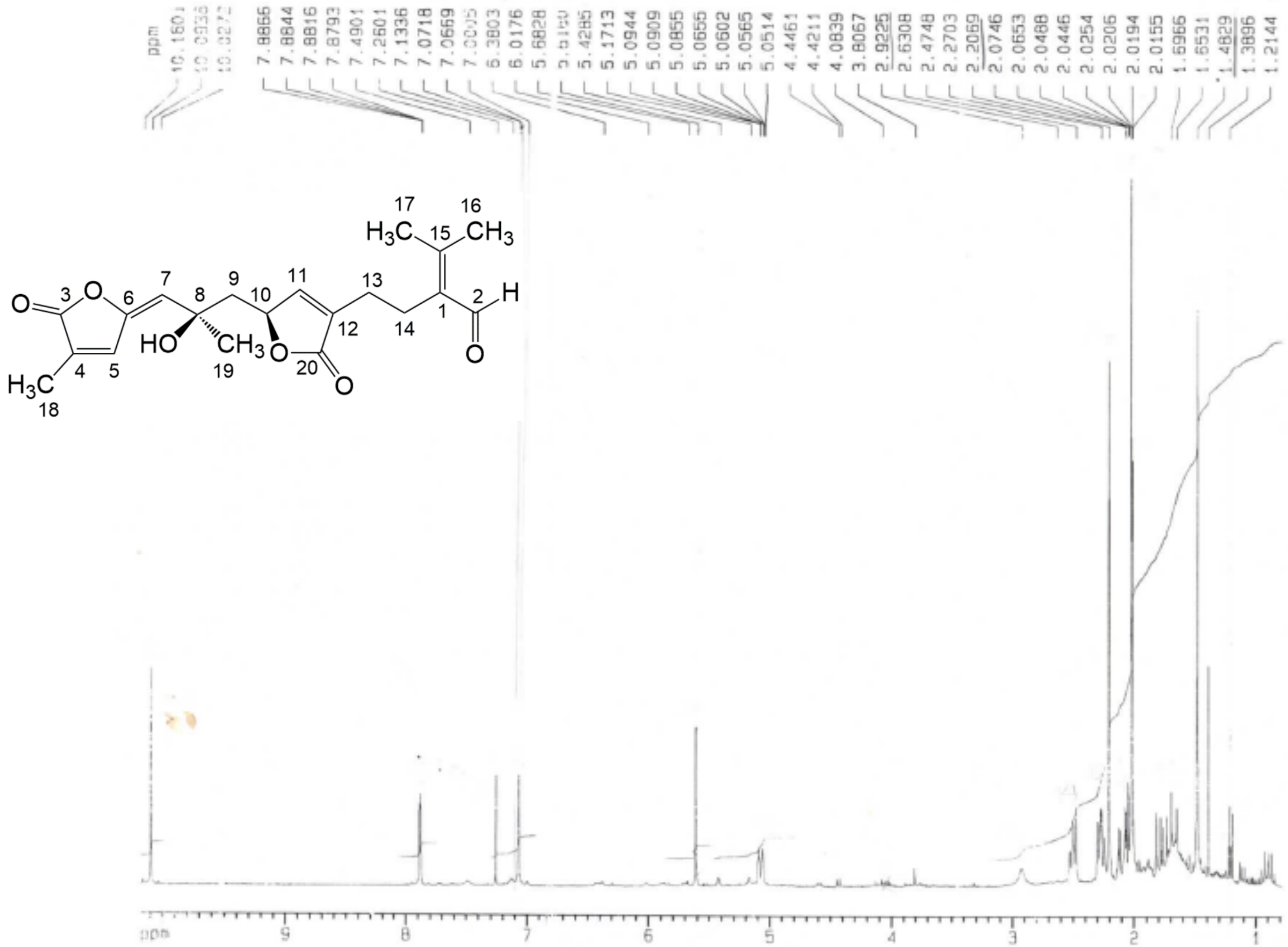

Figure S17. ^{1}H-NMR spectrum ($CDCl_3$, 300 MHz) of Kallopterolide C (**3**)

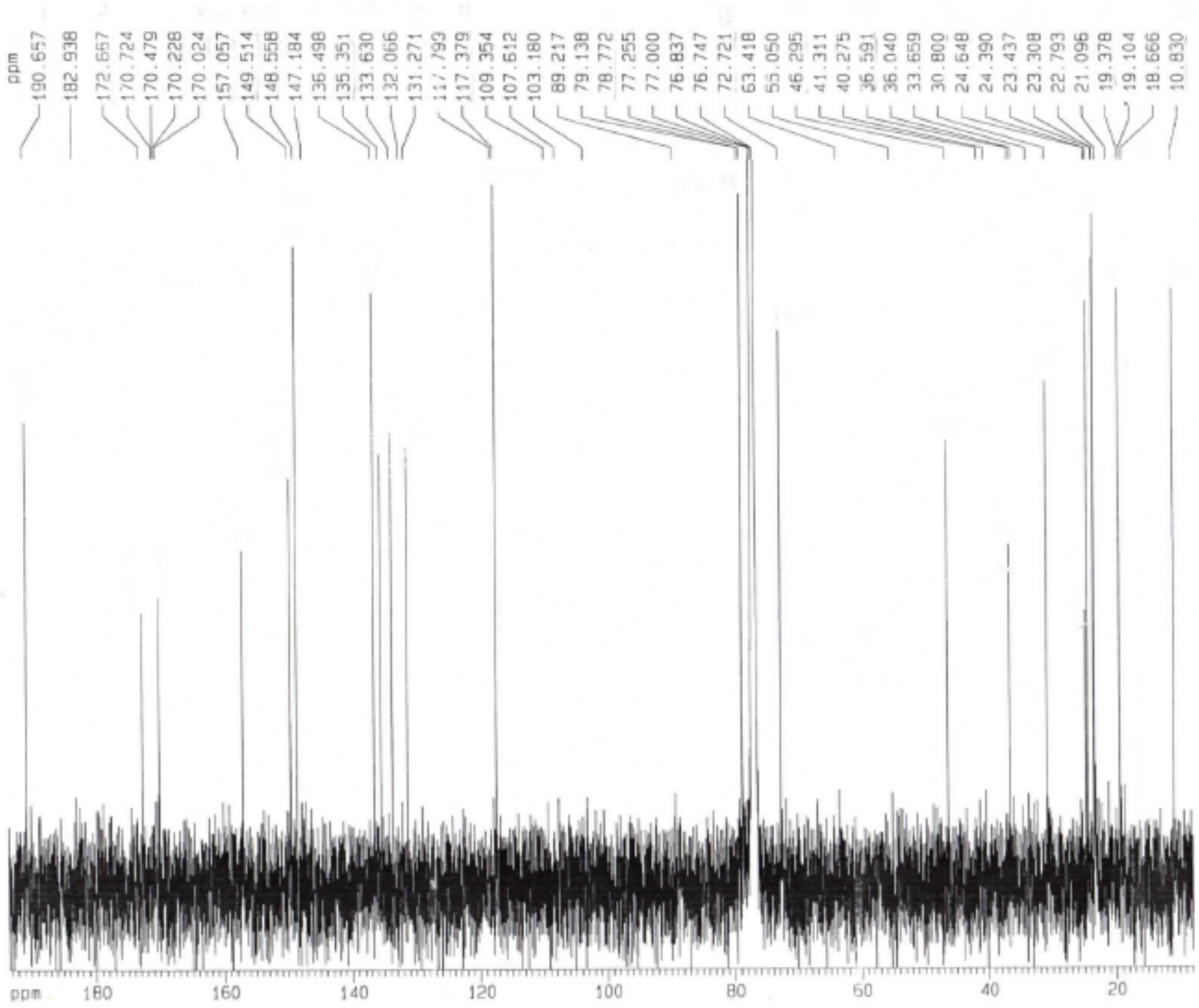

Figure S18. ^{13}C-NMR spectrum ($CDCl_3$, 75 MHz) of Kallopterolide C (**3**)

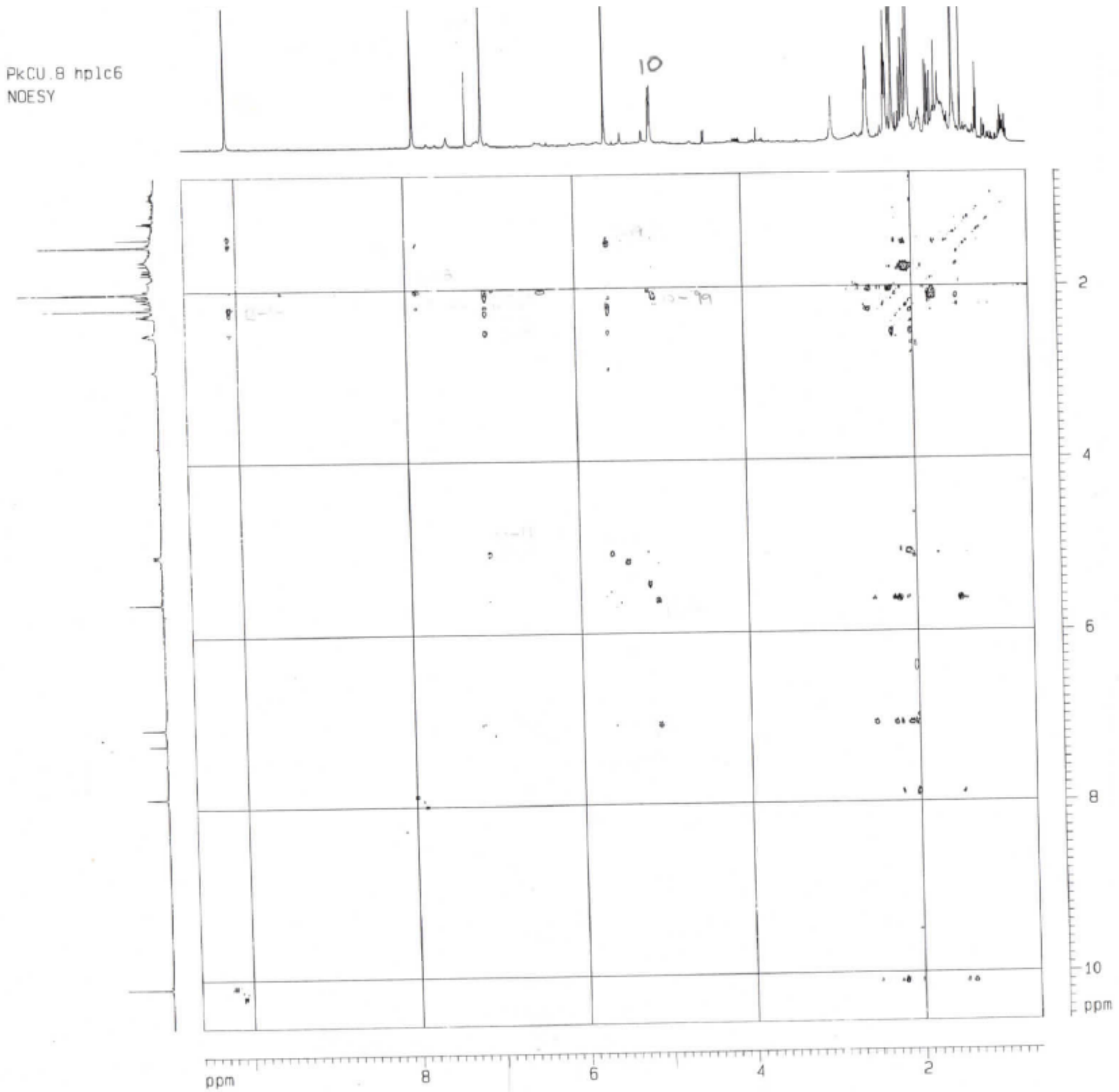

Figure S19. ^1H-^1H-NOESY spectrum ($CDCl_3$) of Kallopterolide C (**3**)

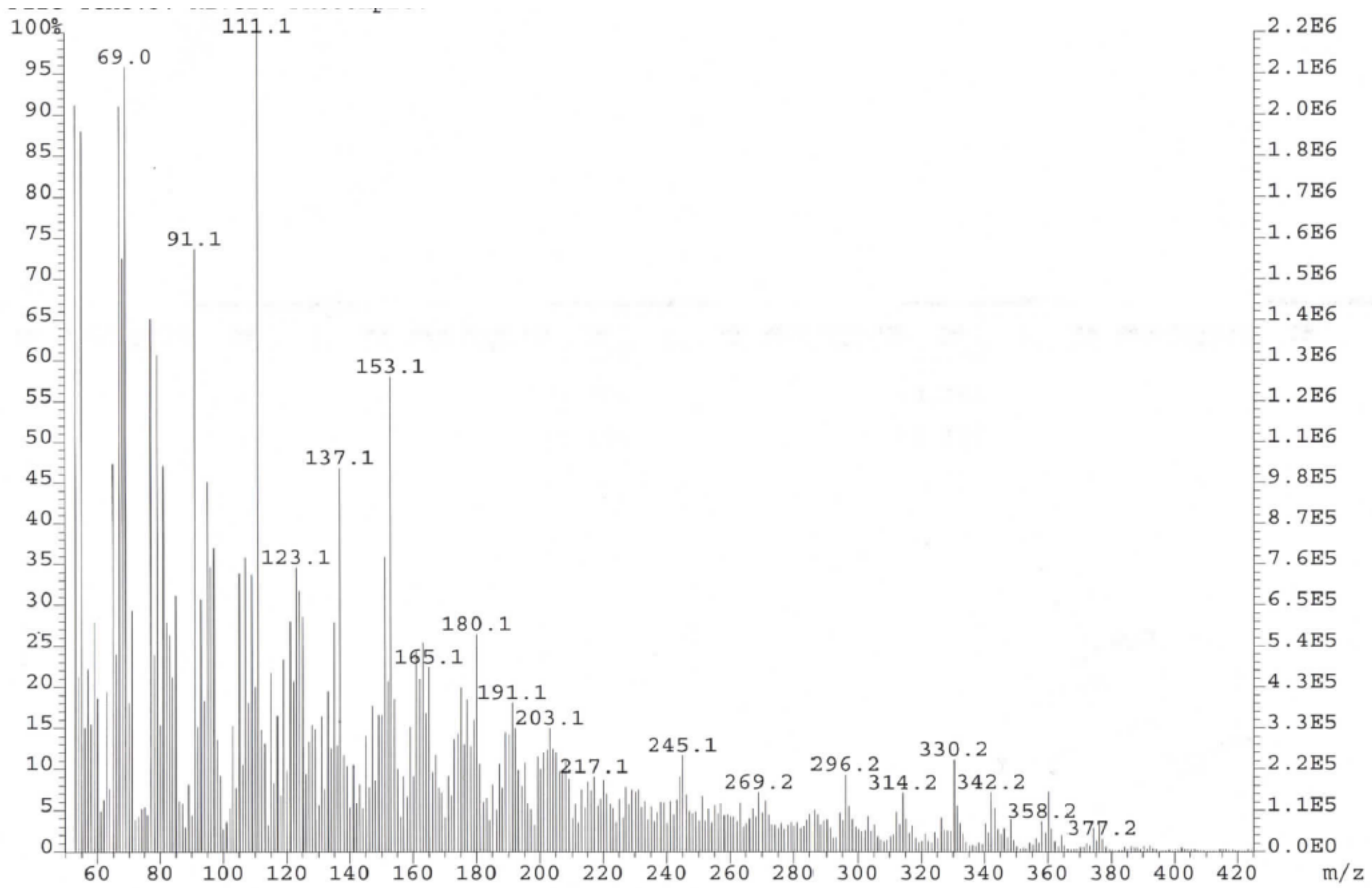

Figure S20. LR-EI-MS spectrum of Kallopterolide C (**3**)|

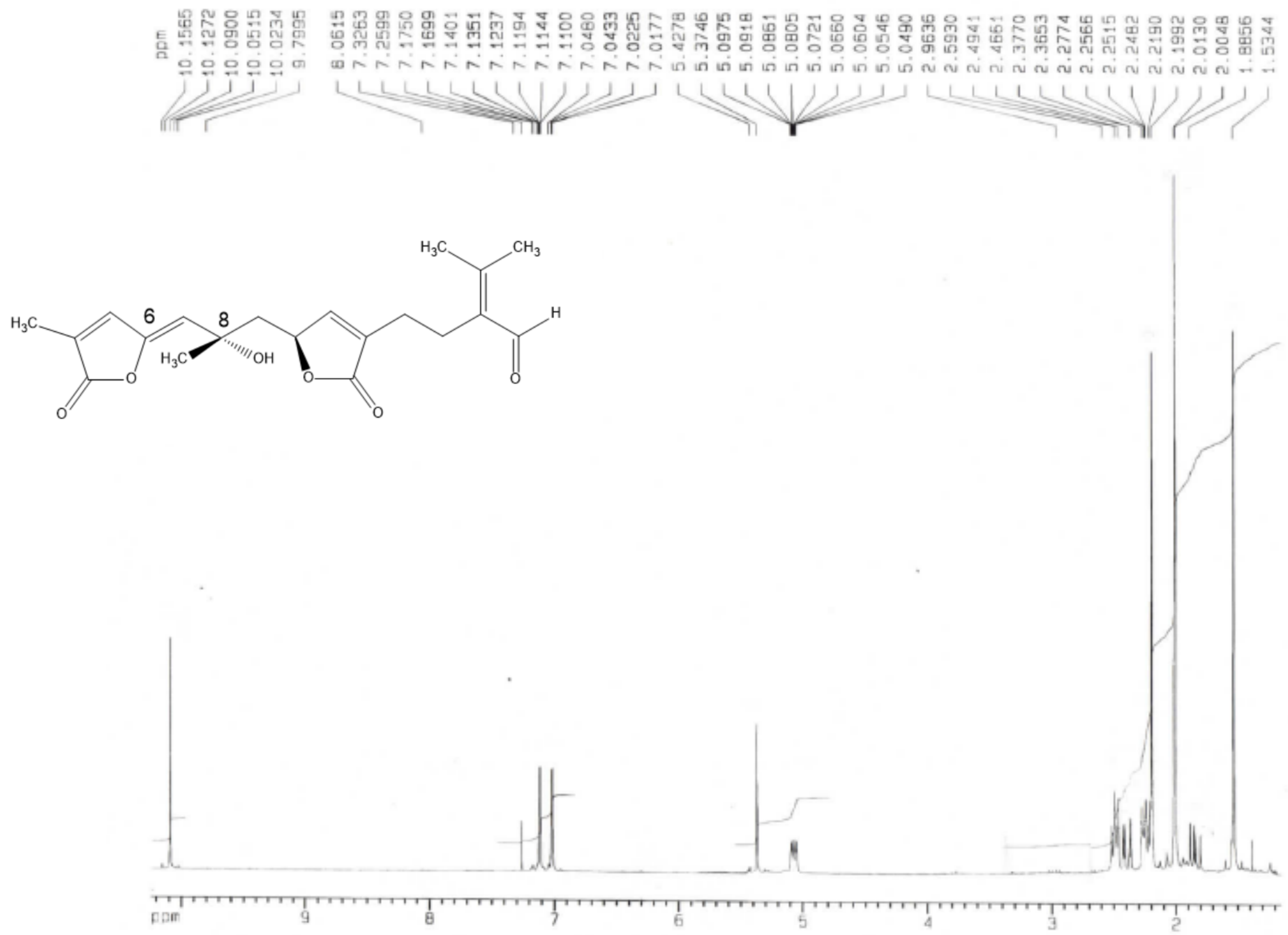

Figure S21. ^{1}H-NMR spectrum ($CDCl_3$, 300 MHz) of Kallopterolide E (**5**)

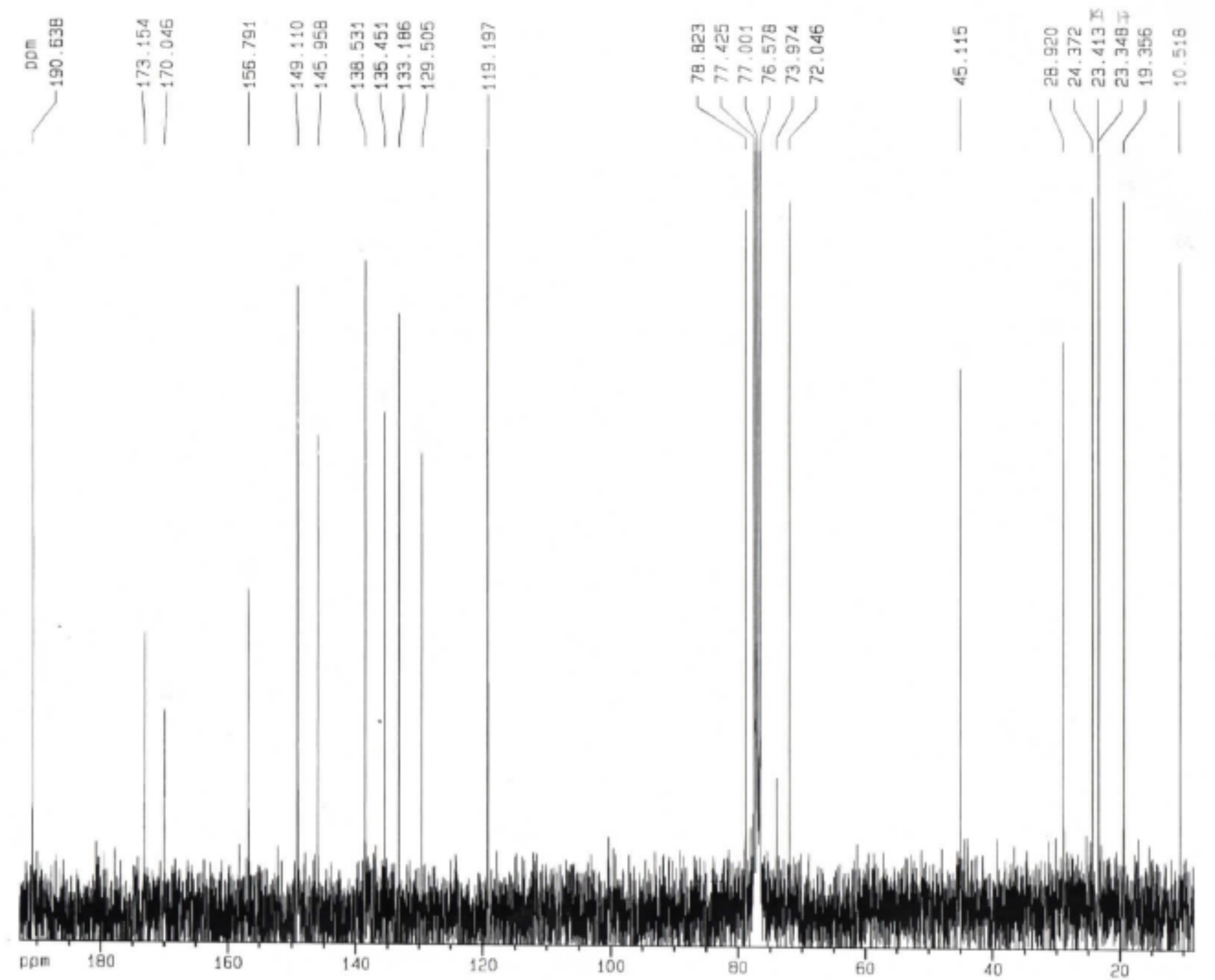

Figure S22. ^{13}C-NMR spectrum ($CDCl_3$, 75 MHz) of Kallopterolide E (**5**)

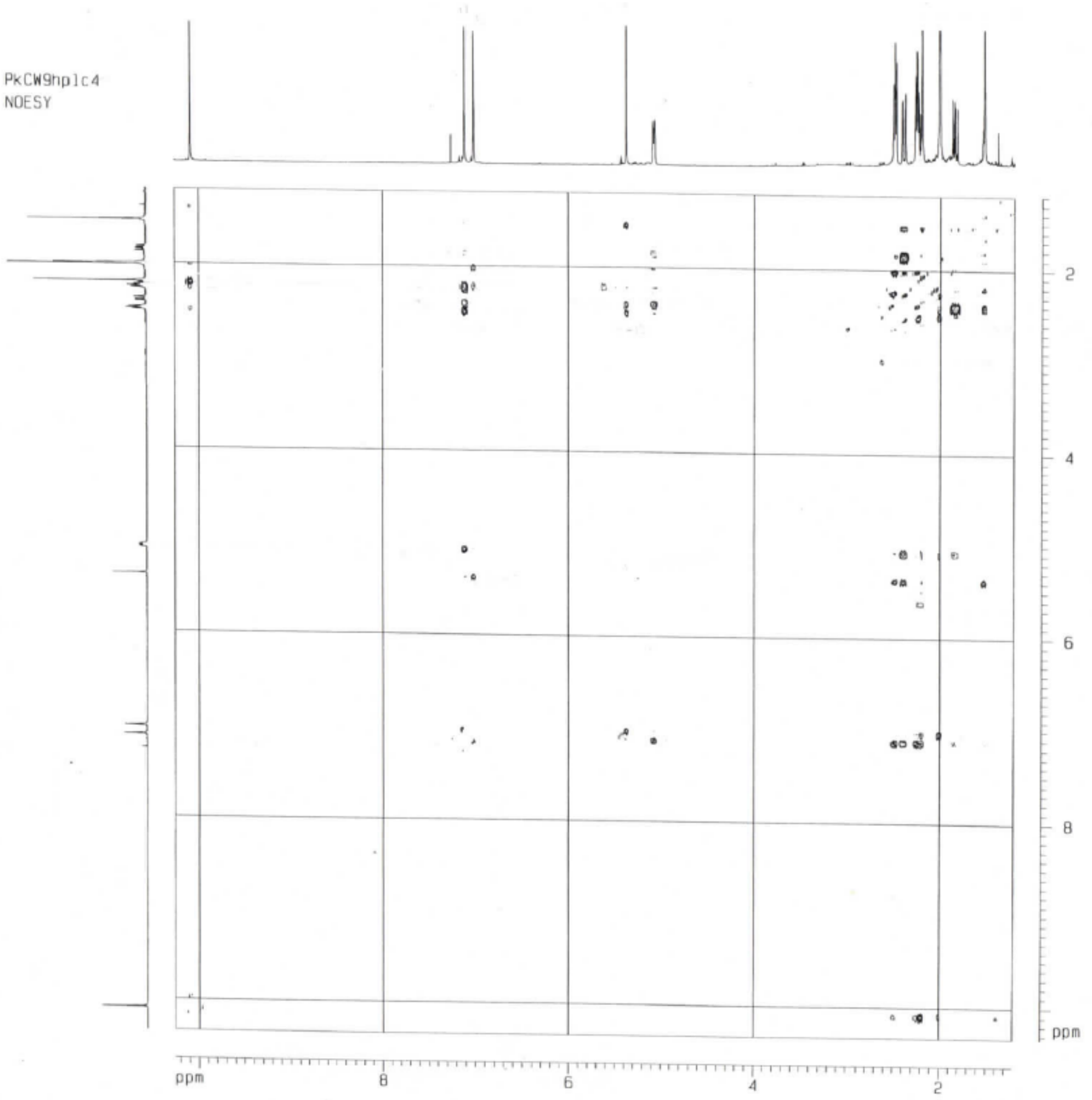

Figure S23. ^{1}H-^{1}H-NOESY spectrum ($CDCl_3$) of Kallopterolide E (**5**)

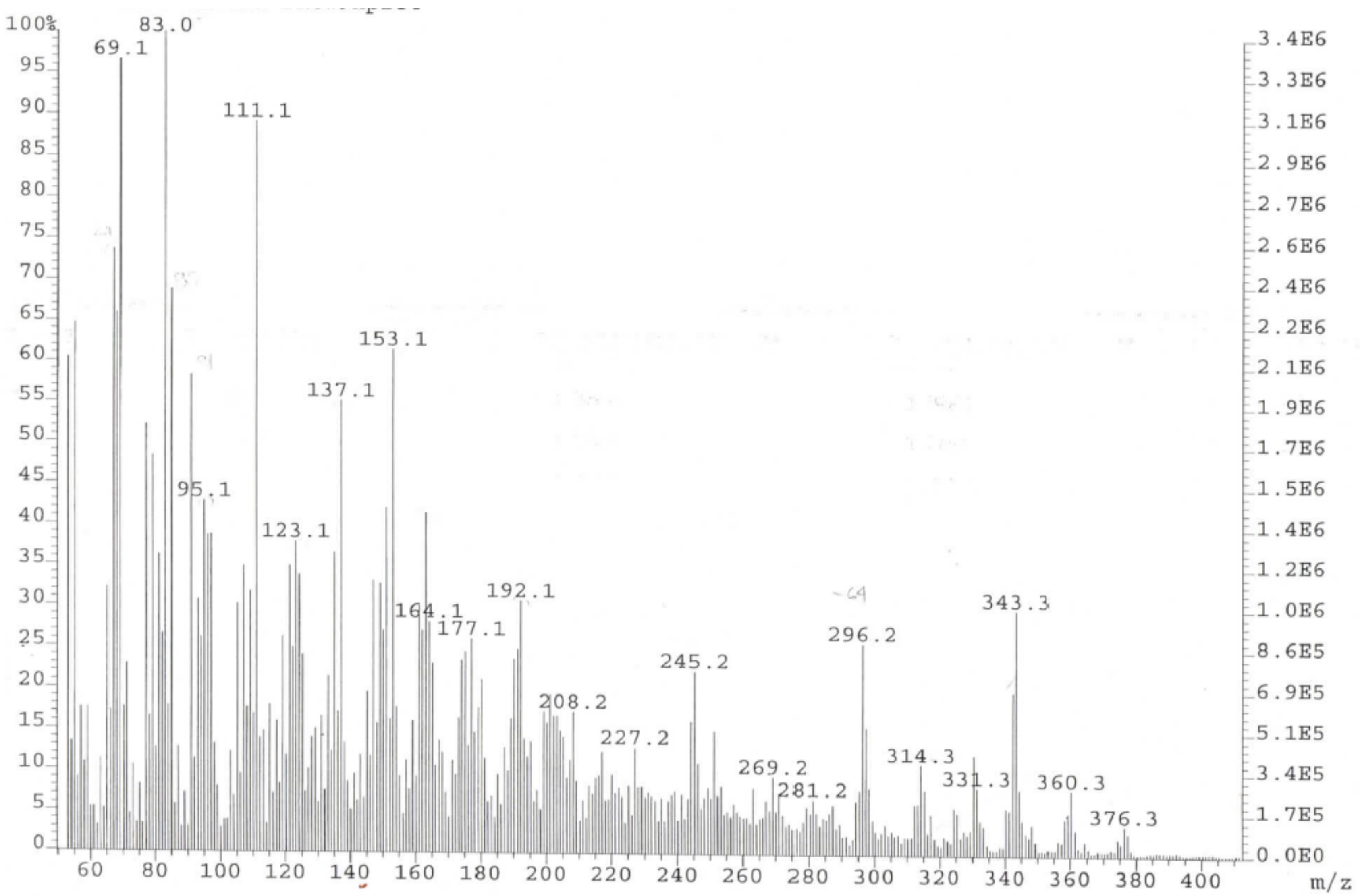

Figure S24. LR-EI-MS spectrum of Kallopterolide E (**5**)

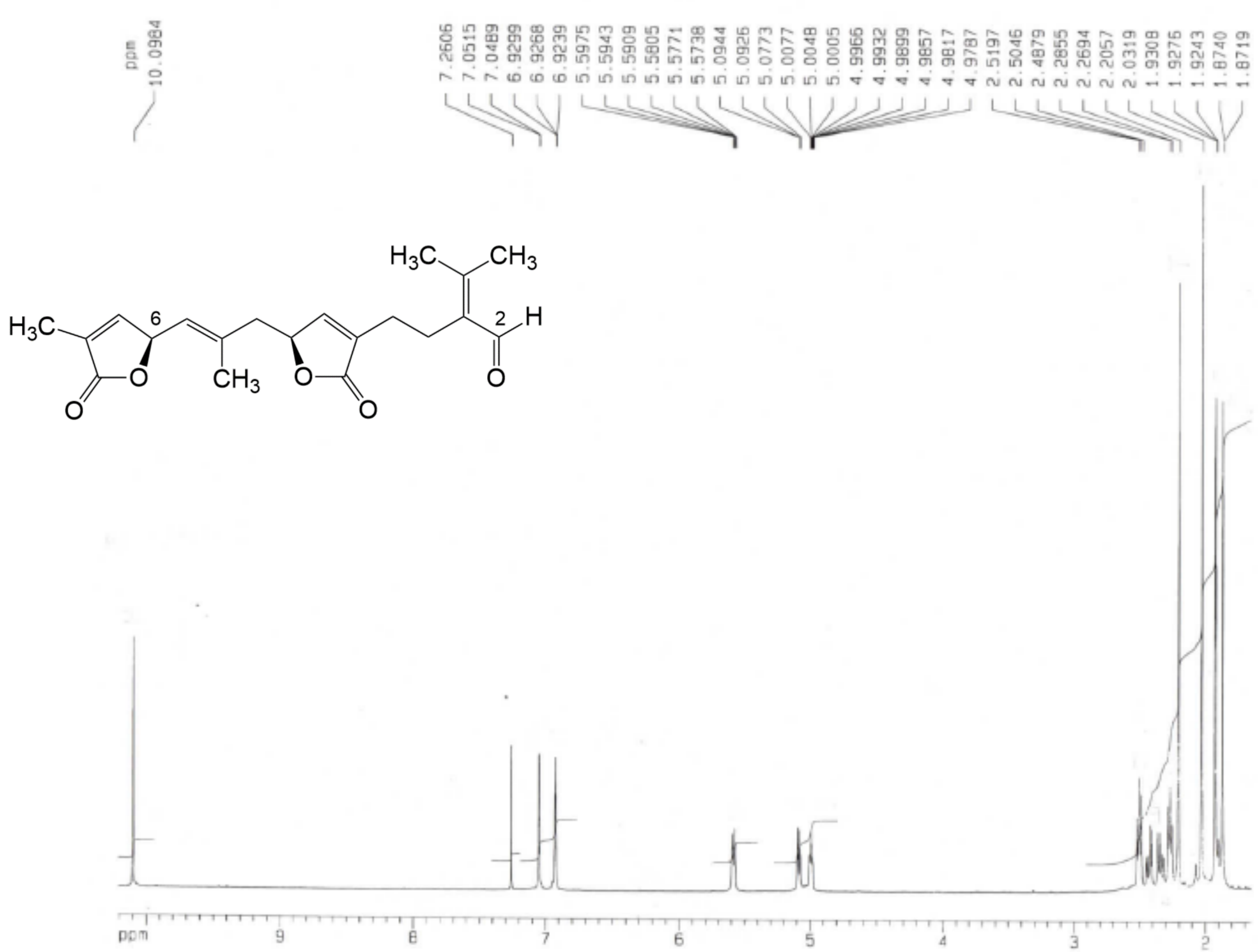

Figure S25. ^{1}H-NMR spectrum ($CDCl_3$, 300 MHz) of Kallopterolide F (**6**)

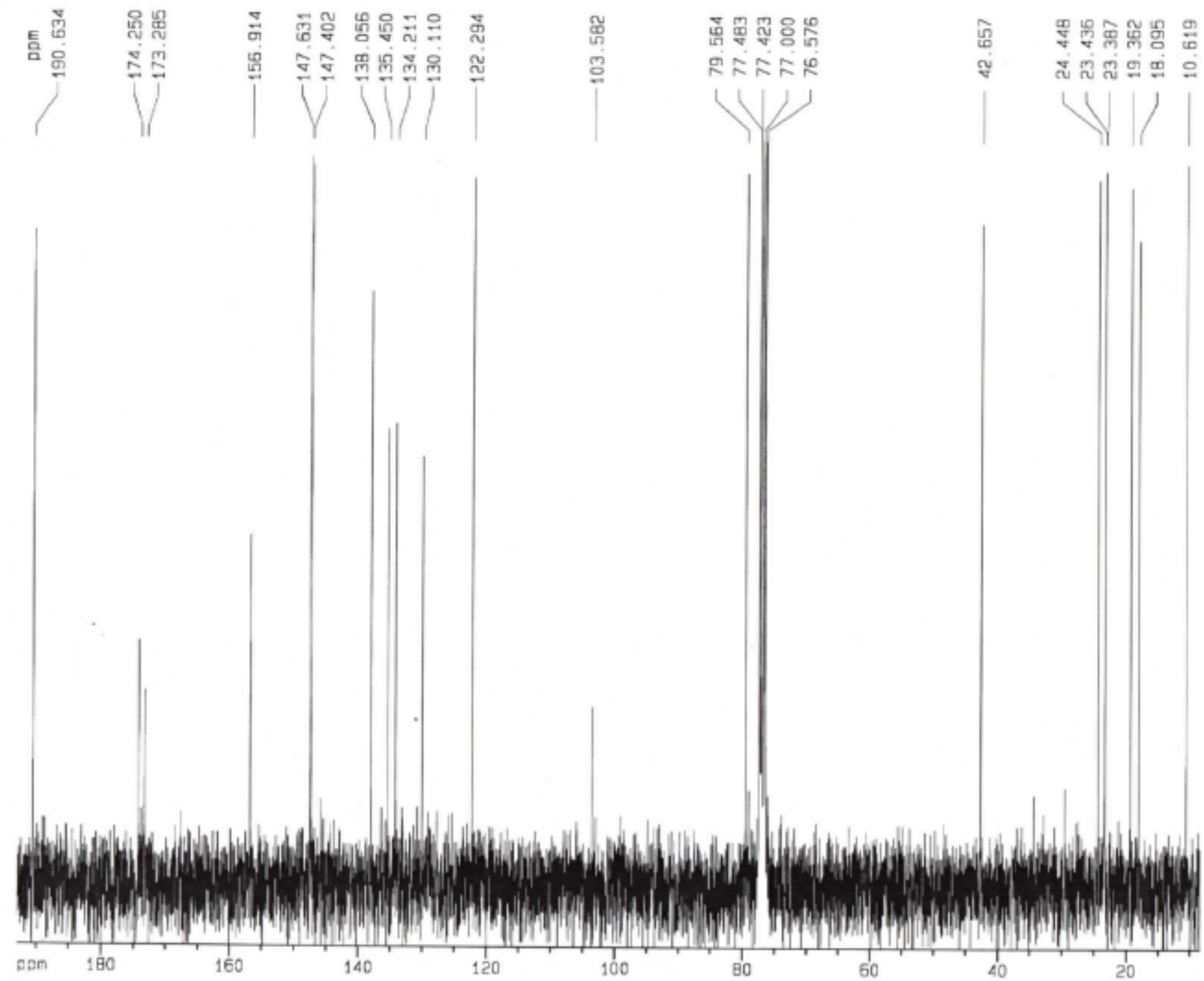

Figure S26. ^{13}C-NMR spectrum ($CDCl_3$, 75 MHz) of Kallopterolide F (**6**)

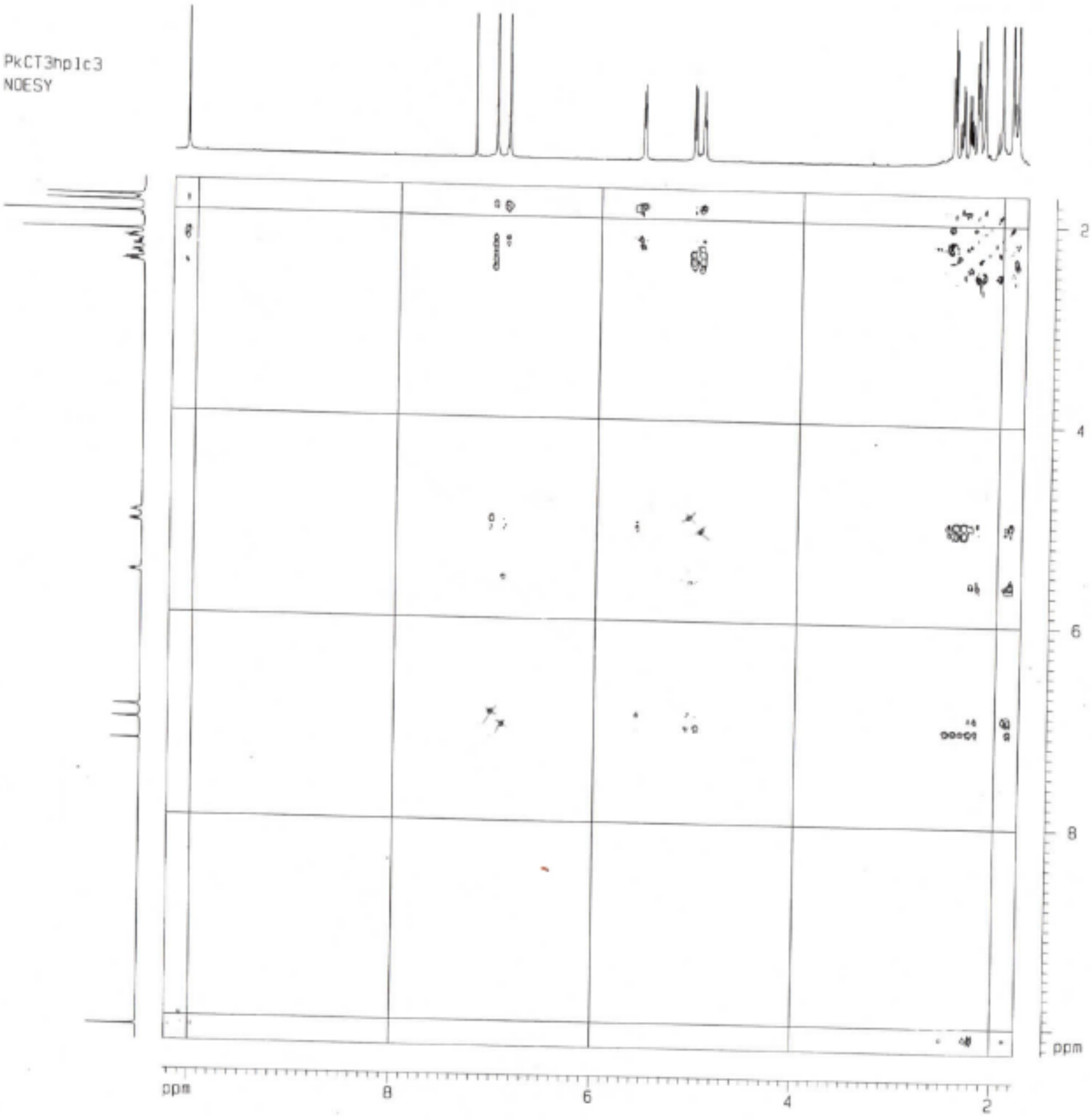

Figure S27. ^{1}H-^{1}H-NOESY spectrum ($CDCl_3$) of Kallopterolide F (**6**)

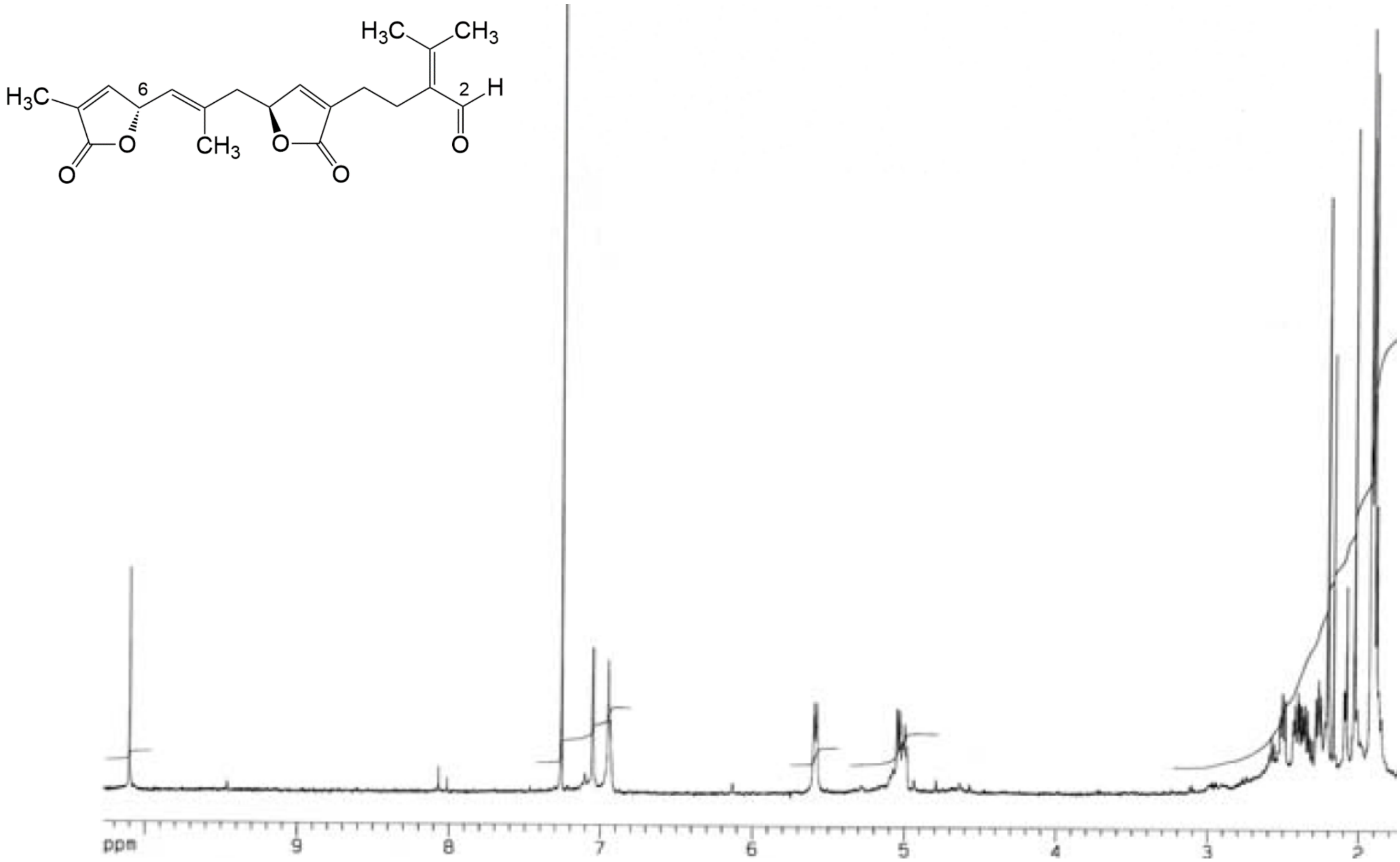

Figure S28. ^{1}H-NMR spectrum ($CDCl_3$, 300 MHz) of Kallopterolide G (**7**)

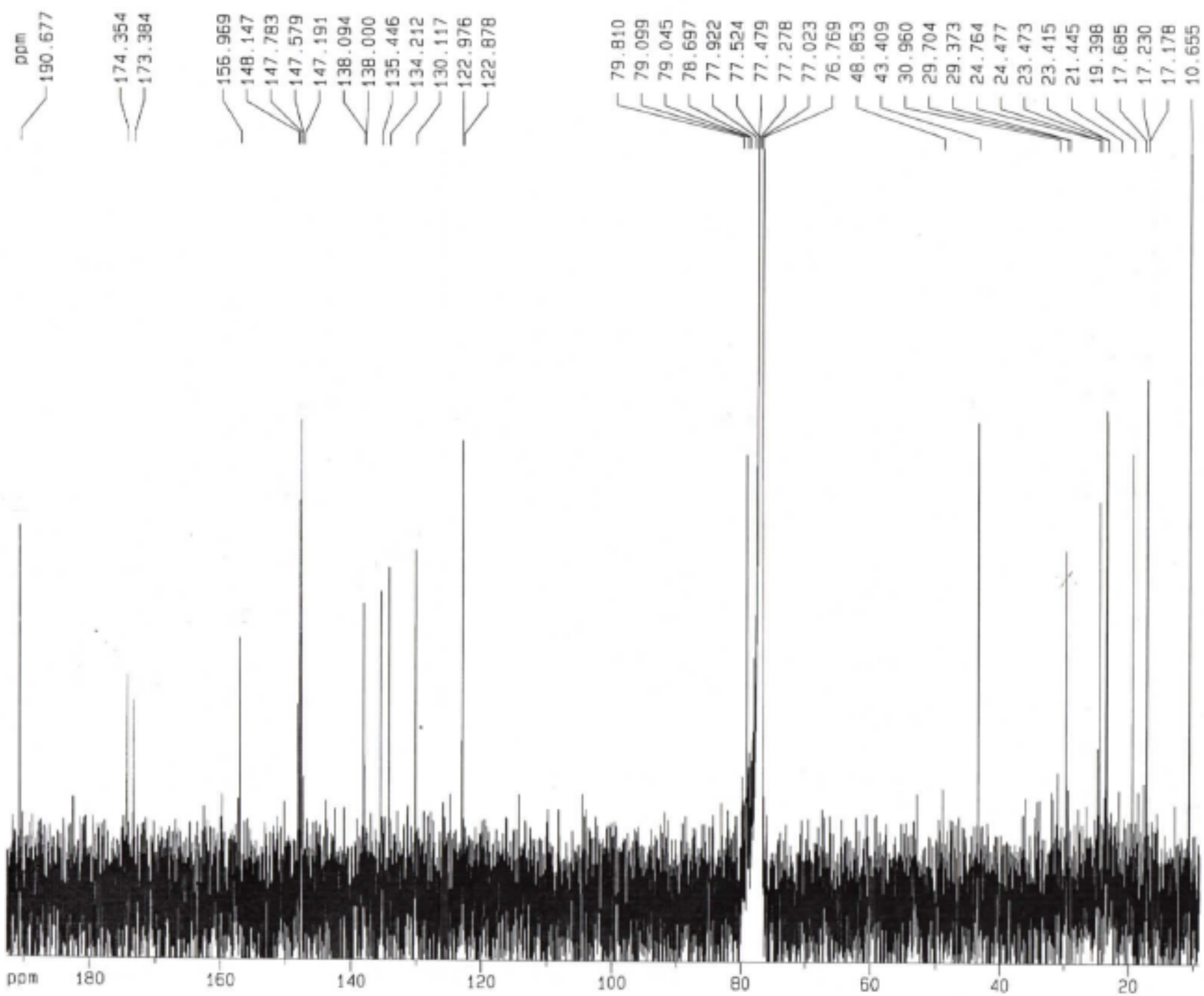

Figure S29. ^{13}C-NMR spectrum ($CDCl_3$, 75 MHz) of Kallopterolide G (**7**)

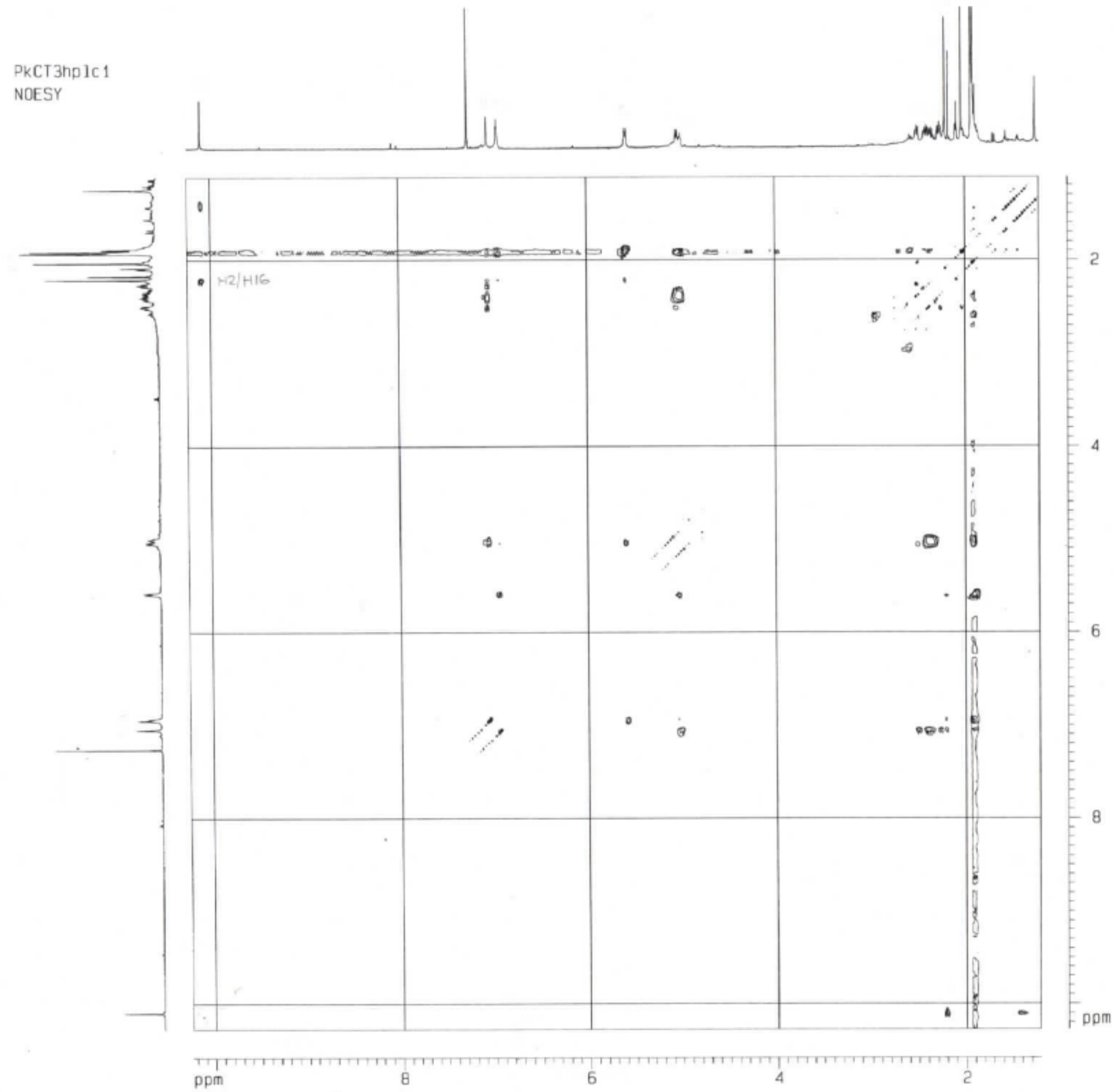

Figure S30. ^1H-^1H-NOESY spectrum ($CDCl_3$) of Kallopterolide G (**7**)

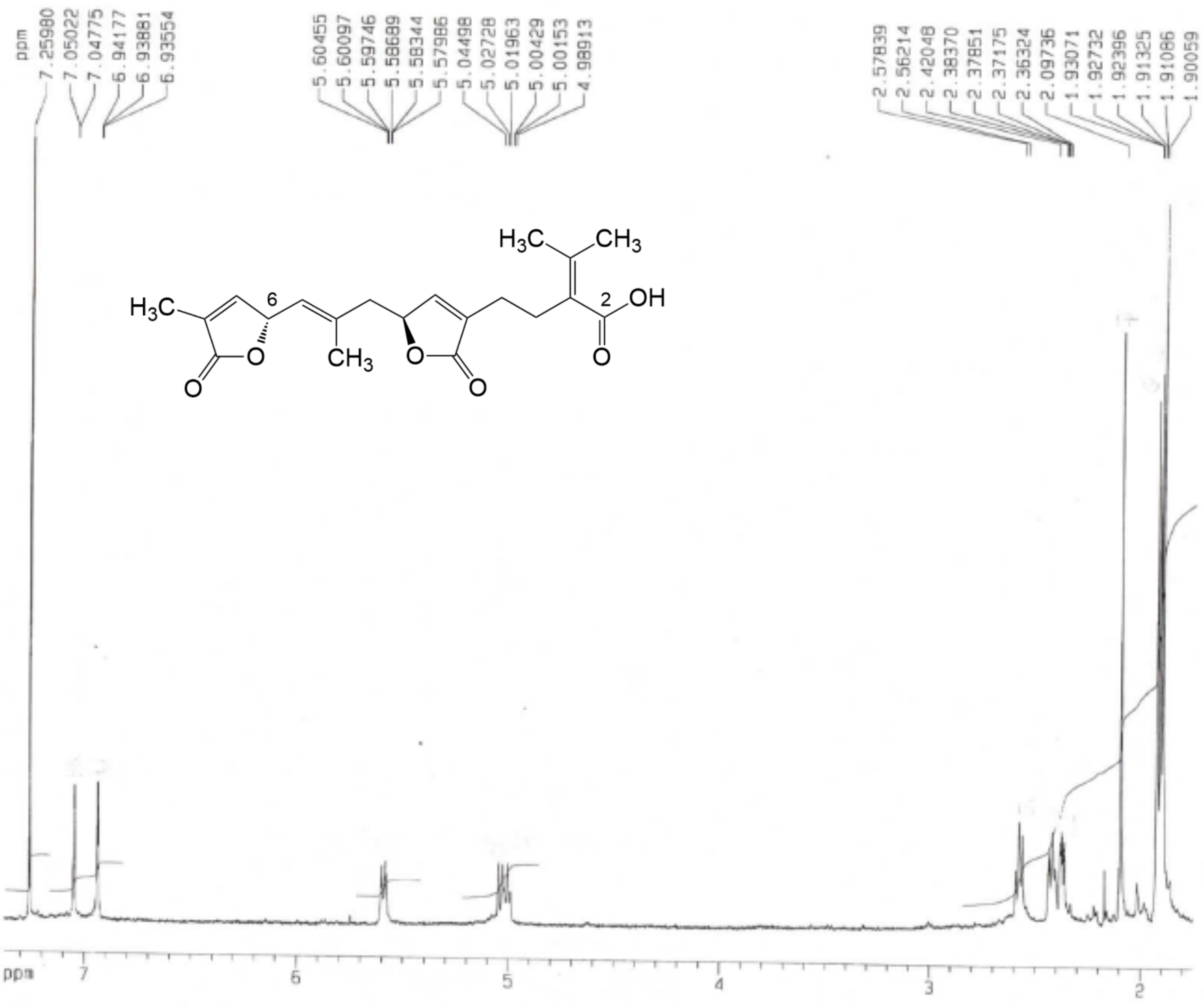

Figure S31. ^{1}H-NMR spectrum ($CDCl_3$, 300 MHz) of Kallopterolide H (**8**)

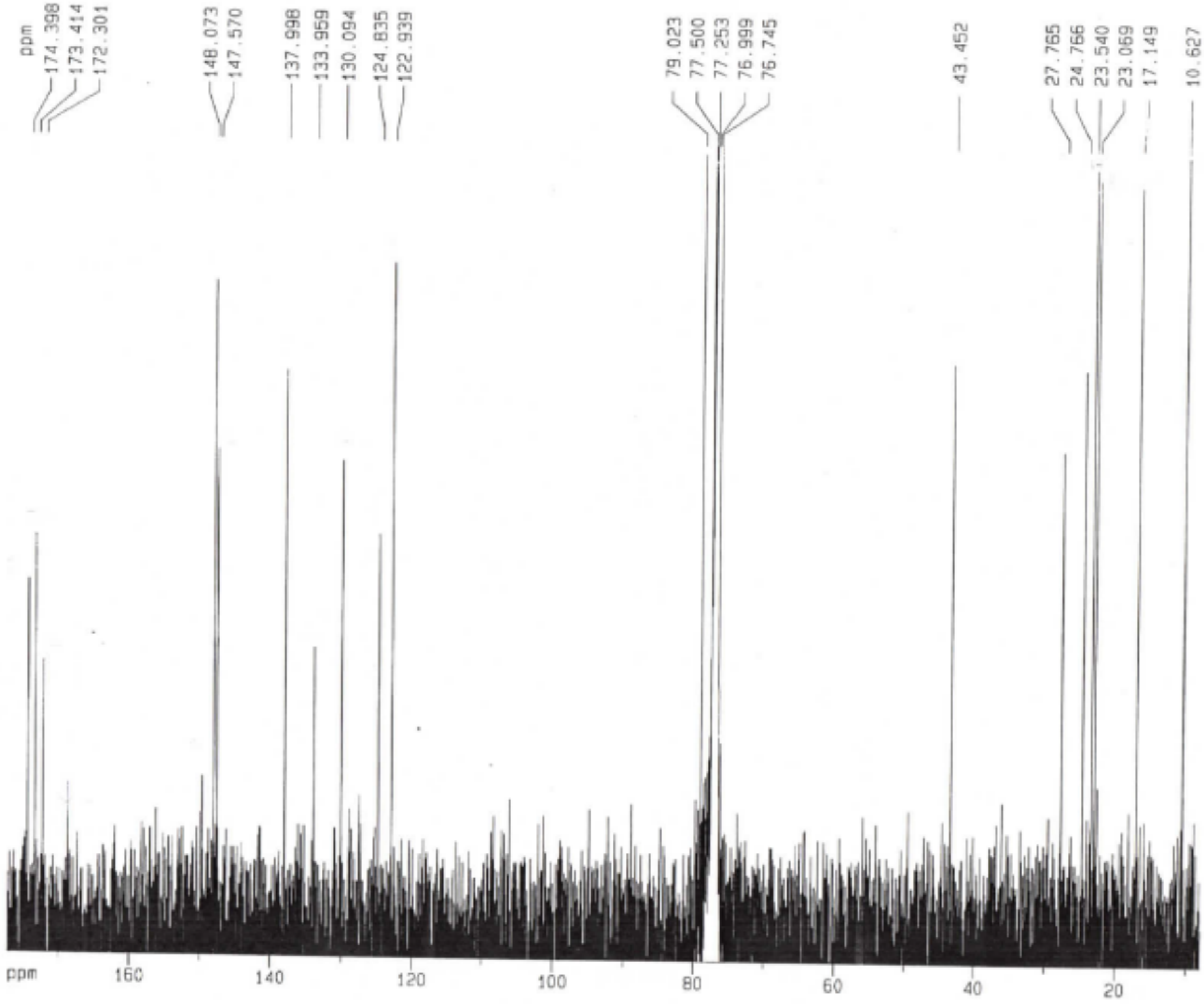

Figure S32. ^{13}C-NMR spectrum ($CDCl_3$, 75 MHz) of Kallopterolide H (**8**)

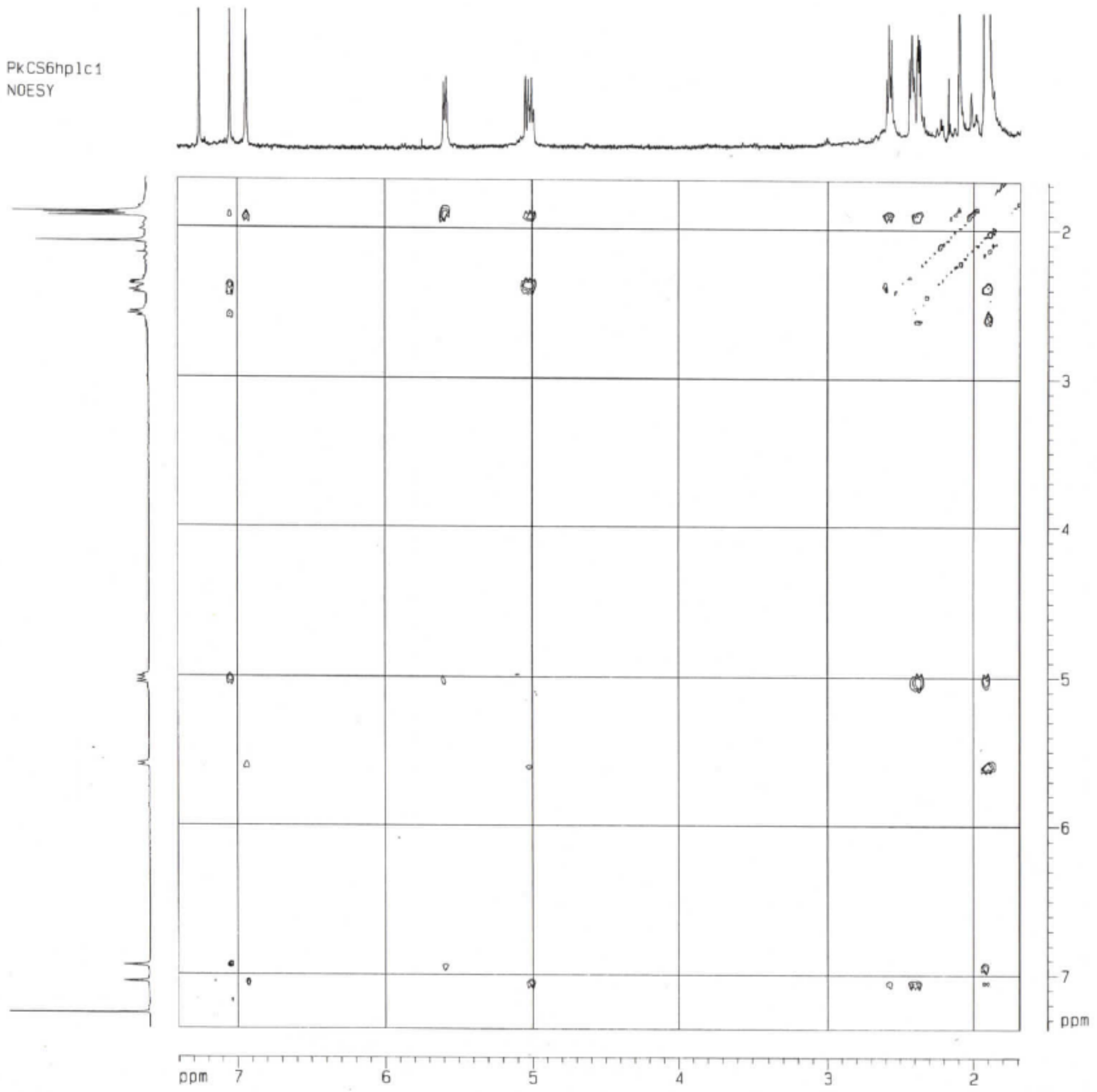

Figure S33. ^{1}H-^{1}H-NOESY spectrum ($CDCl_3$) of Kallopterolide H (**8**)

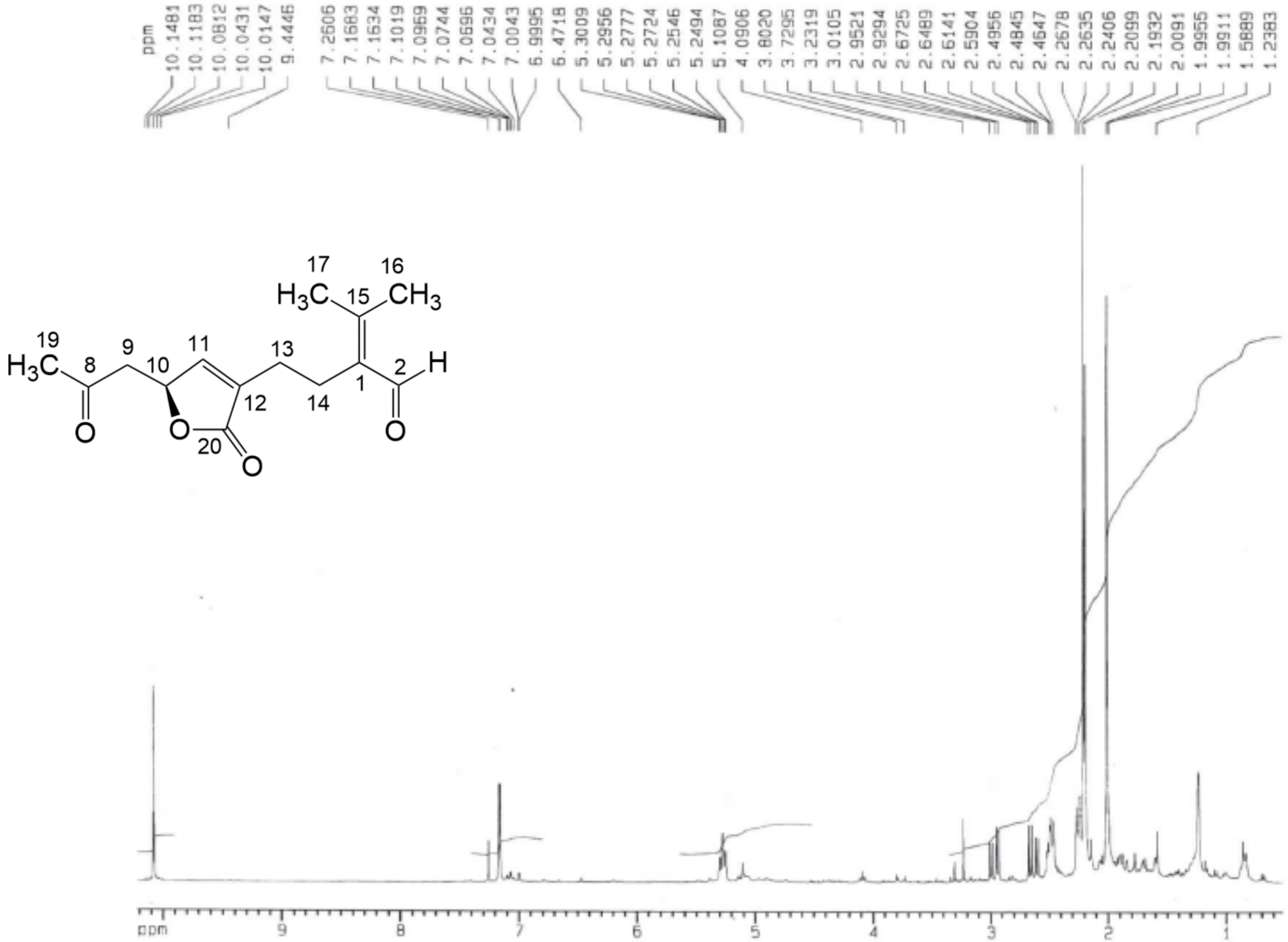

Figure S34. ^{1}H-NMR spectrum ($CDCl_3$, 300 MHz) of Kallopterolide I (**9**)

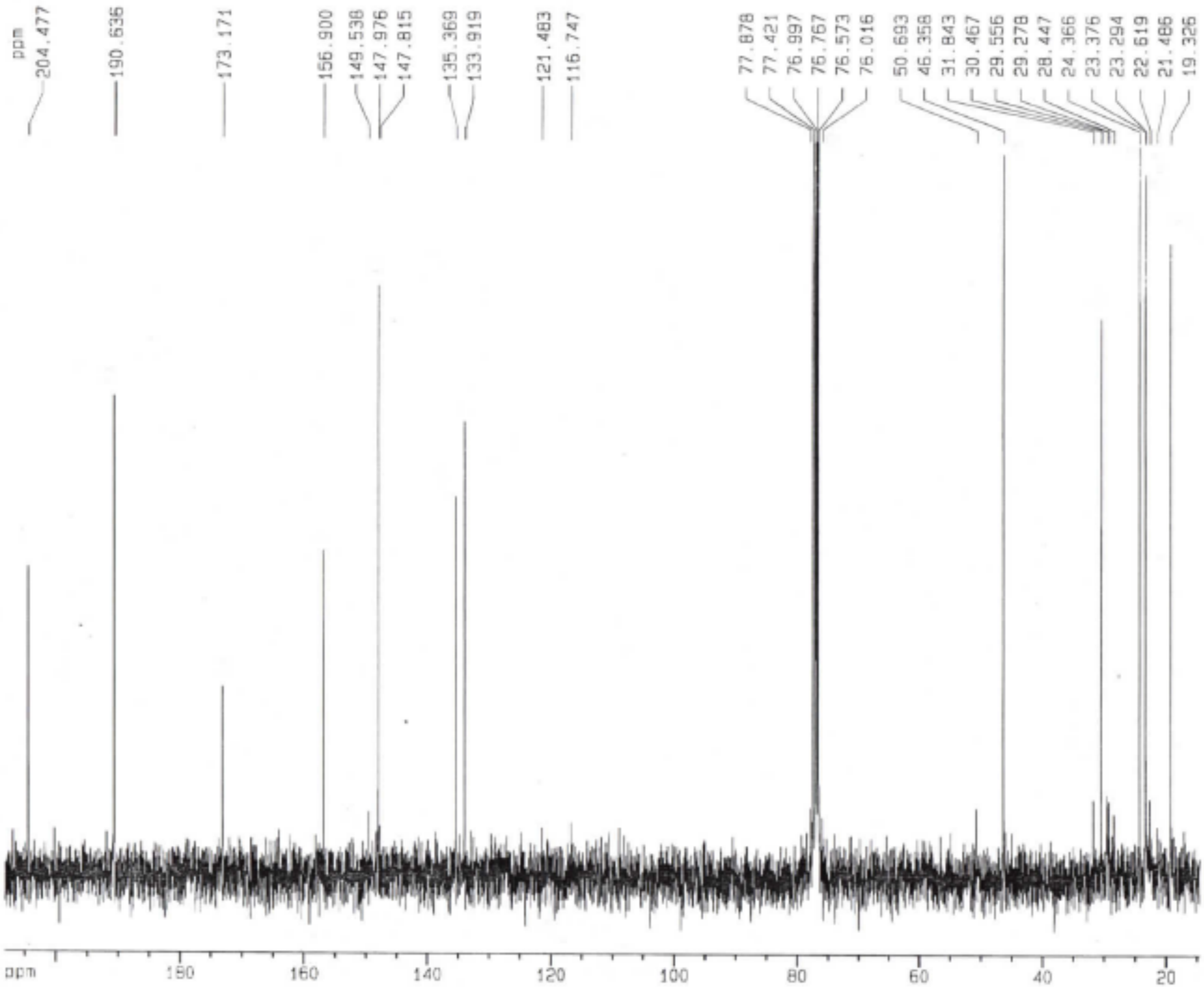

Figure S35. ^{13}C-NMR spectrum ($CDCl_3$, 75 MHz) of Kallopterolide I (**9**)

Table S1. ^{1}H NMR (500 MHz) spectroscopic data for kallopterolide A (**1**) in CD_3OD.[a]

	kallopterolide A (1)
**atom**	**δ_H, mult, intgrt (*J* in Hz)**
1	
2	10.13, s, 1H
3	
4	
5	7.31, dd, 1H (1.62, 1.60)
6	5.35, ddd, 1H (3.91, 2.00, 1.87)
7	2.41, dd, 1H (9.96, 4.08)
8	5.23 ddd, 1H (9.99, 1.63, 1.52)
9α	7.24, dd, 1H (1.53, 1.43)
9β	
10	
11	2.27, m, 2H (12.7, 6.8)
12	2.52, m, 2H (15.7, 7.9)
13	
14	2.04, s, 3H
15	2.22, br s, 3H
16	1.86, dd (1.78, 1.72)
17	
18α	5.06, br s, 1H
18β	4.88, br s, 1H[b]
19	1.72, br s, 3H
20	

[a]NMR spectra were recorded in CD_3OD at 25°C; 1H and ^{13}C NMR chemical shift values are in ppm and referenced to the residual CD_3OD (δ = 3.31) ppm signals. [b]H-18β was reported as an approximated value due to overlapping with the residual D_2O (δ = 4.79) ppm signal.

Table S2. ^{1}H NMR (500 MHz) and ^{13}C NMR (125 MHz) spectral data for kallopterolide A (**1**), kallopterolide B (**2**), and caucanolide A.[a]

	kallopterolide A (1)[a]		kallopterolide B (2)[a]		caucanolide A[a]	
**atom**	**δ_H, mult, intgrt (*J* in Hz)**	**δ_C (mult)[b]**	**δ_H, mult, intgrt (*J* in Hz)**	**δ_C (mult)[b]**	**δ_H, mult, intgrt (*J* in Hz)**	**δ_C (mult)[b]**
1		135.5 (C)		135.4 (C)		135.5 (C)
2	10.10, s, 1H	190.6 (CH)	10.10, s, 1H	190.7 (CH)	10.10, s, 1H	190.7 (CH)
3		173.8 (C)		173.5 (C)		170.6 (C)
4		130.9 (C)		130.9 (C)		134.7 (C)
5	7.06, dd, 1H (1.2, 1.0)	146.8 (CH)	7.10, dd, 1H (1.6, 1.5)	147.0 (CH)	6.77, br q, 1H (1.6)	145.1 (CH)
6	5.34, ddd, 1H (3.9, 2.0, 1.9)	79.8 (CH)	5.05, dd, 1H (1.7, 1.6)[c]	80.0 (CH)		108.4 (C)
7	2.21, dd, 1H (10.0, 4.3)	53.5 (CH)	2.60, dd, 1H (7.0, 7.0)	52.8 (CH)	2.78, d, 1H, (5.8)	57.3 (CH)
8	5.16, ddd, 1H (10.3, 1.4, 1.0)	80.1 (CH)	5.07, m, 1H[c]	79.9 (CH)	5.25, dd 1H (5.8, 1.4)	79.8 (CH)
9	7.09, dd, 1H (1.6, 1.2)	147.4 (CH)	7.13, d, 1H (1.4)	146.8 (CH)	7.20, br d, 1H (1.4)	148.4 (CH)
10		134.6 (C)		134.6 (C)		133.7 (C)
11	2.29, m, 2H	24.5 (CH_2)	2.28, m, 2H	24.6 (CH_2)	2.25, br t, 2H (7.9)	24.5 (CH_2)
12	2.52, m, 2H	23.4 (CH_2)	2.52, m, 2H	23.4 (CH_2)	2.47, br t, 2H, (7.9)	23.6 (CH_2)
13		156.8 (C)		156.9 (C)		156.8 (C)
14	2.03, s, 3H	23.4 (CH_3)	2.03, s, 3H	23.4 (CH_3)	2.02, s, 3H	23.4 (CH_3)
15	2.21, s, 3H	19.4 (CH_3)	2.21, s, 3H	19.4 (CH_3)	2.20, s, 3H	19.4 (CH_3)
16	1.92, d (1.7)	10.8 (CH_3)	1.94, d (1.6)	10.7 (CH_3)	1.99, d, 3H (1.6)	10.6 (CH_3)
17		137.8 (C)		139.2 (C)		138.1 (C)
18α	4.89, br s, 1H	117.1 (CH_2)	4.83, br s, 1H	116.8 (CH_2)	4.90, br s, 1H	118.8 (CH_2)
18β	5.07, br s, 1H		5.08, br s, 1H		5.05, br s, 1H	
19	1.72, br s, 3H	23.7 (CH_3)	1.81, br s, 3H	23.6 (CH_3)	1.76, s, 3H	23.9 (CH_3)
20		172.9 (C)		173.0 (C)		173.3 (C)
21					3.21, s, 3H	51.0 (CH_3)

[a]NMR spectra were recorded in $CDCl_3$ at 25°C; ^{1}H and ^{13}C NMR chemical shift values are in ppm and referenced to the residual $CHCl_3$ (δ = 7.26) or $CDCl_3$ (δ = 77.0) ppm signals. [b] ^{13}C NMR multiplicities were deduced from a DEPT NMR experiment. [c]Chemical shifts and ^{1}H–^{1}H coupling constant values are approximated due to second-order effects.

Table S3. ^{1}H NMR (500 MHz) and ^{13}C NMR (125 MHz) spectral data for kallopterolide D (**4**), caucanolide E, kallopterolide E (**5**) and caucanolide F.[a]

	kallopterolide D (4)[a]		caucanolide E[a]		kallopterolide E (5)[a]		caucanolide F[a]	
atom	δ_H, mult, intgrt (*J* in Hz)	δ_C (mult)[b]	δ_H, mult, intgrt (*J* in Hz)	δ_C (mult)[b]	δ_H, mult, intgrt (*J* in Hz)	δ_C (mult)[b]	δ_H, mult, intgrt (*J* in Hz)	δ_C (mult)[b]
1		135.5 (C)		135.5 (C)		135.5 (C)	101.10, s, 1H	135.5 (C)
2	10.10, s, 1H	192.7 (CH)	10.10, s, 1H	190.7 (CH)	10.10, s, 1H	190.6 (CH)		190.7 (CH)
3		170.0 (C)		170.6 (C)		170.0 (C)		170.5 (C)
4		129.3 (C)		129.6 (C)		129.5 (C)	7.01, br q, 1H (1.3)	129.7 (C)
5	7.05, dd, 1H (1.5, 1.0)	138.6 (CH)	7.05, br q, 1H (1.4)	138.8 (CH)	7.02, dd, 1H (1.5, 1.0)	138.5 (CH)		138.7 (CH)
6		146.9 (C)		147.9 (C)		146.0 (C)		147.9 (C)
7	5.43, s, 1H	118.3 (CH)	5.26, s, 1H	115.9 (CH)	5.37, s, 1H	119.2 (CH)	5.11, s, 1H	116.8 (CH)
8		71.8 (C)		76.0 (C)		72.0 (C)		76.1 (C)
9α	2.11, dd, 1H (14.6, 4.3)	45.8 (CH_2)	2.08, br m, 1H	44.7 (CH_2)	2.40, dd, 1H (14.7, 3.5)	45.1 (CH_2)	2.05, br m, 1H	43.2 (CH_2)
9β	1.92, dd, 1H (14.6, 8.3)		2.08, br m, 1H		1.85, dd, 1H (14.6, 5.3)		2.20, br m, 1H	
10	5.15, ddd, 1H (8.2, 4.2, 2.6)	80.9 (CH)	5.09, ddd, 1H (7.0, 5.5, 1.5)	78.0 (CH)	5.07, ddd, 1H (5.1, 3.4, 1.7)	78.8 (CH)	5.09, m, 1H	77.9 (CH)
11	7.14, dd, 1H (2.8, 1.4)	149.6 (CH)	7.13, br d, 1H (1.5)	149.7 (CH)	7.12, dd, 1H (1.6, 1.2)	149.1 (CH)	7.08, br d, 1H, (1.2)	149.5 (CH)
12		133.1 (C)		132.9 (C)		133.2 (C)		133.1 (C)
13	2.26, m, 2H	24.4 (CH_2)	2.25, t, 2H, (7.9)	24.4 (CH_2)	2.26, m, 2H	24.4 (CH_2)	2.25, t, 2H (7.9)	24.4 (CH_2)
14	2.50, m, 2H	23.4 (CH_2)	2.50, t, 2H (7.9)	23.4 (CH_2)	2.49, m, 2H	23.3 (CH_2)	2.50, t, 2H, (7.9)	23.5 (CH_2)
15		156.7 (C)		156.7 (C)		156.8 (C)		156.8 (C)
16	2.20, s, 3H	19.4 (CH_3)	2.20, s, 3H	19.3 (CH_3)	2.20, s, 3H	19.4 (CH_3)	2.20, s, 3H	19.4 (CH_3)
17	2.01, s, 3H	23.4 (CH_3)	2.01, s, 3H	23.4 (CH_3)	2.01, s, 3H	23.4 (CH_3)	2.02, s, 3H	23.5 (CH_3)
18	2.01, s, 3H	10.5 (CH_3)	2.01, d, 3H (1.4)	10.5 (CH_3)	2.00, s, 3H	10.5 (CH_3)	2.01, d, 3H (1.3)	10.5 (CH_3)
19	1.55, s, 3H	30.1 (CH_3)	1.57, s, 3H	24.4 (CH_3)	1.53, s, 3H	28.9 (CH_3)	1.61, s, 3H	23.3 (CH_3)
20		173.5 (C)		173.7 (C)		173.2 (C)		173.7 (C)
21			3.26, s, 3H	50.7 (CH_3)			3.25, s, 3H	50.7 (CH_3)

[a]NMR spectra were recorded in $CDCl_3$ at 25°C; ^{1}H and ^{13}C NMR chemical shift values are in ppm and referenced to the residual $CHCl_3$ (δ = 7.26) or $CDCl_3$ (δ = 77.0) ppm signals. [b] ^{13}C NMR multiplicities were deduced from a DEPT NMR experiment.

Table S4. ^{1}H NMR (500 MHz) and ^{13}C NMR (125 MHz) spectral data for kallopterolide F (**6**), caucanolide D, and kallopterolide G (**7**).[a]

	**kallopterolide F (6)**		**Caucanolide D**		**kallopterolide G (7)**	
**atom**	**δ_H, mult, intgrt (*J* in Hz)**	**δ_C (mult)[b]**	**δ_H, mult, intgrt (*J* in Hz)**	**δ_C (mult)[b]**	**δ_H, mult, intgrt (*J* in Hz)**	**δ_C (mult)[b]**
1		135.5 (C)		135.5 (C)		135.5 (C)
2	10.10, s, 1H	190.7 (CH)	10.11, s, 1H	190.6 (CH)	10.10, s, 1H	190.7 (CH)
3		174.3 (C)		171.8 (C)		174.4 (C)
4		130.1 (C)		131.9 (C)[c]		130.1 (C)
5	6.93, dd, 1H (1.6, 1.5)	147.5 (CH)	6.80, br q, 1H (1.4)	145.3 (CH)[c]	6.95, dd, 1H (1.5, 1.0)	147.6 (CH)
6	5.59, ddq, 1H (8.6, 1.7, 1.6)	77.5 (C)		106.6 (C)	5.59, ddq, 1H (8.6, 1.8, 1.7)	77.5 (C)
7	5.08, dd, 1H (8.6, 1.0)	122.3 (CH)	5.41, br q, 1H, (1.0)	123.5 (CH)[c]	5.04, dd, 1H (8.6, 1.7)	122.9 (CH)
8		138.1 (C)		139.9 (C)[c]		138.1 (C)
9α	2.43, dd, 1H (14.7, 5.4)	42.7 (CH_2)	2.36, dd, 1H, (14.8, 3.3)	43.8 (CH_2)	2.36, m, 2H	43.4 (CH_2)
9β	2.34, dd, 1H (14.7, 7.7)		2.39, dd, 1H (14.8, 2.8)			
10	4.98, ddd, 1H (7.3, 5.5, 1.6)	79.6 (CH)	5.00, br, 1H	79.4 (CH)	5.02, m, 1H	79.1 (CH)
11	7.05, d, 1H (1.3)	147.7 (CH)	7.05, br d, 1H (1.1)	147.6 (CH)[c]	7.05, d, 1H (1.3)	147.8 (CH)
12		134.2 (C)		134.3 (C)		134.2 (C)
13	2.27, m, 2H	24.5 (CH_2)	2.26, t, 2H (7.8)	24.5 (CH_2)	2.27, m, 2H	24.5 (CH_2)
14	2.50, m, 2H	23.4 (CH_2)	2.51, t, 2H (7.8)	23.4 (CH_2)	2.51, m, 2H	23.4 (CH_2)
15		157.1 (C)		156.9 (C)		157.0 (C)
16	2.21, s, 3H	19.4 (CH_3)	2.21, s, 3H	19.4 (CH_3)	2.21, s, 3H	19.4 (CH_3)
17	2.03, s, 3H	23.5 (CH_3)	2.03 s, 3H	23.5 (CH_3)	2.03, s, 3H	23.5 (CH_3)
18	1.93, d, 3H (1.6)	10.7 (CH_3)	1.94, d, 3H (1.4)	10.4 (CH_3)	1.93, d, 3H (1.5)	10.7 (CH_3)
19	1.87, d, 3H (1.0)	18.1 (CH_3)	1.91, br d, 3H (1.0)[c]	18.1 (CH_3)[c]	1.91, d, 3H (1.7)	17.2 (CH_3)
20		173.3 (C)		173.3 (C)		173.4 (C)
21			3.33, s, 3H	51.5 (CH_3)		

[a]NMR spectra were recorded in $CDCl_3$ at 25°C; ^{1}H and ^{13}C NMR chemical shift values are in ppm and referenced to the residual $CHCl_3$ (δ = 7.26) or $CDCl_3$ (δ = 77.0) ppm signals. [b] ^{13}C NMR multiplicities were deduced from a DEPT NMR experiment. [c]Signal appeared as two closely spaced lines of lower intensity.
